# Toxicity during induction of pulsed versus continuous prednisolone in children with acute lymphoblastic leukaemia: a multi-centre, open label, randomised, phase 3 trial from India (2016–2022)

**DOI:** 10.1016/j.lansea.2026.100788

**Published:** 2026-06-07

**Authors:** Parag Das, Nandana Das, Gaurav Narula, Amita Trehan, Sameer Bakhshi, Venkatraman Radhakrishnan, Rachna Seth, Prashant Tembhare, Man Updesh Singh Sachdeva, Anita Chopra, Shirley Sundersingh, Soumyadeep Das, Manash Pratim Gogoi, Mayur Parihar, Rahul Bhattacharya, Shripad Banavali, Vaskar Saha, Shekhar Krishnan

**Affiliations:** a Clinical Research Unit, Tata Translational Cancer Research Centre, Tata Medical Center, Kolkata, West Bengal, 700160, India; b Department of Pediatric Oncology, Tata Memorial Centre, Tata Memorial Hospital, Mumbai, Maharashtra 400012, India; c Homi Bhabha National Institute, Mumbai, Maharashtra, 400 094, India; d Pediatric Hematology-Oncology Unit, Department of Pediatrics, Advanced Pediatrics Center, Postgraduate Institute of Medical Education and Research, Chandigarh, 160012, India; e Department of Medical Oncology, BR Ambedkar Institute Rotary Cancer Hospital, All India Institute of Medical Sciences, New Delhi, 110029, India; f Department of Medical Oncology, Cancer Institute (WIA), Adyar, Chennai, Tamil Nadu 600020, India; g Oncology Division, Department of Pediatrics, All India Institute of Medical Sciences, New Delhi, 110029, India; h Hematopathology Laboratory, ACTREC, Tata Memorial Centre, Homi Bhabha National Institute, Mumbai, Maharashtra, 410210, India; i Department of Hematology, Postgraduate Institute of Medical Education and Research, Chandigarh, 160012, India; j Laboratory Oncology, All India Institute of Medical Sciences, New Delhi, 110029, India; k Department of Oncopathology, Cancer Institute (WIA), Adyar, Chennai, Tamil Nadu 600020, India; l Department of Statistics, Bidhannagar College, Kolkata 700064, India; m Cytogenetics Department, Tata Medical Center Kolkata, West Bengal, 700160, India; n Department of Statistics, University of Calcutta, Kolkata 700019, India; o Department of Paediatric Haematology and Oncology, Tata Medical Center, Kolkata, West Bengal, 700160, India; p Division of Cancer Sciences, School of Medical Sciences, Faculty of Biology, Medicine and Health, University of Manchester, Manchester, M20 4GJ, UK

**Keywords:** Acute lymphoblastic leukaemia, Child, Steroid, Randomised clinical trial

## Abstract

**Background:**

Despite the use of similar treatment protocols, induction treatment-related mortality (TRM) in children with ALL in India is 5%, compared to <1% in high income countries. This study evaluated pulsed prednisolone during induction to reduce toxicity.

**Methods:**

ICiCLe-ALL-14 (CTRI/2015/12/006434), an open-label, randomised, multicentre trial. Children ≤ 10-years with newly diagnosed B-cell precursor ALL, risk-stratified to standard- or intermediate-risk, were randomised (1:1) to receive prednisolone 60 mg/m^2^/day, for 4 weeks with taper (R1A) or on days 1–14 and 22–28 (R1B) during induction. Primary outcomes were grade 3–5 toxicities. Secondary outcomes were complete remission (CR) rates, measurable residual disease (MRD) at end of induction (EoI), event-free (EFS), and overall survival (OS).

**Findings:**

Of 3315 patients screened at 6 centres, 2505 were eligible and 1246 randomised (623 per group). Thirty (2.4%) patients died during induction: 22/623 (3.5%) and 8/623 (1.3%) in R1A and R1B respectively (absolute risk difference 2.3%, 95% CI 0.54–4.1; p = 0.0149). Relative risk of induction death was 2.75 (95% CI 1.2–6.1) for R1A versus R1B. Grade 3–5 toxicities occurred in 46.4% (289/623) and 45.4% (283/623) of patients (p = 0.7762). CR rates were 98.8% (581/588) and 98.0% (594/606); MRD ≥ 0.01% at EoI was 26.2% and 27.9%; 3-year EFS 72.7% (95% CI 68.2–76.7) and 72.2% (67.6–76.3); OS 85.0% (81.5–87.8) and 87.0% (83.6–89.3) in R1A and R1B respectively. Multivariable analysis confirmed higher risk of induction death with continuous prednisolone (HR 3.06, 95% CI 1.4–6.9, p = 0.0069) and anthracycline use (HR 5.44, 95% CI 1.6–18.9 p = 0.0077).

**Interpretation:**

Pulsed prednisolone (R1B) during induction significantly reduced treatment-related deaths, particularly early deaths from infections compared to continuous dosing (R1A), without compromising CR rates, MRD clearance, EFS, or OS. Anthracycline use was an independent risk factor for induction mortality. Higher white cell count and anthracycline use independently predicted grade 3–4 toxicity.

**Funding:**

This study was funded partly by the 10.13039/501100001411Indian Council of Medical Research (Reference 79/159/2015/NCD-III), the National Cancer Grid (Reference 2016/001), DBT-Wellcome India Alliance (Margdarshi IA/M/12/1/500261) and a centre grant from the Tata Consultancy Services Foundation to the trial centre.

**Research in context**

**Evidence before this study**

We previously reported that treatment-related mortality (TRM) ranged from 11% to 38% when centres in India treated children with acute lymphoblastic leukaemia (ALL) using protocols from high-income countries (HICs). Modern ALL regimens use integrated risk stratification incorporating clinical characteristics, somatic genetic abnormalities, and measurable residual disease (MRD) at end-induction to tailor treatment intensity and reduce relapse risk. The Indian Collaborative Childhood Leukaemia (ICiCLe) group implemented a consensus risk-stratified protocol, leading to a reduction in overall TRM to 11% over a 5-year period, with significantly lower TRM in standard-risk (SR) patients compared with intermediate- (IR) and high-risk (HR) groups. Nearly half (45%) of TRMs occurred during induction therapy. Two contemporary randomised trials have explored reducing induction intensity to limit toxicity. The United Kingdom ALL 2011 compared dexamethasone 6 mg/m^2^/day for 28 days versus 10 mg/m^2^/day for 14 days. There were 16 (0.8%) induction deaths in 1897 patients, with 6 (0.6%)/948 and 10 (1.1%)/949 (p = 0.45) deaths in long and short steroid arms respectively. Grade 3–4 toxicities occurred in 242 (26%)/948 and 227 (34%)/949 (p = 0.41) respectively. The Associazione Italiana di Ematologia e Oncologia Pediatrica-Berlin Frankfurt Münster (AIEOP-BFM) 2009 clinical trial evaluated two versus four weekly doses of daunorubicin (30 mg/m^2^) during induction in non-high risk B-cell precursor ALL patients who were either *ETV6::RUNX1* positive or had a rapid treatment response (<0.1% lymphoblasts on a day 15 bone marrow). Fourteen (0.7%) induction deaths were reported in 2039 patients, with 6 (0.6%)/1016 and 8 (0.8%)/1023 deaths in the two-dose and four-dose arms respectively. There were no significant differences in rates of neutropenia and sepsis between the two arms. In patients receiving four daunorubicin induction doses, a higher incidence of fungal infections (3-fold) and cerebral sinus venous thrombosis (6-fold) was reported. Both the UKALL and AIEOP-BFM studies reported comparable survival in the randomised arms (Supplementary Table S16).

**Added value of this study**

The ICiCLe group introduced India’s first MRD- and genetics-based risk-stratified ALL protocol across six centres in 2012. In an observational cohort (2012–2018), this strategy allowed reduced-intensity therapy for low-risk patients, lowering TRM and treatment costs. Overcoming regulatory and financial hurdles, the group subsequently launched the first multicentre randomised clinical trial for children with cancer in 2016. Although trial participation and randomisation were novel concepts, families quickly accepted and embraced them. Despite using the same protocol as the prior observational study, overall TRM declined further from 11% to 8%. All centres shared in the progressive TRM reduction, underscoring the importance of collaboration, protocol standardisation, and context-specific modifications to minimise toxicity. Nevertheless, induction mortality remained nearly 4-fold higher than reported in HICs. Deaths typically occurred from approximately two weeks after therapy initiation, when hematopoietic recovery is yet to begin and patients are profoundly neutropenic. Sepsis was the most common severe adverse event and the leading cause of death. A recent Swedish study also reported a high frequency of infection during induction, with 97% (335/345) requiring hospitalisation, but a low induction TRM at ∼1%. Postulating that the prolonged use of steroids increases the risk of infection in induction, this study demonstrates that a pulsed steroid schedule significantly decreases induction TRM without adversely affecting early treatment response or survival. Prolonged steroid exposure combined with anthracycline use increased fungal infections, echoing findings of the AIEOP-BFM trial. Unlike the AIEOP-BFM study, toxicity was comparable with two versus four doses of daunorubicin in induction. Younger children were particularly vulnerable to complications from prolonged steroid use, anthracycline treatment and intensive consolidation (BFM Protocol 1 b).

**Implications of all the available evidence**

Although infection and sepsis rates in modern ALL protocols are comparable between HICs and low and middle-income countries (LMICs), sepsis-related mortality is considerably higher in LMICs. This could be due to a higher prevalence of drug-resistant pathogens and limited supportive care resources. Reducing intensity of induction therapy in LMICs is therefore a priority. While the benefits of a pulse steroid schedule in UKALL 2011 may have been offset by the higher dexamethasone dosing, both that trial and the present study show that shortening steroid duration does not compromise outcomes. Importantly, in our trial, a shorter steroid course significantly decreased induction TRM.

## Introduction

Over the past decade, survival rates for childhood acute lymphoblastic leukaemia (ALL) in high-income countries (HICs) have approached 90%, driven by risk-adapted protocol-based therapy and collaborative research networks, in contrast, similar regimens in low- and middle-income countries (LMICs) reported survival that were typically ≥20% lower.^1^ A major contributor is the lack of effective risk stratification in LMICs, resulting in uniform intensive therapy to all patients. In settings with hot, humid climates and crowded living conditions, this approach heightens the risk of opportunistic infections and leads to excess treatment-related mortality (TRM).^2^ Risk stratification in LMICs helps identify patients curable with less intensive therapy, reducing TRM without compromising disease control.^3^, ^4^, ^5^, ^6^ In an observational cohort, we demonstrated that risk stratified therapy for childhood ALL significantly decreased TRMs,^7^ and was very cost effective (WHO-CHOICE criteria).^8^ In this cohort, the cumulative incidence of deaths was 11%, with 45% of deaths occurring in the first 4–6 weeks of therapy (induction). Although toxicity data were not systematically collected, sepsis was the most common cause of death and TRM significantly lower among standard-risk (SR) patients who did not receive anthracyclines in induction.^7^

Alongside anthracyclines, vincristine, and L-asparaginase, corticosteroids are a core component of induction therapy and are also immunosuppressive. In randomised trials, dexamethasone improved event-free survival (EFS) compared with prednisolone but was associated with a significantly higher risk of death, largely due to infections.^9^ Continuous corticosteroid for 4–5 weeks during induction remains standard, yet this duration is based on historical precedent rather than high-level evidence. In relapsed ALL, pulsed corticosteroid regimens have been effective, suggesting that continuous corticosteroid exposure may not be essential for therapeutic efficacy.^10^^,^^11^

In the observational cohort, along with TRMs, relapse rates were higher than expected across all risk categories, reflecting multiple contributing factors and limiting options for treatment reduction. Maintaining the successful collaborative, risk-adapted framework designed by the Indian Childhood Collaborative Leukaemia (ICiCLe) group, we designed the ICiCLe-ALL-14 randomised trial to test whether shortening the duration of corticosteroid exposure during induction for children with non–high-risk B-cell precursor (BCP) ALL could reduce induction TRM and toxicity while maintaining remission rates and long-term survival outcomes.

## Methods

ICiCLe-ALL-14 (CTRI/2015/12/006434) was an open-label, randomised, multicentre clinical trial conducted from October 24, 2016, to August 15, 2022. The trial recruited children with newly diagnosed ALL from six Indian paediatric oncology centres (Supplementary). This was an open-label trial; neither participants, care providers, nor outcome assessors were blinded to treatment allocation due to the nature of the interventions. The data centre responsible for statistical analyses was blinded to group allocation until the database was locked and recruitment completed.^12^

Children and adolescents aged 1–18 years with a confirmed diagnosis of ALL, determined through microscopy and flow cytometry, were eligible. Participants were required to be previously untreated, except for limited prior exposure to prednisolone (≤7 days or >7 days if prednisolone response data were available), a single dose of vincristine, or a single dose of intrathecal methotrexate. Exclusion criteria included mature B-cell leukaemia, MYC rearranged B-cell precursor (BCP) ALL, Down syndrome, and mixed phenotype acute leukaemia. A secure web-based Remote Data Entry and Decision Support System (RDES) was used to assess eligibility, enrol, randomise and record trial and treatment-related data through electronic case report forms (eCRFs) linked to unique patient trial numbers. Sex was self-reported by participants or their guardians, with options limited to male and female. Consent was obtained in a staged manner. Initial consent for clinical management and study participation was obtained at registration and during the 7-day prednisolone prophase. On day 8, after provisional risk stratification, consent for continued trial participation was reaffirmed. For participants aged <10 years stratified as standard-risk (SR) or intermediate-risk (IR) ALL,^7^^,^^12^ additional consent was sought for the first randomisation (R1). Participants and their guardians could choose to participate in the trial alone or in both the trial and randomisation. Designated trained staff obtained informed consent and assent, as appropriate.

Randomisation used a permuted block design with variable block sizes and 1:1 allocation, stratified by treatment centre. Randomisation was performed centrally through the RDES on treatment day 8, ensuring concealment for the data centre. Participants aged <10 years with SR or IR ALL were randomised to one of two induction arms, R1A: Standard-duration prednisolone (60 mg/m^2^/day for 4 weeks with one week taper; cumulative dose: <2100 mg/m^2^) or, R1B: Pulsed prednisolone (60 mg/m^2^/day as two pulses: days 1–14 and days 22–28; cumulative dose: 1260 mg/m^2^) (Supplementary Fig. S1).

Risk stratification to identify SR, IR, high-risk and T-ALL were performed as reported previously.^7^^,^^12^ Prednisolone response was established by microscopy enumeration of circulating blasts at day 8. DNA ploidy was assessed using flow cytometry, supplemented by fluorescence in situ hybridisation (FISH) screening using fusion (*ETV6::RUNX1*, *BCR::ABL1*, *TCF3::PBX1*) and break-apart (*KMT2A*, *TCF3*, *CRLF2*, *ABL* class) probes. Measurable residual disease (MRD) was assessed at the end of induction (EoI) using 8-10-colour flow cytometry, to a required sensitivity of 0.01%. Induction therapy has been described.^7^^,^^12^ The rationale for comparing standard-duration versus pulsed prednisolone was to assess whether reducing corticosteroid exposure during induction could decrease treatment-related mortality without compromising efficacy. All other aspects of induction therapy were standardised across both groups, and no additional co-interventions were permitted during the induction phase. Institution-specific strategies were implemented to monitor and enhance adherence to treatment and follow-up. Post-therapy, patients were followed up every 6 months, with clinical status recorded in the eCRF. Data accuracy was ensured through automated range, validity, and consistency checks in the database. Implausible or missing data were corrected only after consultation with trial investigators, with all corrections documented.

Primary outcomes were the incidence of (i) TRM and (ii) non-fatal severe toxicities during the induction phase in R1A and R1B groups. Outcomes were assessed centrally to ensure consistency. Adverse events were recorded prospectively and graded according to the National Cancer Institute Common Terminology Criteria for Adverse Events (NCI-CTCAE) version 4.0. Secondary outcomes included complete remission rates (CR, defined as bone marrow blasts <5% at the end of induction and where relevant, remission at extramedullary sites), proportion with EoI MRD ≥ 0.01% (MRD^hi^), and survival (EFS and overall survival [OS]) in the randomised groups.

Adverse events were recorded prospectively throughout induction for the primary harms analysis. Events were graded by site investigators. Serious adverse events (SAEs) were defined as events resulting in death, life-threatening conditions, hospitalisation, or significant disability. All grade 3–4 events requiring hospitalisation or ICU care were systematically captured as SAEs. Causality was assigned by site investigators and centrally reviewed; all deaths and grade 3–4 toxicities were centrally adjudicated.

### Statistical analysis

The target sample size was calculated assuming 50% of enrolled patients would be eligible for randomisation. Based on an anticipated baseline rate of 39 ± 2% for severe treatment-related toxicity (NCI-CTCAE grades 3–5) during induction, and accounting for <5% drop-out, a total enrolment of 3056 patients was projected to provide 764 patients in each randomised arm. This sample size was designed to detect a 7% absolute difference in severe toxicity between arms with 80% power and a two-sided alpha error of 5%.^12^ All deaths and severe toxicities were reviewed by the trial coordinating centre to determine their relationship to study treatment. Recruitment began in October 2016. Recruitment was interrupted by the SARS-CoV-2 pandemic in February 2020 and resumed in January 2022. Recruitment closed in August 2022 due to funding constraints, resulting in fewer randomised participants than planned. Follow-up was censored on 31st March 2024, with a database lock on 30th June 2024. The primary analysis was conducted on a modified intention-to-treat population, excluding ineligible randomised patients. Secondary analyses included per-protocol assessments of patients who received the allocated interventions. Proportions were compared using Fisher’s exact test. Time-to-event outcomes, including EFS and OS, were analysed using Kaplan–Meier with log-rank tests. The cumulative incidence of induction death was estimated using cumulative incidence functions and compared between arms using Gray’s test, with induction treatment discontinuation/abandonment prior to completion of induction specified as the competing event. Cox regression models were used to identify determinants of induction deaths, with proportional hazards assumptions tested. Severe non-fatal induction toxicity was analysed using Poisson regression with overdispersion testing. Missing data for induction deaths were addressed using multiple imputation by chained equations, with pooled estimates following Rubin’s rules under a missing-at-random assumption. The proportion of missing data for primary and secondary outcomes was 2% (27/1246) and was addressed using multiple imputation by chained equations. EFS was defined as the time from day 8 of provisional risk stratification to treatment-related death, relapse, or poor treatment response (M3 marrow at end of induction, M2/M3 marrow until end of consolidation, or disease progression). OS was defined as the time from day 8 of provisional risk stratification to death. CR and EoI MRD^hi^ rates were compared using Fisher’s exact test. All statistical analyses were conducted using R (version 4.4.1), SPSS (version 25), Stata (BE18), and GraphPad Prism (version 9). An independent data monitoring committee oversaw trial conduct and decisions.

### Ethics statement

The ICiCLe-ALL-14 trial protocol (version 5.1, dated 22 January 2020) is registered with the Clinical Trials Registry-India (CTRI/2015/12/006434) and the WHO Trial Registration Data Set is available in the Supplementary data of the protocol publication.^12^ The study protocol was approved by the institutional review boards at each participating centre after approval was obtained by the Institutional Review Board of Tata Medical Center, Kolkata (the coordinating centre) on October 28th 2013 (reference EC/TMC/12/13). Written informed consent was obtained from parents or legally authorised surrogates, and assent sought for participants aged ≥ 8 years.

### Role of the funding source

The sponsor and funding organisations had no role in study design, data collection, data analysis, data interpretation, writing of the report, or the decision to submit for publication.

### Patient and public involvement

Patients and the public were not involved in the design, conduct, reporting, or dissemination of this research.

## Results

**Fig. 1:**
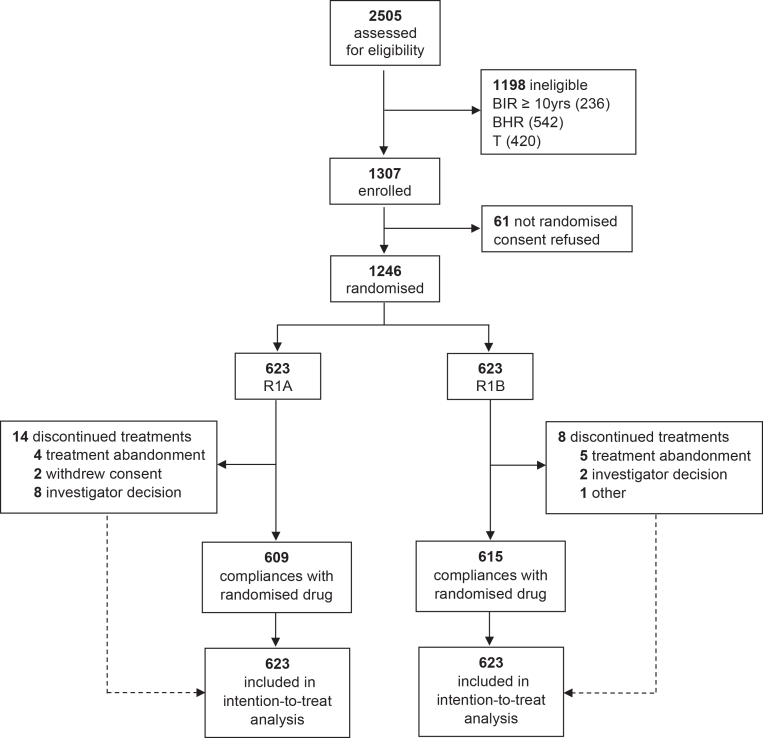
**ICiCLe-ALL-14: CONSORT diagram of patient flow and R1 randomisation.** Eligible for R1 randomisation in Induction: BCP-ALL patients, age < 10 years at diagnosis, with provisional Standard or Intermediate Risk; R1A: standard-duration prednisolone, 60 mg/m2/day, daily, weeks 1–4 with taper on week 5; R1B: pulsed prednisolone, 60 mg/m2/day, weeks 1, 2 and 4, with no taper. SR, IR, HR, Standard-, Intermediate- and High-Risk B cell-precursor acute lymphoblastic leukaemia (BCP-ALL); T, T-lymphoblastic leukaemia/lymphoblastic lymphoma.

Between October 2016 and August 2022, 3315 patients with newly diagnosed ALL were evaluated across six ICiCLe centres. After excluding 810 patients for predefined reasons (Supplementary Table S1) 2505 patients were enrolled. Among these 2085 (83.2%) with BCP-ALL were provisionally risk-stratified on day 8 of induction therapy into SR (842; 40.4%), IR (701; 33.6%) and HR (542; 26.0%). Steroid randomisation was offered at day 8 to 1307 eligible BCP-ALL patients aged <10 years (SR, 842; IR, 465). A total of 1246 patients were randomised 1:1 to receive either standard-duration (R1A, n = 623) or pulsed (R1B, n = 623) prednisolone therapy during induction (Fig. 1). Baseline characteristics including age, sex, highest presentation white cell count (WCC), bulky disease, good risk cytogenetics (*ETV6::RUNX1* and high hyperdiploidy), and provisional risk groups were balanced between arms (Table 1). Median follow-up was 29.2 months (IQR 19.9–42.4) for R1A and 30.2 months (IQR 20.7–41.8) for R1B.

**Table 1:** Patient characteristics of the R1 randomisation cohort.

Randomised arms	R1A	R1B
N (%)	623	623
Age (years)		
Median age with IQR	4.4 (3.0; 6.1)	4.0 (2.8, 5.9)
Sex		
Male	413 (66.3%)	404 (64.8%)
Female	210 (33.7%)	219 (35.2%)
Male:Female	2.0	1.8
Bulky disease		
Yes	132 (21.2%)	137 (22.0%)
No	491 (78.8%)	486 (78.0%)
Presenting WCC (x10^9^/L)		
Median with IQR	12.2 (5.8; 35.0)	12.4 (5.1; 37.2)
<50	500 (80.3%)	492 (79.0%)
≥50	123 (19.7%)	131 (21.0%)
Cytogenetics		
High hyperdiploid	189 (30.3%)	201 (32.3%)
*ETV6::RUNX1*	131 (21.0%)	107 (17.2%)
B other	302 (48.5%)	312 (50.1%)
*BCR::ABL1*^a^	0	1 (0.2%)
*KMT2A*-rearranged^a^	1 (0.2%)	1 (0.2%)
Low hypodiploid^a^	0	1 (0.2%)
Provisional risk		
SR	400 (64.2%)	400 (64.2%)
IR	223 (35.8%)	223 (35.8%)

IQR, interquartile range; WCC, white cell count.

SR, standard risk; IR, intermediate risk.

a High risk cytogenetics identified post randomisation R1A: standard-duration prednisolone; 60 mg/m^2^, daily; 4 weeks & taper, R1B: pulsed prednisolone; 60 mg/m^2^/day, weeks 1, 2 and 4, no taper.

Thirty (2.4%) patients died during induction therapy, across both groups. Induction mortality was significantly higher in R1A (22/623, 3.5%) compared to R1B (8/623, 1.3%) (p = 0.0149, Fisher’s exact test). The absolute risk difference was 2.3% (95% CI: 0.5–4.1), corresponding to 2.3 additional deaths per 100 patients in R1A. The relative risk of induction death was 2.75 (95% CI: 1.2–6.1) in R1A versus R1B. Cumulative incidence of induction deaths was higher in R1A (3.1%, 95% CI: 1.9–4.7%) than R1B (1.3%, 95% CI: 0.6–2.4%, p = 0.0090, Gray’s test).

**Fig. 2:**
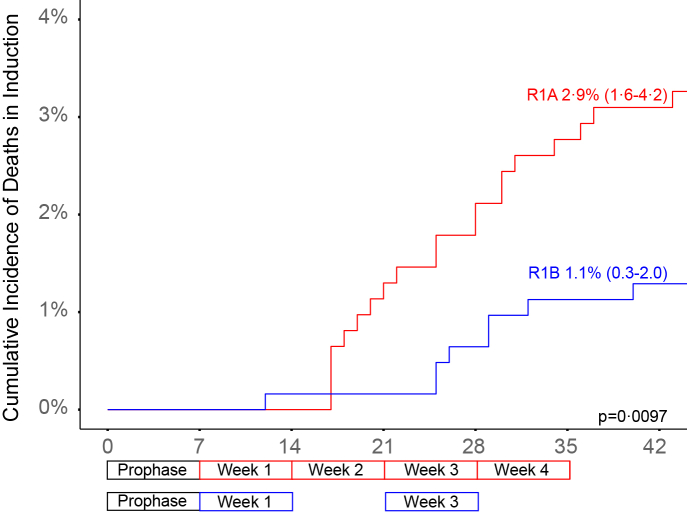
**Cumulative incidence of induction death in the R1 randomised cohort.** Analysis by intention-to-treat; p values based on the Gray-test. X-axis: time from start of steroid prophase, W1-5: weeks 1–5.

When accounting for competing risks such as treatment discontinuation, induction mortality remained higher in R1A compared to R1B (2.9% versus 1.1%; p = 0.0097) (Fig. 2).

Infections were the predominant cause of induction deaths: 21 bacterial, 6 fungal, and 1 viral, with 2 deaths of unknown causes. Fungal deaths were reported in patients receiving a combination of standard-duration steroid and anthracyclines. Timing differed between groups: R1A deaths began after the second week of treatment (coinciding with the administration of the second anthracycline dose in the IR group) and continued through induction and consolidation, whereas R1B deaths were later, clustering within a 7-day period between the third and fourth weeks (Fig. 2). Grade 3–4 toxicities were reported in 267 (42.9%) of 623 and 275 (44.1%) of 623 patients in R1A and R1B respectively. No significant differences were observed between groups for combined deaths and severe toxicities (CTCAE Grades 3–5; p = 0.7762) or for Grade 3–4 toxicities alone, either overall (p = 0.6892) or by specific toxicity type (Table 2; Supplementary Tables S2 and S3).

**Table 2:** Grade 3–5 toxicities in the randomised cohort.

Randomised arms	R1A	R1B	p
N	623	623	
CTCAE Grades 3-5	289 (46.4%)	283 (45.4%)	0.7762
CTCAE Grade 5	22 (3.5%)	8 (1.3%)	0.0149
CTCAE Grades 3-4	267 (42.9%)	275 (44.1%)	0.6892

CTCAE, common terminology criteria for adverse event.

R1A: standard-duration prednisolone; 60 mg/m2, daily; 4 weeks & taper, R1B: pulsed prednisolone; 60 mg/m2/day, weeks 1, 2 and 4, no taper.

**Fig. 3:**
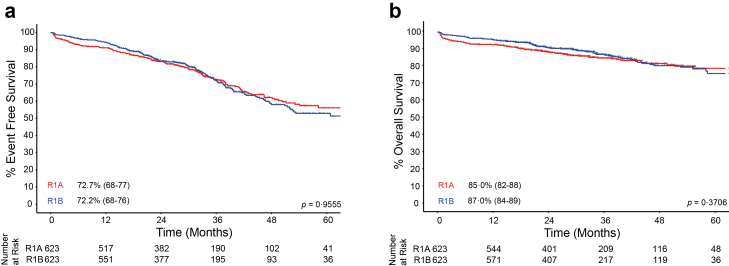
**(a) Event free survival and (b) Overall survival of the randomised cohort.** Analysis by intention-to-treat; p values based on the Log-rank test.

Treatment efficacy was similar between groups. CR rates were 98.8% (581/588) in R1A and 98.0% (594/606) in R1B, with comparable EoI MRD^hi^ rates (26.2% versus 27.9%) respectively (Supplementary Table S4). The 3-year EFS and OS were 72.7% (95% CI: 68–77), 72.2% (95% CI: 68–76) (p = 0.9555, Log-rank test), and 85.0% (95% CI: 82–88), 87.0% (95% CI: 84–89) (p = 0.3706, Log-rank test) for R1A and R1B respectively (Fig. 3a and b).

Multivariable Cox regression confirmed that standard-duration prednisolone was independently associated with increased induction mortality (hazard ratio 3.06, 95% CI 1.4–6.9; p = 0.0069). Anthracycline exposure was another independent risk factor (hazard ratio 5.44, 95% CI 1.6–18.9; p = 0.0077) (Supplementary Table S5) confirmed with per-protocol (Supplementary Table S6) and multiple imputation analysis (Supplementary Table S7).

Subgroup analyses highlighted the interaction between steroid schedule and anthracycline exposure. Deaths were nearly 3 times higher in IR (22/446, 4.9%) compared to SR (p < 0.0001). HR patients, who received four doses of anthracycline and continuous steroids, had induction TRM of 6.1%, comparable to IR patients receiving continuous steroids. In SR patients (who did not receive anthracyclines), induction mortality was higher in R1A (1.8%) compared with R1B (0.3%); this difference reached significance in per-protocol analysis (p = 0.0380). In IR patients (who received two doses of anthracycline), mortality was higher overall (4.9%) and approximately twofold higher in R1A (6.7%) than R1B (3.1%), although not statistically significant (p = 0.1241) (Supplementary Fig. S2).

Grades 3–4 toxicities were higher in IR compared to SR patients (Supplementary Table S8). Multivariable Poisson regression identified high WCC (p = 0.0323) and anthracycline exposure (p = 0.0393) as independent predictors of Grade 3–4 toxicity in induction (Supplementary Tables S9 and S10).

**Fig. 4:**
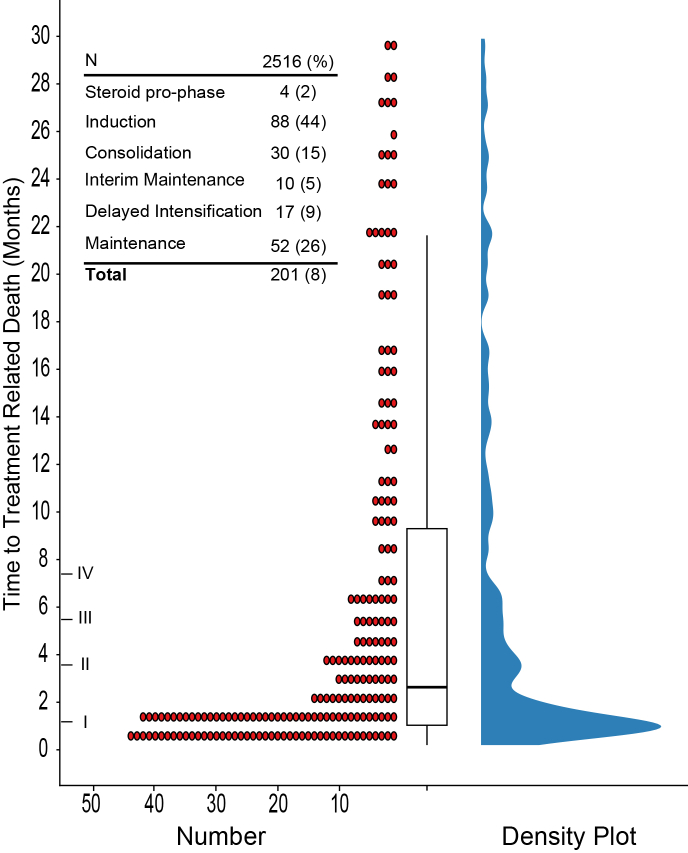
**Temporal distribution of treatment-related deaths in trial-enrolled patients.** Raincloud plot of time from diagnosis to treatment-related death. Each dot represents an individual treatment-related death plotted by time from diagnosis (months). The box-whisker plot indicates the median and interquartile range of time to treatment-related death, with the range indicated by whiskers; the density plot depicts the overall distribution of treatment-related deaths during ICiCLe-ALL treatment. The inset table lists the number and proportion of deaths by treatment phase. I, induction; II, consolidation; III, interim maintenance, IV, delayed intensification.

**Fig. 5:**
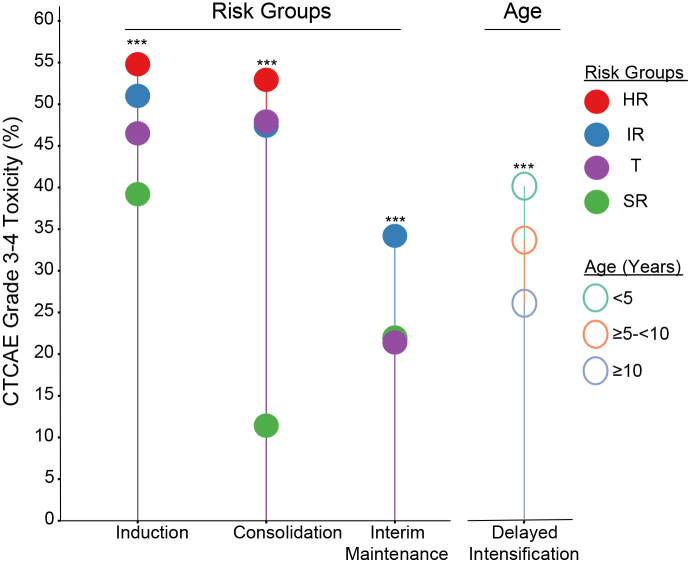
**Lollipop plot representation of NCI-CTCAE grade 3–4 treatment-related toxicity rates among risk groups during each phase of intensive ALL treatment.** For induction, consolidation and interim maintenance, Y-axis represents the CTCAE Grade 3–4 toxicity rate (in percentage) based on risk groups (SR, IR, HR and T). For delayed intensification, Y-axis represents the CTCAE Grade 3–4 toxicity rate (in percentage) based on risk as well as age groups (<5, ≥5–<10 and ≥10 years). SR, IR, HR: standard-, intermediate- and high-risk B cell-precursor ALL; T, T-lymphoblastic leukaemia/lymphoma; ‘∗∗∗’- p value < 0.0001, Chi-square test.

Across the entire duration of treatment (∼2.5 years), 201 (8.0%) TRMs occurred among 2516 patients, with a median time to death of 2.6 months (IQR 1.0–9.3). Of these, 92 (46%) deaths occurred during induction and 122 (61%) within the first three months of therapy. Fifty-two deaths (26%) occurred during the maintenance phase, distributed evenly over time (Fig. 4). Induction deaths differed significantly by risk group (p = 0.0001) (Table 3). Post-induction TRM rates did not differ significantly between risk groups and randomised cohorts (Supplementary Tables S11 and S12 and Supplementary Fig. S3). Median time to death in IR, (1.5 months, IQR 0.9–7.1) and HR, (2.0 months, IQR 1.1–9.6) spanned induction and consolidation, whereas in SR (4.7 months, IQR 1.0–9.7) extended to interim maintenance. This suggests timing and causes of toxicity varied with the intensity of treatment. Grade 3–4 toxicities were highest for all risk groups in induction. SR experienced lower toxicity during induction and consolidation; IR had increased toxicity in interim maintenance related both to sepsis (p = 0.0037) and mucositis (p < 0.0001) likely due to escalating intravenous methotrexate. During delayed intensification with uniform intensity treatment for all risk groups, toxicity was highest in SR (220/520, 42.3%) compared to IR (37.3%, 176/472), HR (32.8%, 241/734) and T-ALL (27.7%, 92/332). Median age was significantly lower in SR (4.2 years) compared to IR (5.7 years), HR (5.1 years) and T-ALL (9.0 years) (p < 0.0001) (Supplementary Table S13) and grade 3–4 toxicity in delayed intensification was significantly higher in those <5-years of age (379/933, 40.6%) compared to ages 5–10 years (238/699, 34%) and ≥10-years (112/426, 26.4%) (p < 0.0001) (Fig. 5).

**Table 3:** Treatment-related deaths in the different risk groups.

Induction (Provisional Risk)	Patients	Deaths (%)
SR	842	11 (1.3%)
IR	701	32 (4.6%)
HR	542	33 (6.1%)
T	420	12 (2.9%)
**Post-Induction (Final Risk)**		

SR	545	23 (4.2%)
IR	506	26 (5.1%)
HR	888	39 (4.4%)
T	392	21 (5.4%)

SR, IR, HR: standard-, intermediate- and high-Risk B cell-precursor ALL; T, T cell lymphoblastic leukaemia/lymphoma.

Overall, SR treatment was associated with significantly lower toxicity risk (p < 0.0001) while younger age, female sex, higher WCC, central nervous system involvement, and induction treatment significantly increased the risk of treatment-related toxicity (Supplementary Table S14). Phase-specific analyses showed increased toxicity with anthracycline during induction, cyclophosphamide-cytarabine in consolidation and escalating intravenous methotrexate administration in interim maintenance for all patients (all p < 0.0001) (Supplementary Table S15).

In summary, pulsed prednisolone during induction significantly reduced treatment-related mortality compared with continuous prednisolone without compromising remission rates, MRD response, or long-term survival. The excess mortality observed with continuous steroids appears to be driven by infection-related deaths, particularly in the context of anthracycline exposure, highlighting steroid scheduling as a modifiable determinant of early treatment mortality in childhood ALL.

## Discussion

Prior to the ICiCLe-ALL-14 observational study,^7^ early deaths during induction were largely attributed to the fact that children had travelled long distances, passed through multiple hospitals, and presented in poor condition, with high disease burden and sepsis at admission.^13^ To address this, the ICiCLe group implemented a 7-day prednisolone prophase, introduced by the Berlin-Frankfurt-Münster (BFM) group^14^ primarily to stabilise patients before commencing full induction therapy, and deferred intrathecal methotrexate administration to day 8. All participating centres were tertiary-level facilities with established supportive and intensive care capacity to manage critically ill patients. Within the trial, induction deaths were more than two-fold higher in the standard-duration group (R1A). The relative risk of 2.75, along with a statistically significant absolute risk difference of 2.3 deaths per 100 treated patients, indicates that continuous steroid exposure may significantly exacerbate early vulnerability to fatal infections. This effect persisted in multivariable models, where R1A remained an independent predictor of induction death (HR 3.06). Importantly, CR rates and MRD responses were nearly identical between groups, confirming that modifying steroid scheduling did not impair antileukaemic efficacy. The temporal distribution of deaths provides further mechanistic insight. In R1A, deaths clustered after the second week of therapy, coinciding with the cumulative immunosuppressive burden of continuous steroids plus anthracyclines. By contrast, R1B deaths tended to occur later and over a narrower window. This timing difference suggests that pulsed dosing may partially mitigate early immune suppression by allowing intermittent recovery of host defences.

Anthracycline exposure emerged as a strong independent risk factor for early death (HR 5.44). As anthracyclines were administered only to IR patients during induction, the observation that both SR and IR subgroups experienced higher mortality with continuous prednisolone is noteworthy. In IR patients, induction mortality in R1A was nearly twice that of R1B. In SR patients who received no anthracyclines, per-protocol analysis indicated a significantly higher mortality with R1A. These results suggest that continuous prednisolone independently increases early fatal toxicity, with anthracyclines conferring additional risk. Induction mortality was five-fold lower in children 1–10 years old who received pulsed steroids without anthracycline (p = 0.0380). The findings indicate that reducing induction treatment intensity in young children with standard risk disease yields the greatest mortality benefit.^15^

Toxicity analyses demonstrate that induction remains the most dangerous phase of therapy, accounting for nearly half of all TRM events. The commonest adverse event is sepsis, and most deaths are due to gram negative bacterial infections. In our region multidrug bacterial resistance poses a major challenge,^16^ with the cost of antimicrobials contributing to over half of overall treatment cost.^8^ Although overall grade 3–4 toxicity frequencies were similar between R1A and R1B, the qualitative patterns align with the mortality data: the combination of prolonged steroids and anthracyclines appearing particularly hazardous. Across the entire treatment course, toxicity profiles diverged by risk group as treatment intensity increased. IR patients experienced disproportionate toxicity during interim maintenance with escalating intravenous methotrexate, while SR patients who are younger, experience higher toxicity during delayed intensification. Age emerged as a key determinant, with patients under 5 years experiencing the highest rates of severe toxicity.

Risk-stratified therapy focuses primarily on avoiding treatment intensification in SR/IR patients who have no detectable MRD at EoI. In LMICs, at least half of TRMs occur during induction.^3^^,^^17^^,^^18^ A recent randomised trial, UKALL 2011, assessed the impact of shortened steroid exposure during induction in ALL.^19^ In that study, patients aged 1–25 years were randomised to receive dexamethasone 10 mg/m^2^/day for 14 days or 6 mg/m^2^/day for 28 days, tapered over 7 days. The overall induction TRM for the cohort was 0.6% with no significant differences in grade 3–5 toxicities between arms. A non-significant excess of TRM was observed in younger patients receiving anthracycline-free induction and shortened steroids. It is possible that the higher initial intensity of the short dexamethasone schedule resulted in similar toxicity profiles as the longer, lower-daily-dose standard schedule. In contrast to the UK experience, the UKALL 2011 regimen in Pakistan was associated with a 30% TRM, with 63% of deaths in induction.^20^ These findings underscore the disparity in TRM rates between HICs and LMICs, and the higher toxicity associated with dexamethasone compared to prednisolone.^9^^,^^21^ The AIEOP-BFM 2009 clinical trial randomised low-risk patients to two versus four weekly doses of daunorubicin in induction. Outcomes were comparable with no significant differences in grade 3/4 neutropenia or sepsis, though invasive fungal infections were significantly lower in those who received 2 daunorubicin doses.^22^ In our study, IR patients received two anthracycline doses during induction yet experienced significantly higher rates of grade 3–5 toxicity that extended into post-induction phases. Toxicity was potentially exacerbated by prolonged steroid exposure (R1A). Induction TRMs were comparable in IR patients treated with continuous steroid and HR patients (continuous steroid, four daunorubicin doses), suggesting that reducing daunorubicin dose does not decrease toxicity at this stage of treatment.

This study should be viewed in light of the substantial logistical and operational challenges that were overcome.^2^^,^^23^ This was the first randomised multicentre clinical trial in paediatric cancers in India across a diverse population. Strengths include the large sample size, robust randomisation, and multicentre collaboration across six specialised paediatric oncology centres in India. For most centres, this was the first experience in recruiting patients into a clinical trial involving standard-of-care treatment, and the concept of randomisation required careful explanation to families. Approximately 16% of patients refused consent or declined treatment at registration, often due to the perception that clinical trials are experimental and unsuitable for their child. Those who continued treatment at the study centres but declined randomisation were assigned to the standard arm. Encouragingly, among those providing consent, 95% accepted randomisation. Limitations include relatively short median follow-up, a learning curve for trial conduct and adaptation to human resource shortages. Primarily due to the SARS-CoV-2 pandemic, the final randomised sample size (n = 1246) was lower than the targeted enrolment (n = 1528). The below-target sample size likely limited the ability to detect statistically significant differences in toxicity outcomes, particularly for subgroup analyses. While all deaths were documented, over half of patients had no grade 3–4 toxicities recorded during induction. This probably reflects incomplete toxicity reporting, common during intensive treatment phases with multiple overlapping toxicities, compounded by challenges with toxicity documentation in busy outpatient settings. For example, a sepsis event reported for hospitalised management at one centre may remain unreported at another centre where treatment is outpatient-based. Toxicities also vary by their functional impact – a grade 2 non-ulcerative oral mucositis may require admission for intravenous fluids, yet remain unreported as a significant toxicity. The UKALL 2011 study reported adverse events in only a quarter of patients. Underreporting of adverse events in phase III cancer trials is common, potentially arising from variations in interpretation of CTCAE terminology. To address this a consensus approach has been developed for ALL^24^ and guidelines developed to systematically report toxicities.^25^ Despite these challenges, we achieved 95% adherence to risk stratification and decreased the induction TRM to 3.5% from the 5% reported in the preceding observational study (which followed the same protocol, apart from the randomisation).^7^ Overall TRMs decreased to 8% from 11%,^7^ highlighting the value of collective experience within a consensus-driven collaborative framework.

The treatment landscape for ALL is evolving rapidly, with immunotherapies and cellular therapies promising improved cure rates and reduced toxicity. The bispecific T-cell engager blinatumomab is poised to become standard of care in many HIC settings.^26^ These advances remain largely unavailable or unaffordable for most children with ALL in LMICs. While a humanitarian access programme for blinatumomab exists,^27^ the need for inpatient administration imposes significant costs that further restrict access. In LMICs, high TRM (as high as 25%)^2^^,^^28^ remains a more pressing challenge than relapse. Consequently, treatment protocols must be adapted to regional circumstances, with an emphasis on avoiding TRM. Our study demonstrates that collaborative groups facing similar constraints can successfully conduct randomised clinical trials to develop context-specific solutions. For immuno- or cellular therapy to be effective, disease burden must first be reduced with chemotherapy. Current ALL protocols may be excessively intensive; while TRM and toxicity are manageable in HICs, opportunities for de-escalation are likely to be most relevant in LMIC settings, ideally through international collaboration.

Future global cooperative efforts should prioritise: (1) evaluating whether steroid exposure can be similarly safely reduced in high-risk patients; (2) identifying factors that contribute to the relatively inferior survival outcomes in non-high risk patients in LMICs; (3) exploring the feasibility of delaying, reducing or eliminating anthracycline use, particularly in younger children; and (4) finally, addressing shortcomings related to drug quality,^29^ purity,^30^^,^^31^ adherence,^32^ and other systemic healthcare deficiencies.

In conclusion, the ICiCLe-ALL-14 trial provides evidence that pulsed prednisolone during induction significantly reduces TRM in childhood ALL without compromising early treatment response or survival outcomes. This is a potentially practice-changing modification to frontline therapy, especially in low-resource environments. These findings have clinical relevance and warrant consideration for incorporation into standard induction regimens globally, especially in settings with high baseline TRM.

## Contributors

SB, SaB, AT, GN, VR, RS, SK and VS conceptualised the study, developed methodology and enrolled patients; PT, MS, AC and MP performed laboratory tests; PD, ND, MG, SD, VS and SK curated and analysed data. PD, VS and SK wrote the paper. SK provided supervision for the study and VS was responsible for funding. All authors reviewed and approved the final draft submitted.

## Data sharing statement

Beginning 3 months after and ending 36 months after publication, individual participant data that underlie the results reported in this article after deidentification will be available from the corresponding authors. Study protocol and statistical plan are already available. Post 36 months, data will be available on the institution server and accessed via ttcrc.org but without investigator support other than deposited metadata. Data will be provided to researchers who provide a methodologically sound proposal to enhance the aims of the study which has been approved by an independent review board.

## Declaration of interests

Authors do not have any conflict of interest to declare.

## Appendix A Supplementary data

Supplementary Figs. S1–S3 and Tables S1–S16

ICiCle 14 protocol_Trials

ICiCLe Protocol_ Version 5.1

Statistical Plan v2
