# Supplementary material for: Toxicity during induction of pulsed versus continuous prednisolone in children with acute lymphoblastic leukaemia: a multi-centre, open label, randomised, phase 3 trial from India (2016–2022)
Source: Lancet Reg Health Southeast Asia. 2026 Jun 7;50:100788. doi: 10.1016/j.lansea.2026.100788 (PMC13366301; doi:10.1016/j.lansea.2026.100788)

**Content List: Supplementary Figures and Tables**

**Note on missing data**

1. **Supplementary Table S1:** Patients who did not meet the eligibility criteria or not enrolled
2. **Supplementary Table S2:** Intention-to-treat analysis of the frequency of severe non-fatal toxicity during induction in the R1 randomisation cohort
3. **Supplementary Table S3:** Per-protocol analysis of the frequency of severe non-fatal toxicity during induction in the R1 randomisation cohort
4. **Supplementary Table S4:** Treatment response, Final Risk stratification and Survival Outcomes
5. **Supplementary Table S5:** Intention to Treat analysis of factors influencing risk of induction death in the R1 randomised cohort
6. **Supplementary Table S6:** Per-protocol analysis of factors influencing risk of induction death in the R1 randomised cohort
7. **Supplementary Table S7:** Multiple Imputation analysis of factors influencing risk of induction death in R1 randomised cohort
8. **Supplementary Table S8:** Frequency of severe non-fatal toxicity during induction in R1 randomised provisional risk groups
9. **Supplementary Table S9:** Intention-to-treat analysis of factors influencing risk of non-fatal severe toxicity during induction treatment in the R1 randomised cohort
10. **Supplementary Table S10:** Per-protocol analysis of factors influencing risk of non-fatal severe toxicity during induction treatment in the R1 randomised cohort
11. **Supplementary Table S11:** Induction and post-induction events in the risk stratified groups within treatment arms
12. **Supplementary Table S12:** Per‑protocol induction and post‑induction events by treatment arm
13. **Supplementary Table S13:** Overall cohort characteristics, categorised by final risk group
14. **Supplementary Table S14:** Factors influencing risk of Grade 3-4 toxicities across all intensive phases of treatment
15. **Supplementary Table S15:** Multivariable regression analysis of factors influencing risk of Grades 3-4 toxicity in each of the intensive treatment phases (Induction to Delayed intensification) in ICiCLe-ALL-14 trial patients
16. **Supplementary Table S16:** Comparative Outcomes of Clinical Trials in ALL with randomisations in induction.
17. **Supplementary Figure S1:** ICiCLe-ALL-14_InPOG-15-01 Study Design
18. **Supplementary Figure S2:** Cumulative incidence of treatment-related death during induction within provisional risk groups of the R1 randomised cohort
19. **Supplementary Figure S3:** Treatment-related death in overall cohort based on the risk groups

**ICiCLe-ALL-14: Participating centres**

Cancer Institute (Women’s Indian Association), Chennai

BR Ambedkar Institute Rotary Cancer Hospital, All India Institute of Medical Sciences, New Delhi

Paediatric Oncology Unit, All India Institute of Medical Sciences, New Delhi

Postgraduate Institute of Medical Education and Research, Chandigarh

Tata Memorial Centre, Mumbai

Tata Medical Center, Kolkata (trial centre)

**Missing Data**

The proportion of missing data for outcomes was 2% (27/1,246), reflecting 27 patients who withdrew during the induction phase for whom the primary endpoint, induction TRM (Grade 5 death), could not be ascertained and was therefore classified as missing. For Grade 3–4 induction toxicity, among these 27 withdrawn patients, toxicity CRFs were available for 7 patients and included in the analysis; for the remaining 20 patients (1.6% of the randomised cohort; 13 in R1A and 7 in R1B), toxicity CRFs were not completed and toxicity data were classified as missing.

**Supplementary Table S1: Patients who did not meet the eligibility criteria**

| **Total registered** | 3315 |
| --- | --- |
| **Not meeting the eligibility criteria, n (%)** | 222 (6.7%) |
| Previously treated | 157 |
| Age <1-year or ≥10-year | 36 |
| Mixed phenotype ALL | 18 |
| Down syndrome | 9 |
| Mature B-cell ALL | 2 |

ALL: acute lymphoblastic leukaemia

Two hundred twenty-two patients (6·7%) did not meet study inclusion criteria, 371 (10·6%) declined consent, 206 (5·9%) were registered but did not proceed to enrolment and 11 patients were lost during the prophase.

**Supplementary Table S2: Intention-to-treat analysis of the frequency of severe non-fatal toxicity during induction in the R1 randomisation cohort**

| Randomised arms | **R1A** | **R1B** |  |
| --- | --- | --- | --- |
| N | 623 | 623 | *p* |
| **Overall, CTCAE Grade 3-4** | **267 (42·9%)** | **275 (44·1%)** | 0·6892 |
| Sepsis | 210 (33·7%) | 231 (37·1%) | 0·2361 |
| Hypertension | 59 (9·5%) | 51 (8·2%) | 0·4847 |
| Hyperglycaemia | 3 (0·5%) | 4 (0·6%) | 0·7252 |
| Proximal myopathy | 6 (1·0%) | 7 (1·1%) | 1·0000 |
| Allergy | 4 (0·6%) | 2 (0·3%) | 0·6867 |
| Bleeding | 9 (1·4%) | 6 (1·0%) | 0·6050 |
| Constipation | 16 (2·6%) | 22 (3·5%) | 0·4104 |
| Diarrhoea | 19 (3·0%) | 15 (2·4%) | 0·6026 |
| Encephalopathy | 3 (0·5%) | 3 (0·5%) | 1·0000 |
| Infusion site extravasation | 6 (1·0%) | 2 (0·3%) | 0·2875 |
| Mucositis | 11 (1·8%) | 15 (2·4%) | 0·5530 |
| Neuropathy | 3 (0·5%) | 4 (0·6%) | 1·0000 |
| Pancreatitis | 4 (0·6%) | 3 (0·5%) | 1·0000 |
| Seizure | 9 (1·4%) | 6 (1·0%) | 0·6050 |
| Serum albumin | 2 (0·3%) | 0 | 0·4996 |
| Serum bilirubin | 2 (0·3%) | 1 (0·2%) | 1·0000 |
| Serum creatinine | 0 | 2 (0·3%) | 0·4996 |
| SIADH | 5 (0·8%) | 4 (0·6%) | 1·0000 |
| Thromboembolic event | 5 (0·8%) | 2 (0·3%) | 0·4518 |
| Vomiting | 8 (1·3%) | 8 (1·3%) | 1·0000 |
| Others | 2 (0·3%) | 1 (0·2%) | 1·0000 |
| Intention-to-treat analysis; *p* values, 2-tailed Fisher exact test | | | |
| R1A: standard-duration prednisolone; daily; 4 weeks & taper, 60 mg/m^2^/day | | | |
| R1B: pulsed prednisolone; weeks 1, 2 & 4; 60 mg/m^2^/day | | | |

Patients with Grade 3 (requiring hospital-based management) and Grade 4 (life-threatening) toxicities in each randomised arm, based on the National Cancer Institute Common Terminology Criteria for Adverse Events (NCI-CTCAE version 4·03). ‘Others’ include patients with meningism/aseptic meningitis following intrathecal treatment, and patients with probable treatment-related illness but without definitive toxicity diagnosis.

**Supplementary Table S3: Per-protocol analysis of the frequency of severe non-fatal toxicity during induction in the R1 randomisation cohort**

| Randomised arms | **R1A** | **R1B** |  |
| --- | --- | --- | --- |
| N | 610 | 616 | *p* |
| **Overall** | **267 (43·8%)** | **275 (44·6)** | 0·7739 |
| Sepsis | 210 (34·4%) | 231 (37·5%) | 0·2841 |
| Hypertension | 59 (9·7%) | 51 (8·3%) | 0·4246 |
| Hyperglycaemia | 3 (0·5%) | 4 (0·6%) | 1·0000 |
| Proximal myopathy | 6 (1·0%) | 7 (1·1%) | 1·0000 |
| Allergy | 4 (0·7%) | 2 (0·3%) | 0·4499 |
| Bleeding | 9 (1·5%) | 6 (1·0%) | 0·4497 |
| Constipation | 16 (2·6%) | 22 (3·6%) | 0·4105 |
| Diarrhoea | 19 (3·1%) | 15 (2·4%) | 0·4916 |
| Encephalopathy | 3 (0·5%) | 3 (0·5%) | 1·0000 |
| Infusion site extravasation | 6 (1·0%) | 2 (0·3%) | 0·1763 |
| Mucositis | 11 (1·8%) | 15 (2·4%) | 0·5530 |
| Neuropathy | 3 (0·5%) | 4 (0·6%) | 1·0000 |
| Pancreatitis | 4 (0·7%) | 3 (0·5%) | 0·7246 |
| Seizure | 9 (1·5%) | 6 (1·0%) | 0·4497 |
| Serum albumin | 2 (0·3%) | 0 | 0·2474 |
| Serum bilirubin | 2 (0·3%) | 1 (0·2%) | 0·6229 |
| Serum creatinine | 0 | 2 (0·3%) | 0·4996 |
| SIADH | 5 (0·8%) | 4 (0·6%) | 0·7519 |
| Thromboembolic event | 5 (0·8%) | 2 (0·3%) | 0·2855 |
| Vomiting | 8 (1·3%) | 8 (1·3%) | 1·0000 |
| Others | 2 (0·3%) | 1 (0·2%) | 0·6229 |
| R1A: standard-duration prednisolone; daily; 4 weeks & taper, 60 mg/m*^2^*/day | | | |
| R1B: pulsed prednisolone; weeks 1, 2 & 4; 60 mg/m^2^/day | | | |
| Per-protocol analysis; *p* values, 2-tailed Fisher exact test | | | |

**Supplementary Table S4: Treatment response, Final Risk stratification and Survival Outcomes**

| Randomised arms | R1A | R1B |  |
| --- | --- | --- | --- |
| N | 623 | 623 | *p* |
| Response assessed | 588 (94·4%) | 606 (97·3%) |  |
| Non-CR | 7 (1·2%) | 12 (2·0%) | 0·3565 |
| CR | 581 (98·8%) | 594 (98·0%) |  |
| MRD ≥ 0·01% | 152 (26·2%) | 166 (27·9%) | 0·5117 |
| MRD < 0.01% | 429 (73·8%) | 428 (72·1%) |  |
| Final risk |  |  |  |
| SR | 268 (45·9%) | 251 (41·5%) | 0·3150 |
| IR | 159 (27·2%) | 177 (29·3%) |  |
| HR | 157 (26·9%) | 177 (29·3%) |  |
| 3-Year Survival outcome (95% CI) | |  |  |
| Event-free survival (EFS) | 72·7% (68-77) | 72·2% (68-76) | 0·9555 |
| Overall survival (OS) | 85·0% (82-88) | 87·0% (84-89) | 0·3706 |
| Non-CR, not in complete remission; CR, in complete remission | | |  |
| MRD, minimal/measurable residual disease; SR, standard risk; IR, intermediate risk; HR, high risk; | | | |
| Intention-to-treat analysis; *p* value, 2-tailed Fisher exact test; p value for EFS and OS, Log-rank test | | | |

**Supplementary Table S5: Intention to Treat analysis of factors influencing risk of induction death in the R1 randomised cohort**

|  | Univariable Cox |  | Multivariable Cox |  |
| --- | --- | --- | --- | --- |
| Covariates | HR (95% CI) | p | HR (95% CI) | p |
| Sex (ref: Female) | 1.00 |  | 1.00 |  |
| Male | 0·78 (0·38-1·62) | 0·5010 | 0·68 (0·33-1·42) | 0·3048 |
| Age at diagnosis | 0·83 (0·68-1·01) | 0·0599 | 0·83 (0·69-1·00) | 0·0521 |
| Presenting WCC | 1·54 (1·17-2·02) | 0·0019 | 1·07 (0·74-1·56) | 0·7202 |
| Bulky disease | 2·11 (1·00-4·43) | 0·0488 | 0·76 (0·28-2·08) | 0·5972 |
| Anthracycline | 5·03 (2·24-11·30) | <0·0001 | 5·44 (1·56-18·91) | 0·0077 |
| Randomisation 1 (Ref: R1B) | 1.00 |  | 1.00 |  |
| R1A | 2·81 (1·25-6·32) | 0·0122 | 3·06 (1·36-6·91) | 0·0069 |
| Cox regression analysis by intention-to-treat; HR, hazard ratio; 95% CI, 95% confidence interval Ref, reference; WCC, white cell count; Age and WCC as continuous variables | | | | |

**Supplementary Table S6: Per-protocol analysis of factors influencing risk of induction death in the R1 randomised cohort**

|  | Univariable Cox | | Multivariable Cox | |
| --- | --- | --- | --- | --- |
| Covariates | HR (95% CI) | p | HR (95% CI) | p |
| Sex (ref: Female) | 1.00 |  | 1.00 |  |
| Male | 0·77 (0·37 - 1·60) | 0·4820 | 0·67 (0·32 - 1·38) | 0·2791 |
| Age at diagnosis | 0·83 (0·68 - 1·01) | 0·0586 | 0·83 (0·68 - 1·00) | 0·0496 |
| Presenting WCC | 1·54 (1·17 - 2·01) | 0·0020 | 1·07 (0·73 - 1·55) | 0·7361 |
| Bulky disease | 2·13 (1·01 - 4·48) | 0·0460 | 0·76 (0·28 - 2·06) | 0·5917 |
| Anthracycline | 5·06 (2·25 - 11·4) | <0·0001 | 5·55 (1·60 - 19·2) | 0·0069 |
| Randomisation 1 (Ref: R1B) | 1.00 |  | 1.00 |  |
| R1A | 2·81 (1·25 - 6·32) | 0·0122 | 3·10 (1·38 - 7·00) | 0·0064 |

Cox regression analysis by per-protocol

HR, hazard ratio; 95% CI, 95% confidence interval; Ref, reference; WCC, white cell counts

**Supplementary Table S7: Multiple Imputation analysis of factors influencing risk of induction death in R1 randomised cohort**

| Covariates | *OR (95% CI)* | *p* |  |
| --- | --- | --- | --- |
| Univariable logistic regression |  |  |  |
| Randomisation 1 (Ref: R1B) | 1.00 |  |  |
| R1A | 2·74 (1·24 - 6·05) | 0·0147 |  |
|  | *HR (95% CI)* | *p* |  |
| Univariable Cox-regression |  |  |  |
| Randomisation 1 (Ref: R1B) | 1.00 |  |  |
| R1A | 2·72 (1·25 - 5·90) | 0·0197 |  |
| Multivariable Cox-regression |  |  |  |
| Sex (ref: Female) | 1.00 |  |  |
| Male | 0·64 (0·30 - 1·35) | 0·2574 |  |
| Age at diagnosis | 0·82 (0·70 - 0·95) | 0·0122 |  |
| Presenting WCC | 1·01 (0·71 - 1·43) | 0·9779 |  |
| Bulky disease | 0·72 (0·30 - 1·73) | 0·4650 |  |
| Anthracycline | 5·07 (1·80 - 14·3) | 0·0051 |  |
| Randomisation 1 (Ref: R1B) | 1.00 |  |  |
| R1A | 2·94 (1·36 - 6·37) | 0·0135 |  |

Univariable logistic regression and pooled multivariable Cox-regression estimates derived from multiple imputations (5 iterations) in R1-randomised patients with missing induction death data (27 patients, 2·2% missingness). Multiple imputation by chained equations was employed to address missingness following Rubin’s rules and assuming missing-at-random data.

HR, hazard ratio; OR, odds Ratio; 95% CI, 95% confidence interval

Ref, reference; WCC, white cell counts;

**Supplementary Table S8: Frequency of severe non-fatal toxicity during induction in provisional risk groups (per protocol analyses)**

Proportion of patients with Grade 3 (requiring hospital-based management) and Grade 4 (life-threatening) toxicities in each randomised arm and in each provisional risk group (Standard and Intermediate Risk B cell-precursor ALL), based on the National Cancer Institute Common Terminology Criteria for Adverse Events (NCI-CTCAE version 4·03) ‘Others’ include patients with meningism/aseptic meningitis following intrathecal treatment, and patients with probable treatment-related illness but without definitive toxicity diagnosis

| Provisional risk groups | Standard risk | | | Intermediate risk | | |
| --- | --- | --- | --- | --- | --- | --- |
| **Randomised arms** | **R1A** | **R1B** |  | **R1A** | **R1B** |  |
| N (%) | 394 | 396 | *p* | 216 | 220 | *p* |
| **Overall** | **156 (39·6)** | **156 (39·4)** | 1·0000 | **111 (51·4)** | **119 (54·1)** | 0·6315 |
| Sepsis | 120 (30·5) | 126 (31·8) | 0·7012 | 90 (41·7) | 105 (47·7) | 0·2118 |
| Hypertension | 36 (9·1) | 24 (6·1) | 0·1087 | 23 (10·6) | 27 (12·3) | 0·6532 |
| Hyperglycaemia | 1 (0·3) | 2 (0·5) | 1.0000 | 2 (0·9) | 2 (0·9) | 1·0000 |
| Proximal myopathy | 6 (1·5) | 4 (1·0) | 0·5454 | 0 | 3 (1·4) | 0·2483 |
| Allergy | 2 (0·5) | 2 (0·5) | 1·0000 | 2 (0·9) | 0 | 0·2449 |
| Bleeding | 4 (1·0) | 4 (1·0) | 1·0000 | 5 (2·3) | 2 (0·9) | 0·2812 |
| Constipation | 6 (1·5) | 14 (3·5) | 0·1107 | 10 (4·6) | 8 (3·6) | 0·8094 |
| Diarrhoea | 10 (2·5) | 7 (1·8) | 0·4750 | 9 (4·2) | 8 (3·6) | 0·8094 |
| Encephalopathy | 1 (0·3) | 1 (0·3) | 1·0000 | 2 (0·9) | 2 (0·9) | 1·0000 |
| Infusion site extravasation | 4 (1·0) | 1 (0·3) | 0·2164 | 2 (0·9) | 1 (0·5) | 0·6207 |
| Mucositis | 5 (1·3) | 4 (1·0) | 0·7520 | 6 (2·8) | 11 (5·0) | 0·3230 |
| Neuropathy | 2 (0·5) | 1 (0·3) | 0·6236 | 1 (0·5) | 3 (1·4) | 0·6233 |
| Pancreatitis | 2 (0·5) | 1 (0·3) | 0·6236 | 2 (0·9) | 2 (0·9) | 1·0000 |
| Seizure | 3 (0·8) | 3 (0·8) | 1.0000 | 6 (2·8) | 3 (1·4) | 0·3348 |
| Serum albumin | 1 (0·3) | 0 | 0·4987 | 1 (0·5) | 0 | 0·4954 |
| Serum bilirubin | 1 (0·3) | 1 (0·3) | 1·0000 | 1 (0·5) | 0 | 0·4954 |
| Serum creatinine | 0 | 1 (0·3) | 1·0000 | 0 | 1 (0·5) | 1·0000 |
| SIADH | 2 (0·5) | 2 (0·5) | 1·0000 | 3 (1·4) | 2 (0·9) | 0·6832 |
| Thromboembolic event | 1 (0·3) | 2 (0·5) | 1·0000 | 4 (1·9) | 0 | 0·0594 |
| Vomiting | 4 (1·0) | 2 (0·5) | 0·4504 | 4 (1·9) | 6 (2·7) | 0·7511 |
| Others | 1 (0·3) | 1 (0·3) | 1·0000 | 1 (0·5) | 0 | 0·4954 |

Per-protocol analysis; p values, 2-tailed Fisher exact test

| Covariates | RR (95% CI) | p |
| --- | --- | --- |
| Sex (ref: Female) | 1.00 |  |
| Male | 0·93 (0·80 - 1·07) | 0·3008 |
| Age at diagnosis | 0·99 (0·96 - 1·03) | 0·6987 |
| Presenting WCC | 1·08 (1·01 - 1·16) | 0·0323 |
| Bulky disease | 1·11 (0·88 - 1·40) | 0·3816 |
| Anthracycline | 1·31 (1·01 - 1·68) | 0·0393 |
| Randomisation 1 (Ref: R1B) | 1.00 |  |
| R1A | 1·00 (0·87 - 1·15) | 0·9837 |
| Poisson multivariable regression by intention-to-treat | | |
| RR, rate ratio; 95% CI, 95% confidence interval | | |
| Age and white cell count as continuous variables; WCC, white cell counts | | |

**Supplementary Table S9: Intention-to-treat analysis of factors influencing risk of non-fatal severe toxicity during induction treatment in the R1 randomised cohort**

**Supplementary Table S10: Per-protocol analysis of factors influencing risk of non-fatal severe toxicity during induction treatment in the R1 randomised cohort**

| Covariates | RR (95% CI) | p |
| --- | --- | --- |
| Sex (ref: Female) | 1.00 |  |
| Male | 0·93 (0·80 - 1·07) | 0·3155 |
| Age at diagnosis | 0·99 (0·96 - 1·02) | 0·5474 |
| Presenting WCC | 1·08 (1·01 - 1·16) | **0·0340** |
| Bulky disease | 1·11 (0·88 - 1·40) | 0·3921 |
| Anthracycline | 1·33 (1·03 - 1·72) | **0·0265** |
| Randomisation 1 (Ref: R1B) | 1.00 |  |
| R1A | 1·01 (0·87 - 1·16) | 0·9188 |
| Poisson multivariable regression by per-protocol  RR: Rate ratio; 95% CI: 95% Confidence interval | | |
| Ref, reference; WCC, white cell counts | |  |

**Supplementary Table S11: Induction and post-induction events in the risk stratified groups within treatment arms**

|  | **R1A** |  |  |  | **R1B** |  |  |  |
| --- | --- | --- | --- | --- | --- | --- | --- | --- |
|  | Overall | SR | IR | HR | Overall | SR | IR | HR |
| **N (Induction)** | **623** | **400** | **223** | **0** | **623** | **400** | **223** | **0** |
| Induction death | 22 (3.5%) | 7 (1.8%) | 15 (6.7%) | 0 | 8 (1.3%) | 1 (0.3%) | 7 (3.1%) | 0 |
| Induction withdrawal | 17 (2.7%) | 8 (2.0%) | 9 (4.0%) | 0 | 10 (1.6%) | 7 (1.8%) | 3 (1.4%) | 0 |
| **N (Post-induction)** | **584** | **268** | **159** | **157** | **605** | **251** | **177** | **177** |
| Post-induction death | 21 (3.6%) | 9 (3.4%) | 8 (5.0%) | 4 (2.6%) | 28 (4.6%) | 12 (4.8%) | 10 (5.6%) | 6 (3.4%) |
| Relapse | 112 (19.2%) | 44 (16.4%) | 24 (15.1%) | 44 (28.0%) | 124 (20.5%) | 55 (21.9%) | 30 (17.0%) | 39 (22.0%) |
| Non-remission | 6 (1.0%) | 0 | 0 | 6 (3.8%) | 5 (0.8%) | 0 | 0 | 5 (2.8%) |
| Disease progression | 1 (0.2%) | 0 | 1 (0.6%) | 0 | 0 | 0 | 0 | 0 |
| R1A vs R1B: Post induction deaths p=0·3854; Relapses p=0·5116 (Fisher's exact test) | | | | | | | | |

**Supplementary Table S12: Per‑protocol induction and post‑induction events by treatment arm**

|  | **R1A** | **R1B** | **Absolute differences** | **Risk ratio** |  |
| --- | --- | --- | --- | --- | --- |
| **Induction** | 623 | 623 | (95% CI) | (95% CI) | p value |
| TRM* | 22/606 (3.6%) | 8/613 (1.3%) | 2.33 (0.58-4.24) | 2.78 (1.25-6.20) | **0.0094** |
| Non-CR at EOI$ | 6/585 (1.0%) | 12/605 (2.0%) | -0.96 (-2.51-0.50) | 0.52 (0.20-1.37) | 0.2355 |
| MRDhi at EOI^ | 151/579 (26.1%) | 165/593 (27.8%) | -1.75 (-6.81-3.34) | 0.94 (0.78-1.13) | 0.5109 |
| **Post-induction** | |  |  |  |  |
| TRM# | 21/554 (3.8%) | 28/591 (4.7%) | -0.95 (-3.35-1.46) | 0.80 (0.46 -1.39) | 0.4672 |
| Relapse# | 112/554 (20.2%) | 124/591 (21.1%) | -0.77 (-5.44-3.94) | 0.96 (0.77-1.21) | 0.7703 |
| Non-CR at EOC# | 6/554 (1.1%) | 5/591 (0.9%) | 0.24 (-1.03-1.59) | 1.28 (0.39- 4.17) | 0.7673 |
| * Per-protocol induction denominators: 606 (R1A) and 613 (R1B) a | | | | | |
| $ Response not assessed due to induction deaths in R1A, 21 and in R1B, 8 | | | | | |
| ^ MRDhi at EOI calculated for those in CR at EOI with MRD available: R1A 579; R1B 593 | | | | | |
| # Patients evaluable for post-induction events in R1A, 554 (30 withdrawn post-induction and | | | | | |
| 22 deaths in induction); R1B 591 (613-22, 14 withdrawn post-induction and 8 deaths in induction) | | | | | |
|  | | | | | |

**Supplementary Table S13: Overall cohort characteristics, categorised by final risk group**

| Final risk groups | | Standard risk | Intermediate risk | High risk | T-ALL / LL |
| --- | --- | --- | --- | --- | --- |
| N (%) | 545 | | 506 | 888 | 392 |
| Age (years) |  | |  |  |  |
| Median age with IQR | 4·2 (3·1; 6·1) | | 5·7 (3·4, 10·6) | 5.1 (3·1; 8·7) | 8·9 (5·9, 12·3) |
| Sex |  | |  |  |  |
| Male | 339 (62·2%) | | 348 (68·8%) | 586 (66·0%) | 314 (80·1%) |
| Female | 206 (37·8%) | | 158 (31·2%) | 302 (34·0%) | 78 (19·9%) |
| Male::Female | 1·7 | | 1·6 | 1·9 | 4·0 |
| Bulky Disease |  | |  |  |  |
| Yes | 0 | | 243 (48·0%) | 205 (23·1%) | 228 (58·2%) |
| No | 545 (100%) | | 263 (52·0%) | 683 (76·9%) | 164 (41·8%) |
| Presenting WCC (x10^9^ L) |  | |  |  |  |
| Median with IQR | 7·7 (4·3; 16·6) | | 28·6 (9·0; 79·8) | 20·6 (7·4; 60·1) | 80·7 (18·3; 217·8) |
| <50 | 545 (100%) | | 290 (57·3%) | 628 (70·7%) | 157 (40·1%) |
| ≥50 | 0 | | 216 (42·7%) | 260 (29·3%) | 235 (59·9%) |
| Cytogenetic groups |  | |  |  |  |
| Non-high risk | 545 (100%) | | 506 (100%) | 734 (82·7%) | - |
| High risk | 0 | | 0 | 154 (17·3%) | - |
| Day8 Prednisolone response |  | |  |  |  |
| Not evaluable/abrogated | 0 | | 0 | 54 (6·1%) | 68 (17·4%) |
| Good | 545 (100%) | | 506 (100%) | 569 (64·1%) | 241 (61·5%) |
| Poor | 0 | | 0 | 265 (29·8%) | 83 (21·1%) |
| CNS Disease |  | |  |  |  |
| Yes | 0 | | 0 | 33 (3·7%) | 13 (3·3%) |
| No | 545 (100%) | | 506 (100%) | 855 (96·3%) | 379 (96·7%) |
| EoI Response assessed |  | |  |  |  |
| Non-CR | 0 | | 0 | 52 (5·9%) | 33 (8·4%) |
| CR | 545 (100%) | | 506 (100%) | 836 (94·1%) | 359 (91·6%) |
| MRD ≥ 0·01% | 0 | | 0 | 477 (57·1%) | - |
| MRD < 0.01% | 545 (100%) | | 506 (100%) | 356 (42·6%) | - |
| Indeterminate | 0 | | 0 | 3 (0.3%) | - |

IQR, interquartile range; WCC, white cell counts; CNS, central nervous system;

EoI, end of induction; Non-CR, not in complete remission; CR, in complete remission;

MRD, measurable residual disease

**Supplementary Table S14: Factors influencing risk of Grade 3-4 toxicities across all intensive phases of treatment**

| Covariates | RR (95% CI) | *p* |
| --- | --- | --- |
| Sex (ref: Female) | 1 |  |
| Male | 0·91 (0·84 - 0·98) | 0·0179 |
| Age at diagnosis (years) | 0·98 (0·97 - 0·99) | <0·0001 |
| Presenting WCC (× 10^9^/L) | 1·07 (1·04 - 1·10) | <0·0001 |
| Bulky disease | 0·95 (0·87 - 1·04) | 0·2569 |
| CNS disease | 1·43 (1·11 - 1·83) | 0·0049 |
| Prednisolone response (ref: Good) | 1 |  |
| Abrogated response | 1·19 (1·00 - 1·41) | 0·0424 |
| Poor response | 0·88 (0·78 - 0·99) | 0·0322 |
| Risk groups (ref: HR) | 1 |  |
| IR | 1·02 (0·92 - 1·14) | 0·6877 |
| SR | 0·63 (0·57 - 0·71) | <0·0001 |
| T | 0·90 (0·79 - 1·02) | 0·0983 |
| Treatment phases (Ref: Induction) | 1 |  |
| Consolidation | 0·67 (0·62 - 0·73) | <0·0001 |
| Interim Maintenance | 0·44 (0·40 - 0·48) | <0·0001 |
| Delayed Intensification | 0·56 (0·52 - 0·61) | <0·0001 |
| RR, rate ratio; 95% CI, 95% confidence interval | | |
| CNS, central nervous system; WCC, white cell counts; Ref, reference | | |
| SR, IR, HR: standard-, intermediate- and high-Risk B cell-precursor ALL; | | |
| T, T cell lymphoblastic leukaemia/lymphoma  Factors influencing grade 3-4 toxicity across the four intensive treatment phases were evaluated using Poisson regression modelling of repeated counts of distinct toxicity types recorded for each patient | | |

**Supplementary Table S15:**  **Multivariable regression analysis of factors influencing risk of Grades 3-4 toxicity in each of the intensive treatment phases (Induction to Delayed intensification) in ICiCLe-ALL-14 trial patients**

| **Induction phase** | RR (95% CI) | p |
| --- | --- | --- |
| Sex (ref: Female) | 1.00 |  |
| Male | 0·91 (0·82 - 1·00) | 0·0545 |
| Age at diagnosis | 1·00 (0·99 - 1·01) | 0·8359 |
| Lineage (ref: B) | 1.00 |  |
| T | 0·77 (0·66 - 0·91) | **0·0020** |
| Presenting WCC | 1·08 (1·04 - 1·12) | **0·0001** |
| Bulky disease | 1·10 (0·98 - 1·23) | 0·1174 |
| CNS disease | 1·40 (1·07 - 1·83) | **0·0137** |
| Prednisolone response (ref: Good) | 1.00 |  |
| Abrogated | 1·15 (0·94 - 1·40) | 0·1862 |
| Poor | 0·81 (0·69 - 0·96) | **0·0148** |
| Anthracycline (ref: Four) | 1.00 |  |
| Two | 0·84 (0·72 - 0·99) | **0·0340** |
| None | 0·64 (0·53 - 0·76) | **<0·0001** |
| **Consolidation phase** |  |  |
| Sex (ref: Female) | 1.00 |  |
| Male | 1·01 (0·89 - 1·15) | 0·8724 |
| Age at diagnosis | 0·95 (0·94 - 0·97) | **<0·0001** |
| Lineage (ref: B) | 1.00 |  |
| T | 1·06 (0·90 - 1·24) | 0·4756 |
| Cyclo-Cytarabine block | 5·33 (4·03 - 7·06) | **<0·0001** |
| VCR-AsNase block | 1·19 (1·03 - 1·38) | **0·0179** |
| **Interim maintenance phase** |  |  |
| Sex (ref: Female) | 1.00 |  |
| Male | 0·74 (0·64 - 0·87) | **0·0002** |
| Age at diagnosis | 0·98 (0·96 - 1·00) | 0·0599 |
| Lineage (ref: B) | 1.00 |  |
| T | 1·12 (0·88 - 1·44) | 0·3650 |
| Methotrexate (ref: Oral) | 1.00 |  |
| Escalating IV | 2·08 (1·68 - 2·57) | **<0·0001** |
| High dose | 1·09 (0·88 - 1·35) | 0·4473 |
| **Delayed intensification phase** |  |  |
| Sex (ref: Female) | 1.00 |  |
| Male | 0·60 (0·52 - 0·71) | 0·3470 |
| Age at diagnosis | 0·95 (0·93 - 0·97) | **<0·0001** |
| Lineage (ref: B) | 1.00 |  |
| T | 0·95 (0·77 - 1·17) | 0·6080 |

Poisson regression; RR, risk ratio; 95% CI, 95% confidence interval

Ref, reference; WCC, white cell counts

CNS, central nervous system; Cyclo, cyclophosphamide

VCR, vincristine; AsNase, asparaginase; IV, intravenous

**Supplementary Table S16: Comparative Outcomes of Clinical Trials in ALL with randomisations in induction.**

| **Study** | **Randomisation** |  | **Induction** |  | **Toxicity** |  | **EFS** |  |
| --- | --- | --- | --- | --- | --- | --- | --- | --- |
|  | **Dexamethasone** | N | TRM | p | Grade3-4 | p |  | p |
| UKALL 2011* | 6mg/m^2^ × 28 days | 948 | 6 (0.6%) | 0·45 | 242 (26%) | 0·41 | 84% | 0·069 |
|  | 10mg/m^2 ×^ 14 days | 949 | 10 (1.1%) |  | 227 (34%) |  | 82% |  |
|  |  |  |  |  |  |  |  |  |
|  | **Daunorubicin** |  |  |  |  |  |  |  |
| AEIOP-BFM ALL 2009** | 30mg/m^2 ×^ 4 doses | 1023 | 8 (0.8%) | NA | 97 (9%) | NA | 93% | 0·66 |
|  | 30mg/m^2 ×^ 2 doses | 1016 | 6 (0.6%) |  | 76 (7%) |  | 92% |  |
|  |  |  |  |  |  |  |  |  |
|  | **Prednisolone** |  |  |  |  |  |  |  |
| ICiCLe-ALL-14# | 60mg/m^2^ × 35 days with taper | 623 | 22 (3.5%) | 0·0149 | 267 (43%) | 0·6892 | 73% | 0·9555 |
|  | 60mg/m^2^ × 21 days | 623 | 8 (1.3%) |  | 275 (44%) |  | 72% |  |
| * All patients were randomised | | | | | | | | |
| ** Only patients with *ETV6::RUNX1* and those with blast count <0.1% at day 15 on the bone marrow aspirate and no other high-risk features were | | | | | | | | |
| randomised; toxicities were reported as protocol-specific adverse reactions of special interests | | | | | | |  |  |
| # Only patients aged <10 years with no high-risk features were randomised | | |  |  |  |  |  |  |
|  |  |  |  |  |  |  |  |  |
| UKALL 2011: JCO 20205;43:1810-1823; AEIOP-BFM: JCO 2025 DOI: 10.1200/JCO-25-01357 | | | | | | | | |
| ICiCLe: The Lancet Regional Health - Southeast Asia 2025;37:100593 | | | | | | | | |
|  | |  |  |  |  |  |  |  |


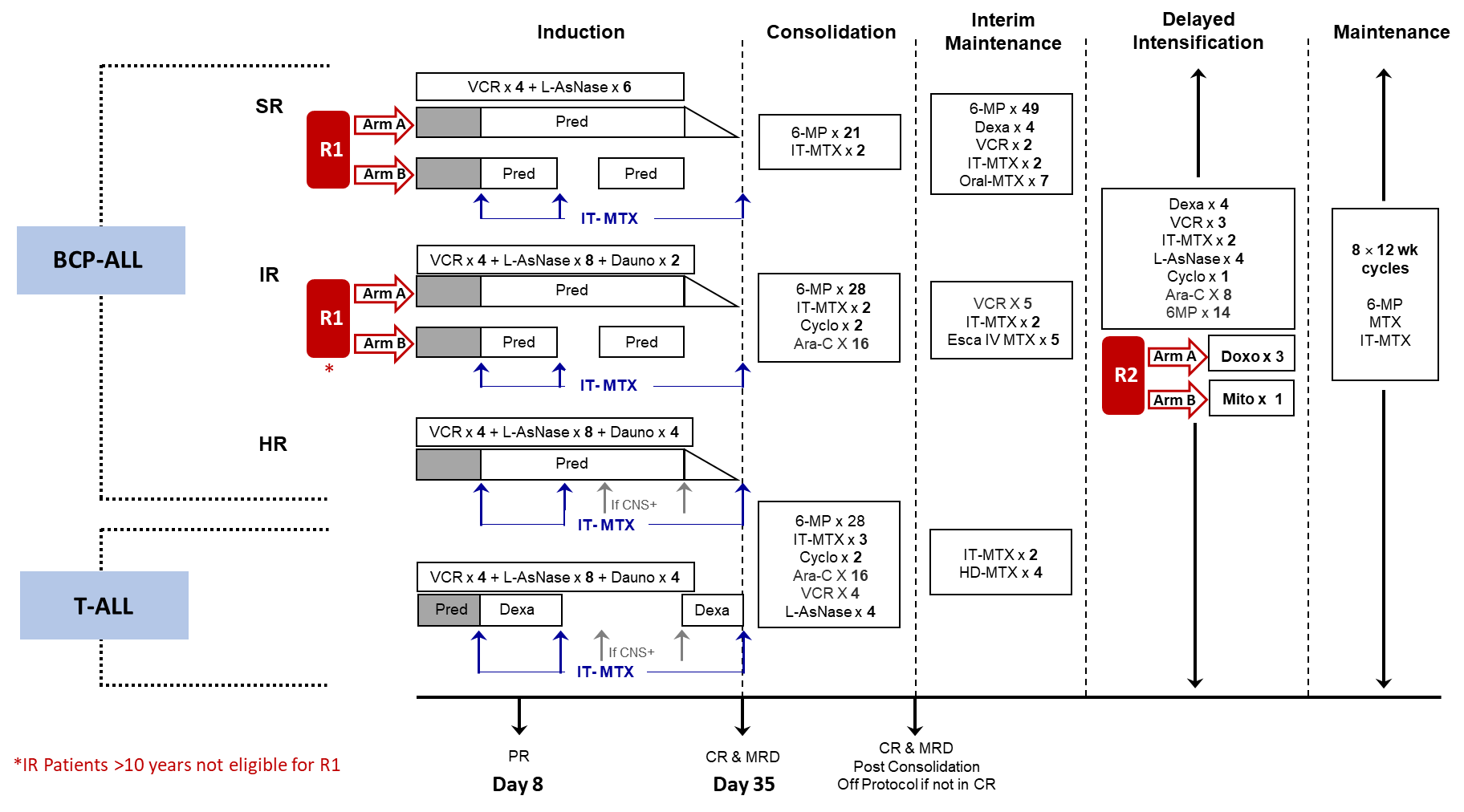


**Supplementary Figure S1: Schematic representation of risk-stratified treatment and randomised interventions in ICiCLe-ALL-14:**

BCP-ALL, B-cell precursor acute lymphoblastic leukaemia; T-ALL, T cell lymphoblastic leukaemia/ lymphoma; SR, standard-risk; IR, Intermediate-risk; HR, high-risk;R1, Randomisation 1; Arm A, standard-duration schedule of prednisolone (4 weeks followed by taper); Arm B, pulsed prednisolone schedule (days 1–14, days 22–28); VCR, vincristine; L-AsNase, native *E.coli* asparaginase; Dauno, daunorubicin; IT-MTX, intrathecal methotrexate; CNS+, with central nervous system leukaemia; 6-MP, 6-mercaptopurine; Ara-C, cytarabine; Cyclo, cyclophosphamide; Oral-MTX, oral methotrexate; Esca IV MTX, escalating intravenous methotrexate; Dexa, dexamethasone; HD-MTX, high dose intravenous methotrexate; MRD, minimal residual disease; MTX, methotrexate; R2, Randomisation 2; Arm-A, standard 3 doses of doxorubicin; Arm-B, 1 dose of mitoxantrone; Doxo, Doxorubicin; Mito, Mitoxantrone

**Supplementary Figure S2: Cumulative incidence of treatment-related death during induction within provisional risk groups of the R1 randomised cohort**

Cumulative incidence representation, with treatment abandonment/discontinuation considered as competing event

**
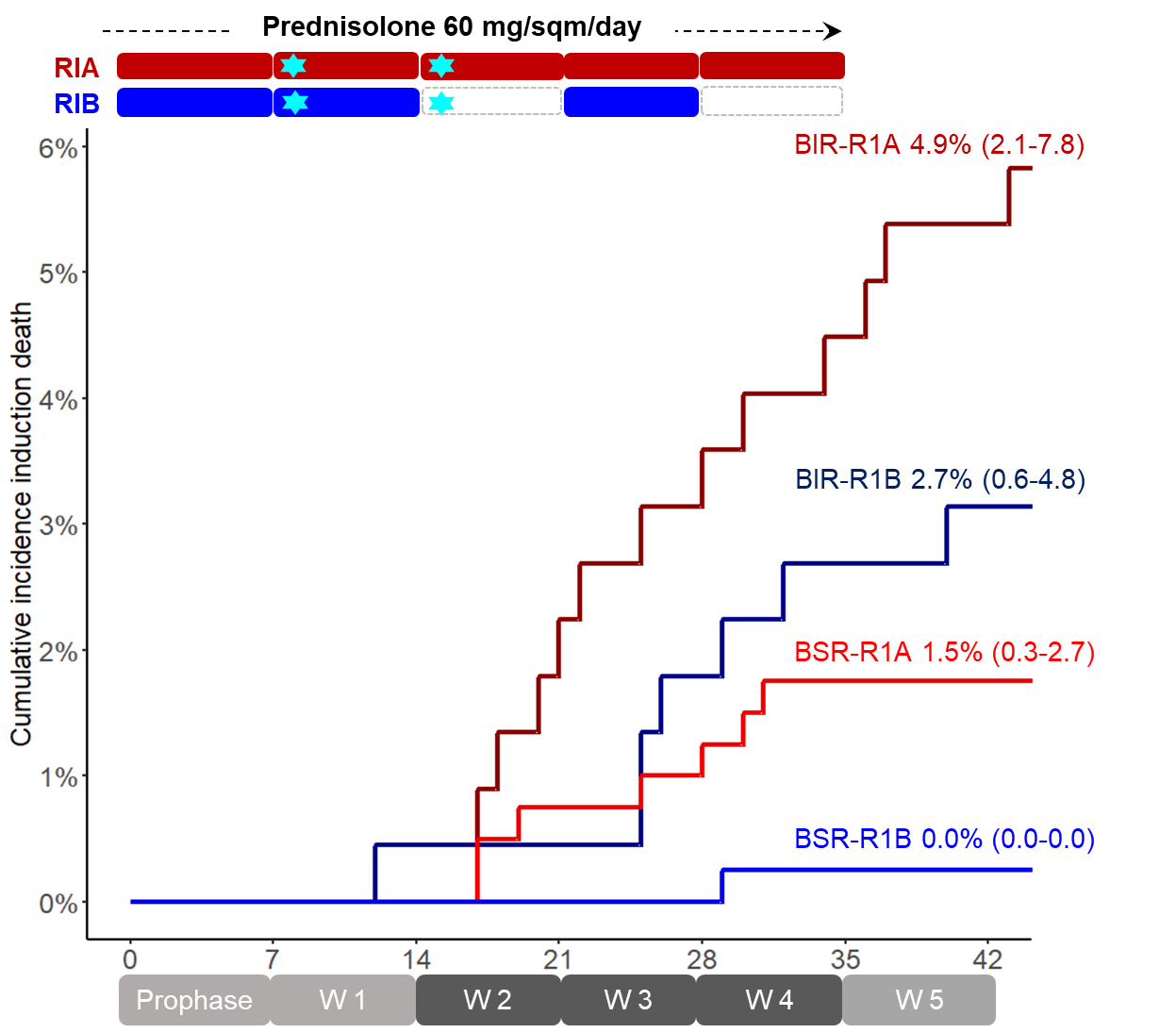
**

**Supplementary Figure S3: Temporal distribution of treatment-related deaths in ICiCLe-ALL risk groups**

Raincloud plot indicating timing of treatment-related death within ALL risk groups (A) B‑cell precursor standard-risk (BSR), (B) B‑cell precursor intermediate-risk (BIR), (C) B‑cell precursor high-risk (BHR), and (D) T‑lineage (T) acute lymphoblastic leukaemia/lymphoblastic lymphoma.

Each red dot represents an individual treatment‑related death plotted by time from diagnosis (months). Box plots show median and interquartile ranges with whiskers indicating the range, and the coloured density plot to the right of each box plot depicts the distribution of time to treatment‑related mortality within the corresponding risk group.


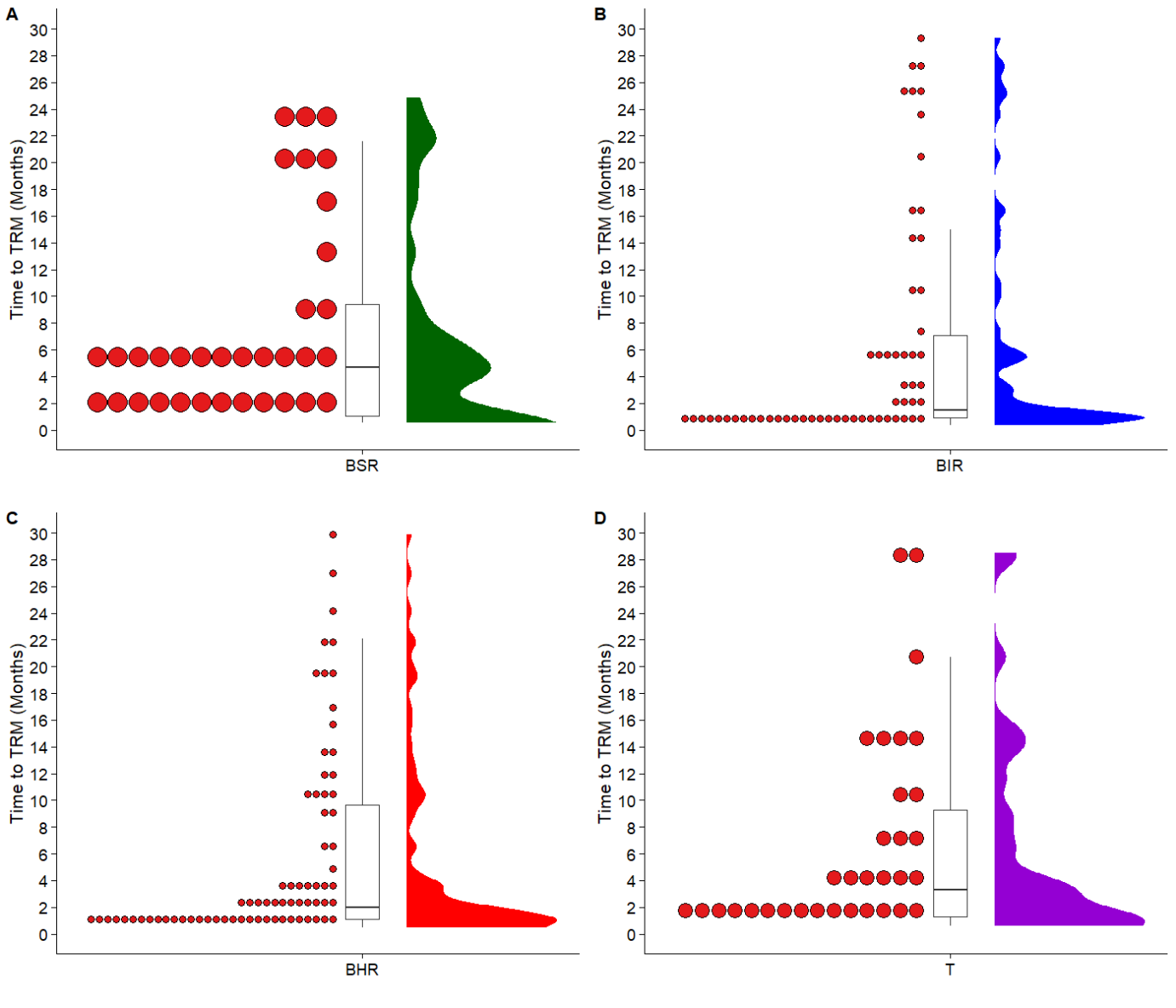

Supplement: Supplementary Figs. S1–S3 and Tables S1–S16 [file mmc1.docx]
