# Supplementary material for: Toxicity during induction of pulsed versus continuous prednisolone in children with acute lymphoblastic leukaemia: a multi-centre, open label, randomised, phase 3 trial from India (2016–2022)
Source: Lancet Reg Health Southeast Asia. 2026 Jun 7;50:100788. doi: 10.1016/j.lansea.2026.100788 (PMC13366301; doi:10.1016/j.lansea.2026.100788)
Supplement: ICiCle 14 protocol_Trials [file mmc2.pdf]

Das et al. Trials (2022) 23:102
https://doi.org/10.1186/s13063-022-06033-1

Trials

STUDY PROTOCOL Open Access

Protocol for ICiCLe-ALL-14 (InPOG-ALL-15-01): a prospective, risk stratified, randomised, multicentre, open label, controlled therapeutic trial for newly diagnosed childhood acute lymphoblastic leukaemia in India

Nandana Das[1], Shripad Banavali[2,3], Sameer Bakhshi[4], Amita Trehan[5], Venkatraman Radhakrishnan[6], Rachna Seth[7], Brijesh Arora[2,3], Gaurav Narula[2,3], Subir Sinha[8], Prakriti Roy[1], Manash Pratim Gogoi[1], Sayan Chatterjee[1], Bindhu Abraham[9], Parag Das[1], Vaskar Saha[1,10,11] and Shekhar Krishnan[1,10,11*]

Abstract

Background: In the west, survival following treatment of childhood acute lymphoblastic leukaemia (ALL) approaches 90%. Outcomes in India do not exceed 70%. To address this disparity, the Indian Collaborative Childhood Leukaemia group (ICiCLe) developed in 2013 a contemporary treatment protocol for uniform risk-stratified management of first presentation ALL based on cytogenetics and minimal residual disease levels (MRD). A multicentre randomised clinical trial opened in 2016 (ICiCLe-ALL-14) and examines the benefit of randomised interventions to decrease toxicity and improve outcomes.

* Correspondence: shekhar.krishnan@ttcrc.tmckolkata.org
[1]Clinical Research Unit, Tata Translational Cancer Research Centre, Tata Medical Centre, 14 MAR (E-W), New Town, Kolkata, West Bengal 700160, India
[10]Department of Pediatric Oncology, Tata Medical Center, Kolkata, West Bengal 700160, India
Full list of author information is available at the end of the article

© The Author(s). 2022 **Open Access** This article is licensed under a Creative Commons Attribution 4.0 International License, which permits use, sharing, adaptation, distribution and reproduction in any medium or format, as long as you give appropriate credit to the original author(s) and the source, provide a link to the Creative Commons licence, and indicate if changes were made. The images or other third party material in this article are included in the article's Creative Commons licence, unless indicated otherwise in a credit line to the material. If material is not included in the article's Creative Commons licence and your intended use is not permitted by statutory regulation or exceeds the permitted use, you will need to obtain permission directly from the copyright holder. To view a copy of this licence, visit http://creativecommons.org/licenses/by/4.0/. The Creative Commons Public Domain Dedication waiver (http://creativecommons.org/publicdomain/zero/1.0/) applies to the data made available in this article, unless otherwise stated in a credit line to the data.

Methods: Patients 1–18 years with newly diagnosed ALL are categorised into four risk groups based on presentation features, tumour genetics and treatment response. Standard risk includes young (< 10 years) B cell precursor ALL (BCP-ALL) patients with low presentation leucocyte count (< 50 × 10^9/L) and no high-risk features. Intermediate risk includes BCP-ALL patients with no high-risk features but are older and have high presentation leucocyte counts and/or bulky disease. High risk includes BCP-ALL patients with any high-risk feature, including high-risk genetics, central nervous system leukaemia, poor prednisolone response at treatment day 8 and high MRD (≥ 0·01%) at the end of induction. Patients with T-lineage ALL constitute the fourth risk group. All patients receive four intensive treatment blocks (induction, consolidation, interim maintenance, delayed intensification) followed by 96 weeks of maintenance. Treatment intensity varies by risk group. Clinical data management is based on a web-based remote data capture system. The first randomisation examines the toxicity impact of a shorter induction schedule of prednisolone (3 vs 5 weeks) in young non-high-risk BCP-ALL. The second randomisation examines the survival benefit of substituting doxorubicin with mitoxantrone in delayed intensification for all patients. Primary outcome measures include event-free survival (overall, by risk groups), sepsis rates in induction (first randomisation) and event-free survival rates following second randomisation.

Discussion: ICiCLe-ALL-14 is the first multicentre randomised childhood cancer clinical trial in India. The pre-trial phase allowed standardisation of risk-stratification diagnostics and established the feasibility of collaborative practice, uniform treatment, patient enrolment and data capture. Pre-trial observations confirm the impact of risk-stratified therapy in reducing treatment-related deaths and costs. Uniform practice across centres allows patients to access care locally, potentially decreasing financial hardship and dislocation.

Trial registration: Clinical Trials Registry-India (CTRI) CTRI/2015/12/006434. Registered on 11 December 2015

Keywords: Acute lymphoblastic leukaemia, Childhood, Randomised trial, Risk stratification, Open label

Administrative information

Note: the numbers in curly brackets in this protocol refer to SPIRIT checklist item numbers. The order of the items has been modified to group similar items (see http://www.equator-network.org/reporting-guidelines/spirit-2013-statement-defining-standard-protocol-items-for-clinical-trials/

Title {1a}	Protocol for ICiCLe-ALL-14 (InPOG-ALL-15-01): A prospective, risk stratified, randomised, multicentre, open label, controlled therapeutic trial for newly diagnosed childhood acute lymphoblastic leukaemia in India.
Trial registration {2a and 2b}	Clinical Trials Registry - India (CTRI), ID: CTRI/2015/12/006434. Registered on 11 December 2015. The WHO Trial Registration Data Set is documented in Supplementary data. (ref comment i)
Protocol version {3}	Protocol Version 5.1. Dated 22 January 2020.
Funding {4}	Trial funding from Indian Council of Medical Research for initial 2 years (Reference 79/159/2015/NCD-III; 21 March 2017) and National Cancer Grid for 4 years (Reference 2016/001, 10 Aug 2016). Trial support from grant from DBT- Wellcome India Alliance (Margdarshi IA/M/12/1/500261).
Author details {5a}	[1] Clinical Research Unit, Tata Translational Cancer Research Centre, Tata Medical Centre, Kolkata, West Bengal, 700160, India<br>[2] Department of Pediatric Oncology, Tata Memorial Centre, Tata Memorial Hospital, Mumbai, Maharashtra 400012, India<br>[3] Homi Bhabha National Institute, Mumbai, Maharashtra ,40094, India.<br>[4] Department of Medical Oncology, All India Institute of Medical Sciences, New Delhi, 110029, India<br>[5] Pediatric Hematology-Oncology Unit, Department of Pediatrics, Advanced Pediatrics Center, Postgraduate Institute of Medical Education and Research, Chandigarh, 160012, India<br>[6] Department of Medical Oncology, Cancer Institute (WIA), Adyar, Chennai, Tamil Nadu 600020, India.<br>[7] Department of Pediatrics, All India Institute of Medical Sciences, New Delhi, 110029, India.<br>[8] Department of Statistics, Tata Medical Center, Kolkata, West Bengal, 700160, India.<br>[9] Tata Consultancy Services, Kolkata, West Bengal, 700135, India.<br>[10] Department of Pediatric Oncology, Tata Medical Center, Kolkata, West Bengal, 700160, India.<br>[11] Division of Cancer Sciences, School of Medical Sciences, Faculty of Biology, Medicine and Health, University of Manchester, Manchester, M20 4BX, UK.
Name and contact information for the trial sponsor {5b}	Tata Medical Center<br>14, MAR (E-W), DH Block (Newtown), Action Area I, Newtown, Kolkata, West Bengal 700160.
Role of sponsor {5c}	The sponsor and funding organisations have no role in study design; collection, management, analysis, interpretation of data; writing of the report; and the decision to submit for publication.

Introduction

Background and rationale {6a}

Survival rates for childhood acute lymphoblastic leukaemia (ALL) approach 90% in high-income countries [1]. This is a reflection of the consolidated efforts of national and multi-national collaborative groups [2] to systematically optimise the treatment schedule and treatment elements of combination chemotherapy protocols. Common to these protocols, the intensity of treatment is stratified at diagnosis based on clinical features and genetics of the disease. Following completion of the initial induction treatment phase, decisions regarding the requirement for further treatment intensification is based on the levels of minimal residual disease (MRD) in the bone marrow, measured using techniques able to detect 1 leukaemia cell in a background of 10,000 cells.

Outcomes in childhood ALL lag behind In India, with survival rates of around ~ 65% [3–5]. The first cooperative protocol in India, MCP841 [3], was launched in 1984. Subsequently, centres adopted other western protocols, often modifying them for local use [5]. None of these approaches was risk stratified. While outcomes have improved in the last decade, treatment-related mortality has ranged from 11 to 25% with relapse rates at 15–41% [3–5]. Treatment-related deaths were primarily due to sepsis, with the highest incidence noted during the induction treatment phase [3, 5, 6]. A number of factors contribute to treatment-related deaths and relapses. In India, families often travel long distances to a treatment centre where they perceive their child will receive the best care [7]. This potentially leads to delay in diagnosis and treatment, with children often presenting in poor clinical condition. In the absence of risk stratification, centres have primarily used a four-drug induction schedule containing anthracycline and/or dexamethasone. Anthracycline use increases sepsis-related mortality [8]. Corticosteroid is the mainstay of induction therapy. While randomised comparisons suggest superior event-free survival with dexamethasone compared to prednisolone [9, 10], dexamethasone treatment is associated with increased induction mortality [11]. Poor tolerance of intensive chemotherapy, leading to therapy modifications [5] and gaps in therapy [12] could contribute to the high recurrence rates. Once the initial phase of treatment is completed, families often choose to relocate to centres closer to home. The local centre that provides continuing care may not always be familiar with the therapeutic protocol used by the referring centre, leading to further treatment delay or suboptimal therapy.

To address these issues, five major paediatric oncology centres came together in 2012 to form the Indian Collaborative Childhood Leukaemia (ICiCLe) study group. Geographically, the centres are located in the main metropolises of India, where families often travel to for treatment. The group developed a risk-stratified treatment protocol for children (1–18 years) with newly diagnosed ALL. Patients are categorised into four risk groups: B cell precursor ALL (BCP-ALL) patients with standard (SR), intermediate (IR) and high (HR) risk disease and patients with T-ALL. Age and the white blood cell (WBC) count at presentation collectively determine the National Cancer Institute (NCI) risk groups [13]. All patients are administered combination chemotherapy, including an intensive phase for 6-7 months involving sequential treatment with multi-drug chemotherapy blocks (induction, consolidation, interim maintenance, delayed intensification) followed by a 24-month maintenance phase that involves outpatient chemotherapy with the oral antimetabolites 6-mercaptopurine and methotrexate. The treatment intensity of induction, consolidation and interim maintenance phases varies by risk groups and is highest in HR and T-ALL patients. Delayed intensification and the maintenance treatment phases are similar in all risk groups. Central nervous system (CNS) directed treatment is administered using a combination of systemic and intrathecal chemotherapy with cranial irradiation restricted to a small proportion of patients (~ 2%) with involvement of the CNS at diagnosis.

The ICiCLe initiative is based on the hypothesis that uniform risk-adapted therapy of paediatric ALL, based on a contemporary treatment protocol and use of standardised investigations of disease genetics and minimal residual disease, will identify patients at lower risk of relapse who require lower-intensity therapy to obtain cure, while intensifying therapy appropriately in patients predicted to be at higher risk of treatment failure. This approach seeks to decrease treatment-related toxicity and deaths, treatment costs and improve outcomes. Uniform standardised treatment at treatment centres located in all four regional zones of the country will enable patients to obtain contemporary treatment more locally, obviating the need for travel to distant centres and alleviating the financial hardship of families.

A pre-trial phase was initiated in 2013, providing the opportunity for centres to establish collaborative practice, evaluate the feasibility of uniform protocol-based treatment, enrol and monitor patients, collect and record data, standardise the specialised tests required for risk stratification, develop a remote customised web-based electronic data capture system and obtain funding to run a multi-centre clinical trial. The ICiCLe-ALL-14 randomised trial opened three years later (2016) and is currently underway. The trial includes randomised interventions to decrease toxicity (shorter course of induction prednisolone) and improve survival (mitoxantrone in place of doxorubicin in delayed intensification).

Objectives {7}

Primary objectives

1. Decrease treatment-related toxicity and mortality and improve survival outcomes using a risk-stratified approach to ALL therapy based on disease genetics and levels of minimal residual disease (MRD)
2. Decrease toxicity and mortality in induction by shortening the duration of prednisolone therapy in patients with non-high risk ALL
3. Improve event-free survival (EFS) in risk groups by replacing doxorubicin with mitoxantrone in delayed intensification

Secondary Objectives

1. Standardisation of risk stratified therapy across participating centres, establishing a standard of care for children with ALL in India
2. Improve overall survival (OS) of children with ALL in India

Trial design {8}

ICiCLe-ALL-14 is an investigator-initiated, institution-led, multicentre, open-label, parallel-group randomised, phase III/IV study in children aged 1–18 years with newly diagnosed ALL. Following registration, eligible children are enrolled in the clinical trial after informed consent. Patients are categorised into four risk groups. The initial risk classification is based on lymphoblast lineage, presentation clinical features (age, leucocyte count, disease bulk, CNS disease status), leukaemia cytogenetics and prednisolone response at treatment day 8. The final risk stratification is determined at the end of the induction treatment phase and is based on treatment response, including remission status and the level of bone marrow MRD. All risk groups receive sequential blocks of chemotherapy that includes 6–7 months of intensive therapy (induction, consolidation, interim maintenance and delayed intensification) and 24 months of maintenance therapy. A web-based remote data entry system (RDES) supports data management, randomisations and study notifications. Parallel-arm 1:1 permuted block randomisation is performed centrally through the RDES and is stratified by trial centre. The first randomisation in induction examines the toxicity impact of a shorter pulsed course of the corticosteroid prednisolone versus the standard 4-week continuous prednisolone schedule in younger patients (< 10 years old) with non-high-risk B cell precursor ALL. The second randomisation examines the survival impact of substituting three doses of the anthracycline doxorubicin with one dose of mitoxantrone during delayed intensification in all risk groups. Trial events include treatment-related deaths, relapses and trial withdrawals (owing to excess toxicity, poor treatment response or treatment abandonment). In the absence of events, follow-up is continued for minimum 5 years from diagnosis.

Methods: participants, interventions and outcomes

Study setting {9}

The study is conducted at 6 participating sites in 5 hospitals. These centres are major paediatric oncology centres in India, three of which have participated in the previous MCP-841 study [3]. Details of participating sites are listed in Supplementary Table 1. At all study sites, children 1–18 years diagnosed consecutively with ALL are registered, screened for enrolment eligibility and their families approached for consent to participate in the study. The study flow is shown in Fig. 1. The protocol is the standard of care for all children with ALL presenting to these centres. Patients who do not consent to the study or randomisation, still receive the standard of care protocol.

Eligibility criteria {10}

Inclusion criteria

Inclusion criteria are evaluated prior to study enrolment, with decision support provided by the RDES. The inclusion criteria are as follows:

1. Diagnosis of ALL confirmed by microscopy and flow cytometry studies
2. Age ≥ 1 year and ≤ 18 years at the time of enrolment
3. Previously untreated patients, with the following exceptions
4. Prior treatment with prednisolone as sole agent, provided (i) has completed less than 7 days of prednisolone treatment or (ii) if has completed more than 7 days of prednisolone treatment, information on prednisolone response is available
5. Prior treatment with no more than one dose of vincristine or one dose of intrathecal methotrexate (IT-MTX)

Exclusion criteria

The exclusion criteria are as follows:

1. Previously treated patients who do not meet the criteria as stated above
2. Patients with mature B cell leukaemia or *c-myc* rearranged B cell precursor ALL
3. Patients with the Down syndrome
4. Mixed phenotype acute leukaemia

Who will take informed consent? {26a}

Informed consent is obtained from the parents of patients or from authorised surrogates. Assent is obtained

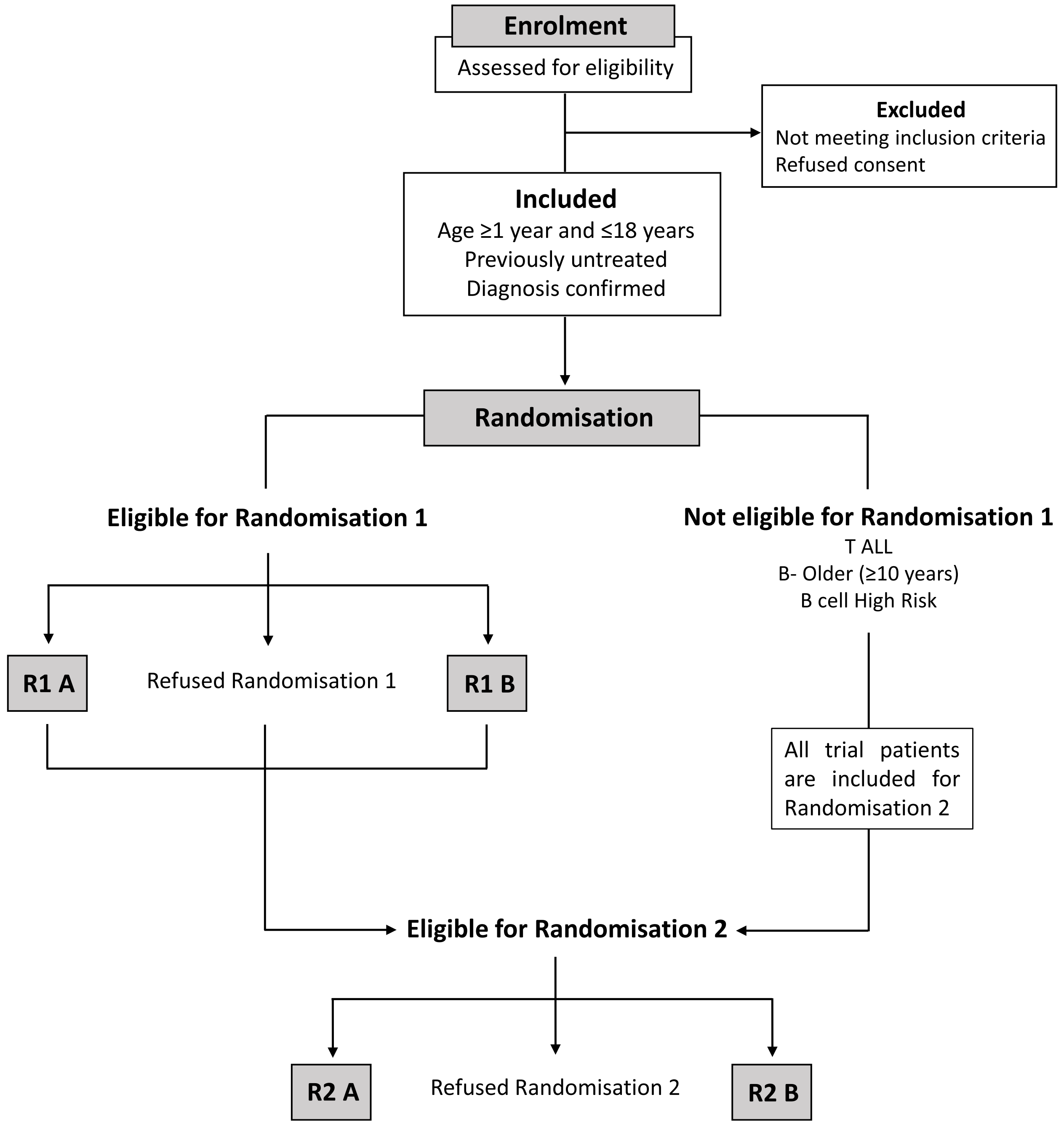

Fig. 1 Study flow in ICiCLe-ALL-14/ Following determination of enrolment eligibility and after obtaining consent to participate, consecutive patients 1–18 years old with newly diagnosed acute lymphoblastic leukaemia (ALL) are recruited to the trial. The first randomisation in the induction phase (R1) is restricted to younger B cell precursor ALL patients (age < 10 years) with standard and intermediate risk disease (as determined by risk stratification at treatment day 8). R1 randomisation compares toxicity of continuous (R1A) versus a pulsed prednisolone schedule (R1B) in the induction treatment phase. A second randomisation (R2) later in treatment (delayed intensification) is open to all risk groups, including patients who have completed R1 randomisation. R2 randomisation compares survival outcome with 3 doses of doxorubicin (R2A) versus 1 dose of mitoxantrone (R2B) in the delayed intensification phase of treatment

in children ≥ 8 years old. Following diagnosis of ALL, patients are registered for study enrolment and treatment is initiated after consent has been obtained for clinical management. All patients receive oral prednisolone alone as treatment for the first 7 days (prednisolone prophase). During this time, patients and families are provided written and verbal information about the clinical trial. Following completion of the 7-day prednisolone prophase, patients

undergo initial risk stratification. Consent to participate in the clinical trial is obtained at day 8, or at any time from registration to initial risk stratification. In young patients (Age at diagnosis < 10 years) with initial risk stratification as standard or intermediate risk ALL, consent is also obtained for the first randomisation (R1). The second randomisation (R2) in the delayed intensification phase of treatment is open to all enrolled patients, and informed consent for participation in this randomisation is obtained prior to the start of the delayed intensification treatment phase. Patients / authorised guardians may opt to participate in the study alone, or in either or both randomisations (wherever applicable). Signed consent and assent (where applicable) are obtained by designed clinical staff assigned to this responsibility and designated as such in the trial delegation log. Exemplar consent form and patient information sheets are provided in supplementary data.

Additional consent provisions for collection and use of participant data and biological specimens {26b}

All patients who consent to participate in the study are also approached for consent for the collection and use of anonymised data relevant to the study objectives. Collection of biological material is not mandated in the study and is specific to each centre. Centres that collect biological samples as part of independent research studies obtain consent separately for the collection and banking of pseudonymised clinical samples as part of peer-reviewed ethics-approved research projects.

Interventions

Explanation for the choice of comparators {6b}

There are two randomisations in ICiCLe-ALL-14. The first is in induction where standard risk patients and intermediate risk patients younger than 10 years are randomised to receive prednisolone (60 mg/m^2/day) either as a pulsed 3-week schedule (days 1–14 and days 22–28) or a continuous 4-week schedule with taper. The second randomisation is in delayed intensification, where all patients are eligible to be randomised to receive either one dose of intravenous mitoxantrone (10 mg/m^2, day 1) or 3 doses of intravenous doxorubicin (25 mg/m2/dose; days 1, 8, 15) (Fig. 2).

For over 5 decades, a continuous 4- to 5-week schedule of corticosteroid has been the mainstay of ALL induction therapy [14, 15]. Both dexamethasone and prednisolone have been used by different study groups in induction. A clear benefit was seen with dexamethasone for T-ALL but not for BCP-ALL [11, 16] and increased toxicities were reported with induction dexamethasone [9–11, 17]. The Berlin-Frankfurt-Münster (BFM) group administers prednisolone at 60 mg/m^2 daily for 5 weeks, including a 1-week prophase (cumulative dose, 2100 mg/m^2). The North American Children's Oncology Group (COG) administers prednisolone at the same dose for 4 weeks, equating to a total 1680 mg/m^2. Complications from prolonged corticosteroid treatment include infection, hypertension, myopathy, hyperglycaemia, weight gain, adrenal axis suppression and osteonecrosis. Older children (age ≥ 10 years) appear to be more susceptible to steroid-associated toxicities [11, 18–20]. Based on these observations and to balance steroid-related efficacy and toxicity, patients with BCP-ALL treated on the ICiCLe-ALL-14 protocol are administered prednisolone (60 mg/m^2/day) while patients with T-ALL are administered dexamethasone (at an equivalent dose of 10 mg/m^2/day). The standard continuous schedule of prednisolone is administered in high-risk BCP-ALL patients (60 mg/m^2/day, days 1–28, with taper over 5 days). To decrease steroid-associated toxicity, a pulsed corticosteroid schedule is administered in older children (age ≥ 10 years) with intermediate risk BCP-ALL (prednisolone 60 mg/m^2/day; days 1–14 and days 22–28) and in children with T-ALL (prednisolone prophase days 1–7 at 60 mg/m^2/day, followed by dexamethasone 10 mg/m^2/day on days 8–14 and days 22–28). In children with standard risk ALL and in younger children (< 10 years) with intermediate risk ALL, induction prednisolone is randomised between the standard 4-week schedule and the pulsed schedule to examine the impact of a shorter prednisolone schedule on rates of infection, induction deaths and other steroid-related toxicities during the induction phase.

The delayed intensification (DI) phase combines the induction and consolidation treatment phases. The corticosteroid administered in this phase is dexamethasone while doxorubicin is conventionally the prescribed anthracycline. After induction, the highest frequency of sepsis and deaths occur in DI [8, 21]. Reducing the duration and treatment intensity in DI does not appear to alter toxicity rates significantly but appears to be associated with poorer disease-free survival, especially in standard risk patients [22]. The anthracycline mitoxantrone has been associated with significant improvements in long term event-free survival in children with relapsed ALL [23, 24], without excess treatment-related toxicities [24, 25]. In ICiCLe-ALL-14, all patients are eligible during DI for randomisation between a single dose of intravenous mitoxantrone (10 mg/m^2, day 1) versus the standard 3-dose schedule of intravenous doxorubicin (25 mg/m^2/dose; days 1, 8, 15). The hypothesis is that administration of a single dose of mitoxantrone will result in lower rates of myelosuppression and sepsis in DI, as well as improve the probability of event-free survival owing to mitoxantrone's potent anti-leukaemic activity.

Intervention description {11a}

Risk stratification

Following diagnosis of ALL, patients are categorised into four risk groups based on immunophenotype, age,

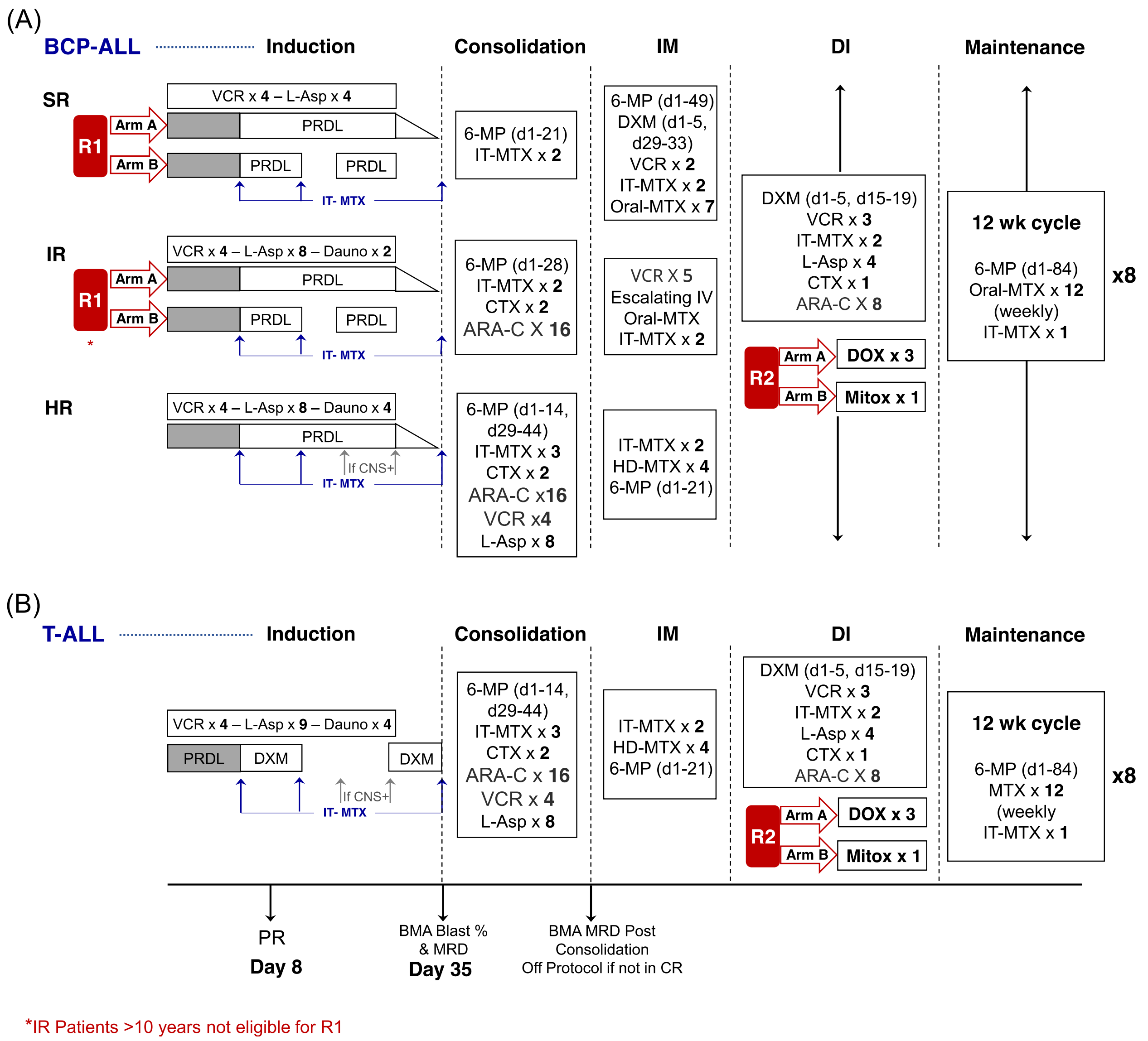

Fig. 2 Schematic representation of risk-stratified treatment and randomised interventions in ICiCLe-ALL-14. Patients 1–18 years old with newly diagnosed acute lymphoblastic leukaemia (ALL) are categorised into four risk groups based on presentation features, disease genetics and treatment response: B cell precursor ALL (BCP-ALL) with standard (SR), intermediate (IR) and high risk (HR) disease (**A**) and T-ALL (**B**). All risk groups receive four consecutive blocks of intensive treatment (induction including a 7-day prednisolone prophase, consolidation, interim maintenance [IM] and delayed intensification [DI]), followed by 24 months of maintenance. Treatment intensity varies by risk group and is highest in HR and T-ALL patients. Treatment response is assessed by determining the prednisolone response (PR) at treatment day 8 and by serial bone marrow assessments (BMA, including microscopy studies and minimal residual disease estimation) at the end of the induction and consolidation treatment phases. Patients with persistent disease at the end of the consolidation phase are withdrawn from the study. Younger (< 10 years) SR and IR patients are randomised to receive the standard continuous schedule (R1 arm A) of prednisolone (4 weeks followed by taper) versus a shorter pulsed prednisolone schedule (R1 arm B, days 1–14, days 22–28) in induction. A second randomisation open to all risk groups randomises patients to receive either the standard 3 doses of doxorubicin (R2 arm A, DOX) or 1 dose of mitoxantrone (R2 arm B, Mitox) in DI. 6-MP, 6-mercaptopurine; ARA-C, cytarabine; CNS+, with central nervous system leukaemia; CTX, cyclophosphamide; Dauno, daunorubicin; DXM, dexamethasone; HD-MTX, high dose intravenous methotrexate; IT-MTX, intrathecal methotrexate; IV, intravenous; L-Asp, *E. coli* L-asparaginase; MRD, minimal residual disease; MTX, methotrexate; PRDL, prednisolone; VCR, vincristine; wk, week

presentation white blood cell (WBC) count, disease bulk, extramedullary disease, cytogenetics and treatment response. Immunophenotype identifies patients with B cell-precursor ALL (BCP-ALL) and T cell ALL (T-ALL). Using the National Cancer Institute (NCI) criteria, BCP-ALL patients are categorised as NCI-standard risk (age < 10 years and highest presentation WBC count < 50 × 10^9/L) and NCI-high risk (age ≥ 10 years and/or highest

presentation WBC count ≥50 × 10^9/L). Bulky disease (indicating high disease burden) includes patients with significantly enlarged liver and/or spleen (at or beyond the level of the umbilicus), enlarged peripheral lymph nodes (≥ 5 cm at longest diameter), bulky mediastinal mass (≥ one third of the transverse thoracic diameter on a chest radiograph) and, in boys, presence of testicular disease. CNS leukaemia is diagnosed in patients with abnormal cerebrospinal fluid (CSF) findings (CSF pleocytosis [≥ 5 white cells/μL in an atraumatic tap] with unequivocal blasts on CSF cytospin) and/or clinical features of CNS disease (e.g. cranial nerve palsies). Cytogenetic studies include fluorescence in situ hybridisation (FISH) [26] assays for recurrent gene fusions, rearrangements and amplifications (*ETV6-RUNX1*; *BCR-ABL1*; *KMT2A* rearrangements; other *ABL*-class rearrangements; intrachromosomal amplification of chromosome 21 [iAMP21]; *TCF3-HLF*) and screening for aneuploidies (specifically hypodiploid ALL, modal chromosome number < 40) by DNA ploidy analysis (flow cytometry) and/or conventional karyotyping. Treatment response is assessed by evaluating the prednisolone response [27] and by assessing the remission status and levels of MRD in the bone marrow following completion of the induction treatment phase. Prednisolone response is assessed at treatment day 8 following 7 days of prednisolone monotherapy (60 mg/m^2/day, minimum cumulative prednisolone dose 210 mg/m^2). Poor prednisolone response refers to patients with circulating lymphoblast count ≥ 1000/μL following 7 days of prednisolone monotherapy. Remission at the end of induction is achieved when patients have no clinical evidence of disease, have satisfactory blood counts and have less than 5% blasts on bone marrow microscopy. MRD is estimated by flow cytometry analysis of the bone marrow (8–10 antigen panel) and a threshold level of 0·01% categorises patients as low (< 0·01%) and high (≥ 0·01%) MRD [28].

Risk groups

Using the above risk stratification criteria, four risk groups are defined:

(a) BCP-ALL Standard Risk (SR)

(i) Patients with NCI-standard risk BCP-ALL
(ii) No bulky disease, CNS leukaemia or high-risk genetics
(iii) Satisfactory treatment response (including good prednisolone response, remission at the end of induction, bone marrow MRD at end of induction < 0·01%)

(b) BCP-ALL Intermediate Risk (IR)

(i) BCP-ALL, with either NCI-high risk or bulky disease features
(ii) No CNS leukaemia, no high-risk genetics
(iii) Satisfactory treatment response

(c) BCP-ALL High Risk (HR)

BCP-ALL with any of the following features:

(i) CNS leukaemia
(ii) High-risk genetics (*BCR-ABL1*, *KMT2A* rearrangement, iAMP21, hypodiploidy, *TCF3-HLF*)
(iii) Poor treatment response, including any of the following: poor prednisolone response, non-remission at end of induction, high MRD level at end of induction
(iv) Insufficient information for risk stratification, including patients with abrogated prednisolone response and with atypical clinical features (e.g. extramedullary disease at unusual sites)

(d) T-ALL
All patients with T-ALL, including patients with T-lymphoblastic lymphoma.

Details of interventions are provided in Supplementary Table 2 and are represented schematically in Fig. 2.

Prednisolone prophase (week 1)

Following diagnosis of ALL, treatment is initiated with prednisolone alone (60 mg/m^2/day, divided doses) for 7 days. This phase allows clinical stabilisation and provides time for patients and families to come to terms with the diagnosis and its treatment. The circulating blast count is estimated at the end of 7 days of the prednisolone prophase and identifies patients with good (< 1000 μ/L) and poor (≥ 1000/μL) prednisolone response. Prednisolone response is considered abrogated when other cytotoxic agents are introduced early in treatment, typically in patients requiring urgent cytoreduction.

Induction (week 1–week 5)

This phase lasts 5 weeks and includes the prednisolone prophase. In this phase, all patients receive corticosteroid, vincristine, *E. coli* L-asparaginase (EcASNase) and intrathecal methotrexate. Patients with IR, HR and T ALL also receive the anthracycline daunorubicin (25 mg/m^2/dose). BCP-ALL patients continue to receive prednisolone (60 mg/m^2/day, divided doses). In patients with HR ALL, prednisolone is administered continuously for 4 weeks (days 1–28) followed by a 5-day taper. In older IR patients (age ≥ 10 years), prednisolone is administered in two pulses (days 1–14 and days 22–28, for a total 3 weeks) without taper. In patients with SR ALL and younger (age < 10 years) IR ALL, prednisolone treatment is randomised between the standard continuous schedule and the pulse

schedule. Following the prednisolone prophase, T-ALL patients are administered pulsed dexamethasone (10 mg/m^2/day in divided doses, days 8–14 and days 22–28). In all patients, EcASNase is administered at 10,000 IU/m^2/dose by intramuscular injection (IM) every 72 h. SR patients receive 4 doses of EcASNase (days 18, 21, 24, 27) while all other risk groups receive 8 doses (days 9, 12, 15, 18, 21, 24, 27, 30). Two doses of daunorubicin are administered to IR patients (days 8, 15) while HR and T-ALL patients are administered 4 doses (days 8, 15, 22, 29). All risk groups receive 4 doses of intravenous vincristine (1·5 mg/m^2/dose) at weekly intervals. Intrathecal methotrexate (IT-MTX, age-based dosing) is administered at days 8, 15 and 35 (typically with the end-of-induction bone marrow). Additional doses of IT-MTX are administered in patients with CNS disease (weekly until clearance of blasts in two successive CSF samples) and in patients with equivocal CSF findings from traumatic lumbar punctures.

Treatment response is assessed at the end of the induction phase.

Consolidation (SR, weeks 6–8; IR, weeks 6–10; HR and T-ALL, weeks 6–14)

The duration of this phase varies by risk group (SR, 3 weeks; IR, 5 weeks; HR and T-ALL, 9 weeks). SR patients receive 6-mercaptopurine (6MP) alone, similar to the UK and the North American Children's Oncology Group (COG) protocols [9, 29]. The consolidation schedule in IR patients is based on the Berlin-Frankfurt-Münster (BFM) protocol IB block [30]. HR and T-ALL patients are administered the COG's augmented BFM IB schedule [31]. In SR patients, 6MP (60 mg/m^2/day) is administered for 21 days during the consolidation phase, together with two IT-MTX treatments (days 8, 15). The BFM IB schedule in IR patients spans five weeks and includes four weeks of daily 6MP (60 mg/m^2/day, days 1–28), two doses of intravenous cyclophosphamide (1000 mg/m^2; days 1, 15), sixteen doses of intravenous cytarabine (75 mg/m^2/dose) administered as four consecutive daily doses every week for 4 weeks (days 2–5; days 9–12; days 16–19; days 23–26) and two IT-MTX treatments (days 8,15).

The 9-week augmented BFM IB consolidation schedule in HR and T-ALL patients includes oral 6MP (60 mg/m^2/day), intravenous cyclophosphamide (1000 mg/m^2/dose), intravenous cytarabine (75 mg/m^2/dose), intravenous vincristine (1·5 mg/m^2) and EcASNase (10,000 IU/m^2/dose, IM). Three IT-MTX treatments are administered in this phase (days 1, 8, 29). Cyclophosphamide, cytarabine and 6MP are administered in two 2-week hemi-blocks: hemi-block 1 (cyclophosphamide, day 1; cytarabine, days 2–5 and days 9–12; 6MP, days 1–14) and hemi-block 2 (cyclophosphamide, day 29; cytarabine, days 30–33 and days 37–40; 6MP, days 29–42). Vincristine and asparaginase are administered similarly as two hemiblocks: hemiblock 1 (vincristine, days 16, 23; EcASNase days 15, 18, 21, 24) and hemiblock 2 (vincristine, days 44, 51; EcASNase days 43, 46, 49, 52). Remission status is re-assessed at the end of the consolidation phase.

Interim maintenance (SR, weeks 9–17; IR, weeks 11–18; HR and T-ALL, weeks 15–22)

Following confirmation of continuing remission, all risk groups proceed to interim maintenance. This phase is time-bound with no catch-up for missed treatments. In patients with IR, HR and T-ALL, intravenous methotrexate is the principal cytotoxic agent during this phase. SR patients receive an 8-week schedule of daily oral 6MP (60 mg/m^2/day, days 1–49) and weekly oral methotrexate (20 mg/m^2/dose, once a week except in the weeks of IT-MTX; 7 doses), combined with two pulses of vincristine (1·5 mg/m^2) and corticosteroid (dexamethasone, oral, 6 mg/m^2/day in divided doses, 5 consecutive days) in weeks 1 and 4. IR patients are administered an escalating-dose schedule of intravenous methotrexate without leucovorin rescue at 10-day intervals (total 5 doses, starting at 100 mg/m^2 and increasing by 50 mg/m^2 in each subsequent treatment, to a maximum 300 mg/m^2; days 2, 12, 22, 32, 42) together with vincristine (1·5 mg/m^2). HR and T-ALL patients receive four doses of high-dose methotrexate (HR, 3 g/m^2/dose; T-ALL, 5 g/m^2/dose) [32] with leucovorin rescue at 14-day intervals, together with oral 6MP (25 mg/m^2/dose, days 1–49). Two doses of IT-MTX are administered in SR (days 15, 43) and IR (days 1, 31) patients while HR and T-ALL patients receive 4 doses of IT-MTX, timed with each high-dose methotrexate treatment.

Delayed intensification (SR, weeks 18–24; IR, weeks 19–25; HR and T-ALL, weeks 23–29)

This phase is common to all risk groups. The first 4 weeks is similar to the induction phase and includes a randomised intervention between two anthracycline-class agents offered to all risk groups. The next 2 weeks is similar to the consolidation schedule and includes intravenous cyclophosphamide (1000 mg/m^2, day 29), cytarabine (intravenous or subcutaneous; 75 mg/m^2/dose; daily for 4 days, repeated twice, days 30–33 and days 37–40) and oral 6MP (60 mg/m^2/day, days 29–42). In the first 4 weeks, patients are administered vincristine (1.5 mg/m^2; 3 doses, days 1, 8, 15), corticosteroid (dexamethasone, 10 mg/m^2/day in divided doses; days 1–5 and days 15–19), EcASNase (10,000 IU/m^2, IM; 4 doses, days 4, 7, 10, 13) and IT-MTX (2 doses, days 1 and 15). During this phase, patients from all risk groups are randomised to receive one of two anthracycline-class agents: intravenous doxorubicin (25 mg/m^2/dose; 3

doses on days 1, 8, 15; standard arm) or intravenous mitoxantrone (10 mg/m^2/dose; 1 dose on day 1).

Maintenance (96 weeks; SR, weeks 25–120; IR, weeks 26–121; HR/T-ALL, weeks 30–125)

This phase is common to all risk groups and spans 96 weeks. The 96 weeks are divided into eight 12-week treatment blocks or cycles, with IT-MTX administered in each cycle. Through 96 weeks, patients are administered daily oral 6MP (60 mg/m^2/day) and weekly oral methotrexate (20 mg/m^2/dose). Oral methotrexate is omitted in the weeks of IT-MTX treatments. Drug doses of both antimetabolites are titrated to ensure treatment at maximum tolerated doses with minimum interruptions due to toxicity.

Response assessment

Response to treatment is assessed at the end of the first week of corticosteroid therapy (day 8 prednisolone response) and at the end of the induction and consolidation phases. Remission is a composite measure that includes assessments of clinical, haematological (blood counts and bone marrow microscopy studies) and submicroscopic (i.e. MRD) levels of disease. In select situations, remission is assessed additionally by radiology and CSF studies. SR and IR patients with excess bone marrow blasts (≥ 5%) and/or high levels of MRD (≥ 0·01%) at the end of the induction phase are switched to the high-risk schedule. Patients with persistent disease at the end of the consolidation phase are withdrawn from the study.

Randomisation

The study includes two randomised interventions. The first in induction examines the toxicity impact of a shorter pulsed prednisolone schedule (60 mg/m^2/day, days 1–14 and days 22–28) versus the conventional continuous schedule (60 mg/m^2/day, days 1–28 followed by taper) in SR and younger (age < 10 years) IR ALL patients. The second in delayed intensification examines the survival benefit from substituting doxorubicin (3 doses, 25 mg/m^2/dose; days 1, 8, 15) with mitoxantrone (1 dose, 10 mg/m^2; day 1) in all risk groups.

Withdrawal from study

Patients are withdrawn from the study when protocol-based treatment is no longer feasible or advisable, owing to poor response (i.e. persistent disease at end of consolidation) and/or treatment-related toxicities. Additional reasons for withdrawal include withdrawal of study consent, incorrect risk stratification and treatment abandonment (i.e. non-adherence to prescribed treatment ≥ 12 weeks).

Intrathecal treatment and cranial irradiation

IT-MTX is dosed by age (1–2 years, 8 mg; 2–3 years, 10 mg; ≥ 3 years, 12 mg). SR and IR patients receive a cumulative 17 doses of IT-MTX while HR and T-ALL patients receive a total 20 doses. Patients with CNS disease at diagnosis receive additional IT-MTX doses weekly during induction, until clearance of blasts is established in two successive CSF samples. Patients with CNS disease at diagnosis and older than 3 years are administered 18 Gy cranial irradiation (10 fractions) before the start of the maintenance phase. Following cranial radiotherapy, no further doses of IT-MTX are administered.

Irradiation of other extramedullary disease sites

In boys with testicular disease at presentation, irradiation of both testes (24 Gy, 12 fractions) is recommended when persistent testicular disease is observed at the end of the consolidation treatment phase.

Polyethylene glycol-conjugated EcASNase (PEG-EcASNase)

The treatment protocol provides the option of substituting EcASNase with a suitable PEG-conjugated formulation. One dose of intramuscular PEG-EcASNase (1000 IU/m^2) replaces 4 doses of EcASNase. SR patients receive 2 doses (induction day 16; delayed intensification day 4), IR patients receive 3 doses (induction days 9 and 23, 1 dose in delayed intensification) and HR and T-ALL are administered 5 doses of PEG-EcASNase (2 doses in induction, 2 doses in consolidation [days 16, 44], 1 dose in delayed intensification).

Philadelphia-chromosome positive ALL (Ph+ALL)

In patients with Ph+ALL and other ABL-class fusions, the ABL tyrosine kinase inhibitor imatinib is recommended in combination with cytotoxic chemotherapy. Imatinib is introduced later in induction when the patient's clinical status is more stable and when blood counts have begun to recover. The targeted treatment dose of imatinib is 360 mg/m^2/day. Imatinib is continued through all phases of treatment until completion of the maintenance phase.

Criteria for discontinuing or modifying allocated interventions {11b}

In the majority of patients, drug dose modifications, drug omissions and treatment interruptions are related to drug-associated toxicities. These modifications are permitted at the discretion of the treating physician and are recorded accordingly in the RDES. Patients in whom disease remission is not achieved at the end of consolidation are withdrawn from the study as treatment

failures, although they may continue on the treatment blocks.

Strategies to improve adherence to interventions {11c}

Adherence to the study protocol is assessed via the RDES. Site-specific adherence is monitored by the National Cancer Grid's Contract Research Organisation. A six-monthly trial report is reviewed by an independent data and safety monitoring committee (DSMC). The study reports and the comments from the DSMC are submitted to the institutional review board at the trial coordinating centre (Tata Medical Center).

Relevant concomitant care permitted or prohibited during the trial {11d}

All patients receive supportive care for management of treatment- and disease-related complications, which commonly include transfusion of blood products, management of pain and metabolic disorders, administration of antimicrobials and nutritional support.

Provisions for post-trial care {30}

Following completion of treatment, all patients are followed up periodically to monitor for events or until a minimum 60 months from completion of treatment.

Outcomes {12}

Rationale for outcome measures

Risk-adapted therapy using traditional clinical features (immunophenotype, age, presentation leucocyte count, extramedullary disease) together with contemporary risk variables (tumour cytogenetics and levels of minimal residual disease) is expected to identify patient groups who require lower-intensity chemotherapy to obtain cure. This approach should decrease treatment-related toxicities and deaths, especially during the induction phase of treatment. Risk stratified therapy, when combined with collaborative multi-institutional practice, uniform clinical management as part of a contemporary treatment protocol, and close monitoring and supervision as part of clinical trial practice, is predicted to improve survival outcomes overall. Randomisations have been introduced to examine the impact of interventions anticipated to decrease treatment-related toxicity in lower-risk patients (shorter course of induction prednisolone in young [< 10 years] non-high risk BCP-ALL patients) and decrease rates of relapse (1 dose of mitoxantrone in place of 3 doses of doxorubicin in delayed intensification in all risk groups). Uniformity of practice across the five major participating paediatric cancer centres enables access to contemporary treatment more locally, decreasing delays in diagnosis and treatment, reducing treatment-related costs and contributing additionally to improved outcomes.

Primary outcomes

1. Event-free survival, overall and in all risk groups. Event-free survival will be estimated three years from diagnosis and the estimates will be compared with survival outcomes in patients treated in the pre-trial phase. Events include treatment-related deaths (including deaths in induction), relapses and trial withdrawals (due to non-response, severe toxicity or treatment abandonment)
2. Incidence rates of severe sepsis (NCI CTCAE grade 3 and higher) and deaths in younger (< 10 years) non-high risk BCP-ALL patients randomised to receive a shorter induction course of prednisolone (60 mg/m^2/day, pulsed schedule, days 1-14 and days 22–28) versus the standard induction prednisolone schedule (60 mg/m^2/day, continuous, days 1–28, followed by a 5-day taper).
3. Event-free survival in patients randomised to receive 1 dose of mitoxantrone (10 mg/m^2, day 1) versus the standard 3 doses of doxorubicin (25 mg/m^2/dose; days 1, 8, 15) in delayed intensification. Event-free survival will be estimated at 3 years from randomisation.

Secondary outcomes

1. Proportion of patients who demonstrate good prednisolone response, achieve remission at the end of induction and have low levels of minimal residual disease at the end of induction. This will be examined in all risk groups and in the steroid-randomised patients
2. Incidence rates of severe infection and non-infection toxicities (NCI CTCAE Grade 3 and higher), overall, by treatment phase, by risk groups and by first and second randomisations
3. Proportion of events including treatment-related deaths, relapse and trial withdrawal (due to non-response, severe toxicity or treatment abandonment), overall, by treatment phase, within risk groups and by randomisations
4. Overall survival, 3 years from diagnosis, in all patients, by risk groups and by randomisations

Other outcomes

1. Analysis of variations in patient and disease characteristics, treatment response, treatment-related toxicities and deaths, event rates and survival outcomes among participating centres, as a representation of geographical variations in patient populations and disease features

2. Analysis of cumulative incidence of relapse and long-term survival outcomes (at a median 5 years from diagnosis) to examine patterns of relapse and late treatment-related effects

Participant timeline {13}

The ICiCLe-ALL-14 protocol follows the SPIRIT guidelines for interventional trials (Standard Protocol Items: Recommendations for Interventional Trials). Figure 3 outlines the schedule of study assessments in the protocol. Patients will be assessed regularly from recruitment to the end of treatment. In the absence of study events, patients will continue to be reviewed periodically for a minimum 60 months from diagnosis.

Sample size {14}

Sample size calculations

1. General remarks

Sample size determinations for the randomised interventions have been powered to observe a clinically meaningful difference of 5% or greater with the experimental treatments. An extended pre-trial pilot at participating centres indicated that loss to follow-up is uncommon (less than 5%), and this has therefore not been factored into the estimations of sample size.

2. First randomisation

An estimated 50% of enrolled patients will be eligible for the first randomisation. At an estimated baseline rate of 39 ± 2% for severe infection-related toxicity in induction and after accounting for drop-outs and refusal to participate (< 5%), a projected enrolment of 3056 patients over an accrual period of 4–5 years is estimated to provide the required sample size, (764 patients in each arm) to detect a 7% difference in rates of severe infection (grades 3 and higher) between the randomised arms, with an alpha error of 5% and at a power of 80%.

Schedule of Assessment for patients on ICiCLe-ALL-14 protocol

	At Diagnosis	Day 8 Induction	End of induction	Consolidation	End of Consolidation	Interim Maintenance	End of Interim Maintenance	At start of Delayed Intensification	End of Delayed Intensification	During Maintenance	End of Treatment	Follow Up
Informed Consent	x							x				
Patient Characteristics	x		x		x		x		x	x	x	
Immunophenotyping	x											
Bone Marrow Morphology	x		x		x							
Flowcytometry based MRD			x		x							
Cytogenetics	x											
Prednisolone Response		x										
CSF Analyses		x										
First Risk Stratification		x										
Steroid Randomization (R1)		x										
Second Risk Stratification			x									
Anthracycline Randomization (R2)								x				
Adverse Events	→	→	→	→	→	→	→	→	→	→	→	→
Survival	→	→	→	→	→	→	→	→	→	→	→	→

Fig. 3 Standard Protocol Items: Recommendations for Interventional Trials (SPIRIT) figure. Schedule of enrolment, interventions, and assessment

3. Second randomisation

An estimated 20% of enrolled patients will experience an event prior to entering the delayed intensification phase and will not be eligible for the second randomisation. At an estimated baseline 3-year EFS estimate of 70% and after accounting for randomisation drop-outs and refusal to participate (< 5%), a projected enrolment of 2004 patients (1002 patients in each arm) over an accrual period of 4–5 years will provide the required sample size to detect a 6% difference in event-free survival between the randomised groups, with an alpha error of 5% and at a power of 80%.

Within the context of the clinical trial, a 5% difference is taken to be clinically significant. The targeted recruitment numbers are powered to detect this difference.

Recruitment {15}

The standard arms of the trial represent standard-of care interventions, minimising the probability of consent refusal. Participating centres have well-established arrangements to provide material and financial support for patients and families, resulting in low rates of treatment abandonment and loss to follow-up. The pre-trial phase additionally provided the opportunity for study centres to strengthen processes for study enrolment and monitoring of patients. The experience gained through the pre-trial phase at study centres has also resulted in low refusal rates for participation in the randomised interventions (≤ 5%).

Assignment of interventions: allocation

Sequence generation {16a}

The study uses a permuted block randomisation design with variable block sizes and 1:1 allocation, stratified by treatment centre. Randomisation is performed centrally through the secure web-based RDES. The first (R1) randomisation is performed on treatment day 8 and involves an open label two-arm randomised design comparing experimental arm R1B (3 weeks of induction prednisolone administered as a pulsed schedule, days 1–14 and days 22–28) against standard arm R1A (4 weeks of induction prednisolone as a continuous schedule followed by taper) in younger (< 10 years) non-high risk BCP-ALL patients. The second open-label randomisation (R2) is performed in delayed intensification and compares 1 dose of mitoxantrone (experimental arm R2B) against 3 doses of doxorubicin (standard arm R2A) in all risk groups.

Concealment mechanism {16b}

Concealment is achieved by randomisation allocations through the RDES.

Implementation {16c}

The entire process from enrolment to assignment of randomisation in the different intervention arms is performed by the web-based RDES.

Assignment of interventions: blinding

Who will be blinded {17a}

ICiCLe-ALL-14 is an open label study. While participants and clinicians will be aware of what treatment is received, the data centre responsible for analyses of the trial is blinded to the actual allocation. Randomisation allocation is done through the RDES and is visible to the centre only at the time of randomisation. This is recorded in the database visible to the trial centre as R1A/B and R2A/B.

Procedure for unblinding if needed {17b}

The randomised data will be unblinded once recruitment is complete. Alternatively, if the trial statistician reports a significant difference in events between the two arms, accepted by the DSMC, then the data will be unblinded.

Data collection and management

Plans for assessment and collection of outcomes {18a}

All trial and treatment-related data are recorded in electronic case report forms (e-CRFs) against unique patient trial numbers. The web-based RDES allows trial registration, enrolment and randomisation in real-time and contemporaneous recording of toxicities and events. Staff at each centre have been trained to use the RDES for trial data management. Data queries raised by the RDES are addressed jointly by the coordinating trials unit and participating trial centres. The coordinating trials unit also organises regular database trawls to evaluate the quality and completeness of data.

Plans to promote participant retention and complete follow-up {18b}

Trial centres have developed institution-specific approaches to monitor and promote adherence of patients to treatment and follow-up [32]. These processes have been further strengthened through experience with the pre-trial phase. Patients who have completed therapy are followed up every 6 months and their clinical status recorded in the eCRF.

Data management {19}

All data are recorded electronically by centres. To verify accuracy of the data, range, validity and consistency checks are performed automatically by the database. Implausible or missing data are corrected or added only after consulting trial investigators, and all corrections are documented. The database will be closed after

termination of the study and following completion of all entries with appropriate documentation of the process.

Confidentiality {27}

Data from each patient is recorded under a unique identifier number instead of patient name so that patient identity remains undisclosed and confidentiality is maintained.

Plans for collection, laboratory evaluation and storage of biological specimens for genetic or molecular analysis in this trial/future use {33}

This activity is not part of the clinical trial.

Statistical methods

Statistical methods for primary and secondary outcomes {20a}

General remarks

Two-sided tests of significance will be used in all analyses. Unless specified, p values less than 0·05 will be considered significant. Randomised interventions will be analysed primarily as modified intention-to-treat (modified to exclude ineligible patients who were randomised). Secondary analyses will include as-treated (all patients administered either the standard or experimental treatments, independent of randomisation) and per-protocol (randomised patients who received the allocated protocol-specified interventions) approaches. These secondary analyses will be performed as part of sensitivity analysis to examine the influence of protocol deviations on study observations, and in the case of as-treated analyses, to evaluate treatment-related toxicities. Cox regression analyses of potential determinants of survival outcomes will be accompanied by tests of the proportional hazards assumption. Poisson regression analysis will be accompanied by testing for dispersion.

Primary outcomes

Primary outcomes measures include the following:

1. For the trial cohort: event-free survival at 3 years from diagnosis

The Kaplan-Meier method will be used to estimate EFS and to plot survival curves. Diagnosis will be from time of trial registration. Events include treatment-related death, relapse and trial withdrawal (due to non-response, severe toxicity or treatment abandonment). EFS will be estimated for the entire cohort and will be stratified by risk group. Survival estimates between groups will be compared by the log-rank test. The influence of risk variables on EFS outcomes will be examined using Cox regression. Risk variables will include patient characteristics (age, sex), disease features (presentation leucocyte count, NCI risk, disease bulk, CNS disease, cytogenetics), treatment response (prednisolone response, complete remission, MRD level at end of induction) and treatment centre.

2. For the first randomisation: Infection and deaths in induction

Rates of severe infection (NCI CTCAE Grade 3 and higher) and deaths in induction will be used as measures of steroid-related toxicity during the induction treatment phase. These will be examined separately (grades 3-4 toxicity, induction deaths) and as a composite endpoint. Incidence rates between the randomised arms will be compared using the Fisher exact test. Association with potential risk variables (e.g. sex, presentation leucocyte count, disease bulk, cytogenetics, risk group, treatment centre) will be examined by Poisson regression.

3. For the second randomisation: EFS at 3 years from second randomisation

The Kaplan-Meier method will be used to estimate EFS and to plot survival curves. EFS will be estimated for all randomised patients and within risk groups. EFS estimates will be compared using the log-rank test. A Cox regression model will be used to examine the influence of risk variables (as outlined above) on EFS outcomes. The influence of the randomised intervention on the risk of relapse and treatment-related death will be examined using a competing-risks model (Fine-Gray), treating relapse and treatment-related death as competing events. Cumulative incidence plots of relapse and treatment-related death will be compared using the Gray test.

Secondary outcomes

Secondary outcome measures include the following:

1. Treatment response

The proportion of patients with good prednisolone response; complete remission at end of induction; low levels of MRD at end of induction; and non-remission at end of consolidation will be reported for all patients, within risk groups and by first randomisation. The Fisher exact test will be used to compare proportions. Logistic regression will be used to examine the influence of presentation risk factors (age, sex, immunophenotype, presentation leucocyte count, disease bulk, cytogenetics, CNS disease, induction risk group, corticosteroid randomisation) on measures of treatment response (induction remission and MRD levels at end of induction).

2. Treatment-related toxicity

Treatment-related toxicity includes severe non-fatal (NCI CTCAE grades 3–4) and fatal toxicities, consequent to infection or otherwise. Recurring non-infection treatment-related toxicities of interest include complications associated with corticosteroid, vincristine and L-asparaginase treatment and select organ-specific toxicities (oral-intestinal mucositis, transfusion-requiring haematological toxicities, cholestatic liver injury and CNS toxicity syndromes). Incidence rates of severe treatment-related toxicities (non-fatal severe, fatal, combined) will be examined for each treatment phase, stratified by risk group, and by second randomisation. The Fisher exact test will be used to compare toxicity rates. The influence of potential risk variables (as outlined above) on incidence rates of toxicity will be examined using Poisson regression.

3. Trial events

Treatment-related death, relapse and trial withdrawal (due to non-response, severe toxicity or treatment abandonment) are recorded as events. Patients are withdrawn from the trial when managed off-protocol (owing to incorrect risk stratification, poor treatment response, severe toxicities) and when non-adherent to treatment (non-adherence for 3 months or more, classified as treatment abandonment). Proportion of events overall and for each of the three categories will be reported for the entire trial cohort, within risk groups, by randomisations and by treatment centres. Proportions will be compared by the Fisher exact test.

4. Overall survival

Estimated using the Kaplan-Meier method at 3 years from diagnosis, for the entire cohort, stratified by risk groups, second randomisation and by treatment centre. The log rank test will be used to compare survival estimates between groups.

Interim analyses {21b}

A single interim analysis is planned 4 years from start of enrolment, when a minimum 30% of events is expected in enrolled patients. The duration of treatment is 2·5 years. As the bulk of relapses occurs ~ 6 months or more following completion of treatment, the major outcome measure at the interim analysis timepoint will be estimation of treatment-related deaths overall and between the randomised arms. The interim analysis will examine the incidence rate of treatment-related deaths and severe non-fatal toxicities between the randomised interventions and determine whether the observed difference exceeds or is within the boundary of tolerable differences on a two-sided Fisher exact test. The boundary of tolerable difference is estimated at 3 standard deviations of the incidence rate overall for treatment-related deaths and severe non-fatal toxicities; surpassing this boundary will be indicated by a p value of 0·001 or less on the Fisher exact test. Event-free survival following second randomisation will also be analysed although here, the number of events will be insufficient to observe a treatment effect and will require the hazard ratio from Cox regression analysis to have a significance level of < 0·00034 to trigger consideration of early termination of the second randomisation.

Methods for additional analyses (e.g. subgroup analyses) {20b}

The following additional analyses will be performed:

1. Patterns of treatment failure following remission-induction

The cumulative incidence of relapse will be examined overall, in risk groups and by treatment centre using a competing-risks model (Fine-Gray), with treatment-related death and trial withdrawal as competing risks. Cumulative incidence curves will be compared using the Gray test. Site of relapse (any bone marrow versus non-marrow sites) and timing of relapse (*very early* within 18 months of diagnosis, *early* between 18 months from diagnosis to within 6 months from end of treatment, *late* more than 6 months from end of treatment) will be reported as proportions overall, within risk groups and by second randomisations. The Fisher exact test will be used to compare proportions.

2. Influence of select covariates on survival outcomes

The influence of continuous risk factors (age, presentation leucocyte counts, prednisolone response, levels of MRD at end of induction) and cytogenetic subtypes (fusion translocations, aneuploidies and amplification in B cell precursor ALL) on survival outcomes will be examined by Cox regression.

3. Interaction of first and second randomisations on EFS outcomes

The first and second randomisations will combine to generate four arms (standard treatment with both randomisations, experimental treatment with both randomisations, experimental treatment in either randomisation). The Kaplan-Meier method will be used to determine estimates of EFS in all four arms and the findings will be compared by the log-rank test.

4. Analysis of steroid-related weight gain in induction

For each patient, changes in weight and body mass index (BMI) will be determined prior to treatment (baseline) and at the end of induction. Weight and BMI values will be reported as percentile, z-score and z-score categories (underweight, normal weight and overweight/obese) using standard age- and sex-matched reference growth charts. Aggregate percentage changes in median weight, weight percentile, z-score and z-score categories will be determined for each risk group and stratified by first randomisation. Changes among groups will be compared by one-way analysis of variance.

5. Treatment delays and relapse

Delays in treatment during the intensive treatment phase (induction to delayed intensification), the maintenance phase and for the entire duration of chemotherapy will be determined for each patient. Logistic regression will be used to examine the influence of treatment delay on remission status.

6. Long-term survival outcomes

Estimates of event-free and overall survival at a median 60 months from diagnosis will be examined using the Kaplan-Meier method to examine late relapses and late treatment effects. The association of risk variables on survival outcomes, including findings from biological sub-studies (somatic gene alterations, constitutional gene polymorphisms, therapeutic drug monitoring) will be analysed using Cox regression.

Methods in analysis to handle protocol non-adherence and any statistical methods to handle missing data {20c}

Robust measures are in place to ensure completeness of data elements critical for analyses of primary and secondary outcomes. The data flow from case report forms in the electronic data capture system ensures capture of all relevant information required for diagnosis, risk stratification, drug treatment, toxicity and treatment response. Completion of data entry in these forms is necessary to progress through trial registration, enrolment, risk stratification and randomisation. Data in the RDES is reviewed, cleaned and updated periodically, both as part of regular data management and in the course of preparing 6-monthly trial reports for review by the data and safety monitoring committee. The objective is to ensure less than 5% missingness in data. For the randomised interventions, the robustness of observations from the primary modified intention-to-treat analysis will be examined using per-protocol and as-treated approaches as secondary analyses. Measures for handling missing data may be required for the exploratory analyses (e.g. association of cytogenetic subtypes with EFS outcomes) and will be addressed using multivariate imputation by chained equations (MICE), assuming data to be missing at random (MAR). For a given variable, missing data will be imputed randomly using the appropriate regression model and iterated to create multiple datasets with imputed values, followed by a pooled estimate derived from statistical analyses performed on each of these imputed datasets.

Plans to give access to the full protocol, participant level-data and statistical code {31c}

Immediately following publication of the results of the study and on reasonable request to the corresponding author, the following information will be made available and will be accessed from the institutional server at the coordinating trial centre: the latest version of the complete trial protocol, the dataset related to the reported study results with appropriate de-identification of study participants, and the accompanying statistical analysis plan.

Oversight and monitoring

Composition of the coordinating centre and trial management committee {5d}

The organisational structure of the trial is shown in Fig. 4. ND, PR, MPG, BA, SC and PD are members of the clinical trials unit (see title page). The RDES has been developed, monitored and maintained by Tata Consultancy Services. The National Cancer Grid Contract Research Organisation is an independent monitoring agency. A detailed monitoring plan is in place. The monitor has access to all study materials needed for source data verification and for review of the study process. All relevant trial-related documentation maintained by participating centres are also reviewed.

Organisational structure and responsibilities
Chief investigator and the clinical trials unit
Design and conduct of ICiCLe-ALL-14
Preparation of protocol and revisions
Preparing reports to data monitoring committee, funding organisations and Institutional Review Board (IRB)
Maintaining database, data verification, randomisation
Organising trial management committee meetings
Managing the Clinical Trials Office
Publication of study reports
Trial Master File management
Trial management committee
Includes lead trial clinicians from participating sites (ShB, SaB, AT, VR, RS, GN, SS, VS and SK; see title page)
Protocol discussions
Review of trial operations (enrolment, data capture)
Review of adverse events
Review of study progress and requirement for protocol modifications
Trial monitoring
National Cancer Grid Contract Research Organisation

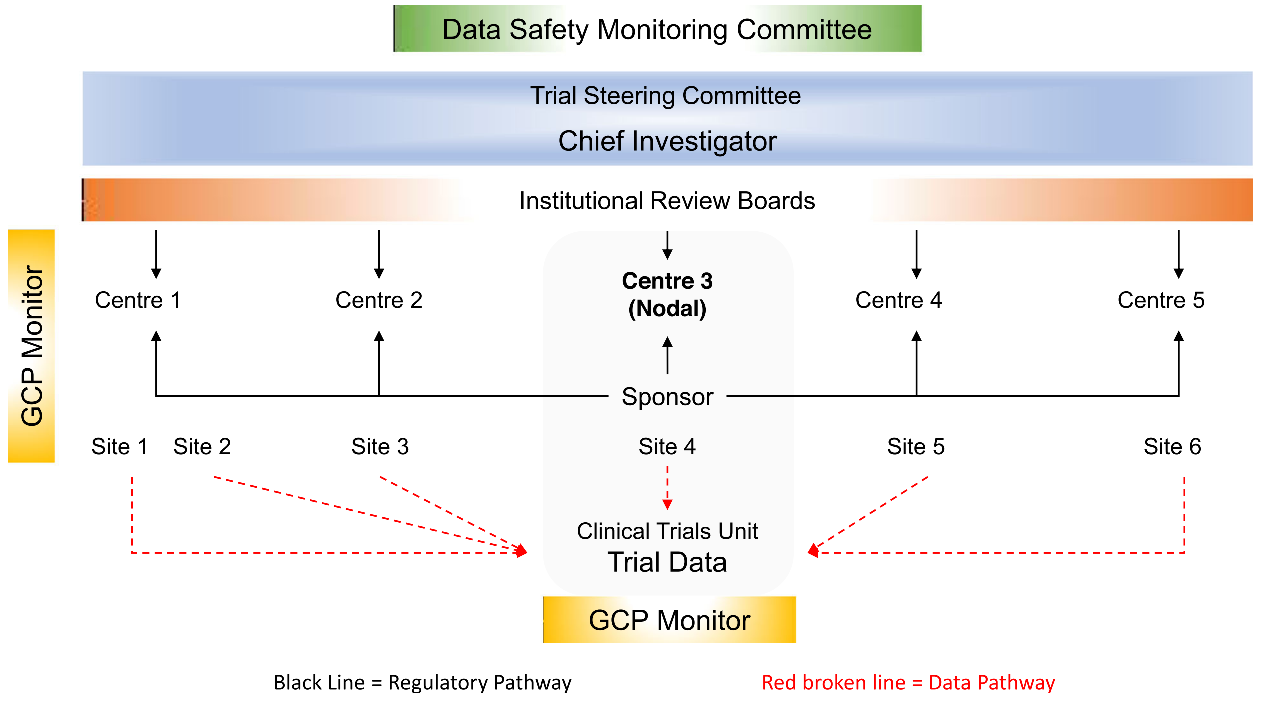

Fig. 4 Overview of the clinical trial management structure in ICiCLe-ALL-14. A Trial Management Committee oversees the daily operations of the clinical trial and includes the chief investigators, principal investigators at participating centres (centres 1–5), the laboratory leads for the specialised diagnostic studies, the trial statistician and the coordinating clinical trials unit. The Trial Management Group reports to the institutional review boards at participating centres and to an independent Data and Safety Monitoring Committee (DSMC). Patients are enrolled at six sites, including two sites at centre 1. Centre 3 functions as the nodal centre for the trial, serving as sponsor and housing the coordinating clinical trials unit. An independent contract research organisation monitors participating centres for adherence to Good Clinical Practice (GCP) standards for clinical trials

Composition of the data monitoring committee, its role and reporting structure {21a}

The independent data and safety monitoring committee (DSMC) includes two international clinical trialists and a statistician with expertise in collaborative multicentre trials in childhood acute lymphoblastic leukaemia. The DSMC receives 6-monthly updates on trial enrolment and randomisation, data recording status, trial events, treatment-related toxicity and serious adverse events (SAE). The trial recruitment committee and the co-ordinating clinical trials unit responsible for analysing data are blinded to the randomisation allocations. The role of the DSMC is to review data produced by the clinical trials unit, comment on the observations, request additional information, make recommendations regarding the trial and data collection and report back to the trial coordinating centre and the institutional review boards on their observations and recommendations. Based on the periodic trial reports, the DSMC may advise continuation of the trial, scheduling of an early interim analysis, trial modification, closure of the randomisation or closure of the trial. The trial management committee will then take a decision based on these recommendations and report to the institutional review boards.

Adverse event reporting and harms {22}

The common terminology criteria for adverse events (NCI CTCAE) version 4.03 (published May 28 2009, by US Department of Health and Human Services, National Institutes of Health, National Cancer Institute website: https://evs.nci.nih.gov/ftp1/CTCAE/CTCAE_4.03/) has been used to grade toxicity. Trial specific toxicity data collection is based on a priori knowledge of known toxicities that are expected with the administration of the protocol drugs at each phase. Specific CTCAE grade 3–5 toxicities (listed in Supplementary Table 3) are collected during all phases of therapy and follow-up and reported to local IRBs within 24 h. Additionally, incidence of grade 3–5 toxicities that are not included in the list are also tracked and reported to the IRBs. All SAEs are collated, including determination of causality and outcome, by the central clinical trials unit. This data is presented every 6 months to the DSMC. The association of adverse events (AEs) and SAEs with interventions in the randomised arms will be evaluated using regression analyses.

Frequency and plans for auditing trial conduct {23}

The RDES allows instantaneous validation of data and identification of errors. An independent external

monitor visits sites twice annually with prior notification. The monitor reviews patient consent and enrolment at trials, evaluates the overall quality and completeness of the recorded data and related source documents, interviews investigators and study coordinators and confirms that the trial centre is compliant with Good Clinical Practice (GCP) guidelines for clinical trials and adheres to institutional procedural standards for conduct of clinical trials. The monitor verifies that all adverse events are documented appropriately and are consistent with protocol definitions; reviews the source (clinical) documents and determines whether the data reported in the RDES are complete and accurate. The monitor confirms that the regulatory binder is complete and that all associated documents are up to date. The monitor also advises centres on measures to ensure adherence to ICH-GCP guidelines. At the end of each visit, the monitor shares a report of identified deficiencies and assists the site in resolving these issues.

Plans for communicating important protocol amendments to relevant parties (e.g. trial participants, ethical committees) {25}

Protocol amendments are reviewed by the trial management committee. The clinical trials unit then submits to the institutional review board of the coordinating centre, an amended protocol version bearing a new version number that contains the tracked changes accompanied by a cover letter listing the changes in the protocol and their rationale. Once approved, the amended protocol version is shared with participating centres for approval by the respective institutional review boards. The amended version is then adopted for use at centres and recorded as the version in use in the trial master file; earlier versions of the protocol are then removed from use and archived.

Dissemination plans {31a}

The decision to publish results of the trial will be taken by the trial management committee. Papers will be produced by a writing group and approved by the trial management committee prior to publication. The study outcomes will be circulated to all participating centres and presented to the community regardless of the magnitude or direction of the results.

Discussion

ICiCLe-ALL-14 is the first risk-stratified multi-centre randomised clinical trial in ALL in India. The benefits include modernisation and standardisation of ALL diagnosis and treatment across the major Indian paediatric oncology centres. The resulting uniformity in care serves to reassure patients and families that they will receive the best available standard of treatment irrespective of the treatment centre they choose to travel to. Furthermore, for patients and families who relocate to continue care at the cancer centre closest to home, the transfer of care would be seamless. A uniform treatment protocol also enables specialty centres to develop shared care arrangements with smaller centres. Contemporaneous recording of information using the RDES has enabled capture of high-quality study data. The sharing of knowledge and experience through the multicentre collaboration has allowed continuous refinement of clinical management through sharing of best practice, improving the quality of care at all centres using an evidence-based approach. The risk stratification and treatment strategies have been designed to reduce morbidity associated with treatment and to facilitate outpatient management, decreasing the burden of treatment for patients and families. At all centres, financial support for hospitalisation, treatment, travel and residential stay are facilitated by a number of government and non-governmental agencies. These measures have been shown to reduce treatment abandonment in low-middle income countries [33–35]. Our vision is to demonstrate the benefits of this approach and to widen its adoption across hospitals in India to ensure equitable care in paediatric ALL. Improving treatment access, service delivery and quality of care are necessary to tackle disparities in survival outcomes in childhood ALL [36].

The trial seeks to improve outcomes in childhood ALL in India. There are a number of limitations. Trial eligibility may favour enrolment of patients with better outcomes and may not reflect real-world experience. Absence of centralised standardisation of genetic and MRD testing results in inconsistencies in risk stratification across centres. Treatment centres also vary by catchment areas, hospital accessibility and inpatient capacity, resulting in variations in treatment practice that potentially influence the outcomes of acute treatment-related toxicities such as sepsis. Finally, the quality and availability of key chemotherapy drugs are variable. In the absence of therapeutic drug monitoring, this variability in drug quality may result in suboptimal dosing or excessive drug toxicity.

Trial status

The trial is underway. Study recruitment began on 24 October 2016 and is projected to be completed on 31 March 2022. As of 10 December 2021, 2301 patients have been enrolled in the trial. The current approved version of the trial protocol is version 5.1 (approved by the institutional review board in February 2020).

Abbreviations
AE: Adverse event; ALL: Acute lymphoblastic leukaemia; BCP-ALL: B-cell Precursor Acute Lymphoblastic Leukaemia; CTRI: Clinical Trials Registry-India; CNS: Central Nervous System; CSF: Cerebrospinal Fluid; CTCAE: Common Terminology Criteria for Adverse Events; DI: Delayed Intensification; DSMC: Data and Safety Monitoring Committee; EcASNase: E. coli L-asparaginase; EFS: Event-free Survival; FISH: Fluorescence in situ Hybridisation; HR: High Risk; IR: Intermediate Risk; IRB: Institutional Review Board; IT-MTX: Intrathecal Methotrexate; MRD: Minimal Residual Disease; NCI: National Cancer Institute; OS: Overall Survival; SAE: Serious Adverse Event; SR: Standard Risk; WBC: White Blood Cell

Supplementary Information
The online version contains supplementary material available at https://doi.org/10.1186/s13063-022-06033-1.

Additional file 1: Supplementary Table 1. List of centres participating in the ICiCLe-ALL-14 trial. **Supplementary Table 2.** The Indian Collaborative Childhood Leukaemia (ICiCLe) study group protocol for risk-stratified treatment of patients aged 1-18 years with first diagnosis of acute lymphoblastic leukaemia (ALL). **Supplementary Table 3.** SPIRIT item 3. **Supplementary Figure 1.** Schema of randomisation in the ICiCLe-ALL-14 protocol.

Acknowledgements
We thank all children and families participating in the study; the clinical and laboratory teams at the centres supervising their care; CanKids KidsCan, Jiv Daya Foundation, St Jude India Childcare Centres and the Sahayta Cancer Sahyog (Chandigarh) for providing support to families; and the National Cancer Grid Contract Research Organisation for study site monitoring.

Authors' contributions {31b}
Authorship eligibility for trial publications will be based on CRediT guidelines [37] as recommended by ICJME journals. The role of all authors will be listed according to the different categories in the submitted manuscript. Authorship will be finalised by the Trial Committee and the corresponding author is responsible for ensuring that the role of each author is accurately described. The final version of the paper(s) requires agreement from all authors prior to submission. There are no plans to use the services of a professional writer. VS, ShB, SaB, AT, VR, RS, BrA, GN and SK wrote the protocol, designed the study and supervise treatment. ND, MPG, SC, BA, PD collect and curate data. ND, VS and SK wrote the manuscript. VS has full access to all data and the final responsibility for publication of protocol and study outcomes. All authors reviewed the manuscript draft submitted for publication. The authors have read and approved the final manuscript.

Funding {4}
Indian Council of Medical Research had part-funded the trial for initial 2 years. (79/159/2015/NCD-III; 21 March 2017) and National Cancer Grid sanctioned funds for 4 years (2016/001, 10 August 2016). Funding from the National Cancer Grid has been approved for 1-year post closure of the trial. The clinical trials unit is part supported by a DBT-Wellcome Trust Margdarshi grant (IA/M/12/500261) to VS. The funding organisations had no role in the design of the study protocol, conduct of the study or analysis of data.

Availability of data and materials {29}
The full protocol with datasets used and/or analysed during the current study will be made available from the corresponding author upon reasonable request at the time of publication.

Declarations

Ethics approval and consent to participate {24}
The protocol has been reviewed and approved by IRBs of all participating centres. Consent is obtained from each participant at each relevant time point during the study.

Consent for publication {32}
The chief investigator and the trial management committee reserve the right to approve publication of study results or any trial related data.

Competing interests {28}
The authors declare that they have no competing interests.

Author details
[1]Clinical Research Unit, Tata Translational Cancer Research Centre, Tata Medical Centre, 14 MAR (E-W), New Town, Kolkata, West Bengal 700160, India. [2]Department of Pediatric Oncology, Tata Memorial Centre, Tata Memorial Hospital, Mumbai, Maharashtra 400012, India. [3]Homi Bhabha National Institute, Mumbai, Maharashtra 40094, India. [4]Department of Medical Oncology, All India Institute of Medical Sciences, New Delhi 110029, India. [5]Pediatric Hematology-Oncology Unit, Department of Pediatrics, Advanced Pediatrics Center, Postgraduate Institute of Medical Education and Research, Chandigarh 160012, India. [6]Department of Medical Oncology, Cancer Institute (WIA), Adyar, Chennai, Tamil Nadu 600020, India. [7]Department of Pediatrics, All India Institute of Medical Sciences, New Delhi 110029, India. [8]Department of Statistics, Tata Medical Center, Kolkata, West Bengal 700160, India. [9]Tata Consultancy Services, Kolkata, West Bengal 700135, India. [10]Department of Pediatric Oncology, Tata Medical Center, Kolkata, West Bengal 700160, India. [11]Division of Cancer Sciences, School of Medical Sciences, Faculty of Biology, Medicine and Health, University of Manchester, Manchester M20 4BX, UK.

Received: 24 May 2021 Accepted: 15 January 2022
Published online: 31 January 2022

References

1. Hunger SP, Mullighan CG. Acute lymphoblastic leukemia in children. N Engl J Med. 2015;373(16):1541–52. https://doi.org/10.1056/NEJMra1400972.
2. Pui CH, Yang JJ, Hunger SP, Pieters R, Schrappe M, Biondi A, et al. Childhood acute lymphoblastic leukemia: progress through collaboration. J Clin Oncol. 2015;33(27):2938–48. https://doi.org/10.1200/JCO.2014.59.1636.
3. Magrath I, Shanta V, Advani S, Adde M, Arya LS, Banavali S, et al. Treatment of acute lymphoblastic leukaemia in countries with limited resources; lessons from use of a single protocol in India over a twenty year period [corrected]. Eur J Cancer. 2005;41(11):1570–83. https://doi.org/10.1016/j.ejca.2004.11.004.
4. Radhakrishnan V, Gupta S, Ganesan P, Rajendranath R, Ganesan TS, Rajalekshmy KR, et al. Acute lymphoblastic leukemia: a single center experience with Berlin, Frankfurt, and Munster-95 protocol. Indian J Med Paediatr Oncol. 2015;36(4):261–4. https://doi.org/10.4103/0971-5851.171552.
5. Trehan A, Bansal D, Varma N, Vora A. Improving outcome of acute lymphoblastic leukemia with a simplified protocol: report from a tertiary care center in north India. Pediatr Blood Cancer. 2017; 64(4). doi: 10.1002/pbc.26281. Epub 2016 Oct 20.
6. Marwaha RK, Kulkarni KP, Bansal D, Trehan A. Pattern of mortality in childhood acute lymphoblastic leukemia: experience from a single center in northern India. J Pediatr Hematol Oncol. 2010;32(5):366–9. https://doi.org/10.1097/MPH.0b013e3181e0d036.
7. Mallath MK, Taylor DG, Badwe RA, Rath GK, Shanta V, Pramesh CS, et al. The growing burden of cancer in India: epidemiology and social context. Lancet Oncol. 2014;15(6):e205–12. https://doi.org/10.1016/S1470-2045(14)70115-9.
8. O'Connor D, Bate J, Wade R, Clack R, Dhir S, Hough R, et al. Infection-related mortality in children with acute lymphoblastic leukemia: an analysis of infectious deaths on UKALL2003. Blood. 2014;124(7):1056–61. https://doi.org/10.1182/blood-2014-03-560847.
9. Bostrom BC, Sensel MR, Sather HN, Gaynon PS, La MK, Johnston K, et al. Dexamethasone versus prednisone and daily oral versus weekly intravenous mercaptopurine for patients with standard-risk acute lymphoblastic leukemia: a report from the Children's Cancer Group. Blood. 2003;101(10):3809–17. https://doi.org/10.1182/blood-2002-08-2454.
10. Mitchell CD, Richards SM, Kinsey SE, Lilleyman J, Vora A, Eden TO, et al. Benefit of dexamethasone compared with prednisolone for childhood acute lymphoblastic leukaemia: results of the UK Medical Research Council ALL97 randomized trial. Br J Haematol. 2005;129(6):734–45. https://doi.org/10.1111/j.1365-2141.2005.05509.x.
11. Moricke A, Zimmermann M, Valsecchi MG, Stanulla M, Biondi A, Mann G, et al. Dexamethasone vs prednisone in induction treatment of pediatric ALL: results of the randomized trial AIEOP-BFM ALL 2000. Blood. 2016;127(17):2101–12. https://doi.org/10.1182/blood-2015-09-670729.
12. Howard SC, Pedrosa M, Lins M, Pedrosa A, Pui CH, Ribeiro RC, et al. Establishment of a pediatric oncology program and outcomes of childhood

acute lymphoblastic leukemia in a resource-poor area. JAMA. 2004;291(20): 2471–5. https://doi.org/10.1001/jama.291.20.2471.
13. Smith M, Arthur D, Camitta B, Carroll AJ, Crist W, Gaynon P, et al. Uniform approach to risk classification and treatment assignment for children with acute lymphoblastic leukemia. J Clin Oncol. 1996;14(1):18–24. https://doi.org/10.1200/JCO.1996.14.1.18.
14. Krivit W, Brubaker C, Hartmann J, Murphy ML, Pierce M, Thatcher G. Induction of remission in acute leukemia of childhood by combination of prednisone and either 6-mercaptopurine or methotrexate. J Pediatr. 1966; 68(6):965–8. https://doi.org/10.1016/S0022-3476(66)80216-0.
15. Louis J, Sanford HN, Limarzi LR. Treatment of acute leukemia in children and adults. J Am Med Assoc. 1958;167(16):1913–7. https://doi.org/10.1001/jama.1958.02990330009003.
16. Igarashi S, Manabe A, Ohara A, Kumagai M, Saito T, Okimoto Y, et. al. No advantage of dexamethasone over prednisolone for the outcome of standard- and intermediate-risk childhood acute lymphoblastic leukemia in the Tokyo Children's Cancer Study Group L95-14 protocol. J Clin Oncol. 2005; 23(27):6489-6498, DOI: https://doi.org/10.1200/JCO.2005.01.982.
17. Winick N, Martin PL, Devidas M, Shuster J, Borowitz MJ, Paul Bowman W, et. al. Randomized assessment of delayed intensification and two methods for parenteral methotrexate delivery in childhood B-ALL: Children's Oncology Group Studies P9904 and P9905. Leukemia. 2020; 34(4):1006-1016, DOI: https://doi.org/10.1038/s41375-019-0642-2.
18. McNeer JL, Nachman JB. The optimal use of steroids in paediatric acute lymphoblastic leukaemia: no easy answers. Br J Haematol. 2010;149(5):638–52. https://doi.org/10.1111/j.1365-2141.2010.08192.x.
19. Teuffel O, Kuster SP, Hunger SP, Conter V, Hitzler J, Ethier MC, et.al. Dexamethasone versus prednisone for induction therapy in childhood acute lymphoblastic leukemia: a systematic review and meta-analysis. Leukemia. 2011; 25(8):1232-1238, DOI: https://doi.org/10.1038/leu.2011.84.
20. Vora A, Goulden N, Mitchell C, Hancock J, Hough R, Rowntree C, et.al. Augmented post-remission therapy for a minimal residual disease-defined high-risk subgroup of children and young people with clinical standard-risk and intermediate-risk acute lymphoblastic leukaemia (UKALL 2003): a randomised controlled trial. Lancet Oncol. 2014; 15(8):809-818, DOI: https://doi.org/10.1016/S1470-2045(14)70243-8.
21. Inaba H, Pei D, Wolf J, Howard SC, Hayden RT, Go M, et. al. Infection-related complications during treatment for childhood acute lymphoblastic leukemia. Ann Oncol. 2017; 28(2):386-392, DOI: https://doi.org/10.1093/annonc/mdw557.
22. Schrappe M, Bleckmann K, Zimmermann M, Biondi A, Moricke A, Locatelli F, et. al. Reduced-intensity delayed intensification in standard-risk pediatric acute lymphoblastic leukemia defined by undetectable minimal residual disease: results of an international randomized trial (AIEOP-BFM ALL 2000). J Clin Oncol. 2018; 36(3):244-253, DOI: https://doi.org/10.1200/JCO.2017.74.4946.
23. Parker C, Krishnan S, Hamadeh L, Irving JAE, Kuiper RP, Revesz T, et. al. Outcomes of patients with childhood B-cell precursor acute lymphoblastic leukaemia with late bone marrow relapses: long-term follow-up of the ALLR3 open-label randomised trial. Lancet Haematol. 2019; 6(4):e204-e216, DOI: https://doi.org/10.1016/S2352-3026(19)30003-1.
24. Parker C, Waters R, Leighton C, Hancock J, Sutton R, Moorman AV, Ancliff P, et. al. Effect of mitoxantrone on outcome of children with first relapse of acute lymphoblastic leukaemia (ALL R3): an open-label randomised trial. Lancet. 2010; 376(9757):2009-2017, DOI: https://doi.org/10.1016/S0140-6736(10)62002-8.
25. Nickel RS, Keller F, Bergsagel J, Cooper T, Daves M, Sabnis H, et.al. Mitoxantrone as a substitute for daunorubicin during induction in newly diagnosed lymphoblastic leukemia and lymphoma. Pediatr Blood Cancer. 2014; 61(5):810-814, DOI: https://doi.org/10.1002/pbc.24892.
26. Parihar M, Singh MK, Islam R, Saha D, Mishra DK, Saha V, et al. A triple-probe FISH screening strategy for risk-stratified therapy of acute lymphoblastic leukaemia in low-resource settings. Pediatr Blood Cancer. 2018;65(12): e27366. https://doi.org/10.1002/pbc.27366.
27. Riehm H, Reiter A, Schrappe M, Berthold F, Dopfer R, Gerein V, et.al. Corticosteroid-dependent reduction of leukocyte count in blood as a prognostic factor in acute lymphoblastic leukemia in childhood (therapy study ALL-BFM 83). Klin Padiatr. 1987; 199(3):151-160, DOI: https://doi.org/10.1055/s-2008-1026781.
28. Tembhare PR, Ghogale S, Ghatwai N, Badrinath Y, Kunder N, Patkar NV, et. al. Evaluation of new markers for minimal residual disease monitoring in B-cell precursor acute lymphoblastic leukemia: CD73 and CD86 are the most relevant new markers to increase the efficacy of MRD 2016; 00B: 000-000. Cytometry B Clin Cytom. 2018; 94(1):100-111, DOI: https://doi.org/10.1002/cyto.b.21486.
29. Vora A, Goulden N, Wade R, Mitchell C, Hancock J, Hough R, Rowntree C, et. al. Treatment reduction for children and young adults with low-risk acute lymphoblastic leukaemia defined by minimal residual disease (UKALL 2003): a randomised controlled trial. Lancet Oncol. 2013; 14(3):199-209, DOI: https://doi.org/10.1016/S1470-2045(12)70600-9.
30. Moricke A, Zimmermann M, Reiter A, Henze G, Schrauder A, Gadner H, et.al. Long-term results of five consecutive trials in childhood acute lymphoblastic leukemia performed by the ALL-BFM study group from 1981 to 2000. Leukemia. 2010; 24(2):265-284, DOI: https://doi.org/10.1038/leu.2009.257.
31. Nachman JB, Sather HN, Sensel MG, Trigg ME, Cherlow JM, Lukens JN, et.al. Augmented post-induction therapy for children with high-risk acute lymphoblastic leukemia and a slow response to initial therapy. N Engl J Med. 1998; 338(23):1663-1671, DOI: https://doi.org/10.1056/NEJM199806043382304.
32. Vaishnavi K, Bansal D, Trehan A, Jain R, Attri SV. Improving the safety of high-dose methotrexate for children with hematologic cancers in settings without access to MTX levels using extended hydration and additional leucovorin. Pediatr Blood Cancer. 2018; 65(12):e27241.Holistic support coupled with prospective tracking reduces abandonment in childhood cancers: A report from India. Pediatr Blood Cancer. 2019; 66:e27716
33. Arora RS, Pizer B, Eden T. Understanding refusal and abandonment in the treatment of childhood cancer. Indian Pediatr. 2010;47(12):1005–10. https://doi.org/10.1007/s13312-010-0172-5.
34. Mostert S, Arora RS, Arreola M, Bagai P, Friedrich P, Gupta S, et al. Abandonment of treatment for childhood cancer: position statement of a SIOP PODC Working Group. Lancet Oncol. 2011;12(8):719–20. https://doi.org/10.1016/S1470-2045(11)70128-0.
35. Jatia S, Prasad M, Paradkar A, Bhatia A, Narula G, Chinnaswamy G, et al. Holistic support coupled with prospective tracking reduces abandonment in childhood cancers: a report from India. Pediatr Blood Cancer. 2019;66(6): e27716. https://doi.org/10.1002/pbc.27716.
36. Ward ZJ, Yeh JM, Bhakta N, Frazier AL, Girardi F, Atun R. Global childhood cancer survival estimates and priority-setting: a simulation-based analysis. Lancet Oncol. 2019;20(7):972–83. https://doi.org/10.1016/S1470-2045(19)30273-6.
37. Allen L, O'Connell A, Kiermer V. How can we ensure visibility and diversity in research contributions? How the Contributor Role Taxonomy (CRediT) is helping the shift from authorship to contributorship. *Learned Publishing*. 2019;32(1):71–4. https://doi.org/10.1002/leap.1210.

Publisher's Note

Springer Nature remains neutral with regard to jurisdictional claims in published maps and institutional affiliations.

Ready to submit your research? Choose BMC and benefit from:

- fast, convenient online submission
- thorough peer review by experienced researchers in your field
- rapid publication on acceptance
- support for research data, including large and complex data types
- gold Open Access which fosters wider collaboration and increased citations
- maximum visibility for your research: over 100M website views per year

At BMC, research is always in progress.

Learn more biomedcentral.com/submissions
