# Supplementary material for: Toxicity during induction of pulsed versus continuous prednisolone in children with acute lymphoblastic leukaemia: a multi-centre, open label, randomised, phase 3 trial from India (2016–2022)
Source: Lancet Reg Health Southeast Asia. 2026 Jun 7;50:100788. doi: 10.1016/j.lansea.2026.100788 (PMC13366301; doi:10.1016/j.lansea.2026.100788)
Supplement: ICiCLe Protocol_ Version 5.1 [file mmc3.pdf]

InPOG-ALL-15-01

ICiCLe ALL-14

Indian Childhood Collaborative Leukaemia Group

A collaborative, multicentre, national study for newly diagnosed patients with acute lymphoblastic leukaemia

Type of Study: Randomised open label phase III/IV Study

Protocol version 5.1

CTRI registration number-CTRI/2015/12/006434

Please destroy/remove all previous versions of the protocol.

Contents

Contents..2
Investigators and centres 6
Study committee members 7
List of Protocol Amendments..10
Introduction 11
Background: 11
Why have a study?..11
What is the problem to be addressed? 13
Treatment stratification..14
Treatment strategy 14
T-CELL ALL 15
What is the study design and what questions are being asked? 15
Steroid randomisation: 15
Randomisation of Mitoxantrone with Daunorubicin: 16
References 17
Eligibility Criteria..20
Inclusion Criteria for upfront randomisation: 20
BCP ALL 20
T-Cell ALL..21
Cytogenetics 21
Enrolment 22
Definition of Complete Remission (CR) 23
Criteria for removal of patients from Study Protocol..24
Chemotherapy Protocol..30
Aim..33
Study Design and Sample Size..34
Dose Calculations and Adjustments for BMI..36
B-cell Precursor ALLStandard Risk 39
Standard Risk – Summary Page 40
Standard Risk (SR) Induction Phase 43
Standard Risk (SR) Consolidation Phase 47
Standard Risk (SR) Interim Maintenance Phase 50
Standard Risk (SR) Delayed Intensification Phase 53

Standard Risk (SR) Maintenance Phase 56
B-cell PrecursorALL Intermediate Risk 57
Summary page – Intermediate Risk 58
Intermediate Risk (IR) Induction Phase 62
Intermediate Risk (IR) Consolidation Phase 66
Intermediate Risk (IR) Interim Maintenance Phase- Intravenous MTX 69
Intermediate Risk (IR)Delayed Intensification Phase 72
Intermediate Risk (IR) Maintenance Phase 75
B-cell Precursor ALL High Risk 76
Summary page – High Risk 77
High Risk (HR) Induction Phase 79
High Risk (HR) Consolidation Phase 83
High Risk (HR) Interim Maintenance Phase 87
High Risk (HR)Delayed Intensification Phase 90
HighRisk (HR) Maintenance Phase 93
T-cell 94
Summary Page 95
T-cell Induction Phase 97
T-cell Consolidation Phase 101
T-cell Interim Maintenance, HDMTX Phase 105
T-cell Delayed Intensification Phase 108
T-cell Maintenance Phase 111
Ph positive ALL 112
Glucose-6-Phosphate Dehydrogenase (G6PD) Deficiency 113
Pharmacology and Drug Toxicity 114
Anthracyclines 115
Appendix 1: Approximate cost of drugs 122
Appendix 2: Statistical Plan 123
Introduction 123
Aim of the Trial 123
Participating Centres 123
Study Design 123
Risk Stratification 123
Study Population 124

Exclusion Criteria 124
BCP-ALL-SR..124
BCP-ALL-IR..124
BCP-ALL-HR..125
Definitions..125
Complete Remission 125
Induction death 125
Non-response 125
Death in remission 125
Treatment Related Mortality (TRM)..125
Relapse Death..125
Relapse 125
Secondary malignancy..125
Event Free Survival (EFS) 126
Overall Survival (OS) 126
Withdrawal 126
Toxicity 126
Abandonment 126
Study Objectives 126
Primary Objectives..126
Secondary Objectives..126
Randomistion..127
Sample Size Calculation 127
Randomisation 1 (R1) 127
Randomisation 2 (R2) 129
Data Entry and Data Management..130
Data collection/Case Report Form (CRF) 130
Source Data and Patient Files..130
Data Processing..130
Monitoring..131
Audits/Inspections..131
Data and Safety monitoring Committee and Interim Analyses..131
Interim Safety Analysis 132
Interim efficacy analysis..133

Early stopping rules 133
Descriptive Analyses 133
The Study Population 133
Baseline characteristics 134
Comparison of Losses to Follow-up 135
Primary Analyses 136
Randomisatoion 1 136
Randomisation 2 136
Survival Analyses 136
Missing Data 137
Secondary Analyses 137
Reporting Adverse Events 137
Appendix 3: Intravenous MTX-Interim Maintenance 138
Appendix 4: Administration of High Dose Methotrexate 139
Appendix 5:DosingRecommendations For Erwinia Asparaginase 143
Appendix 6:Recommendations for Cerebrospinal Fluid analysis 144
Appendix 7: MRD 146
Appendix 8: Cytogenetics 147
Appendix9: Definition of Adverse Events 150
Appendix 10: Philadelphia Positive (Ph+) Acute Lymphoblastic Leukaemia/BCR-ABL 152
Appendix 11: Thrombo-embolic events 153
Background 153
Screening 153
Catheter (CVL) Related Thrombosis 153
Loss of CVL patency 153
CNS Thrombosis 156
Appendix 12: Guidelines for Intrathecal Therapy 157
Appendix 13: Radiotherapy 158
Appendix 14: Guidelines for antimetabolite dosing during maintenance for all patients 161
Appendix 15: Notes for Ethics Committees 163
Synopsis 163
The Management Structure 165

Appendix 16: Informed Consent Template……………………………………………………………………………167
Appendix 17: Assent form, PIS…………………………………………………………………………………………170
Appendix 18: Assent Form……………………………………………………………………………………………….174
Appendix 19: Dose Rounding Rules 175
Appendix 20: Toxicities (Descriptions & Grading)……………………………………………………………..176
Appendix 21: Declaration of Helsinki………………………………………………………………………………..177
Appendix 22: IAP Body Mass Index Reference Charts_2015………………………………………………..182
Appendix 23: Trial Withdrawal, Treatment abandonment, Lost to follow-up…………………………………184

Investigators and Centres

**New Delhi**	All India Institute of Medical Science	Prof. Sameer Bakhshi<br>Dr. Rachna Seth
	MAX Superspecialty	Dr. Ramandeep Arora
**Chandigarh**	Post Graduate Institute of Medical Education and Research	Dr. Amita Trehan
**Mumbai**	Tata Memorial Centre	Prof. Shripad Banavali<br>Prof. Gaurav Narula<br>Dr Girish Chinnaswamy
**Kolkata**	Tata Medical Center:	Prof. Shekhar Krishnan<br>Prof. Vaskar Saha
**Chennai**	Adyar Cancer Institute:	Prof. TG Sagar<br>Dr. R Venkataraman

Study committee members

Chief Investigator: Vaskar Saha
Tata Medical Center
14 Main Arterial Road (EW), Rajarhat, New Town
Kolkata 700160
Email: vaskar.saha@tmckolkata.com

Tel: 033 66057001

Co-Chief Investigator: Shripad Banavali
Tata Memorial Hospital
Parel, Mumbai-400012
Email: banavali2000@yahoo.com

Tel: +91222417-7000

Chemotherapy: Shekhar Krishnan
Tata Medical Center
14 Main Arterial Road (EW), Rajarhat, New Town
Kolkata 700160
Emails: shekhar.krishnan@tmckolkata.com
arpita.bhattacharyya@tmckolkata.com

Tel: +91336605-7001

Pharmacology and Toxicity: Amita Trehan
Additional Professor, Paediatric Haematology Oncology Unit,
Advanced Paediatric Centre,
Post Graduate Institute of Medical Education & Research,
Chandigarh 160012, India
Email: trehanamita@hotmail.com

Tel: +91(172) 2755312

Study Design and Funding: Sameer Bakhshi

All India Institute of Medical Sciences, New Delhi-110 029 (AIIMS)
Department of Medical Oncology
Room No. 243
Dr. B. R. A. Institute Rotary Cancer Hospital
All India Institute of Medical Sciences
New Delhi-110 029, INDIA
Email: sambakh@hotmail.com

Tel: +919958828763

Study Statistician: Subir Sinha
Tata Medical Center
14 Main Arterial Road (EW), Rajarhat, New Town
Kolkata 700160
Email: subir.sinha@tmckolkata.com

Tel: +91-33-6605-7197

Minimal Residual Disease: Praveen Amre
PG Subramaniam
Hematopathology Laboratory,
Tata Memorial Hospital,
Parel, Mumbai-400012
Email: pgs_mani@yahoo.com

Tel: +91-22-2417-7000, extension 4367, 4362

Deepak Mishra
Tata Medical Center
14 Main Arterial Road (EW), Rajarhat, New Town
Kolkata 700160
Email: deepak.mishra@tmckolkata.com

Tel: +91-33-6605-7754

Cytogenetics: Mayur Parihar

Consultant, Cytogenetics (Lab Medicine)
Tata Medical Center
14, Main Arterial Road (EW) Rajarhat, New Town
Kolkata 700160
Email: mayur.parihar@tmckolkata.com

Tel:+91-33-6605-7755

Radiotherapy Advisor: Rimpa BasuAchari
Tata Medical Center
14, Main Arterial Road (EW) Rajarhat, New Town
Kolkata 700160
Email:rimpa.achari@tmckolkata.com

Tel:+91-33-6605-7102

Data Monitoring Committee: Professor Ajay Vora (UK)
Professor of Paediatric Haematology
The University of Sheffield

Dr Anja Möricke (Germany)
Study Coordinator BFM
University Hospital Schleswig-Holstein

Professor Meenakshi Devidas (USA)
Children’s Oncology Group
Department of Biostatistics
6011 NW 1st Place
Gainesville, FL 32607-2025

Dr C S Pramesh, MS, FRCS
Chief, Thoracic Surgery
Professor, Department of Surgical Oncology
Tata Memorial Centre
Dr Ernest Borges Road, Parel
Mumbai 400012, India

Prof (Lt Col) Dr ATK Rau
Professor and Head
Paediatric Haematology-Oncology,
Department of Paediatrics
MS Ramaiah Medical College
Bangalore, Karnataka 560054

List of Protocol Amendments

Version No.	Version date	Summary of changes
2.0	5 May 2017	- Change of PI from Dr. Brijesh Arora to Dr. Gaurav Narula in Tata Memorial Centre, Mumbai<br>- Addition of the line " Ph+ and ABL class fusion as heading<br>- Edits in text on weight instructions and BMI calculation, addition of new lines<br>- Addition Appendix 20: IAP Body Mass Index Reference Charts_2015<br>- Addition of the word Randomisation 2 in the Consent Form
3.0	5 Dec 2017	- T cell ALL Standard Risk group removed from protocol<br>- Revision of definition of Serious Adverse Event<br>- Details of Cytogenetics testing and Minimal Residual Disease estimation added<br>- Information on risk stratification for CNS disease added
3.1	15 Mar 2018	- Addition of definition of 'Prednisolone response abrogated'<br>- Addition of the definition of 'CNS Disease'<br>- Addition of Appendix 23-Trial Withdrawal, Treatment Abandonment, Lost to Follow Up<br>- Addition of Appendix 17- Patient information sheet on Assent<br>- Addition of Appendix 18- Assent form
4.0	3 May 2018	- Inclusion of MPAL as an exclusion criteria<br>- Modification of the definition of bulky disease<br>- Addition of two new members to DSMC<br>- Recommendation for evaluation of minimal residual disease at the end of consolidation phase<br>- Categorisation of patients with insufficient risk stratification as High Risk and treatment on trial<br>- List of amendments
4.1	8 August 2018	- Change in Chromosome number for Hypodiploidy
5.0	25 July 2019	- Replacement of the term "Phase IV" with "Phase III/IV" in type of study.<br>- Change of Pin code of Tata Medical Centre Kolkata from 700156 to 700160<br>- Modification in the description of enrolment.
5.1	7 January 2019	- Modifications under Appendix 2: Statistical Plan

Introduction

Background:

Acute Lymphoblastic Leukaemia (ALL) is the most common cancer of childhood worldwide with the exception of Sub-Saharan Africa. It is estimated that over 10,000 cases of childhood ALL occur in India every year (1). The lack of a national registry (1), health inequalities, and changing population demographics (2) suggests that this is probably an underestimate.

Figure 1

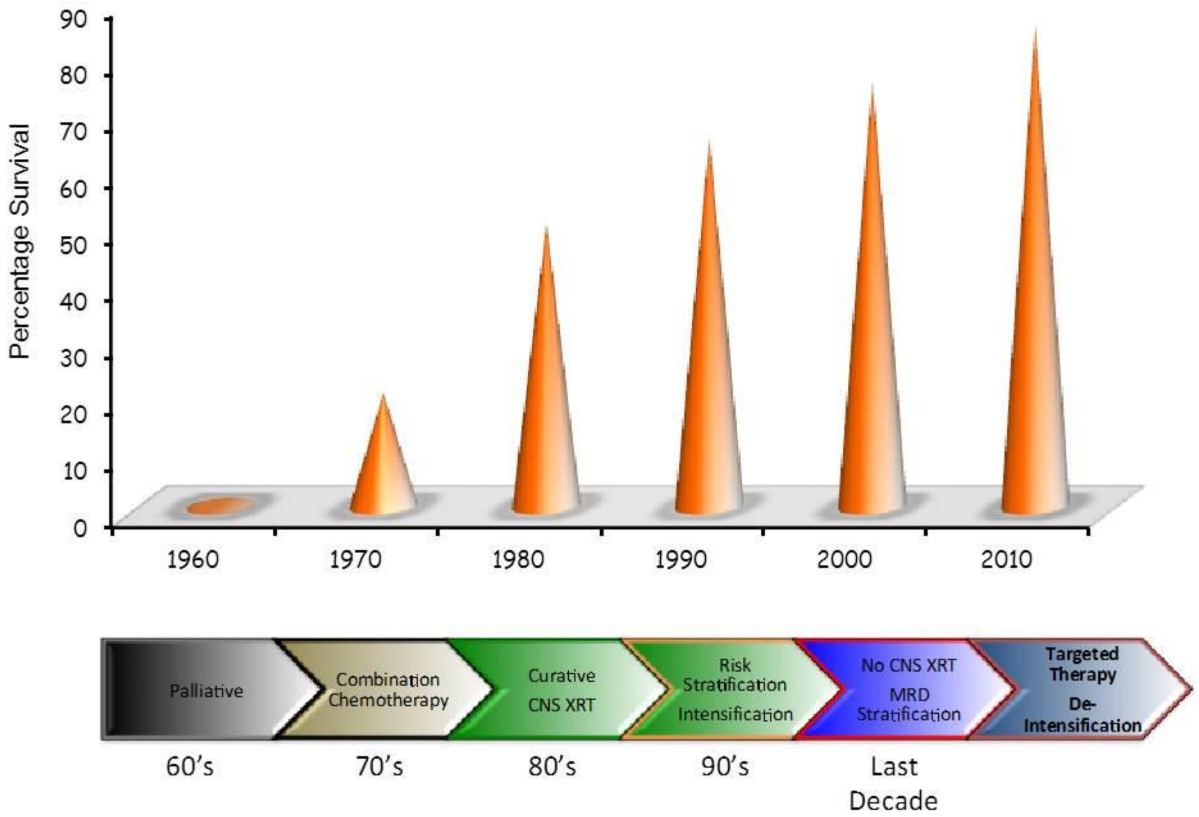

We have been using the same drugs to treat childhood ALL for over 4 decades. In the west, outcomes have improved from near certain failure in the 1960's to more than 80% being cured in the modern era. As shown in Figure 1, this has been primarily achieved by risk stratification and intensifying therapy. In India as well there has been an improvement in outcome in the last decade(3), though this is restricted to a few specialised centres. Nevertheless, overall we still lag behind with what can be achieved within our resources.

Why have a study?

The treatment of childhood ALL is one of the most complex and prolonged of any cancers. Over 10 different drugs are administered by a variety of routes, in combination, over a period of 2-3 years. The agents used are broad spectrum cytotoxic agents. Some are highly specific for ALL like L-Asparaginase, others are used widely in many cancers e.g. Doxorubicin. A number of study groups worldwide have used these drugs in different combinations and schedules, with comparable results (4). Thus dissecting the crucial components of therapy is difficult. One option is to adopt a western

protocol for Indian use. This has been done - CMC Vellore reported on 307 patients treated on a BFM (German) protocol over a 18 year period. They reported an event free survival (EFS) of around 60% with a 2% treatment related mortality (TRM) in induction (5). Tata Memorial reported a 60% EFS in 652 patients around the same time with the collaborative Indian protocol, MCP841(6). They however observed a 11% TRM even though the induction protocol is almost identical to that used in Vellore. Outcome was poorer at the other MCP841 centres, with even higher TRM. Another factor that emerged was a geographical variation in age at diagnosis, immunophenotype and outcome. Ethnic variations in treatment response are well documented in the west. In US studies, those with Hispanic backgrounds do the worst, however those from south Asia have the best outcomes(7). In the absence of properly collated data, within the context of an uniform treatment strategy, it is not possible to comment whether there are true ethnic variations to the therapeutic responses in Indian patients and/or geographical variations in access to health.

The data from the 3 MCP841 centres has consistently shown that children in India present late with advanced disease and high disease burdens compared to west. In the pooled data from 3 centres, 75-85% patients presented with significant lymphadenopathy and hepatosplenomegaly. Majority of the patients presented with NCI high risk disease and 15-25% patients presented with hyperleukocytosis in these 3 centres (WBC >100,000/cmm). Quantitation of differences from western populations (apart from WBC) is not as easy as one would like, since in most studies conducted in the affluent countries (apart from some confined to T-cell leukemia), limited information is provided on lymphadenopathy and hepatosplenomegaly, these features being much less frequently observed in such populations. In a recent Children's Cancer Group (CCG) study involving high risk patients only, splenomegaly was observed in approximately 56% of 636 patients, hepatomegaly in 47% and lymphadenopathy in 45%, very few cases being more than moderately enlarged. These figures are markedly lower than the corresponding figures in Indian populations (76-90%). Patients with a WBC of over 50,000 per cumm and age 1-9, or patients age > 10 years were eligible for this same CCG study. In this selected population, 25 and 22% of patients had a WBC between 50 and 99,000 and >100,000 per cu mm. respectively, compared to 11% and 18% of the Indian populations who were not selected on the basis of WBC or age.

Apart from inconsistent impact of WBC count, no other prognostic factor emerged in the large multicentric MCP841study. Apparent lack of a consistent association of WBC with outcome in pre B-ALL in India could reflect differences either in the biology of the leukaemic cells, or in the patient populations, or both. It is possible that the presence of a significant leukaemic burden outside the bone marrow and peripheral blood compartments and increased tumour burden in Indian patients may decrease the impact of presenting WBC as an independent risk factor. Hence, in ICiCLe ALL -14 significant emphasis has been accorded to the extramedullary burden of disease in risk stratification of patients both in B and T-cell disease unlike in the west. (6). Apart from the significantly high leukemic burden, the MCP841 study also showed a lower frequency of favourable genotypes such as ETV6-RUNX1and hyperdiploidy in the Indian population compared to that reported in the west (8, 9). Similarly, the frequency of unfavourable genotypes such as BCR-ABL1was higher (10).

What we have learnt is that inclusion in clinical trials improves outcome (11, 12). This is an observation made across many study groups. Patients are treated at multiple centres on uniform treatment schedules, standardising care across different healthcare systems. The pooled data is

sufficient to make observations that can lead to further beneficial modifications to therapy. Outcome and morbidity is carefully monitored and periodically assessed. Parents and patients are adequately supported. Over time, the network acquires a collective expertise in managing children with ALL. Current protocols in India have been adapted from those used in the west and have improved the outcome of ALL significantly, but have mostly failed to modernise. For e.g. a number of Indian centres continue to use prophylactic cranial irradiation. This modality is no longer used by most western study groups except in some specified high risk groups. A non-irradiative strategy has recently been used successfully in the Malaysia-Singapore ALL 2003 study (13). Furthermore in most western protocols, toxicity of therapy now outweighs the risk of recurrence. Mortality rates in remission approach 3.5% and over a quarter of patients suffer a severe adverse event (personal communication, Professor Vora). Both UK and BFM have begun to seek ways to de-escalate therapy for low-risk patients.

Thus in the west, therapeutic protocols are now being de-escalated. Carefully conducted randomised trials now show that a second delayed intensification block is unnecessary (14). Such serially conducted trials have also identified racial and ethnic differences in outcome (15, 16) allowing strategies to overcome these disparities (17).

There is evidence to show that for a number of reasons, protocols developed in the west do not necessarily produce comparable results in different geographical healthcare systems (18). Moreover in India, because treatment is not standardised, patients often travel longer distances for medical care at a great expense, as they are unsure of where they will receive the best treatment. To improve the outcome of children with ALL in India, we need to come up with indigenous solutions applicable to the majority of patients. This can only be done by a systemic collaborative effort based on a clinical trial.

What is the problem to be addressed?

We need a modern, scientific protocol for Indian centres. The aim is to standardise therapy across the country providing equity of health care. ALL is a curable condition, and a number of state health schemes and charities now provide financial support for treating children with ALL. Nevertheless toxicity of treatment requiring prolonged hospitalisation and cost of supportive therapy along with frequent visits to the tertiary centres places a considerable burden on most families.

In the west, children of Asian origin have among the best outcomes on modern protocols (16). We have learnt that molecular risk stratification is able to identify a group of children who can be cured with minimal therapy (19). As shown in Figure 1, the proposed clinical study named ICiCLe ALL- 14 will incorporate model designs that are now current worldwide. This includes using molecular tools to stratify patients and risk adapted therapy. The goal is to provide optimal therapy for those at low risk of recurrence to avoid unnecessary toxicity. This is a multicentre randomised control study, designed for Indian children and examines whether reducing the duration of steroids during induction therapy; and replacing 3 doses of Doxorubicin in delayed intensification with a single dose of Mitoxantrone, is able to decrease treatment related morbidity without affecting outcome. We anticipate that by standardising treatment, we will be able to deliver event free survival (EFS) of >80% for standard risk (SR), >70% for intermediate risk (IR) and >60% for high risk patients.

Treatment stratification

The study will use internationally accepted risk stratification. B precursor ALL (BCP-ALL) patients will be stratified according to NCI criteria to standard (SR) and intermediate risks (IR). Children with high risk cytogenetics and a prednisolone poor response (20) will be classified as high risk, irrespective of initial risk category. **Minimal residual disease (MRD)** will be estimated by flow cytometry at the end of induction, and those who have MRD $\geq 10^{-4}$ will be treated as high risk post induction (16).

Treatment strategy

BCP-ALL

Recent data show that toxicity of a 4-drug induction is almost twice that of a 3-drug induction(14). SR patients, as defined by MRD, can be treated with non-intensive protocols with survival rates of over 90%(21). Thus, in ICiCLe ALL-14 Standard risk patients will receive a 3-drug induction. If MRD is low/negative, they will continue on standard consolidation, interim maintenance, delayed intensification and continuation therapy. Around 60% of patients will fall into this category. We expect an EFS of >80% in this group.

IR patients will receive a 4-drug induction. They will receive only 2 doses of anthracycline, an approach now being investigated by the BFM group. The use of 4 doses is historical and does not take into account the long term residual effect of anthracyclines bound to tissue. Thus, in this group of patients, the hypothetical benefit of 4 doses of anthracycline is outweighed by the risk of prolonged myelosuppression. IR patients will receive a BFM type consolidation. Based on the recently published data from COG 1991 study all intermediate risk patients will receive escalating intravenous methotrexate in interim maintenance (8).

High risk patients will receive a 4-drug induction with 4 doses of daunorubicin, followed by augmented BFM consolidation, and high dose intravenous Methotrexate based interim maintenance. Both augmented BFM consolidation and the use of intravenous Methotrexate in this population have been associated with a better outcome in BFM, COG and UK trials. There are two options for intravenous Methotrexate. The first, most widely used, is that of high dose Methotrexate (3-5 g/m^2) given as an infusion over 24 hours. The advantages of this approach are, the therapeutic administration is tightly regulated and toxicity can be minimised with folinic acid rescue. Also, this has been shown to significantly improve the outcome in high risk patients in a recent COG study (22).

The disadvantage is that the patient requires to be hospitalised for 5 days and Methotrexate levels need to be monitored. The second, which has been used by both COG and UK, is to use 100 mg/m^2 of Methotrexate as an intravenous push at 10 day intervals. Traditionally, this has also been given with L-Asparaginase and current data suggests that this is more likely to add to toxicity rather than improve outcome. Thus in ICiCle ALL-14, we propose to use the high dose Methotrexate. Since, recent data suggests that 3gm/m^2 may be sufficient in B-cell ALL and can be safely administered without need for therapeutic drug monitoring,we would use 3 gm/m^2 dose in this population.

Recent studies show no benefit for a second delayed intensification block (14) and (personal observations, **Professor Ajay Vora**), so no second delayed intensification blocks will be used. This is

in line with practices adopted by other international study groups. Similarly, the use of vincristine and steroids during continuation therapy has not shown to be of benefit (23) and will not be used in the ICiCle ALL-14 protocol.

T-CELL ALL

All T-cell patients will receive a 4-drug induction.Based on the BFM observation that Dexamethasone is more effective in T-cell ALL (**Anja Möricke**, personal communication) all patients will receive Dexamethasone and not Prednisolone during induction. All T-ALL and T-Lymphoblastic Lymphoma patients will receive 5 gm/$m^2$of Methotrexate during interim maintenance.

What is the study design and what questions are being asked?

We have defined ICiCLe ALL-14 as an open label, randomised phase III/IV study. The reason for classifying this as a phase IVstudy , is that the risk stratification, all drugs and therapeutic blocks of therapy are those extensively used by the major international study groups. Thus,the main aim of the ICiCLe ALL-14 study is to standardise care across the centres in India. Thus, this qualifies as a phase IV study.

To move forward from ICiCLe ALL-14 to its successor, we also need to understand how best to benefit our patient group. We have looked to decrease toxicity by decreasing therapy in low risk patients, using modern molecular tools (MRD). This kind of evaluation needs to be done in a randomised fashion. Thus with this definition, ICiCLe ALL-2014 can also be considered to be a phase IV study which is seeking to establish the best standard of care.

All patients on the ICiCle ALL-14 protocol will receive prednisolone 60mg/m^2/day during induction and dexamethasone, post induction. Though a number of studies have reported on the superior effects of dexamethasone (24, 25) it is more likely that these results were based on a prednisolone dose of 40mg/m^2(26). The BFM in a recent randomised trial showed that dexamethasone produced better event free survival rates when compared to prednisolone. However, the increased toxicity and low relapse salvage rate led to comparable overall survival and they continue to use prednisolone during induction except in T-cell ALL (27).

Steroid randomisation:

The use of 5 weeks of steroid is historical. There is both clinical and scientific evidence to suggest that pulsed interrupted steroids are as effective (28). In ALLR3, where 2 weekly pulses of high dose dexamethasone is used, we have not observed the degree of hyperglycaemia and obesity seen with continuous use of steroids or the high incidence of osteonecrosis seen in older patients (personal observations seen in frontline trials). Thus, for SR and IR patients under the age of 10 years, we are randomising the duration of steroids, decreasing the duration from 5 to 3 weeks in the test arm. **The primary aim** is to show that there is a decrease in toxicity in the low dose arm. We will also look for equivalence in **EFS, OS, CR** and **MRD** rates between both arms. We and others have observed an increase in toxicity in older patients who receive steroids over a prolonged period. Thus older patients, not in the HR arm, will all receive the shorter course of steroids.

Randomisation of Mitoxantrone with Daunorubicin:

NCI HR and other HR patients do better with the inclusion of an anthracycline during induction. Traditionally, based on early phase clinical data, this has been the use of four doses of Daunorubicin during induction and 3-4 doses of Doxorubicin during delayed intensification. These are not based on any scientific rationale, but practices adopted over time. The other anthracyclines, namely Epirubicin, Idarubicin and Mitoxantrone have been variously used in relapse protocols and occasionally in frontline studies. The BFM group investigated the use of four doses of Mitoxantrone in frontline patients using a complex randomisation involving intensification of other drugs(20). While not reported at that time, subsequent analyses suggests that those who received Mitoxantrone had a better outcome (29).In a subsequent randomised phase III trial in relapsed ALL we have shown the marked superiority with less toxicity of two doses of Mitoxatrone over Idarubicin(28). A recent report also showed that that Mitoxantrone can be safely used instead of Daunorubicin in frontline patients (30). Thus,in ICICLe ALL-14, we are randomisng the use of Mitoxantrone against the conventional 3 doses of Doxorubicin in delayed intensification, testing for both an improvement in outcome and a decrease in toxicity in the test arm. In our pilot studies, we have already observed less toxicity with a single dose of Mitoxantrone.

Thus, overall the study will (i) standardise treatment of childhood ALL across the 4 major metropolis's and beyond (ii) standardise diagnostic and MRD techniques (iii) establish a standard reference for future advancement (iv) demonstrate that a less intensive therapy can offer comparable survivals and (v) investigate the efficacy of Mitoxantrone in frontline protocols. Going forward then, we wish to establish a platform of therapy where we can optimise the duration of steroid therapy;establish a scientific basis for the use of anthracylines and improve outcome with decreased toxicity.

References

1. Marwaha RK, Kulkarni KP. Childhood Acute Lymphoblastic Leukemia: Need of a National Population Based Registry. Indian Pediatr. 2011 Oct;48(10):821-. PubMed PMID: ISI:000296471500020. English.

2. Arora RS, Eden TO, Kapoor G. Epidemiology of childhood cancer in India. Indian J Cancer. 2009 Oct-Dec;46(4):264-73. PubMed PMID: 19749456. Epub 2009/09/15. eng.

3. Kulkarni KP, Arora RS, Marwaha RK. Survival Outcome of Childhood Acute Lymphoblastic Leukemia in India: A Resource-Limited Perspective of More Than 40 Years. J Pediat Hematol Onc. 2011 Aug;33(6):475-9. PubMed PMID: ISI:000293171000028. English.

4. Schrappe M, Nachman J, Hunger S, Schmiegelow K, Conter V, Masera G, Pieters R, Pui CH. 'Educational symposium on long-term results of large prospective clinical trials for childhood acute lymphoblastic leukemia (1985-2000)'. Leukemia. 2010 Feb;24(2):253-4. PubMed PMID: ISI:000274397700001. English.

5. Bajel A, George B, Mathews V, Viswabandya A, Kavitha ML, Srivastava A, Chandy M. Treatment of children with acute lymphoblastic leukemia in India using a BFM protocol. Pediatr Blood Cancer. 2008 Nov;51(5):621-5. PubMed PMID: 18688848. Epub 2008/08/09. eng.

6. Magrath I, Shanta V, Advani S, Adde M, Arya LS, Banavali S, Bhargava M, Bhatia K, Gutierrez M, Liewehr D, Pai S, Sagar TG, Venzon D, Raina V. Treatment of acute lymphoblastic leukaemia in countries with limited resources; lessons from use of a single protocol in India over a twenty year period [corrected]. Eur J Cancer. 2005 Jul;41(11):1570-83. PubMed PMID: 16026693. Epub 2005/07/20. eng.

7. Carroll WL. Race and outcome in childhood acute lymphoblastic leukemia. Jama. 2003 Oct 15;290(15):2061-3. PubMed PMID: 14559962. Epub 2003/10/16. eng.

8. Siraj AK, Kamat S, Gutierrez MI, Banavali S, Timpson G, Sazawal S, Bhargava M, Advani S, Adde M, Magrath I, Bhatia K. Frequencies of the major subgroups of precursor B-cell acute lymphoblastic leukemia in Indian children differ from the West. Leukemia. 2003 Jun;17(6):1192-3. PubMed PMID: 12764391. Epub 2003/05/24. eng.

9. Amare P, Gladstone B, Varghese C, Pai S, Advani S. Clinical significance of cytogenetic findings at diagnosis and in remission in childhood and adult acute lymphoblastic leukemia: experience from India. Cancer Genet Cytogenet. 1999 Apr;110(1):44-53. PubMed PMID: 10198622. Epub 1999/04/13. eng.

10. Gutierrez MI, Timson G, Siraj AK, Bu R, Barbhaya S, Banavali S, Bhatia K. Single monochrome real-time RT-PCR assay for identification, quantification, and breakpoint cluster region determination of t(9;22) transcripts. J Mol Diagn. 2005 Feb;7(1):40-7. PubMed PMID: 15681473. Pubmed Central PMCID: 1867499. Epub 2005/02/01. eng.

11. Pastore G, Viscomi S, Gerov GL, Terracini B, Madon E, Magnani C. Population-based survival after childhood lymphoblastic leukaemia in time periods corresponding to specific clinical trials from 1979 to 1998 - a report from the Childhood Cancer Registry of Piedmont (Italy). European Journal of Cancer. 2003 May;39(7):952-60. PubMed PMID: ISI:000182792200020. English.

12. Stiller CA, Kroll ME, Pritchard-Jones K. Population survival from childhood cancer in Britain during 1978-2005 by eras of entry to clinical trials. Ann Oncol. 2012 Sep;23(9):2464-9. PubMed PMID: ISI:000308017000038. English.

13. Yeoh AEJ, Ariffin H, Chai ELL, Kwok CSN, Chan YH, Ponnudurai K, Campana D, Tan PL, Chan MY, Kham SKY, Chong LA, Tan AM, Lin HP, Quah TC. Minimal Residual Disease-Guided Treatment Deintensification for Children With Acute Lymphoblastic Leukemia: Results From the Malaysia-Singapore Acute Lymphoblastic Leukemia 2003 Study. J Clin Oncol. 2012 Jul 1;30(19):2384-92. PubMed PMID: ISI:000306182300017. English.

14. Matloub Y, Bostrom BC, Hunger SP, Stork LC, Angiolillo A, Sather H, La M, Gastier-Foster JM, Heerema NA, Sailer S, Buckley PJ, Thomson B, Cole C, Nachman JB, Reaman G, Winick N, Carroll WL, Devidas M, Gaynon PS. Escalating intravenous methotrexate improves event-free survival in children with standard-risk acute lymphoblastic leukemia: a report from the Children's Oncology Group. Blood. 2011 Jul 14;118(2):243-51. PubMed PMID: ISI:000292735100008. English.

15. Kadan-Lottick NS, Ness KK, Bhatia S, Gurney JG. Survival variability by race and ethnicity in childhood acute lymphoblastic leukemia. J Am Med Assoc. 2003 Oct 15;290(15):2008-14. PubMed PMID: ISI:000185924200024. English.

16. Bhatia S, Sather HN, Heerema NA, Trigg ME, Gaynon PS, Robison LL. Racial and ethnic differences in survival of children with acute lymphoblastic leukemia. Blood. 2002 Sep 15;100(6):1957-64. PubMed PMID: ISI:000177884800003. English.

17. Pui CH, Sandlund JT, Pei DQ, Rivera GK, Howard SC, Ribeiro RC, Rubnitz JE, Razzouk BI, Hudson MM, Cheng C, Raimondi SC, Behm FG, Downing JR, Relling MV, Evans WE. Results of therapy for acute lymphoblastic leukemia in black and white children. J Am Med Assoc. 2003 Oct 15;290(15):2001-7. PubMed PMID: ISI:000185924200023. English.

18. Tanneberger S, Cavalli F, Pannuti F. Cancer in Developing Countries: The Great Challenge for Oncology in the 21st Century: Zuckschwerdt Verlag 2004.

19. Conter V, Bartram CR, Valsecchi MG, Schrauder A, Panzer-Grumayer R, Moricke A, Arico M, Zimmermann M, Mann G, De Rossi G, Stanulla M, Locatelli F, Basso G, Niggli F, Barisone E, Henze G, Ludwig WD, Haas OA, Cazzaniga G, Koehler R, Silvestri D, Bradtke J, Parasole R, Beier R, van Dongen JJM, Biondi A, Schrappe M. Molecular response to treatment redefines all prognostic factors in children and adolescents with B-cell precursor acute lymphoblastic leukemia: results in 3184 patients of the AIEOP-BFM ALL 2000 study. Blood. 2010 Apr 22;115(16):3206-14. PubMed PMID: ISI:000276956500006. English.

20. Reiter A, Schrappe M, Ludwig WD, Hiddemann W, Sauter S, Henze G, Zimmermann M, Lampert F, Havers W, Niethammer D, Odenwald E, Ritter J, Mann G, Welte K, Gadner H, Riehm H. Chemotherapy in 998 Unselected Childhood Acute Lymphoblastic-Leukemia Patients - Results and Conclusions of the Multicenter Trial All-Bfm-86. Blood. 1994 Nov 1;84(9):3122-33. PubMed PMID: ISI:A1994PP02100037. English.

21. Vora A, Goulden N, Wade R, Mitchell C, Hancock J, Hough R, Rowntree C, Richards S. Treatment reduction for children and young adults with low-risk acute lymphoblastic leukaemia defined by minimal residual disease (UKALL 2003): a randomised controlled trial. Lancet Oncol. 2013 Mar;14(3):199-209. PubMed PMID: 23395119. Epub 2013/02/12. eng.

22. Larsen EC, Salzer WL, Devidas M, Nachman JB, Raetz EA, Loh ML, Heerema NA, Carroll AJ, Gastier-Foster JM, Borowitz MJ, Wood BL, Willman CL, Winick NJ, Hunger S, L. CW. Comparison of high-dose methotrexate (HD-MTX) with Capizzi methotrexate plus asparaginase (C-MTX/ASNase) in children and young adults with high-risk acute lymphoblastic leukemia (HR-ALL): A report from the Children’s Oncology Group Study AALL0232. J Clin Oncol 29 (suppl; abstr 3). 2011.

23. Conter V, Valsecchi MG, Silvestri D, Campbell M, Dibar E, Magyarosy E, Gadner H, Stary J, Benoit Y, Zimmermann M, Reiter A, Riehm H, Masera G, Schrappe M. Pulses of vincristine and dexamethasone in addition to intensive chemotherapy for children with intermediate-risk acute lymphoblastic leukaemia: a multicentre randomised trial. Lancet. 2007 Jan 13;369(9556):123-31. PubMed PMID: ISI:000243538200031. English.

24. Mitchell CD, Richards SM, Kinsey SE, Lilleyman J, Vora A, Eden TOB, Leukaemi MRCC. Benefit of dexamethasone compared with prednisolone for childhood acute lymphoblastic leukaemia: results of the UK Medical Research Council ALL97 randomized trial. Brit J Haematol. 2005 Jun;129(6):734-45. PubMed PMID: ISI:000229635300004. English.

25. Bostrom BC, Sensel MR, Sather HN, Gaynon PS, La MK, Johnston K, Erdmann GR, Gold S, Heerema NA, Hutchinson RJ, Provisor AJ, Trigg ME. Dexamethasone versus prednisone and daily oral versus weekly intravenous mercaptopurine for patients with standard-risk acute lymphoblastic leukemia: a report from the Children's Cancer Group. Blood. 2003 May 15;101(10):3809-17. PubMed PMID: 12531809. Epub 2003/01/18. eng.

26. Teuffel O, Kuster SP, Hunger SP, Conter V, Hitzler J, Ethier MC, Shah PS, Beyene J, Sung L. Dexamethasone versus prednisone for induction therapy in childhood acute lymphoblastic leukemia: a systematic review and meta-analysis. Leukemia. 2011 Aug;25(8):1232-8. PubMed PMID: 21527934. Epub 2011/04/30. eng.

27. Schrappe M, Zimmermann M, Moricke A, Mann G, Valsecchi MG, Bartram CR, Biondi A, Panzer-Grumayer R, Schrauder A, Locatelli F, Reiter A, Basso G, Niggli F, Aricoo M, Conter V. Dexamethasone in Induction Can Eliminate One Third of All Relapses in Childhood Acute Lymphoblastic Leukemia (ALL): Results of An International Randomized Trial in 3655 Patients (Trial AIEOP-BFM ALL 2000). Blood. 2008 Nov 16;112(11):9-. PubMed PMID: ISI:000262104700008. English.

28. Parker C, Waters R, Leighton C, Hancock J, Sutton R, Moorman AV, Ancliff P, Morgan M, Masurekar A, Goulden N, Green N, Revesz T, Darbyshire P, Love S, Saha V. Effect of mitoxantrone on outcome of children with first relapse of acute lymphoblastic leukaemia (ALL R3): an open-label randomised trial. Lancet. 2010 Dec 11;376(9757):2009-17. PubMed PMID: ISI:000285807700030. English.

29. Schrappe M. Mitoxantrone in first-relapse paediatric ALL: the ALL R3 trial. Lancet. 2010 Dec 11;376(9757):1968-70. PubMed PMID: 21131040.

30. Nickel RS, Keller F, Bergsagel J, Cooper T, Daves M, Sabnis H, Lew G. Mitoxantrone as a substitute for daunorubicin during induction in newly diagnosed lymphoblastic leukemia and lymphoma. Pediatr Blood Cancer. 2013 Dec 19. PubMed PMID: 24357218.

Eligibility Criteria

Inclusion Criteria for upfront randomization:

- Age ≥1 year and ≤18 years at time of enrollment
- Previously untreated (see definition below)
- Diagnosis confirmed by morphology and flow-cytometry
- T-cell Lymphoblastic Lymphoma confirmed by cytology or histology

Exclusion Criteria:

- Previously treated patients who do not meet criteria (see below)
- ALL-L3 (mature B-cell ALL)
- Down Syndrome
- 'Mixed phenotype acute leukaemia' (MPAL)

BCP ALL

Standard Risk (SR)

- Age >1 and <10 years
- Non T-cell
- Prednisolone Good Responder
- No high risk cytogenetics
- WBC <50,000/mm^3
- MRD <10^{-4} after induction
- Complete Remission after induction
- No CNS disease

Intermediate Risk (IR)

- Good risk features but age ≥10 years
- Good risk features but WBC ≥50,000/mm^3
- Good risk features but bulky lymph nodes (≥5 cm in peripheral region and in chest >5 cm on CT scan or occupying ≥1/3rd diameter on chest x-ray) and/or bulky liver/spleen reaching to the umbilicus or beyond and/or presence of testicular disease
- MRD <10^{-4} after induction
- No High Risk criteria

High Risk (HR)

- All prednisolone poor responders, irrespective of age and presenting WBC count
- High risk cytogenetics
- CNS disease (as defined below)

- MRD ≥10^{-4} after induction
- Incomplete laboratory information required for risk stratification
- Patients with atypical clinical features that preclude satisfactory risk stratification

T Cell ALL

All T- Cell ALL are treated uniformly.

Cytogenetics

Details of cytogenetic analyses required are given in **Appendix 8**. The following cytogenetic subtypes are classified as high risk:

- *MLL* rearranged leukaemias, e.g. t(4;11) (q21;q23)
- Low hypodiploidy (<40 chromosomes)
- t(17;19) (q22;p13) (*TCF3-HLF*)
- Intrachromosomal amplification of chromosome 21 (iAMP21)
- t(9;22) (q34;q11) (BCR-ABL)*

***All Philadelphia positive patients will receive Imatinib 360mg/m^{2}continuously from Induction to end of therapy in addition to high risk chemotherapy.**

Central Nervous System (CNS) disease

Includes patients with clinical signs of CNS disease (e.g. cranial nerve palsy) and/or CSF pleocytosis (≥ 5 cells/microliter) with detectable blasts on cytospin preparations of cerebrospinal fluid (CSF, see Appendix 12). These patients are categorised as High Risk.

CSF assessment is performed on Treatment Day 8, after completion of the Steroid prophase. In select cases, CSF assessment may be delayed for logistical reasons (permissible up to 2 days, i.e. to Treatment Day 10) prior to commencement of intensive multiagent therapy. In other cases, intensive multiagent therapy may be introduced ahead of scheduled CSF assessment on Treatment Day 8. In both situations, results of CSF assessment will be deemed valid for risk stratification on the Trial. Patients in the latter group may be analysed separately to evaluate potential impact of incomplete CNS risk assignment.

In situations where CSF assessment is delayed ≥ 15 days , patients will be categorised as 'CNS not evaluable' and these patients will be excluded from enrolment on the Trial.

Definition of CNS disease

CNS disease will be diagnosed in the following patients:

(a) Patients with unequivocal signs of CNS disease at presentation (CNS 3b), e.g. cranial nerve palsies

(b) Patients with any detectable blast in cerebrospinal fluid (CSF) cytospin on an atraumatic CSF sample at Induction Day 8

For patients with traumatic CSF samples (blood-stained CSF and/or CSF RBC count ≥ 10/μL) and detectable blasts on CSF cytospin at Induction Day 8, CSF examination will be repeated at Induction Day 15 together with the scheduled Intrathecal Methotrexate instillation

- If Induction Day 15 CSF cytospin shows no blast, these patients will be designated as "Traumatic lumbar puncture with blasts" (CNS 2b/CNS 2c) and will receive an additional dose of Intrathecal Methotrexate on Day 22.

If Induction Day 15 CSF cytospin shows blasts, these patients will be designated as CNS disease and will be treated accordingly.
(Also see Appendix 6: Recommendations for Cerebrospinal Fluid analysis [Page no. 150])

Prednisolone Response

- **Good**: <1000 blasts/l in peripheral blood after 1 week of prophase
- **Poor**: Presence of ≥1000 blasts/l in peripheral blood after 1 week of prophase

Measured as a percentage of blasts of the absolute leukocyte count.

Those who do not have blasts in the peripheral smear at diagnosis, will be treated as good/intermediate risk, **UNLESS**

1. Other features suggest that they should be treated as high risk
2. Peripheral blast count on day 8 is ≥1000/mm^3

Prednisolone response abrogated

Prednisolone response will be considered 'abrogated' in the following situations:

(a) Introduction of multi-agent therapy during the steroid prophase
This may be required in situations where rapid disease progression is observed (e.g. rapidly rising WBC count) and/or where rapid cytoreduction is necessary (such as in mediastinal tumours associated with the superior mediastinal syndrome)

(b) Persistent significantly enlarged organs and/or nodal masses despite clearance of circulating blasts following the steroid prophase

All patients with 'abrogated prednisolone response' will be designated 'High Risk'

Enrolment

Patients are to be enrolled and randomised when appropriate, after day 8 assessment.

Inclusion and Exclusion criteria for partially treated patients

Patients who have already commenced treatment elsewhere are not eligible, especially if a proper diagnosis and risk stratification cannot be made. Where this is still possible, the following exceptions are allowed:

1. Those who have begun steroids only. Irrespective of steroid use or dose, these patients can be considered for enrolment and randomisation, provided they have not crossed the day 8 evaluation time point. Steroid evaluation will be one week from the start of steroid therapy.

2. Those who have received one-dose of vincristine can be enrolled, but cannot be classified as Prednisolone good response as we cannot be clear if this is due to the effect of the drug. Therefore rather than treat these patient as poor risk, they will not be eligible for the steroid randomisation but receive the full 5 weeks of therapy. Those in this category with a poor response at day 8 will be treated on the high risk arm. These patients will be analysed separately.

3. Those who have received one Intrathecal Methotrexate (with or without steroids), may also be enrolled and randomised, but they will be analysed separately to exclude variance, prior to inclusion in the main study cohort.

4. In patients who have received steroid monotherapy for a duration greater than 8 days, enrolment would still be possible if a day 8 peripheral blood smear is available for blast enumeration

5. Patients 1 – 18 years with newly-diagnosed acute lymphoblastic leukaemia, who have received no prior treatment and for whom risk stratification information is insufficient, will be categorised as High Risk and will be included in the trial. This includes patients with

(i) incomplete laboratory information required for risk stratification (cytogenetics, CSF studies, day 8 prednisolone response, MRD analysis); and
(ii) patients with atypical clinical features that preclude satisfactory risk stratification, such as patients with unusual sites of extramedullary disease

These patients will be treated on the protocol but will not be eligible for randomisation and their outcomes will be assessed separately.

Definition of complete remission (CR)

<5% blasts morphologically in the bone marrow aspirate; and absence of blasts in the CSF. This needs to be achieved by induction.

For patients with testicular disease, the testes need to return to normal shape and feel by the end of consolidation. When in doubt, this can be confirmed by ultrasonography.

Allocation of post-induction therapy in T-Lymphoblastic Lymphoma patients-

For patients with mediastinal enlargment, volumetric measurement of tumour mass should be made as soon after presentation preferably by CT scan. The same modality of imaging should then be used to calculate the reduction in tumour volume at day 35. It is important to recognise that tumour regression is frequently incomplete. PET scanning is unproven in LBL and not currently recommended for stratification of treatment.

Patients with <35% reduction in tumour volume are considered poor responders and may be offered off-protocol treatment if their disease was greater than stage II. Those ineligible for aggressive/experimental therapies and patients with ≥ 35% reduction in tumour volume (good responders) are continued on high-risk treatment.

Patients not in CR by the end of consolidation will be considered to be withdrawn.

Criteria for removal of patients from Study Protocol

1. Failure to achieve remission at the end of the Consolidation phase, including *any* of the following

   (a) Persistent clinically significant disease post-consolidation
   (b) Persistent excess blasts (> 5%) on bone marrow microscopy (centrally reviewed)
   (c) Persistent high MRD ('MRD positivity') in T-cell disease

Note: Persistent blasts in the CSF cytospin or persistent abnormally palpable testis at the end of induction do not constitute criteria for removal of patients from the study protocol. These patients will receive regional radiotherapy (see section on Radiotherapy, **Appendix 13**)

2. Necessity for permanent omission of three or more chemotherapy drugs from the treatment protocol due to major dose-limiting toxicity (e.g. L-asparaginase due to prior pancreatitis, anthracycline due to cardiomyopathy and vincristine due to irreversible foot drop)

Laboratory Investigations and Lumbar Punctures

Assessment of peripheral blood blast clearance at Day 8

- Determined after count of 200 white blood cells on peripheral blood
- On non-working days, a stained peripheral blood smear would be prepared and retained for later review

Bone Marrow Studies

- All patients will undergo *at least* two bone marrow examinations (at diagnosis and at end-of-induction)
- A third marrow examination at the end of consolidation is recommended but not mandatory, to confirm MRD clearance.
- Patients with precursor-T ALL will undergo three bone examinations (at diagnosis, at end-of-induction and at week 11)
- Patients with suboptimal marrow studies at end-of-induction will require repeat marrow studies, 1-2 weeks later before continuation of treatment.
- Patients who fail to achieve morphological remission at end-of-induction will undergo a repeat bone marrow examination at the end of consolidation (high-risk consolidation therapy)

Cerebrospinal fluid studies (Day 8, diagnostic)

- Processed within 2 hours of collection as blast viability rapidly declines beyond 2 hours

Marrow assessment at end of Induction (Day 35)

- Await count recovery before performing the end-of-induction marrow study
- Report should include comment on marrow cellularity
- First sample draw should be sent for flow cytometry MRD, subsequent samples for morphology, cytogenetics, cell storage, etc.
- Laboratory studies
- Flow cytometry MRD
- Uniform testing practice required, including instruments and reagents (especially antibodies)
- External Quality Assurance schemes required to ensure/monitor uniformity in testing and reporting

Testing centres

- Regional centres with expertise in flow cytometry MRD
- Includes TMH Mumbai, PGIMER Chandigarh, AIIMS Delhi, CI (WIA) Chennai, TMC Kolkata

Cytogenetics

1. Minimum testing
   Interphase fluorescent in situ hybridisation (FISH) analysis of bone marrow cells
   B-cell precursor ALL: Testing using three FISH probes
   a) ETV6-RUNX1 dual-colour dual-fusion FISH probe
      (i) Diagnosis of ETV6-RUNX1 BCP-ALL
      (ii) Diagnosis of iAMP21 BCP-ALL (three or more copies of RUNX1,in tandem)
      (iii) Suspicion of Hyperdiploidy (extra RUNX1 copies)
   b) BCR-ABL1 dual-colour dual-fusion FISH probe
      Diagnosis of BCR-ABL1 ALL (Ph+ ALL)
   c) KMT2A (MLL) break-apart probe
      Diagnosis of KMT2A (MLL)-rearranged ALL
   T-ALL: Testing using two FISH probes
   a) BCR-ABL1 dual-colour dual-fusion FISH probe
      Diagnosis of BCR-ABL1 ALL (Ph+ ALL)

   b) KMT2A (MLL) break-apart probe
      Diagnosis of KMT2A (MLL)-rearranged ALL
2. Complementary studies
   Identification of aneuploidy in ALL blasts using either
   a) GTG-banded karyotyping; or
   b) DNA ploidy by flow cytometry using the FxCycle Violet dye (incorporated in routine immunophenotyping analysis at diagnosis)
3. Extended testing (in select cases, discretionary)
   a) FISH testing for TCF3 rearrangement, in cases where 3-probe FISH testing in BCP-ALL is not informative
   b) FISH testing using the ABL1 break-apart probe to investigate ABL-class fusion ALLs, in cases where extra ABL1 signals are observed
   c) FISH testing for ABL-class fusion ALL in cases of remission failure (bone marrow blasts ≥ 5%) and/or high MRD (≥ 1%) at end of Induction. FISH testing here will include use of break-apart probes to detect rearrangements of ABL1, ABL2 and PDGFR-beta(also PDGFR-alpha in T-ALL)
   d) FISH testing using chromosome enumeration probes for chromosomes 4, 10 and 17 to confirm Hidden Hyperdiploidy, in cases where karyotyping has failed and/or DNA flow ploidy is not informative. Alternatively, FISH testing for Hidden Hyperdiploidy can include use of break-apart probes for CRLF2 (Xp22.33) and IgH (14q32.33), which testing will allow additionalcharacterisation of the B-Others subtype through investigation of CRLF2 and IgH rearrangements
4. Not recommended
   Use of end-point reverse-transcription PCR for fusion transcripts alone as cytogenetic risk stratification. This strategy will not detect aneuploidies and will miss non-AF4KMT2A (MLL) fusions. Additionally, the technique is less sensitive in detecting ETV6-RUNX1 fusion and tends to overestimate BCR-ABL1 fusion.

Minimal Residual Disease (MRD) estimation

1. Testing
   a) Estimated by flow cytometry analysis in B-cell precursor ALL alone
   b) MRD testing is currently not performed in T-ALL but is likely to be introduced soon
2. Timing
   On Day 35, following completion of the Induction phase
   Estimation of minimal residual disease (MRD) in the bone marrow is

   (i) *mandatory* at the end of the induction phase; and
   (ii) *recommended* at the end of the consolidation phase

3. Sample
    a) Bone marrow sample, first draw of 0.5mL marrow to be placed in an EDTA tube for MRD analysis
    b) Using the same pass, the bone marrow needle will be angled to aspirate an additional 0.5mL marrow from a differentregion of the marrow. This is performed to decrease sampling errors in cases of patchy residual marrow disease post-treatment. This sample will be pooled with the earlier 0.5mL draw and submitted for MRD analysis.
    c) Subsequent marrow samples will be submitted for microscopy analysis to assess post-treatment morphological remission
4. Analysis
    a) Cell suspension prepared by bulk RBC lysis, followed by washing, enumeration and staining with antibody cocktail
    b) Analysis of a minimum 1 million events using an 8-colour or a 10-colour antibody panel, including 6 B-cell differentiation markers as backbone (CD34, CD19, CD10, CD20, CD45, CD38) and 2 to 4 discriminatory (leukaemia-associated) markers (CD58, CD123, CD73, CD81). Nucleated cell count performed in parallel using a cell-permeable fluorochrome included in the MRD tubes
    c) Analysis performed using the Kaluza programme (Beckman Coulter). Determination of MRD events include observation of 'different from normal' and 'leukaemia-associated' features. In 'different from normal' approach, analysis performed using expression patterns observed in non-leukaemia bone marrow samples as template reference
    d) MRD calculated as percentage of MRD events in nucleated cells.
5. Interpretation

    High MRD — MRD ≥ 0.01%

    Negative MRD — MRD < 0.01%
6. Repeat testing is performed in the following situations
    a) Insufficient events (< 1 million) for robust MRD analysis
    b) Discordance between bone marrow morphology and MRD findings, where bone marrow indicates non-remission (blasts ≥ 5%) and MRD is negative. This could be on account of patchy marrow disease. In these cases, the recommendation is to repeat marrow testing for morphology and MRD studies, performing sampling from bilateral posterior superior iliac spines. Morphology will be assessed by studying bone marrow aspirates and biopsies. Samples from bilateral sites will be pooled and analysed for MRD.
7. Follow-up testing

    In cases of high MRD at end of the Induction phase, MRD analysis of bone marrow repeated at the end of the Consolidation phase to confirm MRD clearance

Treatment Delays and Dosing Adjustments

Steroid prophase

Equivalent doses of intravenous Dexamethasone (10mg Pred : 1 mg Dex) are permissible during the steroid prophase

Interruption of therapy

During **Induction, Consolidation** and **Delayed Intensification** phases: Catch-up of treatment is warranted

During the **Interim Maintenance** and **Maintenance** phases: No catch-up is required. Treatment elements not administered during the period of interruption are considered '**burnt**'

Prednisolone taper with continuous therapy; taper over five (5) days

In cases of indeterminate CNS status (e.g. CSF study complicated by traumatic tap with detectable blasts OR in cases where no CSF is available for analysis), an additional intrathecal MTX is administered during Induction (i.e. four [4] IT MTX doses in all)

Routine use of growth factors is not recommended; used at the discretion of the treating Physician

Maintenance phase: Twelve (12) weeks of 6-MP and MTX, 1 dose of intrathecal MTX every cycle, 96 treatment weeks in all.

The start dose for 6-MP during the maintenance phase is 60mg/m^2. Experience during the interim maintenance phase would serve as an indicator of tolerance to 6-MP.

Targeted neutrophil count (ANC) during antimetabolite therapy: 750– 1500 neutrophils/mm^3

Intermediate risk ALL

Induction phase

Two (2) doses of Daunorubicin, at least 1 week apart, but timing may be altered depending on clinical context

Interim maintenance

Intravenous methotrexate, (without L-asparaginase) starting at 100mg/m^{2}and escalated by 50mg/m^2

High risk ALL

Patients with non-remission marrow morphology at end of induction (marrow status M2 or M3) will continue receiving treatment on the High Risk arm. Marrow status will be reassessed at end of Augmented BFM Ib consolidation. Failure of morphological remission at end of consolidation indicates poor-risk disease and patients will be treated off-study with intent to provide allogeneic stem cell transplantation if feasible

Radiotherapy

All patients CNS 3 on day 8 will be treated as HR and receive cranial irradiation (18Gy) during maintenance therapy.

All those with persistently abnormal testes post consolidation will receive irradiation to both testes (24Gy) during maintenance.

BCR-ABL1 disease

Imatinib 360 mg/m^2will commence as soon as feasible and continued through until end of therapy. Cytopenias are anticipated with TKI during the maintenance phase, and may require treatment interruption followed by sequential reintroduction of TKI and antimetabolites, dose adjustments of all drugs maybe required.

Monitoring of response by prednisolone response and flow cytometry MRD. Serial monitoring of BCR-ABL transcript will be a secondary response indicator.

Chemotherapy Protocol

The overall design of the ICICLE ALL-14 study is shown in figures 1 and 2.

Figure 1: Treatment Schema for progenitor-B phenotype

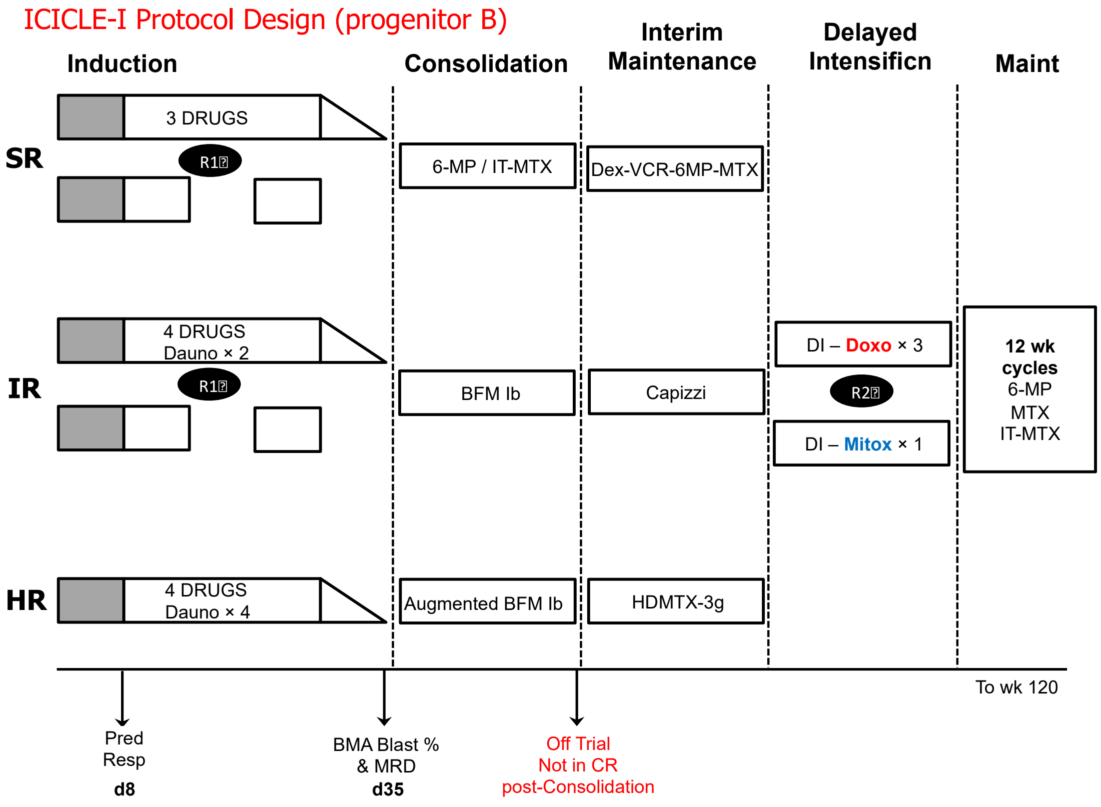

All patients will receive 7 days of prednisolone at 60mg/m^2 for 7 days. On day 8, they will be risk stratified to Standard, Intermediate and High risks.

Figure 1: Treatment Schema for T-phenotype

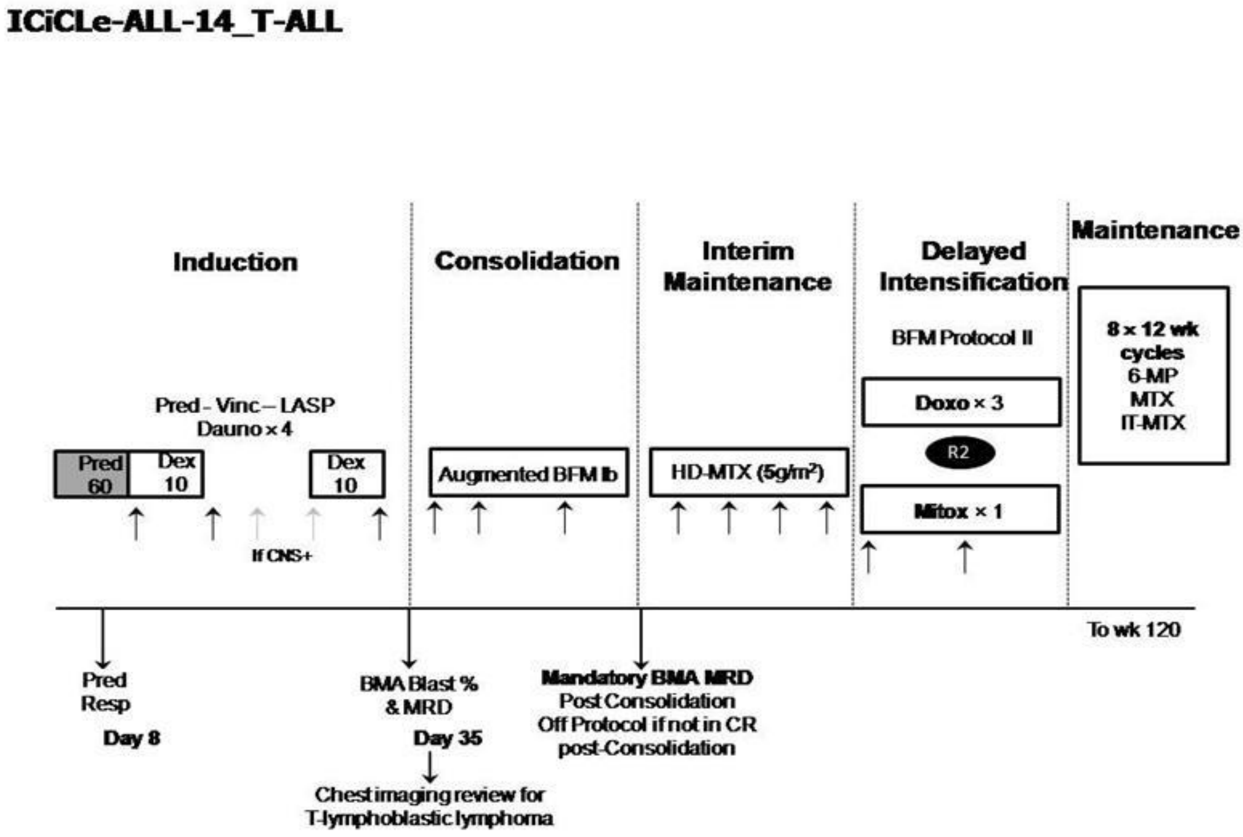

All patients will receive 7 days of prednisolone at 60mg/m^2 for 7 days.

BCP-ALL

Standard Risk (SR)

Age ≥1 year and <10 years old at diagnosis AND presenting white blood cell count (WC) before starting treatment is <50,000/mm^{3}ANDprednisolone good responder. No high risk cytogenetics. MRD <10^{-3} after induction. CR after induction. No CNS disease.

Intermediate Risk (IR)

Age ≥10 years old at diagnosis OR presenting WC before starting treatment is ≥50,000/mm^{3}or bulky/testicular disease or non bulky disease, ANDprednisolone good response.No High risk cytogenetics.

High Risk (HR)

Those with high risk cytogenetics OR CNS disease OR aprednisolone poor response or MRD≥10^{-3} after inductionOR no CR at the end of induction.

BCP-ALL

SR and IR (<10 years) patients will be randomised to receive either 5 weeks or 3 weeks of steroids. All IR patients ≥ 10 years will receive 3 weeks of steroids. In induction, SR patients will receive 3 drugs, IR 4 drugs with decreased use of anthracyclines and HR full intensity treatment. SR patients will receive a low risk consolidation followed by interim maintenance. IR will receive BFM consolidation followed by interim maintenance with Intravenous methotrexate (without L-asparaginase). HR patients will receive augmented BFM followed by interim maintenance with high dose Methotrexate.

IR and SR progenitor B-cell patients with a MRD $\geq 10^{-4}$ at the end of induction will continue treatment on the high risk arm. All patients are eligible for a second randomisation during the delayed intensification phase. This will be between Mitoxantrone and Doxorubicin. All patients will receive 96 weeks of continuation therapy with daily 6-Mercaptopurine, weekly Methotrexate and Intrathecal Methotrexate every 3 months.

T-ALL

All T-cell patients will receive induction with pulsed steroids (dexamethasone). All patients will receive 4 doses of anthracycline. Post augmented consolidation all patients will receive high dose methotrexate and delayed intensification. MRD will be assessed for T-cell patients at the end of consolidation. T-cell patients will not participate in the first randomisation for steroids but are eligible for the second randomisation.

Aim

Primary

(i) Standardise therapy for children with Acute Lymphoblastic Leukaemia across multiple institutions in India.
(ii) Improve outcome for children with Acute Lymphoblastic Leukaemia in India

Secondary

(i) Institute a collaborative group that will develop current and future protocols, appropriate for Indian patients to achieve outcome rates comparable to other countries.

Objectives

Non-Randomised

(i) To improve outcome and decrease toxicity by using a risk stratified approach
(ii) Develop the use of minimal residual disease as a predictive biomarker for risk stratification

Randomised

(i) To reduce toxicity and improve outcome in low-risk patients through introduction of a short 3 week course of steroids instead of 5 weeks during induction.
(ii) To improve outcome and decrease toxicity by replacing Doxorubicin with Mitoxantrone

Primary outcome measures

Non-Randomised

Event free and overall survival in the whole cohort and the risk stratified groups

Randomised

*1. **First Randomisation (R1)***
Steroid induced morbidity defined as all serious adverse events related to induction and categorised as steroid related and steroid contributory, including delayed steroid toxicity.

*2. **Second Randomisation (R2)***
Event free (EFS2) and overall survival in the test arm.

Secondary outcome measures

*1. **Steroid Randomisation (R1)***
Rate of remission, MRD levels, event free (EFS1) and overall survival.

*2. **Anthracycline Randomisation (R2)***
Anthracycline induced morbidity defined as all serious adverse events related to anthracycline toxicity during delayed intensification and subsequently
Clinical outcomes will be measured using internationally accepted definitions of relapse and

treatment-related mortality (TRM), and **NCI CTCAE** toxicity grades (see **Appendix** for **CTCAE** version 4.0). For the R1, induced morbidity in induction will be assessed as all **Serious Adverse Events (SAEs)** within 5 weeks of the start of induction and any SAE's within 8weeks of the start of induction and classified as induction related by the Chief Investigator. Two broad categories of SAE will be measured; steroid related and steroid contributory. Steroid related SAE refers to any events known to be directly caused by exposure to high dose glucocorticoids namely diabetes, psychosis, hypertension, obesity and pathological osteopenia or fracture. Steroid contributory refers to those in which exposure to steroid is a significant contributory factor namely severe bacterial, fungal or viral infection, pancreatitis, thrombosis and encephalopathy. All avascular necrosis events post the 8 weeks will also be recorded.

For R2, morbidity will include all Serious Adverse Events (SAEs) within 5 weeks of the start of Delayed Intensification and any SAE's within 8 weeks of the start of Delayed Intensification and classified as related to this phase by the Chief Investigator. Primarily these will include infections and duration of myelosupression.

Study Design and Sample Size

ICiCle ALL-14 is a multicentre, phase III/IV, randomised controlled study. We aim to recruit approximately 2200 patients in 4-years. Induction follows an open label 2-arm randomized design (R1). The primary endpoint is steroid related toxicity and the primary comparison is steroid related toxicity of arm R1-A (5 weeks of prednisolone + other drugs) versus steroid related toxicity of arm R1-B (3 weeks of prednisolone + other drugs). Thus the study is able to detect a minimum 3% difference in toxicities between the two arms at a power of 80% and a p-value of 0.05 for the first randomization. Within the open label randomization R2, the prognostic effect of 3 doses of Doxorubicin versus 1 dose of Mitoxantrone combined with the conventional intensive consolidation therapy of the two arms is investigated. It is expected that the effect of 3 doses of Doxorubicin versus 1 dose of Mitoxantrone will be independent from the respective treatment arms, but randomization will be stratified by the induction arm to provide balanced groups. The expected EFS2 after 1st randomization in both arms is 70% at 4 years. The hypothesis of the study is that Mitoxantrone compared to Doxorubicin can improve EFS2. The study is able to detect a minimum 5% difference in EFS between the two arms at a power of 80% and a p-value of 0.05 for the second randomization.

Minimum Information prior to initiation of therapy

A. Patient details

1. Date of Birth
2. History of Prior Treatment (if any)
3. Height and weight
4. Highest WBC
5. Size of Liver, Spleen and Lymph Nodes
6. Serum LDH
7. Mediastinal Mass
8. Extramedullary Disease
9. Immunophenotype using eight colour panel
10. Bone Marrow studies

B. Post Prophase, Day 8 Assessment

1. Prednisolone Response (Absolute Blast Count)
2. CNS Disease
3. Cytogenetics
   a. Triple test FISH panel (BCR-ABL, MLL Rearrangement, ETV6-RUNX1)
   b. Karyotyping

C. Post Induction Bone Marrow Assessment

1. Bone Marrow microscopy
2. MRD Status
3. CNS Status (if previously CNS disease)

D. Post Consolidation Assessment

1. Testicular Disease (if Testicular disease at diagnosis)
2. Mediastinal Mass (if Mediastinal Mass at diagnosis)
3. Bone Marrow morphology (if patient is not in Complete Remission post Induction)

Dose Calculations and Adjustments for BMI

Surface Area Calculation (Mosteller)

SQRT ((cm*kg)/3600)

All children should be weighed in light weight clothing and scales should be calibrated regularly. To ensure that children are treated effectively, without overdosing due to treatment related fat deposition, the Body Mass Index (BMI) should be checked at diagnosis, prior to interim maintenance and at the beginning of each cycle in maintenance. Doses have traditionally been based on total weight rather than lean weight, so to change to lean body weight dosing could result in under-treatment.

Calculate using the following formula:

BMI= weight (kg) / height^2 (m)

The BMI can then be compared using the 2015 revised growth charts from the Indian Academy of Paediatrics (Khadilkar VV, Khadilkar AV. Indian J Endocr Metab 2015;19:470-476) .For children with a BMI that falls within the 2nd-98th percentiles, dose by actual weight using the BSA. These weights should be taken:

1) At diagnosis
2) Prior to interim maintenance
3) At the beginning of each maintenance cycle

For children who have a BMI >98th percentile read off the BMI at 98th percentile for their age. Calculate the dosing weight using the formula:

Calculate the dosing weight using the formula:

Dosing weight (kg) = BMI at adult 27 BMI equivalent for age × Ht2 (in m2).For children <3rd percentile, repeat as above reading the BMI at the 3rd percentile for calculation

For children who have greater than 10% weight loss due to illness during treatment it may be necessary to reassess their BMI and dosing weight prior to recommencing treatment.

Doses should be rounded to the nearest half tablet for children taking tablets.

Rationale for frequency of weighing for determination of changes in drug dosage

A normal child aged 2-10 years gains only 2-6kg/year. This equates to an average of 1kg every 3months (maximum 1.5kg). If children are weighed fully clothed rather than in light weight clothing, the extra weight will be greater than the quarterly change in weight. Scales, even regularly calibrated, also carry a measurement error. These errors, coupled with weight variation due to physical, endocrine, nutritional, and psycho-social effects make repeated measurements inaccurate.

Children having repeat doses of steroids will have greater than average weight gain, but this is primarily deposited as fat - particularly truncal fat. Unless drug treatment is with very lipophilic drugs the weight gain should not affect the therapeutic doses required to treat ALL. Only daunorubicin,doxorubicin and vincristine have longer T1/2 and larger volume of distribution,

indicating major transfer to body compartments other than the blood. Both anthracyclines are ionic compounds and they and their active metabolites are highly protein bound, which would suggest less deposit into fat than into lean mass. Vincristine is highly water soluble and readily distributed into tissues. As it is predominantly renally excreted it is again unlikely that it is highly deposited in fat.

Do not adjust doses on the basis of weight change any more frequently than every 3 months. In practice the changes should equate to:

1) At diagnosis
2) Prior to interim maintenance
3) At the beginning of each maintenance cycle

Induction

Treatment

1. General remarks

- **a.** Treatment should not be interrupted solely on account of myelosuppression
- **b.** Serious infection (e.g., varicella, neutropenic enterocolitis, invasive fungal infection) may require treatment interruption or scheduling modification. This will be based on the discretion of the attending physician. In all such situations, communication with the Study Coordinators is advised.

2. Tumour Lysis Prevention

- **a.** Especially important with bulky disease and/or high white blood counts
- **b.** Oral Allopurinol is to be given to all patients
  Commence at least 24 hours before start of cytotoxic therapy
  100mg/m^2/dose, three times a day, for at least 5 days
- **c.** Intravenous fluids for all patients
  For at least 48 hours (**non-potassium containing fluids**, 0.45% to 0.9% Saline at 125mL/m^2/hour)
- **d.** Monitor urine output
  Administer loop diuretic (0.25 – 1mg/kg Furosemide [max 40mg]) only if required
- **e.** Laboratory monitoring
  Based on clinical context; urea, creatinine, electrolytes, calcium, phosphate and uric acid

3. Routine antimicrobial prophylaxis is not recommended. **Antifungals therapy is recommended during intense treatment phases, particularly during IR/HR induction and Delayed Intensification.**The therapy used is according to institutional guidelines.Please note azoles interfere with vincristine metabolism and should preferably be avoided during the induction phase.

B-cell Precursor ALL Standard Risk

Age >1 and <10 years

AND

WBC <50,000/mm^3

AND

Prednisolone Good Responder

AND

No Testicular Disease or Bulky Disease
AND

No high risk cytogenetics

AND

MRD <10^{-4}at Day 35

Standard Risk – Summary Page

The Standard Risk (SR) regimen is for patients with the following features

- Age 1 to less than 10years
- Presentation white blood cell (WBC) count (< 50,000/mm^3)
- No high risk cytogenetic features
- Progenitor B-cell ALL
- No testicular disease or bulky disease

Randomisation elements in the Standard Risk regimen include

- *During the Induction Phase*
  5 weeks *versus* 3 weeks of Prednisolone
- *During the Intensification Phase*
  Doxorubicin (3 doses, Days 1, 8 and 15) *versus* Mitoxantrone (1 dose, Day 1)

Phase	Drugs	Randomisation	Days of therapy	Weeks of Therapy	MRD	Intrathecal Injections
**Induction**	Prednisolone,Vincristine, IT MTX, Asparaginase (Native or PEG)	5 weeks (R1A)*vs* 3 (R1B) weeks of Prednisolone	1-35	1-5	Day 35	3(plus 1 if traumatic tap)
**Consolidation**	ITMTX, 6MP	N/A	1-21	6-8	N/A	2
**Interim Maintenance**	Dexamethasone, Vincristine, IT MTX, Oral MTX, 6MP	N/A	1-63	9-17	N/A	2
**Delayed Intensification**	Pulsed Dexamethasone, Vincristine, IT MTX, Asparaginase (Native or PEG),Cyclophosphamide, Cytarabine, 6MP,Doxorubicin (R2A) or Mitoxantrone (R2B)	3 doses of Doxorubicin*vs* 1 dose of Mitoxantrone	1-49	18-24	N/A	2
**Maintenance**	IT MTX,Oral MTX and 6MP	N/A	1-84	8cycles,12 weeks each	N/A	1

Standard Risk, Induction Phase

SR-R1A

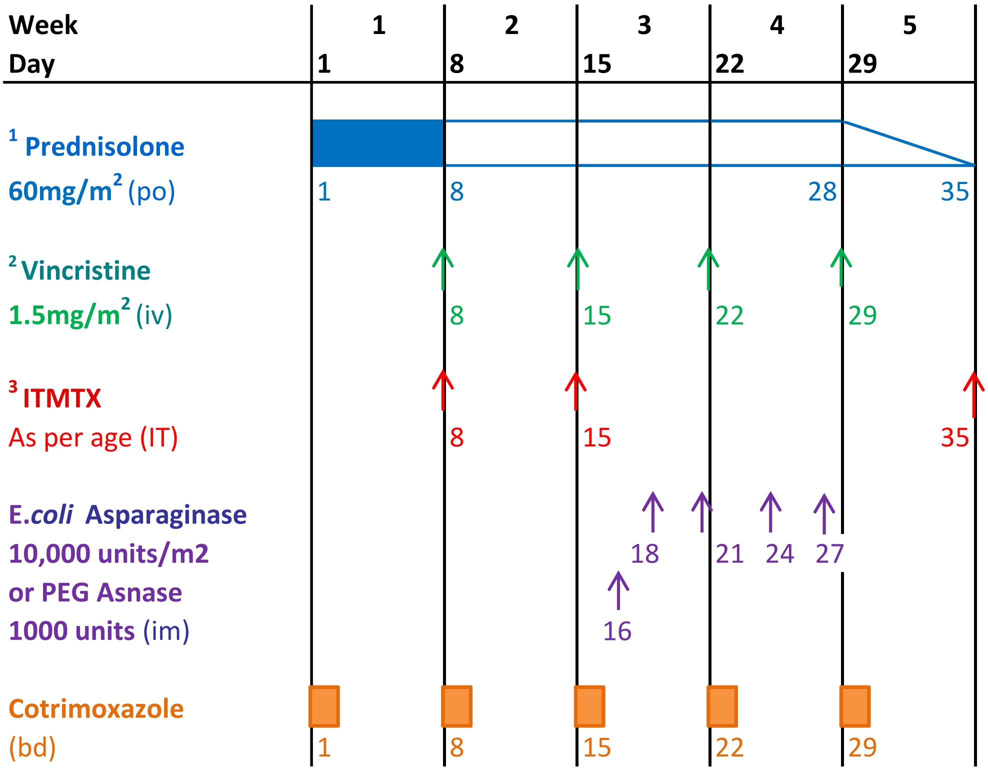

[1] **Prednisolone:** given in three divided doses

[2] **Vincristine:** maximum single dose of Vincristine is 2mg

[3] **Intrathecal Methotrexate:** <2 years= 8mg; 2-Less than 3 years= 10mg; ≥3 years= 12mg

Standard Risk, Induction Phase

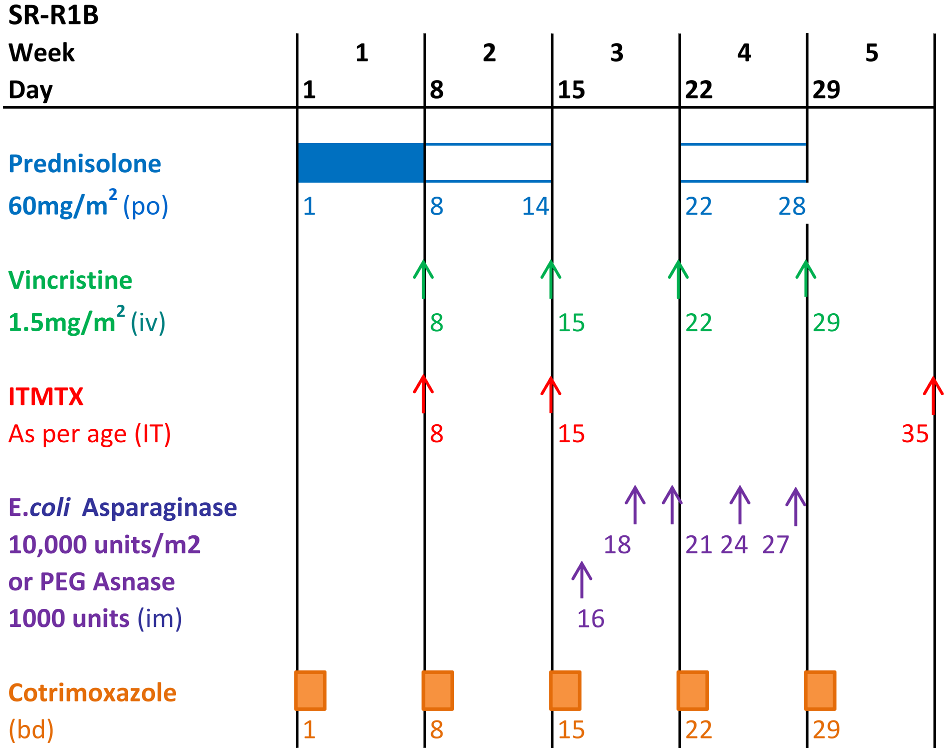

[1] **Prednisolone:** given in three divided doses

[2] **Vincristine:** maximum single dose of Vincristine is 2mg

[3] **Intrathecal Methotrexate:** <2 years= 8mg; 2-Less than 3 years= 10mg; ≥3 years= 12mg

Standard Risk (SR) Induction Phase

***Duration of therapy**: Days 1 – 35 *Inclusive* (Beginning of Week 1 – End of Week 5)

Drug Exposure

1. Prednisolone (PO)

Prophase

Day 1 to Day 7 (Common to both R1-A and R1-B)
Prednisolone 60mg/m^2 in three divided doses, **note** maximum permitted dose is 120 mg per day.

Please note: ***In patients with poorly responding or rising WBC count during the first 72 hours of the steroid prophase the treating physician may bring forward the introduction of additional cytotoxic therapy. In such cases the steroid prophase is terminated and the treatment schedule is as for multi agent therapy.***

Continuous dosing in SR Induction R1-A
Prednisolone 60mg/m^2 in three divided doses, **note** maximum permitted dose is 120 mg per day.
Continuous dosing, Days 8 – 28 (Beginning of Week 1 to End of Week 4)
Taper over 1 week, Days 29 – 35 (Week 5)

Intermittent dosing in SR Induction R1-B
Prednisolone 60mg/m^2 in three divided doses, note maximum permitted dose is 120 mg per day.
Intermittent dosing
Days 8 – 14 (Beginning of Week 1 to End of Week 2) & Days 22 – 28 (Week 4), No Taper

2. Vincristine (IV)
1.5 mg/m^2 (maximum, 2mg), slow intravenous push, weekly, for four (4) doses
Day 8 (Week 2), Day 15 (Week 3), Day 22 (Week 4), Day 29 (Week 5)

ALERT: PLEASE ENSURE THAT VINCRISTINE IS ADMINISTERED ONLY AFTER COMPLETION OF INTRATHECAL METHOTREXATE INJECTION. THIS IS TO AVOID INADVERTENT INTRATHECAL INJECTION OF VINCRISTINE.

3. L-Asparaginase (IM)
Where available, the PEGylated preparation, Pegaspargase (Oncaspar®) will be administered
Where Pegaspargase is not available, native E. coli L-asparaginase will be administered
Pegaspargase
One dose; 1000 Units/m^2, I.M., Days 16 (Week 3)
OR
Native E. *coli* L-asparaginase

Four doses; 10, 000 Units/m^2, I.M. Days 18, 21 (Week 3); 24, 27 (Week 4)

4. Intrathecal Methotrexate (IT)

Three doses- Day 8, Day 15, Day 35

Dose by age**,** Less than 2 years: 8mg; 2 – 3 years: 10mg; ≥ 3 years: 12mg

5. **Cotrimoxazole(PO)**

Twice a day, two consecutive days (e.g. Saturday and Sunday)

***Body Surface* Area (m^2)**	***Cotrimoxazole***	***Trimethoprim***	***Sulfamethoxazole***
0.50 – 0.75	240mg, BD	40mg, BD	200mg,BD
0.76 – 1.00	360mg, BD	60mg, BD	300mg, BD
>1.0	480mg, BD	80mg, BD	400mg, BD

Response assessment post induction

a. Prednisolone response at Day 8

Response is reliably assessed only after a minimum cumulative dose of 210 mg/m^2.

Estimation of peripheral blood blast count :
Prednisolone Good Response (PGR): Peripheral blood blast count<1000/µL)
Prednisolone Poor Response (PPR): Peripheral blood blast count ≥1000/µL)

b. Bone marrow test, Assessed at Day 35

<5% blasts = in remission
5-25% blasts = M2 marrow
>25% blasts = M3 marrow

c. Samples are to be sent for MRD assessment by flow cytometry on day 35

Treatment as Standard Risk will no longer be continued in patients demonstrating <u>any</u> of the following response features and these patients will be restratified to the High Risk arm of the treatment protocol.

1. Poor Prednisolone response
2. Absence of blast clearance (i.e. > 5% blasts and <25% which is M2 marrow ,centrally reviewed) on bone marrow microscopy at day 35
3. Flow cytometry MRD ≥ 0.01% at Day 35 (progenitor-B disease only)

Please note:

Patients with M3 marrow on Day 35 will continue in the study and go on to the next phase.

Standard Risk, Consolidation Phase

**Week**	**6**	**7**	**8**
**Day**	**1**	**8**	**15**
**ITMTX** As per age (IT)		8	15
[4] **Mercaptopurine** **60mg/m^2/day** (po)	1		21
[5] **Cotrimoxazole** (bd)	1	8	15

[4] **Mercaptopurine:** 60mg/m^2/day once in the evening.

Standard Risk (SR) Consolidation Phase

***Duration of therapy**:* Days 1 – 21*Inclusive* (Beginning of Week 6 – End of Week 8)

Eligibility to commence SR Consolidation Phase

Remission status following Induction chemotherapy.Satisfactory blood counts: Neutrophil > 750/mm^3; Platelet count > 75,000/mm^3.If (a) the blood count has not recovered AND (b) the post-induction bone marrow is hypocellular with <5% blastsdelay the start of the Consolidation phase by ONE week ANDrepeat bone marrow studies to establish remission status

Drug exposure

1. 6-Mercaptopurine (PO)

60mg/m^2 daily, in the evening, Days 1-21 (inclusive; beginning of Week 6 to End of Week 8)
Medication to be taken in the evening without milk products. Administer on an empty stomach (at least 1 hour before the evening meal), no solids for 30 minutes after drug intake . Do not increase dose if Absolute Neutrophil Count is >2000/mm^3

2. Intrathecal Methotrexate (IT)

Two doses, Day 8 and Day 15
Dose by age:Less than 2 years: 8mg; 2 – 3 years: 10mg; ≥ 3 years: 12mg

3. Cotrimoxazole:

Twice a day, two consecutive days (e.g. Saturday and Sunday)

***Body Surface Area** (m2)*	***Cotrimoxazole***	***Trimethoprim***	***Sulfamethoxazole***
0.50 – 0.75	240mg,BD	40mg,BD	200mg,BD
0.76 – 1.00	360mg,BD	60mg,BD	300mg,BD
> 1.0	480mgBD	80mg, BD	400mg,BD

Response assessment post consolidation

Bone marrow assessment at the end of this phase is optional and is at the discretion of the treating physician.

If the patient has not achieved complete remission (CR) at the end of this phase , he or she will be withdrawn from the study.

Standard Risk Interim Maintenance

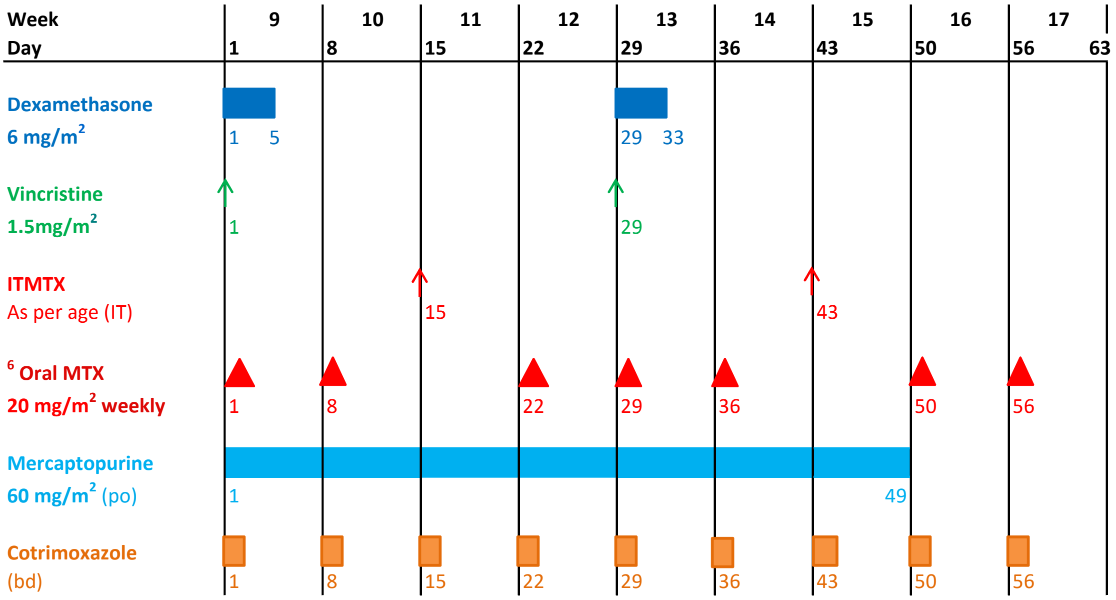

[6] **Oral Methotrexate:** 20mg/m^2 given weekly except on week of intrathecal Methotrexate

Standard Risk (SR) Interim Maintenance Phase

Duration of therapy: Days 1 – 63*Inclusive* (Beginning of Week 9 – End of Week 17)

Eligibility to commence SR Consolidation Phase

Satisfactory blood counts: Absolute Neutrophil Count > 750/mm^3; Platelet count > 75,000/mm^3

Drug Exposure

1. Dexamethasone (PO)

6 mg/m^2 (no maximum dose), two divided doses, Days 1-5 (inclusive, Week 9) and Days 29-33 (inclusive, Week 13)

2. Vincristine (IV)

1.5 mg/m^2 (maximum, 2mg), slow intravenous push

Two doses, Day 1 (Week 9) and Day 29 (Week 13)

3. 6-Mercaptopurine (PO)

60mg/m^2 daily, in the evening, continuously from Day 1-49 (inclusive). Do not give in week 16 (days 50-56)Medication to be taken in the evening without milk products. Administer on an empty stomach.(at least 1 hour before the evening meal).No solids for 30 minutes after drug intake.

4. Oral Methotrexate

20mg/m2 (rounded to the nearest 2.5mg), weekly, Days 1, 8, 22, 29, 36, 50, 56 respectively. Administered as a single dose on a fixed day of a week, taken together with oral 6-Mercaptopurine.

5. Intrathecal Methotrexate (IT)

One dose, Day 15 (Week 11), Day 43 (Week 15)

Dose by age :Less than 2 years: 8mg; 2 – 3 years: 10mg; ≥ 3 years: 12mg

OmitOral Methotrexate on the week of Intrathecal Methotrexate therapy.

6. Cotrimoxazole: Twice a day, two consecutive days (e.g. Saturday and Sunday)

Body Surface Area (m^2)	*Cotrimoxazole*	*Trimethoprim*	*Sulfamethoxazole*
0.50 – 0.75	240mg, BD	40mg, BD	200mg, BD
0.76 – 1.00	360mg, BD	60mg, BD	300mg, BD
> 1.0	480mg, BD	80mg, BD	400mg, BD

Standard Risk, Delayed Intensification Phase
SR-R2A

Week	18	19	20	21	22	23	24	
Day	1	8	15	22	29	36	43	49
**Dexamethasone** $10mg/m^2$ (po)	1–5		15–19					
**Vincristine** $1.5mg/m^2$ (iv)	1	8	15					
**ITMTX** As per age (IT)	1		15					
**E.*coli* Asparaginase 10,000 units/m2**	4, 7	10, 13						
**or PEG Asnase 1000 units** (im)	4							
**Doxorubicin** $25 mg/m^2$	1	8	15					
**Cyclophosphamide** $1,000mg/m^2$ (iv)					29			
**Cytarabine** $75 mg/m^2$/day (IV)/(SC)					30,31,32,33	37,38,39,40		
**Mercaptopurine** $60mg/m^2$/day (po)					29–42			
**Cotrimoxazole** (bd)	1	8	15	22	29	36	43	

Standard Risk, Delayed Intensification Phase
SR-R2B

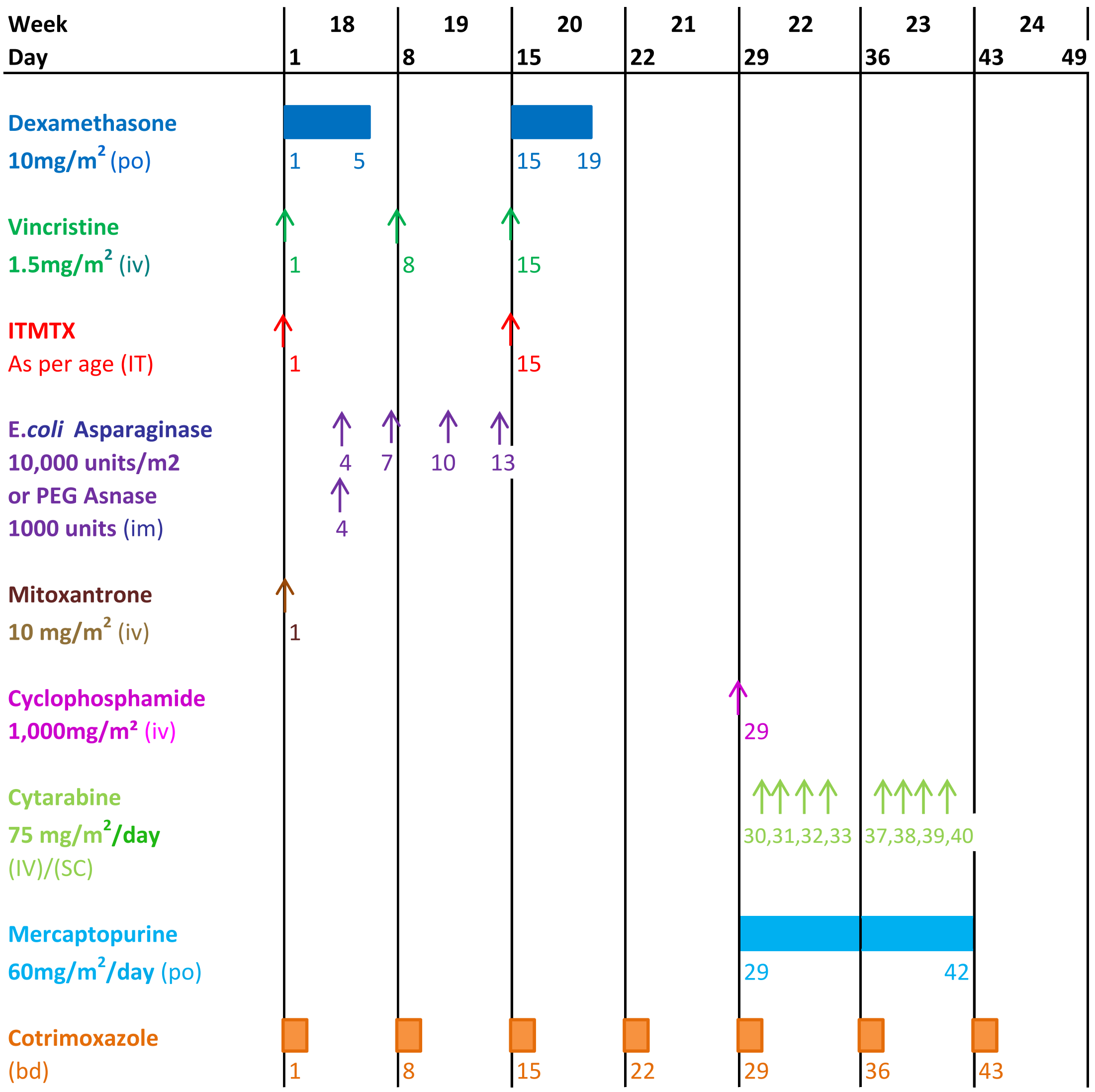

Standard Risk (SR) Delayed Intensification Phase

***Duration of therapy**:* Days 1 – 49 *Inclusive* (Beginning of Week 18 – End of Week 24)
Week 24 is a rest week.

RANDOMISATION ALERT

DOXORUBICIN versus MITOXANTRONE

THREE (3) DOSES OF DOXORUBICIN (SR DELAYED INTENSIFICATION A)
versus
ONE (1) DOSE OF MITOXANTRONE (SR DELAYED INTENSIFICATION B)

Eligibility to commence SR Delayed Intensification Phase:
Satisfactory blood counts: Absolute Neutrophil Count > 750/mm^3; Platelet count >75,000/mm^3

General remarks

Treatment does not need to be interrupted solely on account of myelosuppression.Serious infection (e.g., varicella, neutropenic enterocolitis, invasive fungal infection) will require treatment interruption or scheduling modification. This will be based on the discretion of the attending physician.

***Drug exposure**:*

1. Dexamethasone (PO)
10 mg/m^2 (maximum dose 20mg), two divided doses
Days 1-5 (inclusive, Week 18) and Days 15-19 (inclusive, Week 20)

2. Vincristine (IV)
1.5 mg/m^2 (maximum, 2mg), slow intravenous push, weekly, for three (3) doses
Day 1 (Week 18), Day 8 (Week 19), Day 15 (Week 20)

ALERT: PLEASE ENSURE THAT VINCRISTINE IS ADMINISTERED ONLY AFTER COMPLETION OF INTRATHECAL METHOTREXATE INJECTION. THIS IS TO AVOID INADVERTENT INTRATHECAL INJECTION OF VINCRISTINE

3. L-Asparaginase (IM)
Where available, the PEGylated preparation, Pegaspargase (Oncaspar®) will be administered
Where Pegaspargase is not available, native E. coli L-asparaginase will be administered
Pegaspargase
One doses; 1000 Units/m^2, I.M., Day 4 (Week 18);
OR
Native E. *coli* L-asparaginase

Four doses; 10, 000 Units/m^2, I.M., Days 4, 7, (Week 18), 10, 13 (Week 19)

4. Intrathecal Methotrexate (IT)
Two doses Day 1 (Week 18) ,Day 15 (Week 20)
Dose by age:Less than 2 years: 8mg; 2 – 3 years: 10mg; ≥ 3 years: 12mg

5. Cyclophosphamide (IV)
1000mg/m^2, IV over 30minutes to 1 hour, Day 29 (Week 22)
Give Cyclophosphamide in 100mls in Saline over 30 minutes to 1 hour. Continue hydration at 125ml/m^2 for 2-3 hours.

6. Cytarabine (Ara-C)(IV/SC)
75mg/m^2, IV or SC push for 8 doses
Four consecutive daily doses for two consecutive weeks
Days 30, 31, 32, 33 (Week 22)
Days 37, 38, 39, 40 (Week 23)

7. 6-Mercaptopurine (PO)
60mg/m^2 daily, at night, Days 29-42 (inclusive)
Medication to be taken in the evening without milk products. Administer on an empty stomach (at least 1 hour before the evening meal), no solids for 30 minutes after drug intake .Do not increase dose if Neutrophil >2000/mm^3

8. Doxorubicin *versus* Mitoxantrone

RANDOMISATION
<u>Standard Risk Delayed Intensification A</u>

Doxorubicin (IV)
Three (3) doses, 25 mg/m^2, IV infusion (1 hour)
Day 1 (Week 18), Day 8 (Week 19), Day 15 (Week 20)

<u>Standard Risk Delayed Intensification B</u>

Mitoxantrone (IV)
One (1) dose, 10 mg/m^2, Day 1 (Week 18)
IV infusion over 1 hour, into a side-port of a free-flowing 0.9% Saline infusion

Standard Risk, Maintenance

Week	25	26	27	28	29	30	31	32	33	34	35	36
Day	1	8	15	22	29	36	43	50	57	64	71	78 84
ITMTX<br>As per age (IT)			15									
Oral MTX<br>20 mg/m^2 weekly	1	8		22	29	36	43	50	57	64	71	78
Cotrimoxazole<br>(bd)	1	8	15	22	29	36	43	50	57	64	71	78
Mercaptopurine<br>60 mg/m^2 (po)	1											84

Please note there are 8 such cycles of therapy to end on week 120.

Standard Risk (SR) Maintenance Phase

Duration of therapy:Administered as 12-week cycles; 8 such cycles

Cycle 1 Beginning of Week 25 End of Week 36
Cycle 2 Beginning of Week 37 End of Week 48
Cycle 3 Beginning of Week 49 End of Week 60
Cycle 4 Beginning of Week 61 End of Week 72
Cycle 5 Beginning of Week 73 End of Week 84
Cycle 6 Beginning of Week 85 End of Week 96
Cycle 7 Beginning of Week 97 End of Week 108
Cycle 8 Beginning of Week 109 End of Week 120

Eligibility to commence Maintenance Phase

Satisfactory blood counts: Neutrophil > 1000/mm^3; Platelet count > 100,000/mm^3

General remarks

Maintain Neutrophil count between and 1500/mm^{3}and750/mm^3, platelets above 75,000/mm^3. See **Appendix 14** for dose adjustments.

<u>Drug Exposure:</u>

1. 6-Mercaptopurine (PO)

60mg/m^2 daily, in the evening, continuously daily of each 12-week Maintenance Cycle.
Medication to be taken in the evening without milk products. Administer on an empty stomach (at least 1 hour before the evening meal), no solids for 30 minutes after drug intake

2. Oral Methotrexate

20mg/m^2 (rounded to the nearest 2.5mg), weekly
Eleven (11) weekly doses in each 12-week Maintenance Cycle
Administered as a single dose on a fixed day of a week, taken together with oral 6-Mercaptopurine

3. Intrathecal Methotrexate (IT)

One dose, Day 15 of each 12-week Maintenance Cycle
Dose by age:Less than 2 years: 8mg; 2 – 3 years: 10mg; ≥ 3 years: 12mg
<u>Omit</u>Oral Methotrexate on the week of Intrathecal Methotrexate therapy

END OF B-CELL STANDARD RISK

B-cell PrecursorALL Intermediate Risk

Age ≥ 10 years

OR

Presenting WC ≥50,000/mm^3

OR

Testicular disease/Bulky disease

AND

Prednisolone good response

AND

No high risk cytogenetics

AND

MRD negative(<10^{-3}) at the end of Induction

Summary page – Intermediate Risk

The Intermediate Risk (IR) regimen is for patients at higher risk of relapse due to <u>any</u> of the following features

- Older age (≥ 10 years)
- High presentation white blood cell (WBC) count (≥ 50,000/mm^3)
- Bulky lymph nodes (≥5 cm in peripheral region and in chest >5 cm on CT scan or occupying ≥1/3rd diameter on chest x-ray) and/or bulky liver/spleen reaching to the umbilicus or beyond
- Testicular Disease

Randomisation elements in the Intermediate Risk regimen include

- *During the Induction Phase*
  5 *versus* 3 Weeks Of Prednisolone
- *During the Intensification Phase*
  Doxorubicin (3 doses, Days 1, 8 &15) *versus* Mitoxantrone (1 dose, Day 1)

**Phase**	**Drugs**	**Randomisation**	**Days of therapy**	**Weeks of Therapy**	**MRD**	**Intrathecal Injections**
**Induction**	Prednisolone,Vincristine, IT MTX, Asparaginase(Native or PEG), Daunorubicin	5 weeks (R1A)*vs* 3 (R1B) weeks of Prednisolone	1-35	1-5	Day 35	3 (plus 1 if traumatic tap)
**Consolidation**	IT MTX, Cyclophosphamide, Cytarabine, 6MP	N/A	1-35	6-10	N/A	2
**Interim Maintenance**	Vincristine, IT MTX, IVMethotrexate	N/A	1-56	11-18	N/A	2
**Delayed Intensification**	Pulsed Dexamethasone, Vincristine, IT MTX, Asparaginase (Native or PEG),Cyclophosphamide, Cytarabine, 6MP,Doxorubicin (R2A),Mitoxantrone(R2B)	3 doses of Doxorubicin *vs* 1 dose of Mitoxantrone	1-49	19-25	N/A	2
**Maintenance**	IT MTX, Oral MTX and 6MP	N/A	1-84	8cycles,12 weeks each	N/A	1

Intermediate Risk, Induction phase

IR-R1A

	Week 1	Week 2	Week 3	Week 4	Week 5	
**Day**	**1**	**8**	**15**	**22**	**29**	
[1] **Prednisolone 60mg/m^2** (po)	1	8			28	35
[2] **Vincristine 1.5mg/m^2** (iv)		8	15	22	29	
[3] **ITMTX** As per age (IT)		8	15			35
**E.*coli* Asparaginase 10,000 units/m2**		9, 12	15, 18, 21	24, 27	30	
**or PEG Asnase 1000 units** (im)		9		23		
**Daunorubicin 25mg/m^2**		8	15			
**Cotrimoxazole** (bd)	1	8	15	22	29	

[1] **Prednisolone:** given in three divided doses

[2] **Vincristine:** maximum single dose of Vincristine is 2mg

[3] **Intrathecal Methotrexate:** <2 years= 8mg; 2 years-Less than 3 years = 10mg; ≥3 years= 12mg

Intermediate Risk, Induction phase

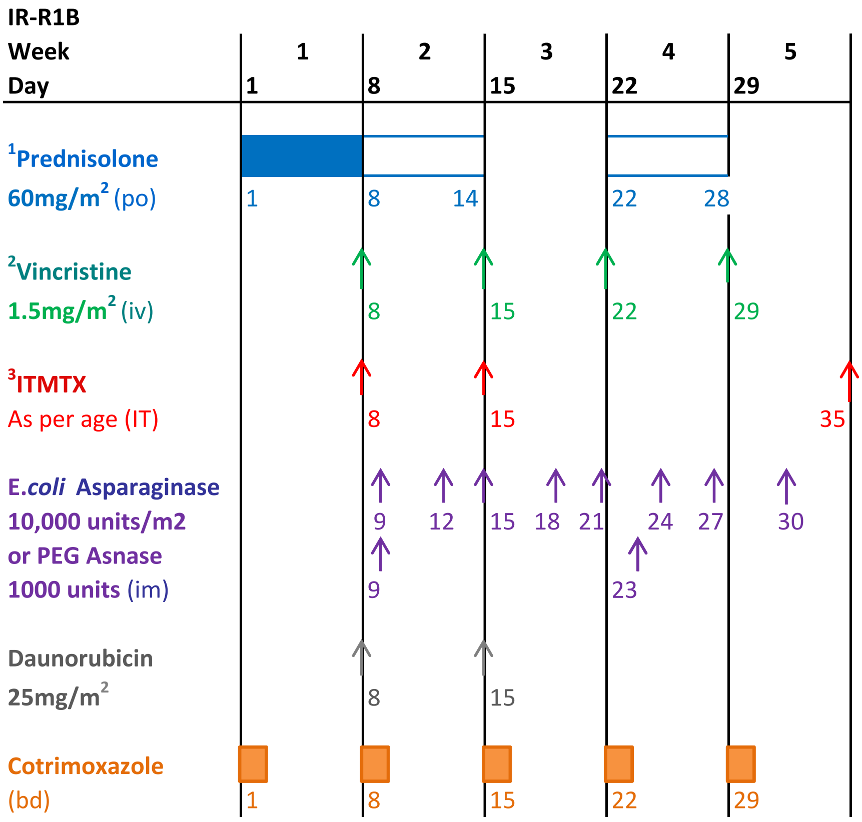

[1] **Prednisolone:** given in three divided doses

[2] **Vincristine:** maximum single dose of Vincristine is 2mg

[3] **Intrathecal Methotrexate:** <2 years= 8mg; 2 years-Less than 3 years= 10mg; ≥3 years= 12mg

Adolescent Patients (≥ 10 years of age)

Intermediate Risk, Induction phase

Week	1	2	3	4	5
**Day**	**1**	**8**	**15**	**22**	**29**
[1]**Prednisolone** **60mg/m^2** (po)	1	8 14		22 28	
[2]**Vincristine** **1.5mg/m^2** (iv)		8	15	22	29
[3]**ITMTX** As per age (IT)		8	15		35
**E.*coli* Asparaginase** **10,000 units/m2**		9 12	15 18 21	24 27	30
**or PEG Asnase** **1000 units** (im)		9		23	
**Daunorubicin** **25mg/m^2**		8	15		
**Cotrimoxazole** (bd)	1	8	15	22	29

[1] **Prednisolone:** given in three divided doses, Days 1-7 max dose of 120 mg
Days 8-14 and Days 22-28 max dose of 80 mg

[2] **Vincristine:** maximum single dose of Vincristine is 2mg

[3] **Intrathecal Methotrexate:** <2 years= 8mg; 2 years- Less than 3 years= 10mg; ≥3 years= 12mg

Intermediate Risk (IR) Induction Phase

Duration of therapy*:* Days 1 – 35 *Inclusive* (Beginning of Week 1 – End of Week 5)

Drug Exposure:

1. Prednisolone (PO)
Prophase

Day 1 to Day 7 (Common to both R1-A and R1-B)
Prednisolone 60mg/m^2 in three divided doses, **note** maximum permitted dose is 120 mg per day.

Please note: ***In patients with poorly responding or rising WBC count during the first 72 hours of the steroid prophase the treating physician may bring forward the introduction of additional cytotoxic therapy. In such cases the steroid prophase is terminated and the treatment schedule is as for multi agent therapy.***

Continuous dosing in IR Induction R1-A

Prednisone 60mg/m^2 (maximum 120mg) in three divided doses
Continuous dosing, Days 8 – 28 (Beginning of Week 1 to End of Week 4)
Taper over 1 week, Days 29 – 35 (Week 5)

Intermittent dosing in IR Induction R1-B

Prednisone 60mg/m^2 (maximum 120mg) in three divided doses
Intermittent dosing :Days 8 – 14 (Beginning of Week 1 to End of Week 2) & Days 22 – 28 (Week 4)

Adolescent Patients :Age≥ 10 Years (Pulsed steroids only)

Days 1-7 (Week 1): Prednisolone 60mg/m^2 (max 120 mg) in 3 divided doses.
Days 8-14(Week 2), Days 22-28 (Week 4): Prednisolone 6 mg/m^2 (max 80 mg/m^2)in 2-3 divided doses.

2. Vincristine(IV)

1.5 mg/m^2 (maximum, 2mg), slow intravenous push, weekly, for four (4) doses
Day 8 (Week 2), Day 15 (Week 3), Day 22 (Week 4), Day 29 (Week 5)

ALERT: PLEASE ENSURE THAT VINCRISTINE IS ADMINISTERED ONLY AFTER COMPLETION OF INTRATHECAL METHOTREXATE INJECTION. THIS IS TO AVOID INADVERTENT INTRATHECAL INJECTION OF VINCRISTINE

3. L-Asparaginase (IM)
Where available, the PEGylated preparation, Pegaspargase (Oncaspar®) will be administered
Where Pegaspargase is not available, native E. coli L-asparaginase will be administered
Pegaspargase
Two doses; 1000 Units/m^2, I.M., Days 9 (Week 2) and 23 (Week 3) ;
OR
Native E. *coli* L-asparaginase
Eight doses; 10,000 Units/m^2, I.M.
Days 9, 12 (Week 2); Days 15, 18, 21 (Week 3); 24, 27 (Week 4); 30 (Week 5)

4. Daunorubicin (IV)
Two doses, 25 mg/m^2, IV infusion (1 hour), Day 8 (Week 2) and Day 15 (Week 3)

5. Intrathecal Methotrexate (IT)
Three doses; Day 8, Day 15, Day 35
Dose by age**:**Less than 2 years: 8mg; 2 – 3 years: 10mg; ≥ 3 years: 12mg

Response assessment post Induction Phase

1. Prednisolone response at Day 8

Prednisone response is reliably assessed only after a minimum cumulative dose of 210 mg/m^2.

Estimation of peripheral blood blast count :

Prednisone Good Response (PGR): Peripheral blood blast count<1000/µL

Prednisone Poor Response (PPR): Peripheral blood blast count≥1000/µL

2. Bone marrow test, Assessed at Day 35

<5% blasts = in remission

5-25% blasts = M2 marrow

>25% blasts = M3 marrow

3. Samples are to be sent for MRD assessment by flow cytometry on Day 35

Treatment as Standard Risk will no longer be continued in patients demonstrating <u>any</u> of the following response features and these patients will be restratified to the High Risk arm of the treatment protocol.

1. Poor Prednisolone response

2. Absence of blast clearance (i.e. > 5% blasts and <25% which is M2 marrow ,centrally reviewed) on bone marrow microscopy at day 35

3. Flow cytometry MRD ≥ 0.01% at Day 35 (progenitor-B disease only)

Please note:

Patients with M3 marrow on Day 35 will be withdrawn from the study.

Intermediate Risk, Consolidation Phase

**Week**	**6**	**7**	**8**	**9**	**10**
**Day**	**1**	**8**	**15**	**22**	**29**
**ITMTX** As per age (IT)		8	15		
**Cyclophosphamide 1,000mg/m²** (iv)	1		15		
[5] **Cotrimoxazole** (bd)	1	8	15	22	29
**Cytarabine 75 mg/m²/day** (IV/SC)	2 ,3,4,5	9,10,11,12	16,17,18,19	23,24,25,26	
[4] **Mercaptopurine 60 mg/m²** (po)	1			28	

[4] **Mercaptopurine:** 60mg/m^2/day once at night.

Intermediate Risk (IR) Consolidation Phase

***Duration of therapy**:* Days 1 – 35*Inclusive* (Beginning of Week 6 – End of Week 10)

Eligibility to commence IR Consolidation Phase

Remission status following Induction chemotherapy.Satisfactory blood counts: Absolute Neutrophil Count > 750/mm^3; Platelet count > 75,000/mm^3.If (a) the blood count has not recovered AND (b) the post-induction bone marrow is hypocellular with <5% blasts delay the start of the Consolidation phase by ONE week ANDrepeat bone marrow studies to establish remission status

<u>Drug Exposure:</u>

1. Cyclophosphamide (IV)

1000mg/m^2, IV over 30 minutes, Day 1 (Week 6) and Day 15 (Week 8)

Dilute Cyclophosphamide in up to 100ml and infuse over 30 minutes. Continue hydration at 125mL/m^2 for 2 hours (no necessity for potassium in IV fluids)

2. Cytarabine (Ara-C)(IV/SC)

75mg/m^2, IV or SC push for 16 doses

Four consecutive daily doses, for four consecutive weeks

Days 2, 3, 4, 5 (Week 6)

Days 9, 10, 11, 12 (Week 7)

Days 16, 17, 18, 19 (Week 8)

Days 23, 24, 25, 26 (Week 9)

3. 6-Mercaptopurine (PO)

60mg/m^2 daily, in the evening, Day 1-28 (inclusive; beginning of Week 6 to End of Week 9)

Medication to be taken in the evening without milk products. Administer on an empty stomach (at least 1 hour before the evening meal), no solids for 30 minutes after drug intake .Do not increase dose if Absolute Neutrophil Count >2000/mm^3

4. Intrathecal Methotrexate (IT)

Weekly for two doses, Day 8 and Day 15

Dose by age: Less than 2 years: 8mg; 2 – 3 years: 10mg; ≥ 3 years: 12mg

Response assessment post consolidation

Bone marrow assessment at the end of this phase is optional and is at the discretion of the treating physician.

If the patient has not achieved complete remission (CR) at the end of this phase , he or she will be withdrawn from the study.

Intermediate Risk Interim Maintenance-IV Methotrexate

**Week**	**11**	**12**	**13**	**14**	**15**	**16**	**17**	**18**
**Day**	**1**	**8**	**15**	**22**	**29**	**36**	**43**	**50**
**Vincristine 1.5mg/m^2 (IV)**	↑ 2	↑ 12		↑ 22	↑ 32	↑ 42		
**ITMTX** As per age (IT)	↑ 1				↑ 31			
**IV MTX 100 mg/m^2 Weekly**	↑ 2	↑ 12		↑ 22	↑ 32	↑ 42		
**Cotrimoxazole** (bd)	■ 1	■ 8	■ 15	■ 22	■ 29	■ 36	■ 43	■ 50

[6] **IV Methotrexate:** 100mg/m^2 given as initial dose on day 2
Escalate subsequent doses by 50mg/m2 to toxicity and modify dosage on following days

Intermediate Risk (IR) Interim Maintenance Phase- Intravenous Methotrexate

Duration of therapy: Days 1 – 56 *Inclusive* (Beginning of Week 11 – End of Week 18)

Eligibility to commence IR Interim Maintenance Phase

Satisfactory blood counts: Absolute Neutrophil >750/mm^3; Platelet count > 100,000/mm^3

<u>Drug Exposure:</u>

1. Vincristine (IV)

1.5mg/m^2 (maximum, 2mg), slow intravenous push

Day 2 (week 11), Day 12 (week 12), Day 22 (Week 14), Day 32 (Week 15), Day 42 (Week 16)

2. Intravenous Methotrexate (IV)

100 mg/m^2 given as initial dose on day 2, escalate subsequent doses by 50mg/m^2 to toxicity and modify dosage on following days

Day 2 (week 11), Day 12 (week 12), Day 22 (Week 14), Day 32 (Week 15), Day 42 (Week 16)

Dose adjustments to be made as outlined earlier

3. Intrathecal Methotrexate (IT)

Two doses, Day 1 (Week 11) and Day 15 (Week 31)

Dose by age:Less than 2 years: 8mg; 2–3 years: 10mg; ≥ 3 years: 12mg

Intermediate Risk, Delayed Intensification Phase
IR-R2A

**Week**	**19**	**20**	**21**	**22**	**23**	**24**	**25**
**Day**	**1**	**8**	**15**	**22**	**29**	**36**	**43**
**Dexamethasone** **10mg/m²** (po)	1 – 5		15 – 19				
**Vincristine** **1.5mg/m²** (iv)	1	8	15				
**ITMTX** As per age (IT)	1		15				
**E.*coli* Asparaginase** **10,000 units/m2**	4, 7	10, 13					
**or PEG Asnase** **1000 units** (im)	4						
**Doxorubicin** **25 mg/m²**	1	8	15				
**Cyclophosphamide** **1,000mg/m²** (iv)					29		
**Cytarabine** **75 mg/m²/day** (IV/SC)					30,31,32,33	37,38,39,40	
**Mercaptopurine** **60mg/m²/day** (po)					29 –	– 42	
**Cotrimoxazole** (bd)	1	8	15	22	29	36	43

Intermediate Risk, Delayed Intensification Phase
IR-R2B

**Week**	**19**	**20**	**21**	**22**	**23**	**24**	**25**
**Day**	**1**	**8**	**15**	**22**	**29**	**36**	**43**
**Dexamethasone** **10mg/m²** (po)	1 5		15 19				
**Vincristine** **1.5mg/m²** (iv)	1	8	15				
**ITMTX** As per age (IT)	1		15				
**E.*coli* Asparaginase** **10,000 units/m2**	4 7	10 13					
**or PEG Asnase** **1000 units** (im)	4						
**Mitoxantrone** **10 mg/m²** (iv)	1						
**Cyclophosphamide** **1,000mg/m²** (iv)					29		
**Cytarabine** **75 mg/m²/day** (IV/SC)					30,31,32,33	37,38,39,40	
**Mercaptopurine** **60mg/m²/day** (po)					29	42	
**Cotrimoxazole** (bd)	1	8	15	22	29	36	43

Intermediate Risk (IR)Delayed Intensification Phase

***Duration of therapy**:* Days 1 – 49 *Inclusive* (Beginning of Week 19 – End of Week 25)

RANDOMISATION ALERT

DOXORUBICIN versus MITOXANTRONE

THREE (3) DOSES OF DOXORUBICIN (IR DELAYED INTENSIFICATION A)

versus

ONE (1) DOSE OF MITOXANTRONE (IR DELAYED INTENSIFICATION B)

Eligibility to commence IR Delayed Intensification Phase

Satisfactory blood counts:Absolute Neutrophil Count >750/mm^3; Platelet count > 100,000/mm^3

General remarks

Treatment does not need to be interrupted solely on account of myelosuppression.Serious infection (e.g., varicella, neutropenic enterocolitis, invasive fungal infection) will require treatment interruption or scheduling modification. This will be based on the discretion of the attending physician.

Drug Exposure:

1. Dexamethasone (PO)

10 mg/m^2 (maximum dose 20mg), two divided doses

Days 1-5 (inclusive, Week 19) and Days 15-19 (inclusive, Week 21)

2. Vincristine (IV)

1.5 mg/m^2 (maximum, 2mg), slow intravenous push, weekly, for three (3) doses

Day 1 (Week 19), Day 8 (Week 20), Day 15 (Week 21)

ALERT: PLEASE ENSURE THAT VINCRISTINE IS ADMINISTERED ONLY AFTER COMPLETION OF INTRATHECAL METHOTREXATE INJECTION. THIS IS TO AVOID INADVERTENT INTRATHECAL INJECTION OF VINCRISTINE

3. L-Asparaginase (IM)

Where available, the PEGylated preparation, Pegaspargase (Oncaspar®) will be administered

Where Pegaspargase is not available, native E. coli L-asparaginase will be administered

Pegaspargase

One doses; 1000 Units/m^2, I.M., Day 4 (Week 19);

OR

Native E. *coli* L-asparaginase

Four doses; 10, 000 Units/m^2, I.M., Days 4, 7 (Week 19) 10, 13 (Week 20)

4. Intrathecal Methotrexate (IT)

Day 1 (Week 19), Day 15 (Week 21)

Dose by age :Less than 2 years: 8mg; 2 – 3 years: 10mg; ≥ 3 years: 12mg

5. Cyclophosphamide (IV)

1000mg/m^2, IV over 30minutes to 1 hour, Day 29 (Week 22)

Dilute Cyclophosphamide in up to 100ml and infuse over 30 minutes. Continue hydration at 125mL/m^2 for 2 hours (no necessity for potassium in IV fluids)

6. Cytarabine (Ara-C)(IV/SC)

75mg/m^2, IV or SC push for 8 doses

Four consecutive daily doses, for two consecutive weeks

Days 30, 31, 32, 33 (Week 23)

Days 37, 38, 39, 40 (Week 24)

7. 6-Mercaptopurine (PO)

60mg/m^2 daily, at night, Days 29-42 (inclusive)

Medication to be taken in the evening without milk products. Administer on an empty stomach (at least 1 hour after the evening meal), no solids for 30 minutes after drug intake.Do not increase dose if Neutrophil > 2000/mm^3)

8. **Doxorubicin *versus* Mitoxantrone**

RANDOMISATION

<u>Intermediate Risk Delayed Intensification A</u>

Doxorubicin (IV)

Three (3) doses, 25 mg/m^2, IV infusion (4 – 6 hours)

Day 1 (Week 19), Day 8 (Week 20), Day 15 (Week 21)

<u>Intermediate Risk Delayed Intensification B</u>

Mitoxantrone (IV)

One (1) dose, 10 mg/m^2, Day 1 (Week 19)

IV infusion over 15 minutes, into a side-port of a free-flowing 0.9% Saline infusion

Intermediate Risk Maintenance

Week	26	27	28	29	30	31	32	33	34	35	36	37
Day	1	8	15	22	29	36	43	50	57	64	71	78
**ITMTX** As per age (IT)			15									
**Oral MTX** 20 mg/m^2 (po)	1	8		22	29	36	43	50	57	64	71	78
**Cotrimoxazole** (bd)	1	8	15	22	29	36	43	50	57	64	71	78
**Mercaptopurine** **60 mg/m^2** (po)	1											84

Please note there are 8 such cycles of therapy to end on week 121

Intermediate Risk (IR) Maintenance Phase

***Duration of therapy**:*Administered as 12-week cycles; 8 such cycles

Cycle 1 Beginning of Week 26 End of Week 37
Cycle 2 Beginning of Week 38 End of Week 49
Cycle 3 Beginning of Week 50 End of Week 61
Cycle 4 Beginning of Week 62 End of Week 73
Cycle 5 Beginning of Week 74 End of Week 85
Cycle 6 Beginning of Week 86 End of Week 97
Cycle 7 Beginning of Week 98 End of Week 109
Cycle 8 Beginning of Week 110 End of Week 121

Eligibility to commence Maintenance Phase

Satisfactory blood counts: Absolute Neutrophil Count > $1000/mm^3$; Platelet count > $100,000/mm^3$

General remarks

Maintain Neutrophil count between 1,500 /mm3 and 750/mm3 and platelets above $75,000/mm^3$. See **Appendix 14** for dose adjustments.

<u>Drug exposure:</u>

1. 6-Mercaptopurine (PO)

$60mg/m^2$ daily, in the evening, continuously daily of each 12-week Maintenance Cycle.
Medication to be taken in the evening without milk products. Administer on an empty stomach (at least 1 hour before the evening meal), no solids for 30 minutes after drug intake

2. Oral Methotrexate

$20mg/m^2$ (rounded to the nearest 2.5mg), weekly
Eleven (11) weekly doses in each 12-week Maintenance Cycle
Administered as a single dose on a fixed day of a week, taken together with oral 6-Mercaptopurine

3. Intrathecal Methotrexate (IT)

One dose, Day 15 of each 12-week Maintenance Cycle
Dose by age:Less than 2 years: 8mg; 2 – 3 years: 10mg; ≥ 3 years: 12mg
<u>Omit</u> Oral Methotrexate on the week of Intrathecal Methotrexate therapy

END OF B-CELL INTERMEDIATE RISK

B-cell Precursor ALL
High Risk

High risk cytogenetics

BCR-ABL
iAMP21
MLL Rearranged
T(17;19)
Hypodiploidy (<40 chromosomes or DNA index <0.81)

OR

Prednisolone poor responder

OR

Incomplete laboratory information required for risk stratification
OR
Patients with atypical clinical features that preclude satisfactory risk stratification

OR

MRD positive (≥10^{-3}) at end of induction

OR

No CR at the end of induction

OR

CNS disease

Summary page – High Risk

The High Risk (HR) regimen is for patients at higher risk of relapse due to <u>any</u> of the following features

- Prednisolone poor responder
- High risk cytogenetics
- CNS disease(based on LP on D8)
- No CR after induction
- MRD positive (≥10^{-3}) at end of induction

Phase	Drugs	Randomisation	Days of therapy	Weeks of Therapy	MRD	Intrathecal Injections
**Induction**	Prednisolone,Vincristine, IT MTX, Asparaginase (Native or PEG), Daunorubicin	N/A	1-35	1-5	Day 35	3<br>(plus 1 if traumatic tap)<br>or<br>(2 more in case of CNS disease)
**Consolidation**	IT MTX, Cyclophosphamide, Cytarabine,Asparaginase(NativeorPEG),Vincristine, 6MP	N/A	1-63	6-14	N/A	3
**Interim Maintenance**	IT MTX, IV Methotrexate, Folinic Acid ,6MP	N/A	1-56	15-22	N/A	4
**Delayed Intensification**	Pulsed Dexamethasone, Vincristine, IT MTX, Asparaginase (Native or PEG),Cyclophosphamide, Cytarabine, 6Mercaptopurine,Doxorubicin (R2A) or Mitoxantrone (R2B)	3 doses of Doxorubicin*vs* 1 dose of Mitoxantrone	1-49	23-29	N/A	2
**Maintenance**	IT MTX,Oral MTX and 6MP	N/A	1-84	8cycles,12 weeks each	N/A	1

High Risk Induction Phase

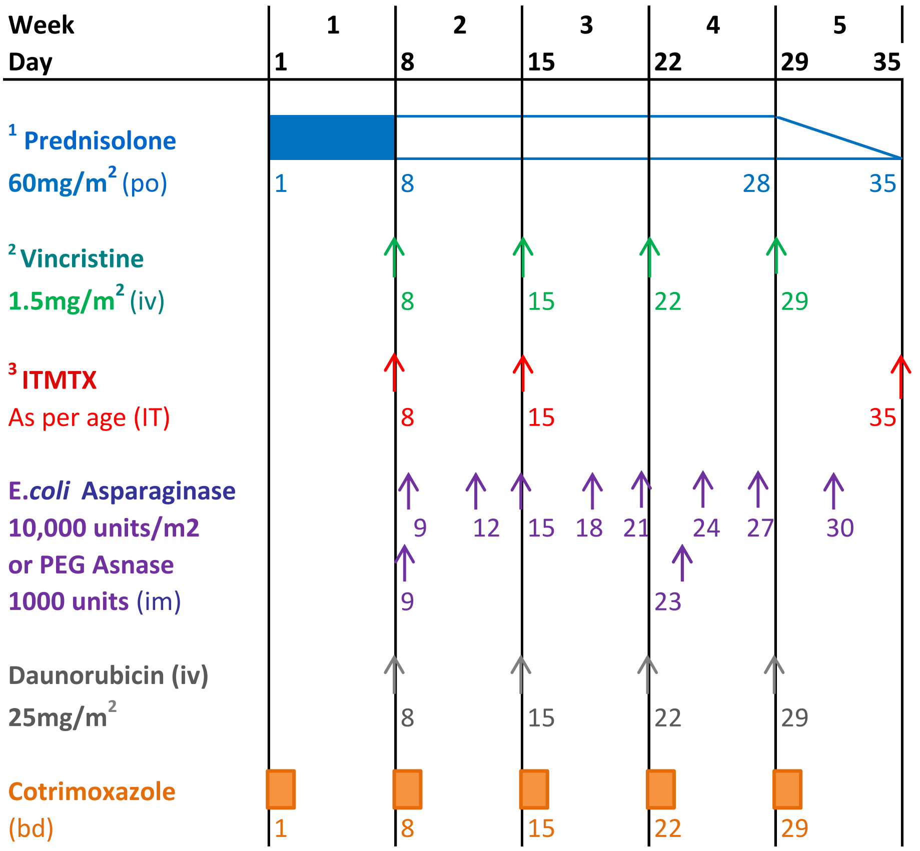

[1] **Prednisolone:** given in three divided doses

[2] **Vincristine:** maximum single dose of Vincristine is 2mg

[3] **Intrathecal Methotrexate:** <2 years= 8mg; 2 years-Less than 3 years= 10mg; ≥3 years= 12mg

MRD estimation on Day 35

High Risk (HR) Induction Phase

Duration of therapy: Days 1 – 35 *Inclusive* (Beginning of Week 1 – End of Week 5)

1. Prednisolone (PO)
Prophase

Day 1 to Day 7
Prednisolone 60mg/m^2 in three divided doses, **note** maximum permitted dose is 120 mg per day.

Please note: ***In patients with poorly responding or rising WBC count during the first 72 hours of the steroid prophase the treating physician may bring forward the introduction of additional cytotoxic therapy. In such cases the steroid prophase is terminated and the treatment schedule is as for multi agent therapy.***

Prednisone 60mg/m^2 (maximum 120mg) in two divided doses
Continuous dosing, Days 8 – 28 (Beginning of Week 1 to End of Week 4)
Taper over 1 week, Days 29 – 35 (Week 5)

2. Vincristine (IV)
1.5 mg/m^2 (maximum, 2mg), slow intravenous push, weekly, for four (4) doses
Day 8 (Week 2), Day 15 (Week 3), Day 22 (Week 4), Day 29 (Week 5)

ALERT: PLEASE ENSURE THAT VINCRISTINE IS ADMINISTERED ONLY AFTER COMPLETION OF INTRATHECAL METHOTREXATE INJECTION. THIS IS TO AVOID INADVERTENT INTRATHECAL INJECTION OF VINCRISTINE

3. L-Asparaginase (IM)
Where available, the PEGylated preparation, Pegaspargase (Oncaspar®) will be administered
Where Pegaspargase is not available, native E. coli L-asparaginase will be administered
Pegaspargase
Two doses; 1000 Units/m^2, I.M., Days 9 (Week 2) and 23 (Week 3) ;
OR
Native E. *coli* L-asparaginase
Eight doses; 10, 000 Units/m^2, I.M.
Days 9, 12 (Week 2); Days 15, 18, 21 (Week 3); 24, 27 (Week 4); 30 (Week 5)

Note: L-asparaginase dose may be rounded off to nearest vial dose; in such cases, a single dose should not be less than 6,000 units/sq.m

4. Daunorubicin (IV)

Four doses, 25 mg/m^2, IV infusion (1 hours), Day 8 (Week 2), Day 15 (Week 3), day 22 (Week 3), day 29 (Week 5). Given on the same days as Vincristine.

5. Intrathecal Methotrexate (IT)

In patients without CNS disease, Three doses; Day 8, Day 15, Day 35
In patients with CNS disease, weekly intrathecal therapy until two consecutive clear CSF samples, for a maximum of **five** intrathecal injections , Day 22 and Day 29 respectively.
Dose by age, Less than 2 years: 8mg; 2 – 3 years: 10mg; ≥ 3 years: 12mg
In cases of indeterminate CNS status (e.g. diagnostic CSF study complicated by traumatic tap with detectable blasts **OR** in cases where no CSF is available for analysis at first LP), an additional intrathecal MTX is administered during Induction (i.e. four [4] IT MTX doses in all) on Day 22 .

NB Children aged ≤ 3years do not receive cranial irradiation. Thus if the clinical conditions do not permit a lumbar puncture at this stage, this can be delayed.

Response assessment post Induction Phase

a. ***Prednisolone response at Day 8***

Prednisolone response is reliably assessed only after a minimum cumulative dose of 210 mg/m^2.

Estimation of peripheral blood blast count :
Prednisolone Good Response (PGR): Peripheral blood blast count<1000/µL
Prednisolone Poor Response (PPR): Peripheral blood blast count ≥ 1000/µ

b. ***Samples are to be sent for MRD assessment by flow cytometry on Day 35***

Patients with non-remission marrow morphology at end of induction (marrow status M2)will receive subsequesnt therapy as high risk disease beginning with HR Consolidation.

Please note:

Patients with M3 marrow on Day 35 will be withdrawn from the study but may continue on treatment.

High Risk Augmented BFM consolidation

Week	6	7	8	9	10	11	12	13	14
Day	1	8	15	22	29	36	43	50	57 63
**ITMTX** As per age (IT)	1	8			29				
**Cyclophosphamide 1,000mg/m²** (iv)	1				29				
Cytarabine **75 mg/m^2/day** (IV or SC)	2 ,3,4,5	9,10,11,12			30,31,32,33	37,38,39,40			
**E.*coli* Asparaginase 10,000 units/m^2 or PEG Asnase 1000 units**			15 18 21<br>16	24			43 46 49<br>44	52	
**Vincristine 1.5mg/m^2** (iv)			16	23			44	51	
[4] **Mercaptopurine 60 mg/m^2** (po)	1	14			29	42			
[5] **Cotrimoxazole** (bd)	1	8	15	22	29	36	43	50	57

[4] **Mercaptopurine:** 60mg/m^2/day once at night.

High Risk (HR) Consolidation Phase

Duration of therapy: Days 1 – 63 *Inclusive* (Beginning of Week 6 – End of Week 14)

Eligibility to commence HR Consolidation Phase:
Remission status following Induction chemotherapy.Satisfactory blood counts: Absolute Neutrophil count > 750/mm^3; Platelet count > 75,000/mm^3.If (a) the blood count has not recovered AND (b) the post-induction bone marrow is hypocellular with<5% blastsdelay the start of the Consolidation phase by ONE week ANDrepeat bone marrow studies to establish remission status

<u>Drug Exposure:</u>

1. Cyclophosphamide (IV)
1000mg/m^2, IV over 30 minutes, Day 1 (Week 6) and Day 29 (Week 9)
Dilute Cyclophosphamide in up to 100ml and infuse over 30 minutes. Continue hydration at)
125mL/m^2 for 2 hours (no necessity for potassium in IV fluids

2. Cytarabine (Ara-C) (IV/SC)
75mg/m^2, IV or SC push for 16 doses
Four consecutive daily doses, for four consecutive weeks:
Days 2, 3, 4, 5 (Week 6)
Days 9, 10, 11, 12 (Week 7)
Days 30, 31, 32, 33 (Week 10)
Days 37, 38, 39, 40 (Week 11)

3. 6-Mercaptopurine (PO)
60mg/m^2 daily, in the evening, Day 1-14 & day 29-42 (inclusive; beginning of Week 6 to End of Week 7 and beginning of week 10-end of week11)Medication to be taken in the evening without milk products. Administer on an empty stomach (at least 1 hour before the evening meal), no solids for 30 minutes after drug intake .Do not increase dose if Neutrophil >2000/mm^3

4. Intrathecal Methotrexate (IT)
Weekly for three doses, Day 1, Day 8 and Day 29
Dose by age: Less than 2 years: 8mg; 2 – 3 years: 10mg; ≥ 3 years: 12mg

5. Vincristine (IV)
1.5 mg/m^2 (maximum, 2mg), slow intravenous push, weekly, for four (4) doses
Day 16 (Week 8), Day 23 (Week 9), Day 44 (Week 12), Day 51 (Week 13)

<u>ALERT</u>: PLEASE ENSURE THAT VINCRISTINE IS ADMINISTERED ONLY AFTER COMPLETION OF INTRATHECAL METHOTREXATE INJECTION. THIS IS TO AVOID INADVERTENT INTRATHECAL INJECTION OF VINCRISTINE

6. L-Asparaginase (IM)
Where available, the PEGylated preparation, Pegaspargase (Oncaspar®) will be administered
Where Pegaspargase is not available, native E. coli L-asparaginase will be administered
Pegaspargase
Two doses; 1000 Units/m^2, I.M., Days 16 (Week 8) and 44 (Week 12) ;
OR
Native E. *coli* L-asparaginase
Eight doses; 10, 000 Units/m^2, I.M.
Days 15, 18, 21, 24, (Week 8-9); Days 43, 46, 49, 52 (Week 12-13)

Note: L-asparaginase dose may be rounded off to nearest vial dose; in such cases, a single dose should not be less than 6,000 units/sq.m

Others
In patients with persistent CNS disease post-induction,

a. Continue weekly Intrathecal Methotrexate
b. Schedule cranial irradiation at the end of delayed intensification for patients ≥3 years old.

Response assessment post Consolidation Phase

Marrow status will be reassessed at end of Augmented BFM consolidation:

a) Compulsory, if patient has not achieved complete remission at the end of Induction

b) Optional and is at the discretion of the treating physician if otherwise

Failure of morphological remission at end of consolidation indicates poor-risk disease and patients will be treated off-study with intent to provide allogeneic stem cell transplantation if feasible.

High Risk Interim Maintenance Phase, HDMTX

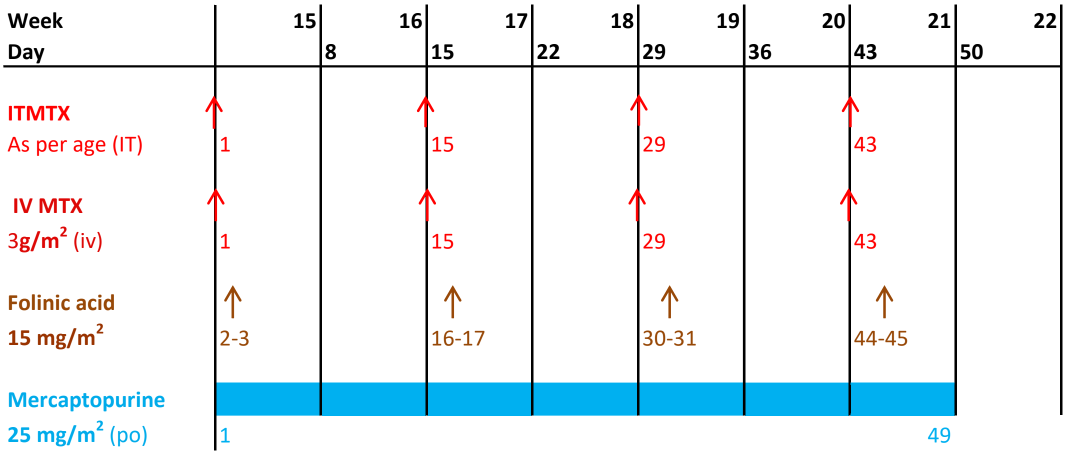

High Risk (HR) Interim Maintenance Phase

Duration of therapy: Days 1 – 56 *Inclusive* (Beginning of Week 15 – End of Week 22)

<u>Eligibility to commence HR IM Phase</u>

This phase runs for 8 weeks from day 1 (beginning of week 15) to day 56 inclusive (end of week 22) (i.e. 8 weeks). Co-trimoxazole must be discontinued at least 6 days prior to commencement of high dose methotrexate.

Prior to starting the first pulse of HDMTX patients must be free of infection, diarrhoea and mucositis and should have an Absolute Neutrophil Count >750/mm^{3}and platelets >100000/mm^3 to start this phase.

Renal function should be within normal limits corrected for age. Prior to starting each subsequent pulse of HDMTX serum creatinine must be <1.5 x baseline or GRF creatinine clearance >65L/minute/1.73m^2.

If renal function does not recover, omit MTX. ALT and AST should be less than 5 x normal. Wherever possible avoid concurrent NSAIDs, aminoglycosides and other nephrotoxic drugs.

Prior to starting each subsequent pulse of high dose methotrexate ANC should be >500/mm^3 and platelets >50000/mm^3. If blood counts are not adequate on the scheduled day of infusion then stop 6-MP. On recovery to ANC >500/mm^{3}and platelets >50,000/mm^{3}then administer the scheduled dose of methotrexate and restart 6-MP at full 25mg/m2 dose.

Drug Exposure:

1. 6-Mercaptopurine (PO)

25mg/m^2 daily, at night, continuously from Day 1-49 (inclusive).

Medication to be taken in the evening without milk products. Administer on an empty stomach (at least 1 hour before the evening meal), no solids for 30 minutes after drug intake

2. Methotrexate :3g/m^{2}intravenously on day 1, 15, 29 and 43 (IV)

All patients must be prehydrated as per guideline in Appendix. The initial 10% of dose is delivered over 30 minutes, the remaining 90% over 23 ½ hours. NB method and schedule of administration should be guided by Appendix 4. Timings of drug administration must be followed strictly.

3. Folinic acid (IV)

15mg/m2 intravenously 42, 48 and 54 hours after the start of each methotrexate infusion. Further details of the folinic acid rescue are given in Appendix 4. Timings of drug administration must be followed strictly.

4. Intrathecal Methotrexate (IT)

Four doses, Days 1(week 15), 15 (week17), 29 (week19), 43(week21)

Dose by age :Less than 2 years: 8mg; 2 – 3 years: 10mg; ≥ 3 years: 12mg

High Risk, Delayed Intensification Phase
HR-R2A

**Week**	**23**	**24**	**25**	**26**	**27**	**28**	**29**
**Day**	**1**	**8**	**15**	**22**	**29**	**36**	**43**
**Dexamethasone** **$10mg/m^2$** (po)	1 – 5		15 – 19				
**Vincristine** **$1.5mg/m^2$** (iv)	1	8	15				
**ITMTX** As per age (IT)	1		15				
**E.*coli* Asparaginase** **10,000 units/m2**	4, 7	10, 13					
**or PEG Asnase** **1000 units** (im)	4						
**Doxorubicin** **$25 mg/m^2$**	1	8	15				
**Cyclophosphamide** **$1,000mg/m^2$** (iv)					29		
**Cytarabine** **$75 mg/m^2$/day** (IV or SC)					30,31,32,33	37,38,39,40	
**Mercaptopurine** **$60mg/m^2$/day** (po)					29 –	– 42	
**Cotrimoxazole** (bd)	1	8	15	22	29	36	43

Intensification - All Regimens NOTE THIS PHASE RANDOMISES BETWEEN DAUNORUBICIN AND MITOXANTRONE

High Risk, Delayed Intensification Phase
HR-R2B

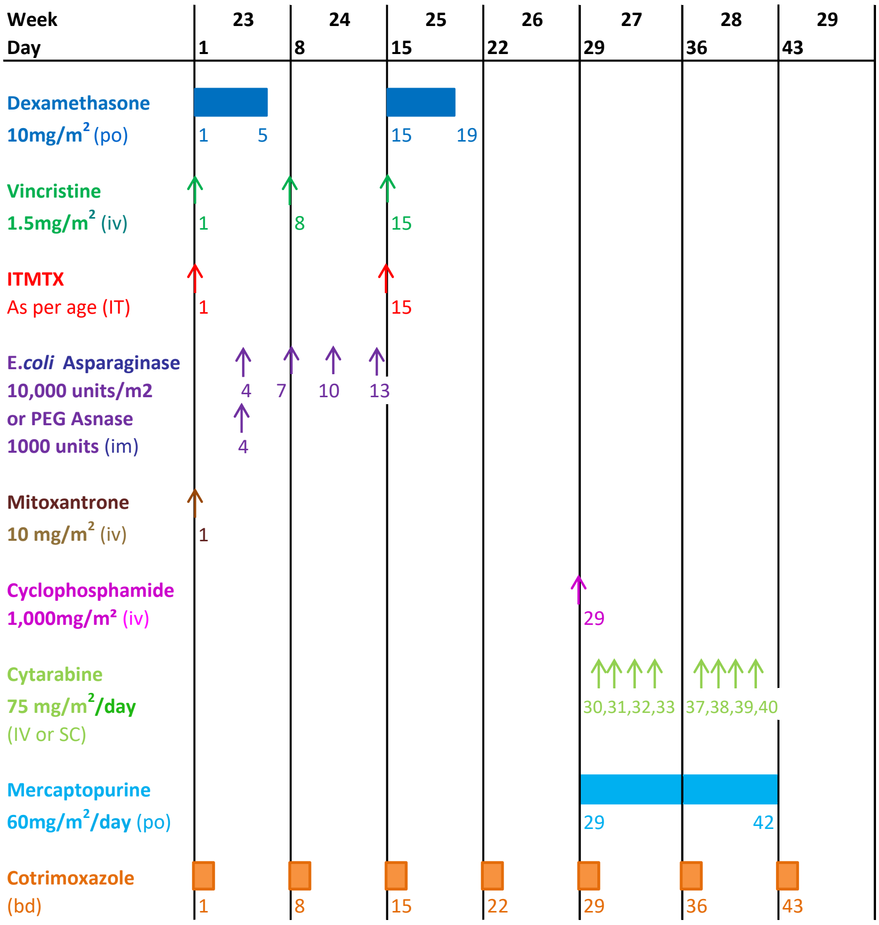

Intensification - All Regimens NOTE THIS PHASE RANDOMISES BETWEEN DAUNORUBICIN AND MITOXANTRONE

High Risk (HR)Delayed Intensification Phase

***Duration of therapy**: Days 1 – 49 *Inclusive* (Beginning of Week 23 – End of Week 29)

RANDOMISATION

DOXORUBICIN versus MITOXANTRONE
THREE (3) DOSES OF DOXORUBICIN (HR DELAYED INTENSIFICATION A)
versus
ONE (1) DOSE OF MITOXANTRONE (HR DELAYED INTENSIFICATION B)

Eligibility to commence HR Delayed Intensification Phase:
Satisfactory blood counts: Absolute Neutrophil Count >750/mm^3; Platelet count >75,000/mm^3

General remarks
Treatment does not need to be interrupted solely on account of myelosuppression.Serious infection (e.g., varicella, neutropenic enterocolitis, invasive fungal infection) will require treatment interruption or scheduling modification. This will be based on the discretion of the attending physician.

Drug Exposure:

1. Dexamethasone (PO)
10mg/m^2 (maximum dose 20mg), two divided doses
Days 1-5 (inclusive, Week 23) and Days 15-19 (inclusive, Week 25)

2. Vincristine (IV)
1.5 mg/m^2 (maximum, 2mg), slow intravenous push, weekly, for three (3) doses
Day 1 (Week 23), Day 8 (Week 24), Day 15 (Week 25)

ALERT: PLEASE ENSURE THAT VINCRISTINE IS ADMINISTERED ONLY AFTER COMPLETION OF INTRATHECAL METHOTREXATE INJECTION. THIS IS TO AVOID INADVERTENT INTRATHECAL INJECTION OF VINCRISTINE

3. L-Asparaginase (IM)
Where available, the PEGylated preparation, Pegaspargase (Oncaspar®) will be administered
Where Pegaspargase is not available, native E. coli L-asparaginase will be administered
Pegaspargase
One doses; 1000 Units/m^2, I.M., Day 4 (Week 23);
OR
Native E. *coli* L-asparaginase
Four doses; 10, 000 Units/m^2, I.M., Days 4, 7 (Week 23)10, 13 (Week24)

Note: L-asparaginase dose may be rounded off to nearest vial dose; in such cases, a single dose should not be less than 6,000 units/m^2

4. Intrathecal Methotrexate (IT)
Two doses; Day 1 (Week 23), Day 15 (Week 25)
Dose by age: Less than 2 years: 8mg; 2 – 3 years: 10mg; ≥ 3 years: 12mg

5. Cyclophosphamide (IV)
1000mg/m^2, IV over 30minutes to 1 hour, Day 29 (Week 27)
Dilute Cyclophosphamide in up to 100ml and infuse over 30 minutes. Continue hydration at 125mL/m^2 for 2 hours (no necessity for potassium in IV fluids)

6. Cytarabine (Ara-C)(IV/SC)
75mg/m^2, IV or SC push for 8 doses
Four consecutive daily doses, for two consecutive weeks
Days 30, 31, 32, 33 (Week 27)
Days 37, 38, 39, 40 (Week 28)

7. 6Mercaptopurine (PO)
60mg/m^2 daily, at night, Days 29-42 (inclusive)
Medication to be taken in the evening without milk products. Administer on an empty stomach (at least 1 hour before the evening meal), no solids for 30 minutes after drug intake
Do not increase dose if Neutrophil > 2000/mm^3

8. Doxorubicin *versus* Mitoxantrone

RANDOMISATION
High Risk Delayed Intensification A
Doxorubicin (IV)
Three (3) doses, 25 mg/m^2, IV infusion (4 – 6 hours)
Day 1 (Week 23), Day 8 (Week 24), Day 15 (Week 25)

High Risk Delayed Intensification B
Mitoxantrone (IV)
One (1) dose, 10 mg/m^2, Day 1 (Week 23)
IV infusion over 15 minutes, into a side-port of a free-flowing 0.9% Saline infusion

High Risk Maintenance

Week	30	31	32	33	34	35	36	37	38	39	40	41
Day	1	8	15	22	29	36	43	50	57	64	71	78
**ITMTX** As per age (IT)			↑ 15									
**Oral MTX** **$20mg/m^2$**	▲ 1	▲ 8		▲ 22	▲ 29	▲ 36	▲ 43	▲ 50	▲ 57	▲ 64	▲ 71	▲ 78
**Cotrimoxazole** (bd)	■ 1	■ 8	■ 15	■ 22	■ 29	■ 36	■ 43	■ 50	■ 57	■ 64	■ 71	■ 78
**Mercaptopurine** **$60\ mg/m^2$** (po)	1											84

Please note there are 8 such cycles of therapy to end on week 125

HighRisk (HR) Maintenance Phase

Note:Patients who receive cranial irradiation do not receive further Intrathecal Methotrexate

Duration of therapy:
Administered as 12-week cycles; 8 such cycles
Cycle 1 Beginning of Week 30 End of Week 41
Cycle 2 Beginning of Week 42 End of Week 53
Cycle 3 Beginning of Week 54 End of Week 65
Cycle 4 Beginning of Week 66 End of Week 77
Cycle 5 Beginning of Week 78 End of Week 89
Cycle 6 Beginning of Week 90 End of Week 101
Cycle 7 Beginning of Week 102 End of Week 113
Cycle 8 Beginning of Week 114 End of Week 125

Eligibility to commence Maintenance Phase
Satisfactory blood counts: Absolute Neutrophil Count > 1000/mm^3; Platelet count > 100,000/mm^3

General remarks

Maintain Neutrophil count between 1500/mm^{3}and 750/mm^{3}and platelets above 75,000/mm^3. See **Appendix 14** for dose adjustments.

Drug Exposure

1. 6-Mercaptopurine (PO)
60mg/m^2 daily, at night, continuously daily of each 12-week Maintenance Cycle.
Medication to be taken in the evening without milk products. Administer on an empty stomach (at least 1 hour after the evening meal), no solids for 30 minutes after drug intake.

2. Oral Methotrexate (PO)
20mg/m^2 (rounded to the nearest 2.5mg), weekly
Eleven (11) weekly doses in each 12-week Maintenance Cycle
Administered as a single dose on a fixed day of a week, taken together with oral 6-Mercaptopurine.

3. Intrathecal Methotrexate (IT)
One dose, Day 15 of each 12-week Maintenance Cycle
Dose by age :Less than 2 years: 8mg; 2 – 3 years: 10mg; ≥ 3 years: 12mg

Omit Oral Methotrexate on the week of Intrathecal Methotrexate therapy

END OF B-CELL HIGH RISK

T-cell ALL

And

T Lymphoblastic Leukaemia

T Cell– Summary Page

Randomisation elements in the T Cell include

- *During the Delayed Intensification Phase*

Doxorubicin (3 doses, Days 1, 8 and 15) *versus* Mitoxantrone (1 dose, Day 1)

**Phases**	**Drugs**	**Randomisation**	**Days of therapy**	**Weeks of Therapy**	**MRD**	**Intrathecal Injections**
**Induction**	Prednisolone,Dexamethasone, Vincristine,IT MTX, Asparaginase (Native or PEG), Daunorubicin	N/A	1-35	1-5	Day 35 (Optional)	3 (plus 1 if traumatic tap)
**Consolidation**	ITMTX, Cyclophosphamide, Asparaginase (Native or PEG),Cytarabine, Vincristine, 6MP	N/A	1-63	6-14	Day 35 (Mandatory)	3
**Interim Maintenance**	IT MTX, IV Methotrexate, Folinic Acid,6 MP	N/A	1-56	11-18	N/A	4
**Delayed Intensification**	Pulsed Dexamethasone, Vincristine, IT MTX, Asparaginase (Native or PEG),Cyclophosphamide, Cytarabine, 6MP,Doxorubicin (R2A),Mitoxantrone(R2B)	3 doses of Doxorubicin*vs* 1 dose of Mitoxantrone	1-49	23-29	N/A	2
**Maintenance**	Oral MTX and 6MP	N/A	1-84	8cycles,12 weeks each	N/A	1

T-cell Induction Phase

Week	1	2	3	4	5	
**Day**	**1**	**8**	**15**	**22**	**29**	**35**
**Prednisolone** **60mg/m²** (po)	1 (days 1–7)					
¹**Dexamethasone** **10mg/m²** (po)		8 – 14		22 – 28		
²**Vincristine** **1.5mg/m²** (iv)		8	15	22	29	
³**ITMTX** As per age (IT)		8	15			35
**E.*coli* Asparaginase** **10,000 units/m2**		9, 12	15, 18, 21	24, 27	30	
**or PEG Asnase** **1000 units** (im)		9		23		
**Daunorubicin (iv)** **25mg/m²**		8	15	22	29	
**Cotrimoxazole** (bd)	1	8	15	22	29	

[1] **Dexamethasone**

[2] **Vincristine:** maximum single dose of Vincristine is 2mg

[3] **Intrathecal Methotrexate:** <2 years= 8mg; 2 years-Less than 3 Years= 10mg; ≥3 years= 12mg

MRD estimation on Day 35 (optional)

T-cell Induction Phase

***Duration of therapy**: Days 1 – 35 *Inclusive* (Beginning of Week 1 – End of Week 5)*

Drug Exposure:

1. Prednisolone (PO)
Prophase

Day 1 to Day 7 (Week 1)
Prednisolone 60mg/m^2 in three divided doses, **note** maximum permitted dose is 120 mg per day.

Please note:* *In patients with poorly responding or rising WBC count during the first 72 hours of the steroid prophase the treating physician may bring forward the introduction of additional cytotoxic therapy. In such cases the steroid prophase is terminated and the treatment schedule is as for multi agent therapy.

2. Dexamethasone (PO)
2 blocks 10mg/m^2 x 2, Days 8-14 (Week 2); Days 22-28 (Week 4), two divided doses.

3. Vincristine (PO)
1.5 mg/m^2 (maximum, 2mg), slow intravenous push, weekly, for four (4) doses
Day 8 (Week 2), Day 15 (Week 3), Day 22 (Week 4), Day 29 (Week 5)

ALERT: PLEASE ENSURE THAT VINCRISTINE IS ADMINISTERED ONLY AFTER COMPLETION OF INTRATHECAL METHOTREXATE INJECTION. THIS IS TO AVOID INADVERTENT INTRATHECAL INJECTION OF VINCRISTINE

4. L-Asparaginase (IM)
Where available, the PEGylated preparation, Pegaspargase (Oncaspar®) will be administered
Where Pegaspargase is not available, native E. coli L-asparaginase will be administered
Pegaspargase
Two doses; 1000 Units/m^2, I.M., Days 9 (Week 2) and 23 (Week 3) ;
OR
Native E. *coli* L-asparaginase
Eight doses; 10, 000 Units/m^2, I.M.
Days 9, 12 (Week 2); Days 15, 18, 21 (Week 3); 24, 27 (Week 4); 30 (Week 5)

Note: L-asparaginase dose may be rounded off to nearest vial dose; in such cases, a single dose should not be less than 6,000 units/sq.m.

5. Daunorubicin (IV)
Four doses, 25 mg/m^2, IV infusion (1 hours), Day 8 (Week 2), Day 15 (Week 3), Day 22 (Week 3), Day 29 (Week 5). Given on the same days as Vincristine.

6. Intrathecal Methotrexate (IT)

In patients without CNS disease, Three doses; Day 8, Day 15, Day 35

In patients with CNS disease, Weekly intrathecal therapy until two consecutive clear CSF samples, for a maximum of five intrathecal injections on Day 22 and Day 29 respectively.

Dose by age, Less than 2 years: 8mg; 2 – 3 years: 10mg; ≥ 3 years: 12mg

In cases of indeterminate CNS status (e.g. diagnostic CSF study complicated by traumatic tap with detectable blasts OR in cases where no CSF is available for analysis at first LP), an additional Intrathecal Methotrexate is administered during Induction (i.e. four [4] Intrathecal Methotrexate doses in all)

Response assessment post Induction phase

a. Prednisolone response at Day 8

Prednisolone response is reliably assessed only after a minimum cumulative dose of 210 mg/m^2.

Estimation of peripheral blood blast count :

Prednisolone Good Response (PGR): Peripheral blood blast count<1000/µL

Prednisolone Poor Response (PPR): Peripheral blood blast count ≥ 1000/µL

b. Samples are to be sent for MRD assessment by flow cytometry on day 35 (Optional)

Patients with non-remission marrow morphology at end of induction (marrow status M2) will continue receiving treatment .

Please note:

Patients with M3 marrow on Day 35 will be withdrawn from the study.

Response assessment of Mediastinal Mass (T-NHL/Lymphoblastic Lymphoma):

For patients with mediastinal enlargment, volumetric measurement of tumour mass should be made as soon after presentation preferably by CT scan. The same modality of imaging should then be used to calculate the reduction in tumour volume on day 35.

≥35% regression of mass- In remission

Patient continues treatment on the same arm and moves to Consolidation phase.

Augmented BFM consolidation

Week	6	7	8	9	10	11	12	13	14
Day	1	8	15	22	29	36	43	50	57
**ITMTX**<br>As per age (IT)	1	8			29				
**Cyclophosphamide**<br>**1,000mg/m²** (iv)	1				29				
**Cytarabine**<br>**75 mg/m²/day**<br>(IV or SC)	2 ,3,4,5	9,10,11,12			30,31,32,33	37,38,39,40			
**E.*coli* Asparaginase**<br>**10,000 units/m²**<br>**or PEG Asnase**<br>**1000 units (im)**			15 18 21<br>16	24			43 46 49<br>44	52	
**Vincristine**<br>**1.5mg/m²** (iv)			16	23			44	51	
[4] **Mercaptopurine**<br>**60 mg/m²** (po)	1	14			29	42			
[5] **Cotrimoxazole**<br>(bd)	1	8	15	22	29	36	43	50	57

[4] **Mercaptopurine:** 60mg/m²/day once at night.

T-cell Consolidation Phase

Duration of therapy: Days 1 –63 *Inclusive* (Beginning of Week 6 – End of Week 14)

Eligibility to commence Consolidation Phase
Remission status following Induction chemotherapy. Satisfactory blood counts:
Absolute Neutrophil Count >750/mm^3; Platelet count > 75,000/mm^3 .If (a) the blood count has not recoveredAND (b) the post-induction bone marrow is hypocellular with <5% blasts delay the start of the Consolidation phase by **ONE** week AND repeat bone marrow studies to establish remission .

Drug Exposure

1. Cyclophosphamide (IV)
1000mg/m^2, IV over 30 minutes, Day 1 (Week 6) and Day 29 (Week 9)
Dilute Cyclophosphamide in up to 100ml and infuse over 30 minutes. Continue hydration at 125mL/m^2 for 2 hours (no necessity for potassium in IV fluids)

2. Cytarabine (Ara-C)(IV/SC)
75mg/m^2, IV or SC push for 16 doses
Four consecutive daily doses, for four consecutive weeks
Days 2, 3, 4, 5 (Week 6)
Days 9, 10, 11, 12 (Week 7)
Days 30, 31, 32, 33 (Week 10)
Days 37, 38, 39, 40 (Week 11)

3. 6-Mercaptopurine (PO)
60mg/m^2 daily, in the evening, Day 1-14 & day 29-42 (inclusive; beginning of Week 6 to End of Week 7 and beginning of week 10-end of week11).
Medication to be taken in the evening without milk products. Administer on an empty stomach (at least 1 hour before the evening meal), no solids for 30 minutes after drug intake.
Do not increase dose if Absolue Neutrophil Count >2000/mm^3

4. Intrathecal Methotrexate (IT)
Weekly for three doses, Day 1, Day 8 and Day 29
Dose by age: Less than 2 years: 8mg; 2 – 3 years: 10mg; ≥ 3 years: 12mg

5. Vincristine (IV)
1.5 mg/m^2 (maximum, 2mg), slow intravenous push, weekly, for four (4) doses
Day 16 (Week 8), Day 23 (Week 9), Day 44 (Week 12), Day 51 (Week 13)

ALERT: PLEASE ENSURE THAT VINCRISTINE IS ADMINISTERED ONLY AFTER COMPLETION OF INTRATHECAL METHOTREXATE INJECTION. THIS IS TO AVOID INADVERTENT INTRATHECAL INJECTION OF VINCRISTINE

6. L-Asparaginase (IM)
Where available, the PEGylated preparation, Pegaspargase (Oncaspar®) will be administered
Where Pegaspargase is not available, native E. coli L-asparaginase will be administered
Pegaspargase

Two doses; 1000 Units/m^2, I.M., Days 16 (Week 8) and 44 (Week 12) ;
OR
Native E. *coli* L-asparaginase
Eight doses; 10, 000 Units/m^2, I.M.
Days 15, 18, 21, 24, (Week 8-9); Days 43, 46, 49, 52 (Week 12-13)

Note: L-asparaginase dose may be rounded off to nearest vial dose; in such cases, a single dose should not be less than 6,000 units/sq.m

Others

In patients with persistent CNS disease post-induction: Continue weekly Intrathecal Methotrexate. Schedule cranial irradiation at the end of delayed intensification for children >3 years of age. Children <3 years of age, who are CNS positive(CNS3 disease) , will receive high dose Methotrexate (5mg/m^2) during Interim Maintenance

Response assessment post Consolidation Phase (Mandatory)

1. Bone marrow test

<5% blasts = in remission
5-25% blasts = M2 marrow
>25% blasts = M3 marrow

2. Samples are to be sent for MRD assessment by flow cytometry

If the patient has not achieved complete remission (CR) at the end of this phase , he or she will be withdrawn from the study.

Response assessment of Mediastinal Mass (T-NHL/Lymphoblastic Lymphoma):

1. Repeated imaging should be done.

2. Mediastinal mass not visible-Patient continues in study in HR arm.

3. Residual Mediastinal mass visible-Remission uncertain . Decision to continue treatment on protocol and further investigations are at the discretion of the treating physician.

T-cell Interim Maintenance Phase, HDMTX

**Week**	**15**	**16**	**17**	**18**	**19**	**20**	**21**	**22**
**Day**	**1**	**8**	**15**	**22**	**29**	**36**	**43**	**50**
**ITMTX** As per age (it)	↑ 1		↑ 15		↑ 29		↑ 43	
**IV MTX** **5g/m^2** (iv)	↑ 1		↑ 15		↑ 29		↑ 43	
**Folinic acid** **15 mg/m^2**	↑ 2-3		↑ 16-17		↑ 30-31		↑ 44-45	
[4] **Mercaptopurine** **25 mg/m^2** (po)	1 ▬▬▬	▬▬▬	▬▬▬	▬▬▬	▬▬▬	▬▬▬	▬▬▬ 49	

T-cell High Risk (HR) Interim Maintenance, HDMTX Phase

***Duration of therapy**: Days 1 – 56 Inclusive* (Beginning of Week 15 – End of Week 22)

Eligibility to commence HR IM Phase

This phase runs for 8 weeks from day 1 (beginning of week 15) to day 56 inclusive (end of week 22) (i.e. 8 weeks). Co-trimoxazole must be discontinued at least 6 days prior to commencement of high dose methotrexate.

Prior to starting the first pulse of HDMTX patients must be free of infection, diarrhoea and mucositis and should have an ANC >750/mm^3 and platelets >100,000/mm^{3}to start this phase. Renal function should be within normal limits corrected for age.

Prior to starting each subsequent pulse of HDMTX serum creatinine must be <1.5 x baseline or GRF creatinine clearancve >65L/minute/1.73m2. If renal function does not recover, omit MTX. ALT and AST should be less than 5xnormal. Wherever possible avoid concurrent NSAIDs, aminoglycosides and other nephrotoxic drugs.

Prior to starting each subsequent pulse of high dose methotrexate ANC should be >500/mm^3 and platelets >50,000/mm^3.If blood counts are not adequate on the scheduled day of infusion then stop 6-MP. On recovery to ANC 500/mm^{3}and platelets >50,000/mm^{3}then administer the scheduled dose of methotrexate and restart 6-MP at full 25mg/m2 dose.

<u>Drug Exposure</u>

1. 6-Mercaptopurine (PO)

25mg/m^2 daily, at night, continuously from Day 1-49 (inclusive).
Medication to be taken in the evening without milk products. Administer on an empty stomach (at least 1 hour before the evening meal), no solids for 30 minutes after drug intake.

2. Methotrexate (IV) : 5g/m^2 intravenously on Days 1, 15, 29 and 43 respectively.
All patients must be prehydrated as per guideline in Appendix. The initial 10% of dose is delivered over 30 minutes, the remaining 90% over 23 ½ hours. NB method and schedule of administration should be guided by **Appendix 4**. Timings of drug administration must be followed strictly.

3. Folinic acid :(IV)

15mg/m^{2}intravenously 42, 48 and 54 hours after the start of each methotrexate infusion. Further details of the folinic acid rescue are given in **Appendix 4**.Timings of drugadministration must be followed strictly.

4. Intrathecal Methotrexate (IT)

Four doses, Days 1 (week 11), 15 (week13), 29 (week15), 43(week17)
Dose by age:Less than 2 years: 8mg; 2 – 3 years: 10mg; ≥ 3 years: 12mg

T-cell Delayed Intensification Phase
R2A

Week	23	24	25	26	27	28	29
Day	1	8	15	22	29	36	43
**Dexamethasone** $10mg/m^2$ (po)	1 – 5		15 – 19				
**Vincristine** $1.5mg/m^2$ (iv)	1	8	15				
**IT MTX** **As per age(IT)**	**1**		**15**				
**E.*coli* Asparaginase 10,000 units/m2**	4, 7	10, 13					
**or PEG Asnase 1000 units** (im)	4						
**Doxorubicin** $25 mg/m^2$	1	8	15				
**Cyclophosphamide** $1,000mg/m^2$ (iv)					29		
**Cytarabine** $75 mg/m^2$/day (IV/SC)					30,31,32,33	37,38,39,40	
**Mercaptopurine** $60mg/m^2$/day (po)					29 –	– 42	
**Cotrimoxazole** (bd)	1	8	15	22	29	36	43

T-cell Delayed Intensification Phase
R2B

Week	23	24	25	26	27	28	29
Day	1	8	15	22	29	36	43
**Dexamethasone** **10mg/m^2** (po)	1 – 5		15 – 19				
**Vincristine** **1.5mg/m^2** (iv)	1	8	15				
**IT MTX** **As per age (IT)**	1		15				
**E.*coli* Asparaginase** **10,000 units/m2**	4, 7	10, 13					
**or PEG Asnase** **1000 units** (im)	4						
**Mitoxantrone** **10 mg/m^2** (iv)	1						
**Cyclophosphamide** **1,000mg/m^2** (iv)					29		
**Cytarabine** **75 mg/m^2/day** (IV/SC)					30,31,32,33	37,38,39,40	
**Mercaptopurine** **60mg/m^2/day** (po)					29 –	– 42	
**Cotrimoxazole** (bd)	1	8	15	22	29	36	43

T-cell High Risk (HR)Delayed Intensification Phase

***Duration of therapy**:* Days 1 – 49 *Inclusive* (Beginning of Week 23 – End of Week 29)

RANDOMISATION
DOXORUBICIN versus MITOXANTRONE

THREE (3) DOSES OF DOXORUBICIN (HR DELAYED INTENSIFICATION A)
versus
ONE (1) DOSE OF MITOXANTRONE (HR DELAYED INTENSIFICATION B)

Eligibility to commence HR Delayed Intensification Phase
Satisfactory blood counts: Absolute Neutrophil Count >750/mm^{3}and Platelet count >100,000/mm^3

General remarks

Treatment need not be interrupted solely on account of myelosuppression.Serious infection (e.g., varicella, neutropenic enterocolitis, invasive fungal infection) will require treatment interruption or scheduling modification. This will be based on the discretion of the attending physician.

Drug Exposure:

1. Dexamethasone (PO)
10 mg/m^2 (maximum dose 20mg), two divided doses
Days 1-5 (inclusive, Week 23) and Days 15-19 (inclusive, Week 25)

2. Vincristine (IV)
1.5 mg/m^2 (maximum, 2mg), slow intravenous push, weekly, for three (3) doses
Day 1 (Week 23), Day 8 (Week 24), Day 15 (Week 25)

ALERT: PLEASE ENSURE THAT VINCRISTINE IS ADMINISTERED ONLY AFTER COMPLETION OF INTRATHECAL METHOTREXATE INJECTION. THIS IS TO AVOID INADVERTENT INTRATHECAL INJECTION OF VINCRISTINE

3. L-Asparaginase (IM)
Where available, the PEGylated preparation, Pegaspargase (Oncaspar®) will be administered
Where Pegaspargase is not available, native E. coli L-asparaginase will be administered
Pegaspargase
One doses; 1000 Units/m^2, I.M., Day 4 (Week 23);
OR
Native E. *coli* L-asparaginase
Four doses; 10, 000 Units/m^2, I.M., Days 4, 7 (Week 23) 10, 13 (Week24)

Note: L-asparaginase dose may be rounded off to nearest vial dose; in such cases, a single dose should not be less than 6,000 units/m^2

4. Cyclophosphamide (IV)
1000mg/m^2, IV over 30minutes to 1 hour, Day 29 (Week 27)
Dilute Cyclophosphamide in up to 100ml and infuse over 30 minutes. Continue hydration at 125mL/m^2 for 2 hours (no necessity for potassium in IV fluids)

5. Cytarabine (Ara-C)(IV/SC)
75mg/m^2, IV push for 8 doses
Four consecutive daily doses, for two consecutive weeks
Days 30, 31, 32, 33 (Week 27)
Days 37, 38, 39, 40 (Week 28)

6. 6-Mercaptopurine (PO)
60mg/m^2 daily, at night, Day 29-42 (inclusive)
Medication to be taken in the evening without milk products. Administer on an empty stomach (at least 1 hour before the evening meal), no solids for 30 minutes after drug intake .Do not increase dose if Neutrophil >2000/mm^3

7. Intrathecal Methotrexate (IT)
Day 1 (Week 19), Day 15 (Week 21)Dose by age: Less than 2 years: 8mg; 2 – 3 years: 10mg; ≥ 3 years: 12mg

8. **Doxorubicin *versus* Mitoxantrone**

RANDOMISATION
High RiskDelayed Intensification A
Doxorubicin (IV)
Three (3) doses, 25 mg/m^2, IV infusion (4 – 6 hours)
Day 1 (Week 23), Day 8 (Week 24), Day 15 (Week 25)
***High RiskDelayed Intensification** B*
Mitoxantrone (IV)
One (1) dose, 10 mg/m^2, Day 1 (Week 23)
IV infusion over 15 minutes, into a side-port of a free-flowing 0.9% Saline infusion

T-cell Maintenance

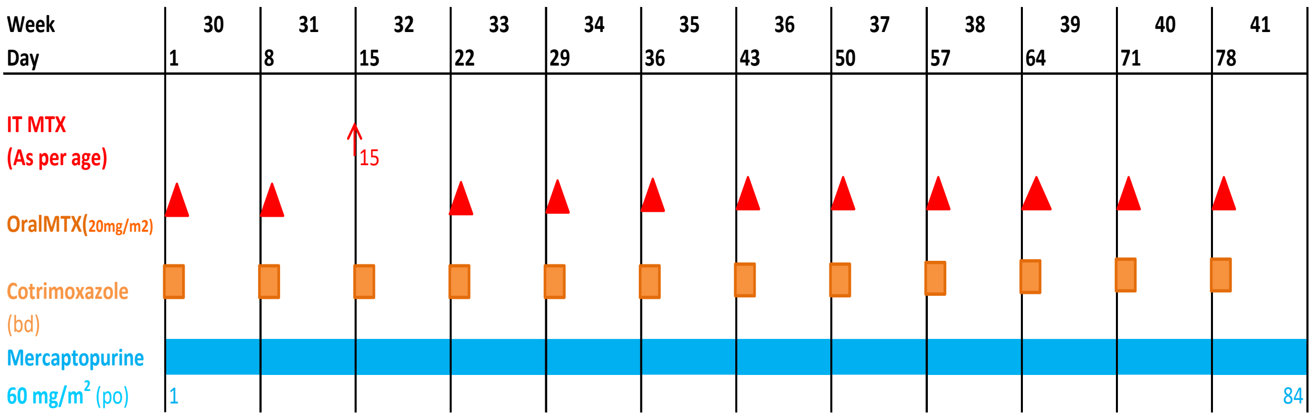

Please note there are 8 such cycles of therapy to end on week 125

T-cell Maintenance Phase

***Duration of therapy**: Administered as 12-week cycles; 8 such cycles*

Cycle 1 Beginning of Week 30 End of Week 41
Cycle 2 Beginning of Week 42 End of Week 53
Cycle 3 Beginning of Week 54 End of Week 65
Cycle 4 Beginning of Week 66 End of Week 77
Cycle 5 Beginning of Week 78 End of Week 89
Cycle 6 Beginning of Week 90 End of Week 101
Cycle 7 Beginning of Week 102 End of Week 113
Cycle 8 Beginning of Week 114 End of Week 125

Eligibility to commence Maintenance Phase
Satisfactory blood counts: Neutrophil > 1000/mm^3; Platelet count > 100,000/mm^3

General remarks

Maintain Neutrophil count between 750/mm^{3}and 150/mm^{3}and platelets above 75,000/mm^3. See **Appendix 14** for dose adjustments.

<u>Drug Exposure</u>

1. 6-Mercaptopurine (PO)
60mg/m^2 daily, at night, continuously daily of each 12-week Maintenance Cycle.
Medication to be taken in the evening without milk products. Administer on an empty stomach (at least 1 hour after the evening meal), no solids for 30 minutes after drug intake

2. Oral Methotrexate
20mg/m^2 (rounded to the nearest 2.5mg), weekly
Eleven (11) weekly doses in each 12-week Maintenance Cycle
Administered as a single dose on a fixed day of a week, taken together with oral 6-Mercaptopurine

3. IT MTX
One dose, Day 15 of each 12-week Maintenance Cycle
Dose by age :Less than 2 years: 8mg; 2 – 3 years: 10mg; ≥ 3 years: 12mg
<u>Omit</u>Oral Methotrexate on the week of Intrathecal Methotrexate therapy

END OF T-CELL

Ph+ and ABL Class Fusion

These children will be treated as High Risk and receive Imatinib continuously at the dose of 360mg/m^2 to be started as soon as the cytogenetic report is available. Imatinib will be continued till the end of last Maintenance phase.

Cytopenias anticipated with TKI during the maintenance phase may require treatment interruption followed by sequential reintroduction of TKI and antimetabolites. Monitoring of response is by prednisolone response and flow cytometry MRD. Serial monitoring of BCR-ABL transcript will be a secondary response indicator.

References:

1. Roberts KG *et al*. Targetable kinase-activating lesions in Ph-like acute lymphoblastic leukaemia. *N Engl J Med*. 2014; 371:1005 - 1015

2. Reshmi SC *et al*. Targetable kinase gene fusions in high risk B-ALL: a study from the Children's Oncology Group. Blood. 2017 Apr 13 (e-pub ahead of print)

Glucose-6-Phosphate Dehydrogenase (G6PD) Deficiency

Please note that these patients do not tolerate doxorubicin and should instead receive daunorubicin or mitoxantrone in delayed intensification. In patient with tumour lysis, also avoid Rasburicase.

Pharmacology and Drug Toxicity

DRUG TOXICITY AND DOSE MODIFICATION:

1. Anthracyclines
2. Cytarabine
3. Cyclophosphamide
4. L- Asparaginase
5. Mercaptopurine
6. Oral Methotrexate
7. IT Methotrexate
8. Mitoxantrone
9. Septran
10. Steroids [Prednisolone and Dexamethasone]
11. Vincristine

Anthracyclines

DAUNORUBICIN

Formulation: Red lyophilised powder for intravenous administration following addition of sterile water for injections. Each vial contains 20mg of daunorubicin base.

Storage:Protected from light at less than 25 degrees C. After reconstitution at 2-8 degrees C in a dark place.

Mixing:Dissolve powder in 4mls of water for injection to give concentration of 5mg/ml.

Stability: Concentration dependent.

Administration:25-45mg/m2 diluted in 0.9% saline or dextrose saline and given IV slowly over 1 hr. **NB** avoid extravasation.

Toxicity: Local necrosis if extravasation occurs. Myelosuppression, mucositis, alopecia, cardiotoxicity. Small risk of secondary AML.

DOXORUBICIN

Formulation: Red lyophilised powder for intravenous administration following addition of sterile water for injections. Each vial contains 10/50mg of daunorubicin base.

Storage:Protected from light at less than 25 degrees C. After reconstitution at 2-8 degrees C in a dark place.

Mixing:Dissolve powder in 4mls of water for injection to give concentration of 2mg/ml.

Stability :Concentration dependent.

Administration: 25-45mg/m2 diluted in 0.9% saline or dextrose saline and given IV slowly over 1 hr. **NB** avoid extravasation.

Toxicity:Local necrosis if extravasation occurs. Myelosuppression, mucositis, alopecia, cardio-toxicity.Small risk of secondary AML.

****Patients who are on intermediate risk and high risk protocols should have an echocardigaram performed prior to initiation of therapy. This should include Left atrial size, Left ventricular ejection fraction &Mitral valve flow velocity.***
Cut off for LVEF abnormaility :< 50%.
American Society for Echocardiography [2006] guidelines for normatives for :Left atrial size & Mitral valve flow velocity.
1 .Left atrial size (in mm) = 28.5 x (BSA)$^{1/3}$ +0.08 x AGE -0.9 ± 18%
2. MV flow velocity = E/A < 1 will be taken as abnormal diastolic function

CYCLOPHOSPHAMIDE

Formulation: 100mg; 200mg; 500mg & 1 gm vials.

Storage: At room temperature

Mixing: Drug is dissolved in NS or 5% dextrose and administered as an infusion

Administration: Administer in 100ml normal saline over 30 minutes; followed by fluids @ 125ml/m2 /hour for a period of 4 hours. MESNA is not needed.

TOXICITY AND DOSE MODIFICATION: Marrow suppression, nausea, vomiting, alopecia and hemorrhagic cystitits, nephrotoxicity.

Prior history of gross haematuria or microscopic haematuria: Hydrate at 125 ml/m²/hr for24 hours after dose and use Mesna 360 mg/m² pre, and 4, 7, 11 hours post dose.

Acute fluid retention: Treat with frusemide and saline; do not modify dose.

CYTARABINE

Formulation: Formulation Ready diluted vials, containing 100mg/ ml. Also available as a 20mg/ml (100mg/5ml) which is used for low dose boluses.

Storage: At room temperature.

Stability: 48 hours at room temperature.

Administration: Reconstitution with supplied dilutent as recommended. Can further dilute with 0.9% saline for infusions by direct IV injection (slow bolus). [IV or SC in interim maintenance].Upto 3000mg/m^2 IV [3hr infusion] every 12 hours.

TOXICITY AND DOSE MODIFICATION :Myelosuppression, alopecia, nausea, vomiting, oral ulceration, fever and arthralgia. Neurotoxicity has been observed mainly with high dose cytarabine manifesting as an acute cerebellar syndrome.

Hyperbilirubinemia Bilirubin> 7.0mg/dl ;**Omit**
Bilirubin 5-7mg/dl : Administer 25% dose .
Bilirubin 3-5 mg/dl:Administer 50% dose.
Bilirubin < 3mg/dl :Administer full dose.
Altered transaminases do not need dose adjustment.

Neurotoxicity : Stop drug

Intrathecal (if substituted for Methotrexate):Only preservative free drug should be given. The dose is 20mg <2yrs, 25mg : 2-3 years and 30mg >3yrs. Rare side effects include aseptic meningitis and myelitis.

L-ASPARAGINASE

Asparaginase (Oncospar) PEG

Dose: 1000 units/m^2(if surface area >0.6m^2) given IM, not subcutaneously.

Formulation : 3,750 units per 5ml vial. DO NOT SHAKE

Storage: At 2-8ºC in refrigerator.

Stability: Single use only. Can be kept for 24 hours only after reconstitution.

Administration: By intramuscular injection. No more than 2 mls in any one site. Do not use if solution is cloudy

L-asparaginase (Native)

Dose : 10,000units /m^2

Formulation : 10,000units & 5000 unit vials

Stability : Single use only. Can be kept for 24 hours only after reconstitution.

Administration: Given intramuscular.No more than 2 ml at each site.

When giving drug in children with thrombocytopenia, ensure application of extra local pressure.

Toxicity & Dose Modifications

Anaphylaxis or anaphylactoid reactions: DISCONTINUE DRUG. The patient can be given Erwinase instead.
Symptomatic pancreatitis: The drug needs to be stopped. Pancreatitis is to be documented by an elevated serum amylase or lipase level or imaging [ultrasonographic/CT] abnormalities. Do not give further doses if there is a prior history of asparaginase induced pancreatitis.
Hyperglycemia:Do not modify dose. Administer Insulin as required.
Ketoacidosis:The drug is withheld until blood glucose can be regulated with insulin.
Liver Dysfunction: Check LFTs only if patient jaundiced. Withhold if total bilirubin > 3mg/dl. Do not alter dose for abnormal transaminases.

ORAL METHOTREXATE

Formulation: 2.5 mg and 10 mg scored tablets. No suspension available

Storage:At room temperature in a dark place.

Stability: Please note the expiry date.

Administration: Is administered once a week, one hour after food. **Omit** dose in the week of IT Methotrexate.

TOXICITY AND DOSE MODIFICATION :

Mucositis: If patient has grade 2 mucositis for more than 3 days, decrease the dose by 30%. For grade 3-4 , withhold drug till resolved and then restart at 50% doses; escalating to 75 &100% at weekly/fortnightly intervals if patient remains well.
Liver: LFT's are not done routinely. Test is done only if the patient has jaundice. If bilirubin is > 3.0 gm/dl the drug is to be stopped. Drug to be at 50% dose if bilirubin is between 1.2-3.0 gm/dl., Dose is to be increased gradually at fortnightly intervals. Dose does not changed with altered aminotransferase levels.
Kidneys: Drug is to be stopped in case of any renal dysfunction. It is to be resumed at full dose once the renal function returns to normal.
Oral toxicity: for grade 2 mucositis of more than 3 days duration, decrease intravenous MTX dose by 30% to 70% of previous dose. Each subsequent dose has to be escalated by 50mg/m^2, as tolerated. For grade 3-4, mucositis, withhold intravenous MTX until mucositis is passive. The drug is resumed at 50% of the previously attained dose and subsequently escalated to 75% and then to 100% dose at 10 day intervals provided grade 3-4 toxicity does not recur.
Haematological toxicity: Omit methotrexate if ANC<750/mm^3 or platelets <75x10^9/L; it should be reinstituted on the first due date following the omitted dose when ANC>0.75x10^9/L and platelets >75x10^9/L, and the dosage should be decreased to 80% of the previously administered dose. Escalate each subsequent dose by 50mg/m^2, as tolerated. Missed doses will not be made up. If counts do not recover within 21 days, check bone marrow status.
Liver Dysfunction: If bilirubin is between 1.5 and 3.0 mg/dl [20-50 µmol/L] decrease MTX to 50% dose and if bilirubin > 3.0mg/dl [>50 µmol/L] omit intravenous MTX until it is less than 1.5mg/dl [20 µmol/L], and then restart at 50% of the previously attained dose. Escalate from 50% to 75% to 100% dose at 10-day intervals provided hyperbilirubinaemia does not recur. Do not modify dosage for elevated aminotransferases.
***Kidney Dysfunction*:** (Grade 3-4): Omit intravenous MTX until grade 0 toxicity (i.e. completely resolved). Resume at 100% of the previously attained dose and then escalate each subsequent dose by 50mg/m^2, as tolerated and continue at 10-day intervals.

N.B:*Toxicity is as per NCI criteria*

INTRATHECAL METHOTREXATE

Formulation : Ampule 1ml =15mg ;Vial 3ml = 15mg

Preservative Free Methotrexate For IT Therapy

Dose is administered as per age of child :<2yrs: 8mg ;2yrs: 10mg; ≥3yrs: 12mg

Encephalopathy during BFM Consolidation or Delayed Intensification

No further **IT MTX** should be administered while patient is also receiving Ara-C (including in future courses containing Ara-C).
Re-expose, and in the absence of recurrence, administer missed doses during Interim Maintenance or maintenance.
In the event of recurrence, change to **IT Ara-C + Hydrocortisone** in the doses given below.

Encephalopathy during other courses

Re-expose as above and continue **IT MTX** if no recurrence.
Change to **IT Ara-C** and **Hydrocortisone** if problem recurs. Doses as below.

Doses:

Age	***Ara- C***	***Hydrocortisone***
≤1	20 mg	7.5 mg
1-2	25 mg	10 mg
3+	30 mg	12.5 mg

MITOXANTRONE

Formulation: 10 ml vials at 2mg/ml concentrations available.

Storage: Store at room temperature

Reconstitution: Ready diluted – should be diluted further with at least 50ml of 0.9% saline or 5% dextrose. Stability Depends on local policy and actual product used – varies between different manufacturers

Administration: As IV infusion (as per protocol)

TOXICITY AND DOSE MODIFICATION:

Cardiotoxicity – NB cumulative doses.Myelosuppression, nausea, vomiting, alopecia and mucositis.

Counselling point: Discolouration of urine and rarely, nails and skin (blue)

6-MERCAPTOPURINE

Formulation: 50 mg tablets. No suspension/liquid available

Storage: At room temperature

Stability: Please note expiry date

Administration:60 mg/m^2 (titratable) during maintenance. Doses to be taken once a day one hour after food in the evening. Do not administer with milk. The dose may be adjusted over days [½ and 1 tablet on alternate days as the tablet is small and not easy to divide]

TOXICITY AND DOSE MODIFICATION:
Bone marrow depression, liver dysfunction.6 mercaptopurine and methotrexate are the only drugs which are interrupted during Maintenance therapy for myelosuppression. The omitted doses will not be made up. The oral doses of6 MP and MTX should be adjusted to maintain ANC between 0.75 and 1.5 x 109/l and platelets between 75 and 150 x 109/l.

COTRIMOXAZOLE

Trimethoprin sulfamethoxazole is used for PCP prophylaxis The drug is given throughout treatment. It is started with induction Therapy. It is given orally on 2 consecutive days every week. The drug should NOT be administered on the same day as oral methotrexate during maintenance therapy.

When to stop the drug ?

1. If a child remains cytopenic after being off chemotherapy for three weeks or more during Maintenance therapy this drug should be stopped.
2. Cotrimoxazole should be reintroduced once 6 Mercaptopurine and Methotrexate are back to standard doses. If cytopenias lasting more than 3 weeks recur then the drug should be stopped for at least 2 months and an alternative prophylaxis considered.
3. 6-Mercaptopurine and methotrexate doses are priority to cotrimoxazole .

Dose of Septran

Surface area	***Co-trimoxazole***	***Trimethoprim***	***Sulphamethoxazole***
0.5-0.75 m2	240 mg bd	40 mg bd	200 mg bd
0.76-1.0 m2	360mg bd	60mg bd	300mg bd
over 1.0 m2	480mg bd	80mg bd	400mg bd

Alterative PCP Prophylaxis

Alternative drugs to Cotrimoxazole are indicated as in case of repeated cytopenias [as above] or drug hypersensitivity.

The drugs in order of preference include:

1.Dapsone
Dapsone is considered the first alternative choice .This is given in a dose of 2mg/kg (maximum dose 100mg) once daily or 4mg/kgweekly (maximum dose 200mg) orally. Side effects of dapsone include fever, rash, and haemolytic anaemia.*G6PDqualitative assay should be performed before startingdapsone therapy.*

2.Nebulised pentamidine
Pentamidine is given monthly. The dose is 300 mg per month is given by nebuliser. It is diluted in 6 ml sterile water and delivered at 6 L/min until the reservoir is dry. This is over a period of 45 minutes to an hour.

3. Atovaquone
Atovaquone is given at a dose of 30mg/kg for children orally once a day withfood. Side effects include gastrointestinal intolerance, rash, headache, and fever. Pentamidine 4mg/kg IV every 2-4 weeks is used if none of the other alternatives are suitable available.

STEROIDS

Administration: The drug is to be given twice a day with meals. An antacid may be given if needed. Side effects of steroids are numerous.

Formulations:

Dexamethasone: Tablets of 0.5mg; 2mg and 4 mg. No liquid preparation

Prednisolone: Tablets of 5mg; 10mg and 20mg.Prednisone is available in a liquid formulation.5 mg/5ml

TOXICITY & DOSE MODIFICATIONS

1. ***Hypertension:*** Steroid should not be reduced. Sodium restriction and anti-hypertensives need to be started. A beta blocker is the preferred drug.
2. ***Malignant Hypertension:*** Reduce dose by 33%. Management as per institute protocol for malignant hypertension.
3. ***Hyperglycemia:*** This may need insulin therapy**.** This is not an indication to decrease/stop the drug.
4. ***Pancreatitis:*** The drug is continued.
5. ***Psychosis:*** Administer half dosage of steroid.
6. ***Suspected steroid-induced myopathy:*** The dose is continued.
7. ***Avascular necrosis:*** This usually develops in maintenance therapy .
8. ***Varicella Zoster:*** Steroids should be withheld if varicella develops during maintenance therapy.

VINCRISTINE

Dose: 1.5mg/m2. Maximum dose : 2mg

Formulation: 1 mg vials with 10 ml of diluent,1mg/ml injection solution

Storage: At 2-8ºC in refrigerator

Stability: Depends on formulation

Administration: By bolus intravenous injection. Ensure that the needle is well into the vein and avoid extravasation. Children <10 years must have vincristine diluted to 0.1mg/ml.

TOXICITY AND DOSE MODIFICATIONS:

Local necrosis if extravasation occurs. Jaw pain, paresis, constipation, systemic neurotoxicity and alopecia are seen frequently.

1. ***Seizures:*** Hold 1 dose, then reinstitute.
2. ***Severe foot drop, paresis or ileus:*** Hold dose(s); institute aggressive regimen to treat constipation (except enemas if neutropenic), if present. When symptoms abate, resume at 1.0 mg/m^2; escalate to full dose as tolerated.
3. ***Jaw pain:*** Treat with analgesics; do not modify vincristine dose.
4. ***Hyperbilirubinemia:*** Check LFTs only if patient jaundiced**.** Withhold if total bilirubin >3 gm/dl. Administer 50% of dose if total bilirubin is between 1.5-3.o gm/dl. Do not alter dose for abnormal transaminases.

Vincristine, given by the wrong route of administration, causes ascending myelitis, excruciating pain, paralysis and death, when given into the CSF.

Appendix 1: Approximate cost of drugs

PRICE IN INR

Drug	Strength of Vial/Tablet	Cost per vial/tablet	Cost for 1m^2 per dose
**Vincristine**	1mg	50.00	100.00
**Doxorubicin**	10mg	170.00	500.00*
**Doxorubicin**	50mg	1100.00	
**Daunorubicin**	20mg	475.00	750.00*
**Cytarabine**	100mg	200.00	200.00
**Cyclophosphamide**	200mg	50.00	200.00
**Cyclophosphamide**	500mg	100.00	
**L Asparaginase**	10.000 units	1,800.00	1200.00*
**L Asparaginase**	5,000 units	1,200.00	
**PEG Asparaginase**	3,750 units	120,000.00	40,000*
**Methotrexate [Inj]**	15 mg	50.00	50.00
**6 Mercaptopurine**	50mg	100.00 [10 tabs]	15.00
**Methotrexate [Tab]**	2.5mg	50.00 [10 tabs]	20.00
**Septran [Tablet]**	80mg	6.50	
**Septran [Syrup]**	40mg/5ml	10.00 [50 ml]	
**Prednisolone**	20mg	18.00 [10 tabs]	
**Prednisolone**	10mg	10.00 [10 tabs]	
**Dexamathasone**	4mg	22.00 [10 tabs]	
**Dexamethasone**	2mg	11.00 [10 tabs]	

NB:* *Cost calculated presuming vial is being shared..Cost may be higher in some ; eg in Vincristine 2 vials would be used. It may not be possible to share.Cost varies between companies. This is an approximated cost.*

Appendix 2: Statistical Plan

1. Introduction

This statistical analysis plan describes the analyses required for the Childhood Acute Lymphoblastic Leukemia (ALL) (ICICLE ALL-14) open label randomised clinical trial. The analysis plan focuses on both efficacy and safety summaries and analysis of efficacy and safety endpoints.

2. Aim of the Trial

The trial seeks to (i) establish a uniform standard of care for newly diagnosed children (aged 1-18 years of age) with ALL across centres in India (ii) improve the prognosis of children with ALL to those published reports (~65%).

3. Participating Centres

Tata Medical Center, Kolkata (Nodal Centre)

Tata Memorial Centre, Mumbai

Postgraduate Institute of Medical Education and Research, Chandigarh

All India Institute of Medical Sciences, New Delhi

MAX Superspeciality Hospital, New Delhi

Cancer Institute (WIA), Chennai

4. Study Design

The InPOG-ALL-15-01, is a multicentre, treatment optimisation, open label randomised trial. It contains two phase III randomisations.

R1 - Randomisation: Prospective, randomised, open label phase III study comparing the efficacy and toxicity of 3 weeks of corticosteroid versus 5 weeks of corticosteroid during induction.

R2 - Randomisation: Prospective, randomised, open label phase III study comparing the efficacy and toxicity of 3 doses of Doxorubicin versus 1 dose of Mitoxantrone.

5. Risk Stratification

There is a two-stage risk stratification. For patients with BCP-ALL prior to start of therapy, patients with a poor response to prednisolone on day 8 and/or CNS3 and/or high risk cytogenetics are classified as high risk (HR). Patients with good prednisolone response, non-high risk cytogenetics and

CNS1/2 are classified as standard risk (SR) if NCI standard risk and intermediate risk (IR) if NCI high risk. T-ALL patients are not risk stratified.

Where risk stratification is not available prior to diagnosis, patients are classified as HR.

At the end of induction SR and IR BCP-ALL patients are re-classified as HR if the minimal residual disease (MRD) is $\geq 10^{-4}$. Where MRD is not evaluable, patients are classified as HR.

6. Study population

1. Age > 1-year and <18 years at time of enrolment
2. Previously untreated
3. ALL diagnosis confirmed by morphology and flow-cytometry

Exclusion Criteria

Patients with any of the following conditions or treatments will be excluded from this study:

1. Previously treated
2. ALL-L3

BCP-ALL - SR

Age >1 and <10 years

WC $< 50 \times 10^9$/L

Prednisolone good responder

No CNS disease

No bulky disease

No high-risk cytogenetics

In CR at end of induction with MRD $<10^{-4}$

BCP-ALL – IR

Age ≥10 and <18years

WC $\geq 50 \times 10^9$/L

Prednisolone good responder

Bulky disease or testicular disease

No CNS disease

No high-risk cytogenetics

In CR at end of induction with MRD $<10^{-4}$

<u>BCP-ALL – HR</u>

High risk cytogenetics

Prednisolone poor responder

CNS disease

End induction MRD ≥10^{-4}

Patients who have been treated prior to enrollment are not eligble for the trial with the exception of those who have received steroids only provided this is not for longer than 8 days. If the patient has received steroids for longer than this, they can still be enrolled provided peripheral blast can be detected which automatically places them as HR.

Those who have received one dose of vincristine are also eligible for the study but not for the first randomisation.

Those who have received one dose of IT Methotrexate are eligible for recruitment to the study and randomisation, but will be analysed separately.

7. Definitions

Complete Remission (CR): At the end of induction **-** <5% blasts at the end of induction in a regenerating marrow; no blasts in CSF. Testicular disease should be in clinical remission at the end of consolidation

Induction death: a treatment- and/or disease-related death that occurs during induction prior to achievement of a CR.

Non-response: patients who have a persisting M3 marrow (≥ 25% ALL cells) after the induction phase (SR arm A week 5, arm B week 6) or who have not achieved a CR (< 5% ALL cells in the bone marrow, no evidence of extramedullary disease) after the first consolidation element (SR arm A week 9, arm B week 10).

Death in remission: Death dues to any cause after achievement of a CR.

Treatment Related Mortality (TRM): Death in a patient in CR with a temporal and/or causal relationship to treatment

Relapse Death: Death in a patient with relapsed ALL due to any cause

Relapse: Disease recurrence after achieving CR. This includes >5% blasts in a bone marrow aspirate, confirmed with flow cytometry; >5 blasts in CSF; biopsy proven testicular relapse or at other sites

Secondary malignancy: any subsequent malignant disease occurring after diagnosis of the ALL which is not relapsed ALL

Event Free Survival (EFS): EFS is defined as the time from entry into the trial or randomisation, using the date of registration until induction failure, relapse, second malignancy or death, censoring at last contact.

Overall Survival (OS): OS is defined as the time from entry into the trial or randomisation to death, censoring at last contact.

Withdrawal: A patient is considered withdrawn if (i) patient withdraws consent (ii) there is unacceptable toxicity and families and physicians agree to withdraw the patient (iii) there is disease progression. This is defined as persistence of morphological disease after consolidation; persistence of MRD positivity prior to delayed intensification or adverse genetic factors identified during the course of trial (e.g. TCF3-HLF1) which suggest that alternative therapeutic approaches are in the best interest of the patient.

Toxicity: Toxicity will be defined using CTCAE 4.03. Only trial specific toxicities will be measured and only grade 3-5 toxicities will be analysed. Grades 3-4 will be grouped and grade 5 (death) analysed separately.

Abandonment: Where a patient who is in CR is lost to further follow up.

8. Study Objectives

8.1 Primary Objectives

1. During induction comparison of toxicity with 5 weeks of corticosteroid versus 3 weeks of corticosteroid.
2. During intensification EFS of patients randomised to receive either 3 doses of doxorubicin or 1 dose of mitoxantrone
3. EFS of whole cohort, by randomisation 1 and by pre-treatment and post treatment risk stratification and by participating centre

8.2 Secondary Objectives

1. OS of whole cohort, by randomisation, by risk stratification and by participating centre
2. EFS and OS of prognostic subgroups
3. Toxicity of each block of therapy for the whole cohort by phases of therapy, risk stratification and by participating centres

9. Randomisation

Informed consent for participation in the randomised parts of the study is given separately from the consent to the enrolment into the study. This enables the patients and parents (or persons entitled to custody) to have the choice to participate in the randomisation, or not to be randomised.

Non-randomised patients will be treated as specified for the control arm but their data will not be used for the main analysis. After consent has been given and the patient has been randomised, consent can be withdrawn at any time. Randomisation occurs when the diagnostic findings required for inclusion/exclusion criteria and randomisation stratification are complete and the informed consent for randomisation has been obtained. Randomisation sequence was created using statistical software and was stratified by center with a 1:1 allocation using concealed randomisation, performed centrally by the internet-based data entry system. The first randomisation of arm A versus B, is performed by day 8 when the first risk stratification is known. The second randomisation is performed prior the delayed intensification phase.

10. Sample Size Calculation

Though the trial opened at the end of October 2016, all centres have been open only since March 2018. As of May 2019, 1347, patients have been recruited to the trial of which, 727 were recruited between Apr 2018 and March 2019. Thus we expect that by end of March 2021 we will have recruited around 2,800 patients.

Randomisation 1 (R1)

Induction deaths in patients with ALL in India at the respective trial centres have been reported at 10-20%. Serious bacterial infections have been reported in 60-70% and a high incidence of fungal infections (19 of 22 isolates) was also reported in induction. No data is available on other toxicities. The primary association with infection is the severity and duration of severe neutropaenia (ANC <0.5 x 10^9/L) and prolonged steroid therapy. In the ICiCLe study, SR patients do not receive daunorubicin as this is associated with prolonged myelosuppression. IR patients receive 2 doses and HR/T 4 doses of daunorubicin. These are non-randomised interventions. The study is investigating a shortened interrupted steroid randomisation in induction. The steroid pulses are phased to coincide with L-Asparaginase administration to decrease the incidence of allergic reactions.

Not all patients recruited to the study are eligible for the steroid (prednisolone) randomisation. T-ALL patients receive dexamethasone and HR BCP-ALL patients receive the full 5-weeks. The IR group contains children aged over 10-years. Older children have increased toxicity to steroids and they will only receive the shorter regimen. Thus, only SR and IR patients < 10-years of age are eligible for the first randomisation. In the pre-trial cohort, 850 patients were SR and 576 were IR and aged between 1-9 years. Thus around 50% (1426/2680 = 53%) of the study population will be eligible for the R1 randomisation. The projected number in R1 randomisation for the duration of the trial is 1400 or 700 in each arm.

Statistical plan v1 (October 2013) assumed a total patient recruitment of 2400. The sample size calculation was based on an annual recruitment of 750 patients and >95% randomisation. With 1120 patients in each arm, it was powered to detect a 3% difference between the two arms at 80% power and a p = 0.05. However, the plan was based on the magnitude of difference that could be detected based on the presumed sample size rather than the actual toxicities that could be measured and also what would be considered to be a significant difference clinically.

No toxicity data was collected in the pre-trial cohort, but this data is now available in the trial cohort. In induction, of the 419 SR and IR patients eligible for randomisation, 146 (35%) had grade 3-5 sepsis. Hypertension was reported in 36 (8.6%). The incidence of other toxicities attributable to steroid therapy are <2%. Assuming that sepsis occurs at 40% in the continuous steroid group and at 30% in the discontinuous steroid group, we expect 280 grade 3-4 sepsis in the continuous steroid arm and 252 in the randomised arm. To detect a 10% difference between the two groups at a power of 80% and a p-value of 0.05, we need 353 patients in each arm. At a 7% difference, i.e. 260 events in the randomised arm, a sample size of 739 patients are required for each arm. Therefore the study is powered to detect a difference between 7-10%. Increased sepsis rates are expected in the IR arm as these patients receive daunorubicin. Separate sub cohort analyses will be performed to explore the differences in events in the SR and IR patient as secondary analyses. Differences in all other toxicities will be descriptive and differences between the categorical variables will be calculated using the x^2 test.

The secondary objective of this randomisation is EFS and OS. This will be considered to be interpretable when the median follow-up time from first randomisation is at least 40 months and accrual has been completed.

Randomisation 2 (R2)

All patients, irrespective of are eligible for the risk status and first randomisation. However only patients still on trial are eligible for this randomisation. In the pre-Trial cohort, there were 93 deaths in induction, and 83 failed inductions and another 9 deaths prior to the start of delayed intensification. Thus we estimate at least 90% of the trial cohort are eligible for the second randomisation. The end point for this randomisation is the 3-year EFS. We assume that 5% of patients will be lost to follow up and current randomisation acceptance rate for R2 is >95%. Thus assuming that 80% of patients are available for R2, we will have 1120 patients available in each arm if all patients recruited are also randomised to R2. The 3-year estimated EFS for the whole cohort is estimated to be 70% but this may increase to 75%. The sample size calculations can then be based on the following assumptions

Power 80% and p value of 0.05

**Difference in EFS**	**Daunorubicin**	**Mitoxantrone**	**Sample Size**
5%			
	60%	65%	1468
	65%	70%	1373
	70%	75%	1248
6%			
	60%	66%	1012
	65%	71%	945
	70%	76%	856
7%			
	60%	67%	738
	65%	72%	687
	70%	77%	620
10%			
	60%	70%	353
	65%	75%	325
	70%	80%	290

Thus at a power of 80% and a p-value of 0.05, the study is able to detect a 6-10% difference in EFS.

The EFS of the different post induction risk groups i.e SR, IR and HR are expected to be different. It is also possible that other prognostic variables, e.g. age, presenting white cell count, sex and cytogenetic risk groups may show a difference in outcomes. The patterns of relapses may also differ. These will be analysed variables that determine outcome and will also be modelled within this analyses.

11. Data Entry and Data Management

All patient related data will be recorded in the electronic database with a unique trial number (IDM v3.0). This is a web based system allowing real time registration and randomisation and collection of toxicities and events.

11.1 Data collection/Case Report Form (CRF)

All data will be recorded in an electronic case report form (e-CRF). The study software used is based on the TCS IDM platform. The e-CRFs will be filled in by an authorized person (defined in the study team log) as soon as possible. All data will be recorded online. Data will be transferred between the workstation computer at the study site and the study server via a secure connection so that the data cannot be manipulated.

11.2 Source Data and Patient Files

The information in original documents and records (e. g. patient files, laboratory notes) are defined as Source Data and will be reviewed by the Monitor for Source Data Verification.

11.3 Data Processing

Centres will file all data electronically. To verify accuracy of the data, range, validity and consistency checks will be performed automatically by the database. Implausible or missing data can be corrected or added after consulting the Investigator. Documentation for these corrections will be stored with the eCRFs. All validated data will be stored in the database. After termination of the study and after completion of all entries, the database will be closed for further entries. This process will be documented.

11.4 Direct Access to Source Data

According to ICH-GCP the principal investigator must permit all authorized third parties access to the trial site and insight into the medical records of the trial subjects (source data). This permission

includes the clinical trial monitors, auditors and other authorised employees of the sponsor, as well as members of the competent authorities. All these persons are sworn to secrecy.

11.5 Monitoring

A detailed monitor plan will be provided within a monitor manual by the sponsor. The investigators allow the monitor to have access to all of the study materials needed for source data verification and proper review of the study process. All times, the sponsor/investigators/monitors will maintain the confidentiality of the study documents. Furthermore, problems with inconsistent and incomplete data will be discussed. By signing the declaration of informed consent, participants allow access to their documents. With the signature in the protocol, the investigators confirm that auditors and health authority inspectors may have access to the study documentation and accordant medical records. Auditors and inspectors are bound by professional confidentiality and may not pass on any personal information that comes to their knowledge. In the course of audits or inspections, data in the case report forms will be compared with the data for medical record. All the documentation held by the investigators within the scope of the clinical trial, as well as the drug logs of the study medications will be verified.

11.6 Audits/Inspections

Authorized representatives of the Sponsor, a regulatory authority, or an Independent Ethics Committee (IEC) may visit the centre to perform audits or inspections, including source data verification. The purpose of a Sponsor audit or inspections is to systematically and independently examine all the study activities and documents to determine whether these activities were conducted, and data were recorded, analysed, and accurately reported according to the protocol, Good Clinical Practise (GCP), guidelines of the International Conference on Harmonization (ICH), and any applicable regulatory requirements

12. Data and Safety monitoring Committee and Interim Analyses

The study will be monitored by an independent Data and Safety Monitoring Committee (DSMC) that is specifically chosen to include an expert in paediatric oncology, in biostatistics and in clinical trials to ensure utmost competence and vigilance. The DSMC will be constituted before the start of the trial, first DSMC meeting is expected at 1-year recruitment or 100 patients recruited whichever first, then every 6 months during the first 4 years recruitment and annually thereafter in order to review the trial’s progress, safety data (SAEs) and adherence to protocol. DSMC will make

recommendations to the independent Trial Management Group (TMG) who will share the decisions with the main IRB. The main IRB will decide if the trial should continue.

Possible recommendations could include:

- No action needed, trial continues as planned
- Early stopping due, for example, to clear benefit or harm of a treatment, futility, or external evidence
- Stopping recruitment within a subgroup
- Extending recruitment (based on actual control arm response rates being different to predicted rather than on emerging differences) or extending follow-up
- Stopping a single arm of the trial
- Sanctioning and/or proposing protocol changes

During the course of the study one interim analysis after 3-years of recruitment for is planned to exclude significant results of the randomised questions that would require a preterm termination of the trial. The results of this analysis will be discussed with the DSMC and are not planned for general release. The DSMC will recommend discontinuing of the trial in all patients or in selected subgroups only if the result is likely to convince a broad range of clinicians (including participants in the trial) and the general clinical community. The ultimate decision for discontinuation of the trial lies with the IRB (TMC).

The interim analysis will be done by the trial statistician and this analyses will be reviewed by an independent statistician who could be the one within the DSMC.

The open report for the DSMC will have overall results (not split by treatment arm) which will be seen by the CI and with permission from the DSMC can be distributed as a confidential document to the Inter-Group TMG, the funder and the IRB’s.

12.1 Interim Safety Analysis

This analysis will check the safety and tolerability for treatment in the randomised arms. Detailed data on toxicity and proportion of patients experiencing any toxicity will be given overall and by stratification groups. SAEs, deaths and main characteristics of them will be assessed overall and by stratification groups. MRD results and proportion of negative/positive MRD will also be made available. This data will also be analysed by centre. Data will be analysed using using Chi-square or Fisher’s exact test as appropriate.

12.2 Interim efficacy analysis

Efficacy will be investigated after 3-years of recruitment. In 2,800 patients, at a 70% EFS, 840 events are expected to occur at the end of follow up. Interim analyses will be considered once 252 (30%) of events have occurred. EFS will be investigated at this analysis using a Cox analysis of treatment effect on EFS adjusting for the factors used in the randomisation stratification. Consideration will be given to stopping the trial early if the significance level for treatment effect in the Cox analysis is < 0.00034

13. Early stopping rules

This is extant of the interim analyses and is based on the annual report. This is based on treatment related mortality (TRM) and if the TRM exceeds 3 standardised deviations between the two randomised arms. This will be shown by a p-value of 0.001 or less on a two-sided Fisher's exact test TRM will be estimated by all deaths in randomised patients regardless of cause and timing. This is done in order to remove bias introduced by multiple centres classifying deaths as treatment related (or not) and in light of the fact that this guideline will be used early in the trial when the majority of the deaths will be treatment related.

Early stopping is based on the difference in proportion of TRM between the two randomised arms when it exceeds three times the standard deviation (SD) for the overall proportion of TRM based on the expected sample size. However, despite defining the expected boundary sample size and proportion of TRM could be different and boundaries may need to be recalculated.

14. Descriptive Analyses

14.1 The Study Population

The recruitment of patient will be illustrated using CONSORT flow diagram (below). In particular numbers will be presented for all patients registered, showing numbers in each randomised arm, compliant, and analysed with respect to the primary objectives. The number of patients who are ineligible or excluded (failure to satisfy inclusion and exclusion criteria, refusal to participate) or withdrawn from treatment (with reasons) will also be recorded.

CONSORT Diagram

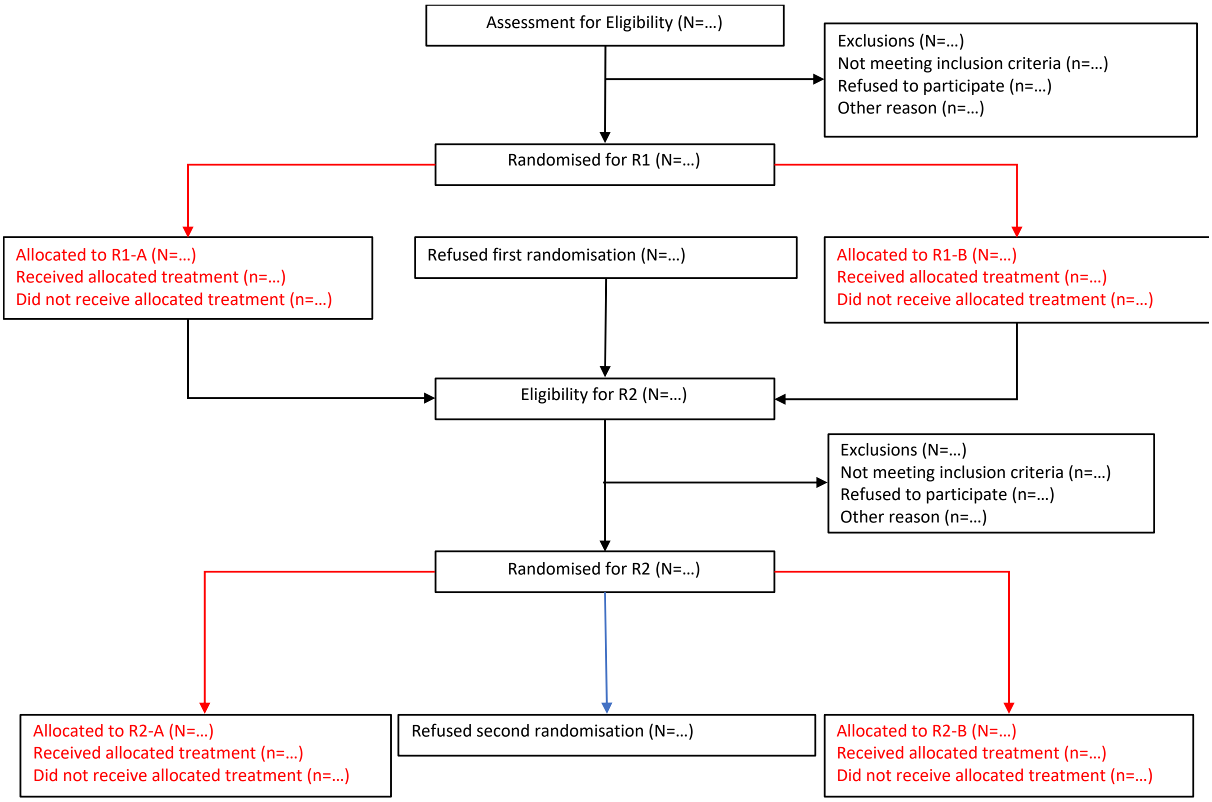

Note: Final analysis needs to also include patients lost to follow up in each arm

A separate table to accompany each section to include reasons for exclusion, e.g. not meeting exclusion criteria; refused to participate; other reasons

14.2 Baseline characteristics

Baseline characteristics will be compared between randomised groups to check that they are similar. Characteristics to be described in tabular form are as follows:

Table 1

Variables used to define risk groups	R1A	R1B	R2A	R2B
Age at diagnosis				
WC at diagnosis				
Sex				
CNS3				
Bulky disease				
T-ALL				
Prednisolone poor response				
Cytogenetics				
CR Rates				
Pre-Treatment risk group				
MRD Good				
MRD Poor				
Post Treatment Risk				

Each of the characteristics shown above will be summarised by showing number and percentage for R-A vs R-B. Numbers (with percentages) for binary and categorical variables and means (and standard deviations), or medians (with lower and upper quartiles) for continuous variables will be presented. The trial statistician, DSMC and TMG will consider if an imbalance is likely to cause concern and will agree an action accordingly. There will be no tests of statistical significance nor confidence intervals for differences between randomised groups on any baseline variable because the treatment arms will be allocated at random, then any difference are due to chance variation and can lead to false assumptions of significant or non-significant differences.

The follow-up will be summarised within each treatment group for each time to event endpoint using the median from the reverse Kaplan-Meier method and the median follow-up for those without an event.

14.3 Comparison of Losses to Follow-up

The numbers and distribution of losses to follow-up (defaulters and withdrawals) over the period of the study will be reported overall and for each randomisation. For each randomisation, analyses

reporting median follow up and number lost to follow up or withdrawn to treatment for patients. For time to event analyses, the baseline characteristics of those lost to follow-up and those to be analysed will be summarised for each randomisation. No test of significance will be used. Those randomised may be balanced, but those with data to analyse on the secondary objectives may not be. The consequences of this examination of the data will be described separately for each objective.

15. Primary Analyses

All validated data will be stored in the database. After termination of the study and after completion of all entries, the database will be closed for further entries. This process will be documented.

15.1 Randomisation 1

The differences in sepsis rates, as a measure of toxicity will will be calculated using the x^2 test. The data will be presented in a tabular form. Increased sepsis rates are expected in the IR arm as these patients receive daunorubicin. Separate sub cohort analyses will be performed to explore the differences in events in the SR and IR patient as secondary analyses. Differences in all other toxicities will be descriptive and differences between the categorical variables using x^2 test or Fishers test as appropriate.

15.2 Randomisation 2

Kaplan-Meier plots will be generated for EFS. A Cox analysis of treatment effect on EFS adjusting for the factors used in the randomisation stratification to remove possible bias between arms due to chance unbalance and to give more statistical power and unbiased results.

15.3 Survival analyses

Kaplan-Meier plots will be generated for EFS. A Cox analysis of treatment effect on EFS adjusting for the factors used for stratification to remove possible bias between arms due to chance unbalance and to give more statistical power and unbiased results.

A Kaplan-Meier plot will be drawn with a line for each treatment combination in the randomised groups to look for a visual sign of interaction between the treatments. An interaction term will also be added to the Cox models containing both of the treatment main effect to test for significance (although this will have low power). If statistically significant interactions are found, the results will also be presented for R2 within each R1 treatment group.

Predefined subgroup analyses of EFS will include all variables listed in Table 1.

Regression analysis will be multivariate regression, including as covariates the factors used in stratification (risk group), and other factors expected to be prognostic (cytogenetics, age, sex and MRD). Only randomised patients will be used for this analysis.

15.3 Missing Data

In randomisation 1, data is collected from the first 5 weeks of therapy. Given that analyses is done at the end of treatment, we anticipate that data will be available on most patients. Randomisation 2 is a time to event analyses. An annual follow up form is part of the study and data should be available on most patients except those lost to follow up.

The numbers and distribution of losses to follow-up (defaulters and withdrawals) over the period of the study will be reported overall and for each randomisation. For each randomisation, analyses reporting median follow up and number lost to follow up or withdrawn to treatment for patients.

For time to event analyses, the baseline characteristics of those lost to follow-up and those to be analysed will be summarised for each randomisation. No test of significance will be used. Those randomised may be balanced, but those with data to analyse on the secondary objectives may not be. The consequences of this examination of the data will be described separately for each objective.

16. Secondary Analyses

Kaplan-Meier plots will be generated for OS whole cohort, by randomisation, by risk stratification, by participating centre and prognostic subgroups.

Toxicity of each phase of the study will be tabulated and presented as percentage of each toxicity occurring per number of patients in one defined phase of treatment. Multiple reports of toxicity in one phase in the same patient will be counted as a single event. Comparison of incidences of SAE, toxicities, treatment related deaths and second malignancy between groups using x^2 test or Fisher's exact test as appropriate. between A p-value adjustment for multiple testing will be made using Bonferroni corrections to protect against making false positive conclusions.

CR and MRD rates, adjusted for factors contributing to risk stratification in the randomised groups will be tabulated and differences analysed using Mann-Whitney or ANOVA as appropriate.

17. Reporting of adverse events

Serious adverse events (SAE) are defined in the protocol. Febrile neutropenia is not classified as an SAE but reported as an adverse event (AE) or toxicity.

Appendix 3: Intravenous MTX, Interim Maintenance

1. This treatment phase runs for 56 days.
2. Blood counts prior to start: ANC > 750/mm3 and platelet count > 75,000/mm^3.
3. Withhold all medications if any serious infection or inter-current illness occurs.Do not catch up if doses are missed.
4. Start IV Methotrexate at 100mg/m2.
a. Escalate dose by 50mg/m2 at 10-day intervals.
b. Evaluate clinical status, blood counts, liver and renal functions prior to dosing.

5. **Withhold** IV Methotrexate if
a. Grade 3 oral mucositis (multiple oral ulcers, only able to tolerate liquids) or higher
i. Withhold until resolved; consider herpes simplex infection.
ii. Resume at 50% ofprevious dose
iii. Escalate to 75% of previous dose and monitor for Grade 3 oromucosal toxicity .
iv. If tolerated, escalate 10 days later to 100% of previous dose .

b. Significant liver toxicity. (overt jaundice and/or serum bilirubin > 3mg/dL) .
i. Withhold untilserum bilirubin < 1.2 mg/dL & resume at 50% of previous dose.
ii. Escalate to 100% of previous dose 10 days later monitor serum bilirubin.
iii. Ignoreraised liver transaminases.

c. Cytopenias (ANC < 750/mm3 and/or platelet count < 75,000/mm^3)
i. Withhold until count recovery & resume at 75% of previous dose when counts recover.
ii. Escalate by 50mg/m2 at 10-day intervals as tolerated.
iii. If no count recovery within 21 days, consider bone marrow studies.

d. Significant renal toxicity (serum creatinine > 3 baseline or > 4mg/dL).
i. Withhold until complete recovery (serum creatinine back to baseline).
ii. Resume at 100% of previous dose.
iii. Escalate dose by 50mg/m2 at 10-day intervals while monitoring renal function.

6. **Reduce** IV Methotrexate if
a. Grade 2 oral mucositis (few oral ulcers, able to tolerate solids) for ≥3 days
i. Reduce to 50% of previous dose
ii. Escalate by 50mg/$m^2$for subsequesnt dose and peoceed as tolerated

b. Mild liver toxicity (serum bilirubin between 1.2 mg/dL and 3 mg/dL)
i. Reduce to 50% of previous dose
ii. Escalate to 100% of previous dose while monitoring serum bilirubin level

Appendix 4: Administration of High Dose Methotrexate

Introduction

The use of high doses of Methotrexate (HD-MTX) may be an advantage in lymphoblastic malignancy for two reasons: first, it may lead to increased uptake of Methotrexate in resistant lymphoblasts and second, it may lead to increased penetration of the drug to "sanctuary sites" such as the CNS and testis. However the administration of HD-MTX carries significant toxicity. This can largely be reversed by effective folinic acid rescue within 42 hours of exposure. Suboptimal rescue can lead to severe irreversible methotrexate toxicity. Consequently, rigorous attention to hydration, alkalinisation, folinic acid rescue and serum methotrexate level monitoring are integral to the safe use of high dose methotrexate.

Pre-therapy checks

1. Check complete blood counts, serum creatinine and liver function tests
2. Withhold treatment if
   (a) ANC < 750/mm^3 and/or platelet count < 100 000/mm^3
   (b) Serum creatinine is $\geq$ 1.5 $\times$ baseline
   (c) Serum bilirubin $\geq$ 2mg/dL
   (d) Liver transaminases $\geq$ 10 $\times$ baseline

Alkaline hyperhydration

1. Commence alkaline hyperhydration at least 6 hours prior to start of HD-MTX.
2. Hydration fluid: 0.45% Sodium chloride; 5% Dextrose solution containing 8.4% Sodium bicarbonate 25mmoL and Potassium chloride 10mmoL in every 0.5L solution. Alternatively the sodium bicarbonate solution can be Y-sited to the hydration fluid.
3. Infusion rate: 125mL/m^2/hour (3 L/m^2/day)
4. Adjust the sodium bicarbonate concentration to maintain the urinary pH between 7 and 8
5. Do not start the HD-MTX infusion until a urinary pH of at least 7 has been achieved

Methotrexate infusion

1. *Pre-B ALL*: Dilute the Methotrexate in 0.9% Sodium chloride or 5% Dextrose. Infuse 0.3grams/m^2 of Methotrexate over 30 minutes, followed by infusion of 2.7grams/m^2 of Methotrexate over 23.5 hours.
   Note: Even if the infusion is not complete at this time point, it must be stopped.
2. *Pre-T ALL*: Dilute the Methotrexate in 0.9% Sodium chloride or 5% Dextrose. Infuse 0.5 grams/m^2 of Methotrexate over 30 minutes, followed by infusion of 4.5 grams/m^2 of Methotrexate over 23.5 hours.
   Note: Even if the infusion is not complete at this time point, it must be stopped.
3. *Hydration during methotrexate infusion*: Continue a parallel infusion of 0.45% Sodium chloride 5% Dextrose solution with 8.4% Sodium bicarbonate (25 mmoL/0.5L) and Potassium chloride (10mmoL/0.5L) at 125mL/m^2/hour

Folinic acid rescue

1. It is important to note that for this schedule, the Folinic acid rescue starts very late (at 42 hours from start of infusion of HD-MTX) and is short (3 doses in cases of normal Methotrexate excretion). Therefore the Folinic acid should be administered intravenously, so that the rescue is optimal.
2. Methotrexate plasma concentrations are measured at 24 and 42 hours from start of the HD-MTX infusion.
3. Additional plasma levels measurements are required if impaired Methotrexate excretion is expected or observed (see Table below)
4. If the 24 hour plasma Methotrexate level is <150µmol/L, then Folinic acid rescue begins 42 hours from the start of methotrexate infusion with the administration of a dose of 15mg/m^{2}Folinic acid.
5. If the 42 hour level is ≤1µmol/L, then 15mg/m^{2}Folinic acid should be given at 42, 48 and 54 hours only (total, 3 doses) as shown in the Table below. No further folinic acid is required and no further levels are required.
6. Maintain alkaline hyperhydration until completion of Folinic acid rescue.
7. The Table below outlines measures to be taken in patients with delayed Methotrexate excretion (with or without accompanying rise in serum creatinine and/or fall in urine output).
   (a) If the 24 hour Methotrexate level exceeds 150µmol/L or the creatinine increases to more than 1.25 × the baseline, increase hydration to 200mL/m^2/hour and keep urine pH >7.0. Estimate urine output every 4 hours and if urine output is less than 80% of fluid intake, considerGlucarpidase. Recheck the plasma Methotrexate level at 36 hours.
   (b) If the 36 hour level exceeds 10µmol/L, consider Glucarpidase regardless of urine output.
   Ifthe 36 hour level is however<10µmol/L, continue hydration at 200ml/m^2/hour (keeping urine pH >7.0). Estimate urine output every 4 hours and if urine output is less than 80% of fluid intake, considerGlucarpidase. Recheck the plasma Methotrexate level at 42 hours.
   (c) If the plasma Methotrexate concentration at hours 42 and 48 from the start of HD-MTX infusion exceeds the normal range for time, increase the Folinic acid rescue according to the schedule provided in the Table below.
   (d) If the plasma Methotrexate concentration at 36 hours was high but the patient then “catches up” and the level falls to ≤1µmol/L at 42 hours, then administer standard Folinic acid rescue as long as the urine output is satisfactory.

Intrathecal Methotrexate during the HD-MTX schedule

1. Intrathecal Methotrexate is to be administered *synchronous* with the intravenous Methotrexate infusion.
2. Do not interrupt the intravenous Methotrexate infusion when Intrathecal Methotrexate is being administered

Cotrimoxazole prophylaxis

1. Omit on all High dose Methotrexate treatment weeks
2. Administer on weeks between High dose Methotrexate treatments

Table: RESCUE SCHEDULE WITH HIGH-DOSE METHOTREXATE (HD-MTX)

Time[a]	Serum MTX (µmol/L)	Hydration[b] (mL/m²/hr)	Folinic acid rescue	Glucarpidase[c]	Monitoring
**T24**	< 150	125	15 mg/m², T42, T48, T54	No	• Serum creatinine<br>• Next MTX level at T42<br>• Urine output &urine pH
	≥ 150[d]	200[e]	15 mg/m², T42, T48, T54	Yes,only if oliguria[f]	• Serum creatinine<br>• Next MTX level at T36<br>• Urine output &urine pH
**T36**[g]	< 10	200[e]	15 mg/m², T42, T48, T54	Yes,only if oliguria[f]	• Serum creatinine<br>• Next MTX level at T42<br>• Urine output &urine pH
	≥ 10	200[e]	15 mg/m², T42, T48, T54	**Yes**[h]	• Serum creatinine<br>• Next MTX level at T42<br>• Urine output &urine pH
**T42**	≤ 1	125	15 mg/m², T42, T48, T54	No	• Urine output &urine pH<br>• No further MTX levels[i]
	> 1 - < 10	125	15 mg/m² at T42 &**every6 hrs**	No	• Serum creatinine<br>• MTX level every 12h until level < 0.1 µmol/L<br>• Urine output &urine pH
	≥ 10 - < 40	125	15 mg/m² at T42 &**every 3 hrs**	Consider	• Serum creatinine<br>• MTX level every 6h until level < 0.1 µmol/L<br>• Urine output &urine pH
	≥ 40 - < 200	125	100 mg/m² at T42 &**every 6 hrs**	Consider	• Serum creatinine<br>• MTX level every 6h until level < 0.1 µmol/L<br>• Urine output &urine pH
	≥ 200	125	1000 mg/m² at T42&**every 6 hrs**	Consider	• Serum creatinine<br>• MTX level every 6h until level < 0.1 µmol/L<br>• Urine output &urine pH

[a] time from commencement of HD-MTX infusion (e.g. T24 = 24 hours from commencement of HD-MTX)

[b] 0.45% Sodium chloride5% Dextrose with 25mmoL 8.4% Sodium bicarbonate& 10 mmoLKCl in every 500mL (ensure urine pH > 7.0)

[c] also known as Carboxypeptidase G2; 50 IU/kg, slow IV bolus (5 min); withhold Folinic acid 1 hour prior and 4 hours post injection; occasionally, 2 doses are required

[d] and/or serum creatinine rises to 1·25 × baseline

[e] ensure urine pH > 7.0

[f] oliguria when urine output < 80% of fluid intake (urine output checked every 4 hours)

[g] only tested if T24 MTX level ≥ 150 μmol/L

[h]Glucarpidase administered irrespective of urine output

[i] no further plasma MTX level estimation unless decrease in urine output is observed

N.B. No penicillin derivative should be used with High Dose Methotrexate

Appendix5:DosingRecommendationsForErwiniaAsparaginase

**E. coli L-Asparaginase**	**Erwinia Asparaginase (Erwinase)***
Induction (standard risk pre-B ALL only)	
Native E. *coli* L-asparaginase 10,000 U/m^2 on Days 18; 21; 24; 27<br>OR<br>PEG Asparaginase 1,000 U/m^2 on Day 16	Erwiniaasparaginase 20,000 U/m^2 on Days 18; 20; 22; 24; 26; 28(total, 6 doses)
Induction (all risk groups except standard-risk pre-B ALL)	
Native E. *coli* L-asparaginase 10,000 U/m^2 on Days 9; 12; 15; 18; 21; 24; 27; 30<br>OR<br>PEG Asparaginase 1,000 U/m^2 on<br>Day 9 and Day 23	Erwiniaasparaginase 20,000 U/m^2 on Days 9; 11; 13; 15; 17; 19; 21; 23; 25; 27; 29; 31<br>(total, 12 doses)
Augmented Consolidation (high risk pre-B and high risk pre-T disease only)	
Native E. *coli* L-asparaginase 10,000 U/m^2 on Days 15; 18; 21; 24; and<br>Days 43; 46; 49; 52<br>OR<br>PEG Asparaginase 1,000 U/m^2 on<br>Day 16 and Day 44	Erwiniaasparaginase 20,000 U/m^2 on Days 15; 17; 19; 21; 23; 25; and<br>Days 43; 45; 47; 49; 51; 53<br>(total, 12 doses)
Delayed Intensification (all risk groups)	
Native E. *coli* L-asparaginase 10,000 U/m^2 on Days 4; 7; 10; 13<br>OR<br>PEG Asparaginase 1,000 U/m^2 on Day 4	Erwiniaasparaginase 20,000 U/m^2 on Days 4; 6; 8; 10; 12; 14<br>(total, 6 doses)

* Dose rounded off to the nearest 500 U

Appendix 6:Recommendations for Cerebrospinal Fluid analysis

Sample

1. Cerebrospinal fluid (CSF) will be collected in two labelled containers (a) one, an EDTA microtainer, for CSF cell count and (b) the other, a plain microtainer, for CSF cytospin examination. Where feasible, CSF is collected in an additional plain microtainer for analysis of glucose and protein levels.
2. The recommended volume is 1 mL of CSF in each container (1mL is equivalent to 16 drops of CSF when using the standard 25G or 23G spinal needle).

Processing time

The CSF cell count drops rapidly after 1 hour of collection. CSF samples are therefore to be processed within 1 hour of collection. Samples must be maintained at room temperature and transported as soon as feasible to the laboratory following collection.

Processing

Macroscopic examination of CSF

A note is made whether the fluid is clear, xanthocromic, haemorrhagic or turbid.

Cell count

Stained cell count

A 1:1 solution of CSF (100 μL) and WBC diluting fluid (100 μL) is prepared. The WBC diluting fluid (Turk's solution) is a solution of methylene blue (30mg/mL) in glacial acetic acid and distilled water. The Neubauer chamber is loaded on both sides with this solution and the coverslip is gently placed. The loaded Neubauer chamber is then kept in ahumidified chamber (e.g. a petridish with water) for 30 seconds for the cells to settle down. Cells are counted in all 9 squares and the WBC count is calculated using the following formula:

Cells /μL = (Average cell count in each square) $\times$ dilution (in this case, 2) $\times$ 10

Unstained cell count

To determine the CSF RBC count, unstained CSF is loaded onto a Neubauer chamber and red blood cells are counted using the technique described above.

Preparation of CSF cytospin

- At least 2 cytospin slides are prepared for each CSF sample.
- A sandwich of glass microscope slide, filter paper and cytospin funnel is created. This sandwich is placed in a slide holder and is secured with the retaining clips of the slide holder.
- The above assembly is placed in the cytospin chamber of the cytocentrifuge apparatus (Shandon or Wescor).
- Two hundred microliters of CSF is loaded in the cytospin funnel (maximum, 500μL). Recommended centrifuge settings: 800 rpm for 3 minutes (or 500 rpm for 4 minutes), low acceleration.
- Following completion of the spin, the sandwich is carefully disassembled, taking care not to disturb the cell deposit on the glass slide. The cell deposit is encircled using a permanent marker or a diamond-tipped glass marking pen.
- Cytospins are air-dried and stained immediately; unfixed cytospinsmay be stored for no longer than 2 days at room temperature
- Staining is performed using the Haemacolor staining kit (Merck Millipore). Hemacolor solution 1

is a fixing solution, Hemacolor solution 2 is a red staining solution and Hemacolor solution 3 is a blue staining solution. Air-dried cytospins are sequentially dipped in Coplin jars containing Hemacolorsolution 1 (1 dip), Hemacolor solution 2 (2 dips), Hemacolor solution 3 (3 dips) and Buffer solution, pH 7.2 (2 × 3 dips). Stained cytospins are then air-dried and examined under a microscope.

- Normal cellular sediments include occasional lymphocytes and monocytes. Excess lymphocytes and rare ependymal cells are sometimes observed after intrathecal therapy.

Classification of CNS leukaemia based on CSF analysis

CNS category	RBCs/μL	WBCs /μL	ALL blasts	Signs of CNS disease
1	< 10	< 5	Absent	Absent
2a	< 10	< 5	Detected	Absent
2b	≥ 10	< 5	Detected	Absent
2c	≥ 10	≥ 5*	Detected	Absent
3a	< 10	≥ 5	Detected	Present / Absent
3b	Any	< 5	Not detected	Present

* Ratio of CSF WBC / CSF RBC count is less than 2× ratio of blood WBC / blood RBC count (Steinherz-Bleyer algorithm)

Appendix 7: MRD

MRD reporting

1.Reported as High, Low or Indeterminate.

2.Indeterminate MRD includes samples with inadequate cellularity (i.e. less than 100,000 mononuclear cells)

3.MRD will be reported at week 5 for BCP-ALL and week 9 for T-ALL. Risk stratification based on MRD is for BCP-ALL in SR and IR categories.

Flow cytometry MRD

Two tubes at diagnosis to determine the LAIP markers for flow cytometry MRD tracking

Time points

The time points for MRD will be from bone marrow aspirates at diagnosis and week 5 for BCP-ALL. The time points for T-cell ALL will be at diagnosis and week 9.

Appendix 8: Cytogenetics

Recommendations for Cytogenetic analysis

Cytogenetic risk stratification

In ICiCLe ALL-14, results of cytogenetic testing (karyotyping and FISH) will be used to stratify patients with B-cell precursor ALL (BCP-ALL) into two risk groups. This includes

- Standard Risk disease
  - High hyperdiploidy (modal chromosome number 51 – 67)
  - ETV6/RUNX1 fusion translocation [t(12;21)(p13; q22)]
- High risk disease
  - MLL gene rearrangement,
  - Ph+ ALL [t(9;22) (q34; q11); BCR/ABL1]
  - Intrachromosomal amplification of chromosome 21 (iAMP21)
  - Hypodiploidy (less than 40 chromosomes)[4, 5]
  - E2A/HLF fusion translocation [t(17;19)(q22;p13)]

Test centres

Cytogenetic analyses will be performed at the respective regional referral laboratories.

Samples

1. Studies will be performed on pre-treatment bone marrow samples.
   - Where diagnostic bone marrow is not available, pre-treatment peripheral blood or early post-treatment bone marrow (≤ 8 days from initiation of therapy) can be used for cytogenetic studies.
2. Samples will be collected in labelled sterile heparin tubes.
   - Samples must be maintained at room temperature and transported as soon as feasible to the laboratory following collection.
     Note: Live cells are required for cytogenetic preparations. Samples must therefore never be frozen.
   - Unstained or stained bone marrow aspirate smears may be used for interphase FISH studies
3. A combination of conventional karyotyping and fluorescent in situ hybridisation (FISH) is recommended.
   - Testing with reverse transcription PCR (RT-PCR) to identify recurrent fusion transcripts associated with ALL is not recommended. RT-PCR will not identify aneuploidies and non-AF4 MLL translocations.
   - In some centres, DNA index, determined by flow cytometry, will be used to identify patients with high hyperdiploidy (DNA index ≥ 1.16) and hypodiploidy (DNA index < 0.86)

Cytogenetic studies

FISH

Briefly, the steps involved in FISH labelling and analysis are as follows

- Analysis is performed using fixed cell suspensions obtained by direct or unstimulated overnight cultures of peripher al blood or bone marrow using standard protocols.1
- Approximately 500µl of the sample is suspended in either phosphate buffer saline (interphase FISH) or RPMI growth medium (metaphase FISH). Cells are subsequently harvested and slides prepared.
- DNA denaturation is performed and fluorescent DNA probes are allowed to hybridise to target genomic regions. Following overnight hybridisation, slides are washed to removeunhybridised probe.
- DAPI is appliedas nuclear counterstain and slides are examined under a fluorescent microscope. At least 200 interphase cells and any available metaphases will be analysed from each sample at 1000 × magnification.
- Results will be reported using the International System for Human Cytogenetic Nomenclature (ISCN) 20132.
- In the case of stained smears, additional steps include the following:
    - An area with uniform distribution of cells is identified.
    - Slides are treated with Xylene and Ethanol and destained using Carnoy's fixative.
    - The treated slides are air dried and a similar procedure of probe hybridisation and post-hybridisation wash is followed.

FISH testing process

All patient samples will undergo FISH analysis using the standard ETV6/RUNX1, BCR/ABL1 and TCF3/HLFfusion probes and the MLL break-apart probe. This FISH panel will be able to define most of the prognostically-relevant cytogenetic categories. The ETV6/RUNX1 probe providesadditional information about the presence of intrachromosomal amplification of chromosome 21 (iAMP21) and the presence of hyperdiploidy. Centers not performing karyotyping will confirm the presence of hyperdiploidy by using additional centromeric probes for chromosomes 4, 10 and 21.

Conventional karyotyping

Samples will be processed using standard cytogenetic protocols3.

- For each sample two overnight cultures will be set up (with and without colcemid).
- The sample cell count will be determined and appropriate amount of sample will be added to the enriched media to obtain a concentration of 1.5 × 106cells/mL.
- Cells will be harvested following overnight incubation. Standard cell harvesting steps include induction of mitotic arrest (by addition of colcemid), hypotonic treatment and fixation. Slides will be prepared to obtain well-spread metaphases.
- The slides will be labelled by G banding, involving trypsin digestion and Giemsa staining (GTG banding).
- Slides prepared from both culture tubes will be screened andimages captured of between 15 and 20 representative metaphases. Analysis will be performed using an automated karyotyping application.
- Karyotypes will be reported in accordance with the International System for Human Cytogenetic

Nomenclature (ISCN) 2013.

Cytogenetic Reporting

- A normal karyotype will be assigned when no clonal abnormalities are seen in 20 metaphases derived from two different cultures.
- A diagnosis of clonal structural abnormality and trisomy of a chromosome is assigned when at least two metaphasesdemonstrate the abnormality.
- Diagnosis of monosomy of a chromosome is assigned when at least three metaphases have the abnormality.

References

1. Knoll, J. H. M. &Lichter, P. In Situ Hybridization to Metaphase Chromosomes and Interphase Nuclei. Current Protocols in Human Genetics (2005).
2. Schaffer LG, Jordan JM, Schmid M. ISCN 2013: an international system for human cytogenetic nomenclature. Basel: Karger; 2013.
3. Korf BR. Overview of clinical cytogenetics. CurrProtoc Hum Genet 2001Ch. 8: Unit 8.1.
4. Safavi S and Paulsson K. Near-haploid and low-hypodiploid acute lymphoblastic leukemia: two distinct subtypes with consistently poor prognosis. Blood. 2017;129:420-423
5. Harrison CJ1, Moorman AV, Broadfield ZJ, Cheung KL, Harris RL, Reza Jalali G, Robinson HM, Barber KE, Richards SM, Mitchell CD, Eden TO, Hann IM, Hill FG, Kinsey SE, Gibson BE, Lilleyman J, Vora A, Goldstone AH, Franklin IM, Durrant J, Martineau M; Childhood and Adult Leukaemia Working Parties.

Appendix 9: Definition of Adverse Events

Adverse Event

Any untoward medical occurrence in a patient or clinical study subject administered a medicinal product and which does not necessarily have a casual relationship with this treatment.

***Comment*:**

An **AE** can therefore be any unfavourable and unintended sign (including abnormal laboratory findings), symptom or disease temporarily associated with the use of an investigational medicinal product, whether or not related to the investigational medicinal product.

Serious Adverse Event

Any untoward medical occurrence or effect, that at any dose:

- Results in death unrelated to original cancer
- Is life-threatening*
- Results in persistent or significant disability or incapacity
- Or is otherwise considered medically significant by the Investigator***

Comments:

The term severe is often used to describe the intensity (severity) of a specific event. This is not the same as serious, which is based on patients/event outcome or action criteria.

*Life threatening in the definition of an **SAE** refers to an event in which the patient was at risk of death at the time of the event; it does not refer to an event that hypothetically might have caused death if it were more severe.

Hospitalisation is defined as an unplanned, formal inpatient admission, even if the hospitalisation is a precautionary measure for continued observation. Thus, hospitalisation for protocol treatment (e.g. line insertion), elective procedures (unless brought forward because of worsening symptoms) or for social reasons (e.g. respite care) are not regarded as an **SAE.

***Medical judgement should be exercised in deciding whether an **AE** is serious in other situations. Important **AE's** that are not immediately life threatening or do not result in death or hospitalisation but may jeopardise the subject or may require intervention to prevent one of the other outcomes listed in the definition above, should be considered serious.

<u>Suspected Unexpected Serious Adverse Reaction</u>

A **SAR** that is unexpected i.e. the nature, or severity of the event is not consistent with the applicable product information.

A **SUSAR** should meet the definition of an **AR, UAR** and **SAR.**

<u>Unexpected Adverse Reaction</u>

An **AR**, the nature or severity of which is not consistent with the applicable product information.

When the outcome of an **AR** is not consistent with the applicable product information the **AR** should be considered unexpected.

Common Toxicity Criteria Gradings

Toxicities will be recorded according to the ***Common Terminology Criteria for Adverse Events (CTCAE)*** version 4.0. The full CTCAE document is available on the National Cancer Institute (NCI) website.

All Serious Adverse Events need to be reported via the clinical study management system within 24 hours of the event being confirmed. These will be reviewed by the Clinical Study Manager and Study Coordinators who may request additional information.

A further report needs to be filed as to the date of termination of the event.

Toxicity data will be collected at the end of each individual phase of treatment, i.e. induction, consolidation, interim maintenance and delayed intensification and every 3 month cycle of maintenance. Only CTCAE grade 3-4 are to be reported and unless falling into the SAE criteria, haematological toxicity does not need to be reported.

Each phase will also collect data on the number of blood and platelet transfusions received.

Appendix 10: Philadelphia Positive (Ph+) Acute Lymphoblastic Leukaemia/BCR-ABL

Identification: FISH with the BCR-ABL probe and/or RT-PCR for the BCR-ABL fusion gene.

Stratification: High risk

Additional Therapy: All Ph+ ALL patients will receive Imatinib 360mg/m^2 daily from cytogenetic identification. . Imatinib will be continued till the end of last Maintenance phase.

Cytopenias anticipated with TKI during the maintenance phase may require treatment interruption followed by sequential reintroduction of TKI and antimetabolites. Monitoring of response is by prednisolone response and flow cytometry MRD. Serial monitoring of BCR-ABL transcript will be a secondary response indicator.

Recommendation: It is recommended to track disease levels using either flow MRD or PCR in these patients. An allogeneic stem cell transplant is recommended where possible for those who have disease levels ≥10-4 at the end of Interim Maintenance.

Appendix 11: Thrombo-embolic events

Background

Thrombosis is a recognised complication of the treatment and management of ALL. The true prevalence in this patient population is unknown and varies with the method of assessment. The PARKAA study (Cancer 2003, Vol.97, 2) reported a prevalence of symptomatic events of 5% and asymptomatic events of 36.5% in children undergoing induction chemotherapy for ALL with a central venous catheter insitu. Asymptomatic events were diagnosed by screening with bilateral venography or MRI. Whilst the authors recommend carefully designed clinical trials of primary prophylaxis for the prevention of TE's in this patient population, there is at present insufficient data from children treated on UK protocols to support this. However, the need to collect such information to judge the appropriateness of prophylaxis is recognised. In general, primary prophylaxis for children with CVL cannot be recommended at this time, because there is no evidence for the efficacy or safety of this approach.

Asparaginase therapy and the presence of a central venous catheter are accepted as the main predisposing factors. The literature on the role of inherited thrombophilia in predisposing to thrombotic events in these children is conflicting, with the BFM reporting a significant association, and the Canadians and other major groups, no association. It is, therefore, considered premature to recommend universal screening or primary prophylaxis, although it may be prudent to try to identify less common high-risk abnormalities e.g. AT deficiency, PC deficiency.

Screening

1. Universal thrombophilia screening is not recommended.
2. A careful family history of thrombosis should be taken.Children with a first or second degree relative with Protein Cor A.T. deficiency should be screened for the relevant deficiency, as prophylaxismaybe indicated.The literature is inconclusive for risk conferred by other inherited thrombophilias.
3. Teenage girls on the combined oral contraceptive pill should stop the pill and change to a low dose progesterone only preparation, or norethisterone.

Catheter (CVL) Related Thrombosis

Loss of CVL patency

Inability to withdraw blood with or without inability or impaired ability to infuse. If there is no evidence to suggest displacement of the catheter tip and there are no signs to suggest the presence of an occlusive thrombus, proceed with urokinase lock:

Urokinase 2,500 units each lumen for 2-4 hours.

Failure to restore patency or recurrent loss of patency

Perform a chest roentgenogram to check the position of the catheter tip. A linogram will be required to assess CVL patency. If the linogram demonstrates the presence of a fibrin sheath with no evidence of significant clot formation around the tip, proceed to urokinase infusion.

Urokinase 150 units/kg/hr via each lumen for 12-24 hours.

Monitor coagulation prior to and every 8 hours during infusion

Low dose Alteplase (0.1mg/kg/hr for 4 – 6 hrs) may be considered as an alternative thrombolytic agent.
Repeat linogram following infusion to confirm resolution. If the linogram demonstrates (or is suspicious of) the presence of significant clot formation around the catheter tip, or of vessel thrombosis, proceed with further imaging studies.
Imaging: consider doppler or MR venography.
Linograms have been shown to be relatively insensitive for the detection of large vessel CVL related thrombosis. In the presence of persistent line dysfunction despite a normal linogram, further imaging is indicated.
Doppler is a sensitive technique for imaging jugular veins, but has poor sensitivity for central intrathoracic veins. MRV is less well evaluated but is likely to provide good sensitivity. Some children are likely to require a GA for this technique

Problem:

1. Doppler or Venography confirms the presence of large vessel thrombosis

2. Clinical symptoms/signs of CVL related thrombosis

Arrange imaging to confirm the presence and extent of thrombosis Imaging: Doppler, venography or MR venography

Treatment:

If the CVL is no longer required or is non-functioning it should be removed. If CVL access is required and the CVL is still functioning then the CVL can remain in situ. Unless otherwise contraindicated, anticoagulant therapy should be commenced. Low molecular weight heparin (LMWH) is probably the anticoagulant of choice for initial therapy in most cases.

LMWH dosing: Enoxaparin (Clexane) 1mg/kg/bd by s/c injection. Monitor using anti-Xa levels taken at 4 hours post dose, therapeutic range 0.5-1.0 iu/ml. (LMWH pharmacokinetics for children have only been established for enoxaparin and reviparin [not available in the UK] but the use of other LMWH may be acceptable with monitoring. For pulmonary thrombus, dalteparin has been recommended.

Prior to a lumbar puncture, or any other invasive procedure, the preceding two doses of LMWH should be omitted.

If there is an occlusive thrombus in a major vessel e.g. IVC, consider local thrombolytic therapy prior to anticoagulation and/or catheter removal. Low-dose Alteplase (0.1mg/kg/hr) may be administered locally via the CVL but higher doses (0.5mg/kg/hr) are required for systemic therapy. Alteplase should be administered for 4 – 6 hours, followed by re-imaging. Maximum dose for Alteplase treatment is 100mg/day.

Following the initial 3 months of therapy for children with a first CVL-related DVT, prophylactic doses of oral anticoagulants (INR 1.5 to 1.8) or LMWH –(anti-factor Xa levels of 0.1-0.3) is an option until the CVL is removed. Children with recurrent CVL related DVT should have prophylactic anticoagulation until the removal of the CVL.

Some children will be scheduled to receive Asparaginase as per protocol having had an earlier catheter-related thrombotic event. Consideration should be given to removal of the CVL but those children receiving Asparaginase with a CVL in-situ should receive prophylactic anticoagulation for the duration of their Asparaginase therapy.

Thrombophilia screening should be performed following completion of anticoagulant therapy and should include Protein C, Protein S, AT, FV Leiden, lupus screen, anticardiolipin antibodies and prothrombin gene 20210A.

CNS Thrombosis

Use of anticoagulants for treatment of the acute phase is contentious. Asparaginase should be suspended from that particular course but can be given in subsequent courses under prophylactic anti-coagulant cover as described above.

Appendix 12: Guidelines for Intrathecal Therapy

Where possible perform spinal taps under sedation or anaesthesia to avoid a traumatic lumbar puncture. Ensure that platelet counts are above 50,000. Local anaesthesia preferably subcutaneous lignocaine is recommended.

Spinal needles: Preferably use 25G bevelled spinal needles with a stylet. Use atraumatic/pencil point needles if you have them.

Recommended length of needle:

Height	*Needle length*
80cm	3cm
120cm	4cm
150cm	5cm
>150cm	6cm

Methotrexate: ***DO NOT KEEP VINCRISTINE IN THE SAME AREA AS INTRATHECAL METHOTREXATE***

Use Methotrexate at a dilution of 2.5mg/ml

Age	*Dose*	*Volume*
<2 years	8mg	3-4mls
2-3 years	10mg	4mls
≥ 3years	12mg	5mls

Procedure: Once cerebrospinal fluid is flowing and has been collected, if necessary, attach chemotherapy syringe to lumbar puncture needle. Inject slowly. Remove syringe attached to needle when administration is complete. Apply pressure with a gauze pad for 1 minute. If there is a leak, either spray up-site or apply a small dressing with gauze and tape. This dressing should be removed within 24 hours.

***Definitions*:**

CNS 1: Absence of blasts in CSF on cytospin

CNS 2: 2a: <10 RBCs; <5 WBC and cytospin positive for blasts

2b: ≥10 RBCs; <5 WBCs and cytospin positive for blasts

2c: ≥10 RBCs; ≥ 5 WBCs and cytospin positive for blasts

CNS 3: 3a. ≥5 WBCs; <10 RBCs, with blasts in the CSF (with or without clinical signs)

3b. Clinical evidence of CNS disease alone (facial nerve palsy, brain, eye involvement, hypothalamic syndrome) with no accompanying CSF findings

If the tap is traumatic, then the steinlez-Bleyer Algorithm can be used.
If CSF WBC/CSF RBC > 2 x Blood WBC/Blood RBC with blasts then the patient qualifies as CNS 3.

Appendix 13: Radiotherapy

Cranial Radiotherapy

Eligibility

Age ≥3 years and CNS Status 3 at day 8. The diagnosis and definition of central nervous system (CNS) disease in leukemia was standardized by the Rome Workshop and currently modified based on disease control in ALL studies through 1990s. CNS 3 status is defined as the presence of $\geq$ 5 WBC/micro liter in the cerebrospinal fluid (CSF) with cytologic or biologic evidence of blasts or the presence of cranial nerve palsies believed to be related to CNS infiltration.

Further Intrathecal Methotrexate is not to be used in these children once radiotherapy is complete.

Timing

Cranial RTwill be administered following completion of the delayed phase of intensification prior to commencement of the first cycle of maintenance.

Technique

The patient is to be immobilized with a thermoplastic mask in the supine position.All patients to undergo computerized tomography (CT) based treatment planning.

A linear accelerator is to be used to deliver cranial irradiation. Lateral opposed fields are to be used to cover all cranial meninges including those surrounding the optic nerve in the retro orbit and it is a standard requirement to include the posterior retina and the orbital apex. Centers could use inferior rotation of the gantry (angling the beam posteriorly for the supine patient) to achieve a parallel anterior margin at the bony orbital rim.

Volume

For cranial irradiation, the target volume includes the entire intracranial subarachnoid space. The most important areas to be adequately covered are at the skull base: the cribriform plate (lowest point of the anterior cranial fossa, located in the midline at a level that is typically below the orbital roof) and the lower limit of the temporal fossa. Bilateral lens may be contoured for dose reporting across centers. It may be a useful practice to contour the cribriform plate to ensure adequate coverage. In order to adequately cover the cribriform plate, one needs to accept a dosage approximating 20% to the lens in certain circumstances. If possible, the dose to the lens should be kept to about 10 – 15% of mid plane prescription dose.

Treatment of additional fields: the use of a nasal electron field to adequately cover the cribriform plate may be used at the discretion of the radiation oncologist. If such a field is used there has to be a clear justification of its use.

Dosage

A midplane dose of 18 Gy 10 fractions over 2 weeks will be used. All fields will be treated daily.

Treatment interruptions

Treatment interruptions during cranial irradiation are best avoided. Radiation treatment need not be withheld due to cytopenias unless the patient is symptomatic or unwell. It is desired that the hemoglobin concentration is kept $\geq$ 10 gm/dl during radiation for optimal radiation effects.

Testicular Radiotherapy

Eligibility
Testicular RT would be administered to all patients with testicular enlargement that persists after completion of the second (i.e. consolidation) phase of chemotherapy.

Timing
Testicular RT would be administered following completion of the delayed phase of intensification prior to commencement of the first cycle of maintenance.Testicular irradiation can be delivered concurrently with cranial irradiation in patients who require both.

Technique
The patient is to be simulated in the supine position. When indicated, irradiation is directed to both testes, usually by an *en face* electron beam, calculating the energy to deliver 90% of the dose to the posterior aspect of the testes based on measurements in the treatment position. Centers may also choose to use low energy photon beams with bolus of adequate thickness to prevent anterior testicular under dosing due to build up in these patients and ensure that 90% of the prescribed dose completely covers the posterior aspect of the testes.

Attention to appropriate daily positioning is of utmost importance to ensure descent of testes into the scrotum during treatment.

Volume
Both testes and spermatic cord to the level of the deep inguinal ring is to be irradiated. Surrounding tissues (including penis) may be shielded or strapped away from the radiation field.

Dosage
A midplane dose of 24 Gy 12 fractions over 2.5 weeks will be used. All fields will be treated daily.

Treatment interruptions
Treatment interruptions during testicular irradiation are best avoided. Radiation treatment need not be withheld due to cytopenias unless the patient is symptomatic or unwell.

Any treatment interruptions longer than 48 hours (excluding weekends) for any site are to be discussed with the radiotherapy coordinator for each center and the reason documented.

<u>Dose reporting and Quality Control for cranial and testicular radiation therapy across centers</u>

All participating centers are requested to report:

- Complete clinical workup and indication to use radiation therapy
- Prescription of treatment to one/ both sites
- Confirm usage of CT based simulation for treatment planning

- Delineation of volumes (lens, cribriform plate, bilateral testes)
- Selection of beam arrangements and energies being used
- Treatment of additional fields if any
- Report maximum lens doses, minimum dose to the cribriform plate and mean testicular dose
- Setup methods at the therapy machine (eg: use of planar imaging)
- Followup

Additional Therapy

It is recommended to avoid concomitant use of thiopurine during this phase of radiotherapy. Patients will receive 5 days of dexamethasone at 6mg/m2 (max daily dose 20 mg) per day orally given in two-three divided doses at the commencement of the radiotherapy block. Maintenance chemotherapy maybe restarted once ANC ≥1000 and Platelets ≥100,000.

Appendix 14: Guidelines for antimetabolite dosing during maintenance for all patients

Dosing modification

Doses of 6-Mercaptopurine and Oral Methotrexate are modified as follows:

Antimetabolite dosing guides: Commence continuation therapy after delayed intensification once neutrophil count is ≥1000/mm^3 and platelet count is ≥100,000/mm^3

1. Start antimetabolites at 100% of prescribed dose
a. Oral 6-Mercaptopurine, 60mg/m^2, daily
b. Oral Methotrexate, 20mg/m^2, weekly

2. Round off doses to the nearest tablet strength
a. Oral 6-Mercaptopurine, to the nearest 12.5 mg (Quarter tablet)
b. Oral Methotrexate, to the nearest 2.5 mg (Quarter tablet)

3.Adjust antimetabolite doses to maintain the following blood counts:
a.Neutrophil count between 750 – 1500/mm^3
b. Platelet count ≥ 75,000/mm^3

4. Antimetabolite doses are not adjusted for
a. Low Haemoglobin
b. Raised liver transaminases

Antimetabolite doses will need to be withheld for cholestasis (raised *conjugated* bilirubin)

5. If the neutrophil count is ≤ 500/mm^3 and/or platelet count is ≤50,000/mm^3
a. STOP 6-Mercaptopurine and Oral Methotrexate
b. Monitor counts weekly
c. Once counts recover to ≥1000/mm^3 and platelet count is ≥75,000/mm^{3}resume at 100% of recommended doses

6. If the neutrophil count is persistently higher than 2000/mm^3 for ≥ four (4) weeks
a. Assess compliance
Besides history, RBC macrocytosis (in non-thalassaemia heterozygotes) and low lymphocyte count (≤ 1.0 × 10^9/L) are useful indicators of compliance
b. If compliance is established and if the platelet count is ≥75,000/mm^3,increase6- MP dose by 25%
c. Review blood counts over the next 4 weeks.
d. If still persistent high neutrophil count (≥2000/mm^3), increase weekly oral Methotrexate dose by 25%
e. Subsequent dose escalations are made every eight (8) weeks using the above approach
f. There are no maximum doses for 6-Mercaptopurine and Methotrexate.

7. In the event of blood counts that remain persistently low (>2weeks) despite interruption of antimetabolites
a. Withhold prophylactic Cotrimoxazole
b. Consider Parvovirus infection
c. Consider marrow relapse of leukaemia

8. In case of persistent thrombocytopenia only, consider the possibility of thiopurine induced veno-occlusive disease.

In no case should children receive Folic Acid/or other vitamin supplements at any time

Do not use G-CSF during maintenance therapy

Appendix 15: Notes for Ethics Committees

The ICICLE ALL-14 Study wishes to standardize therapy, improve outcome and decrease toxicity for children with ALL across the major centres in India,. The protocol has been designed by Indian clinicians with acollective experience of over 2 decades in treating children with ALL across India. The team has an excellent track record in conducting clinical trials in children with cancer, both in India and internationally. International experts have externally reviewed ICICLE ALL -14.

Synopsis

The Indian childhood collaborative leukaemia group study is a modern, scientifically designed approach to provide uniform standards of care for children with ALL across India. It will use state of the art molecular tools to risk stratify patients. Those at low risk will receive curative therapy of a lower intensity compared to those at high risk. The study asks two randomised questions. The first is whether decreasing the use of steroids in induction will reduce toxicity during induction. The second is whether the use of a single drug- Mitoxantrone, instead of 3 doses of Daunorubicin, during delayed intensification decrease toxicity and improve survival. As a multicentre study we anticipate in recruiting 500 patients in the first year and subsequently 750 patients in each subsequent year. A sample size of 2240 patients (4 year recruitment) given as an 80% power to detect a 3% difference between the two induction arms. Similarly 1800 patients will give us 80% power to detect a 5% difference in the second randomisation. The study is thus adequately powered. This approach will not only benefit Indian patients, but also has the potential to influence design of clinical trials internationally.

StudyDesign

We have defined ICiCLe ALL-14 as an open label, randomised phase III/IV study. The reason for classifying this as a phase IVstudy, is that the risk stratification, all drugs and therapeutic blocks of therapy are those extensively used by the major international study groups. Thus the main aim of the ICiCLe ALL-14 study is to standardise care across the centres in India. Thus this qualifies as a phase IV study

To move forward from ICiCLe ALL-14 to its successor, we also need to understand how best to benefit our patient group. We have looked to decrease toxicity by decreasing therapy in low risk patients, using modern molecular tools (MRD). This kind of evaluation needs to be done in a randomised fashion. Thus with this definition, ICiCLe ALL-14 can also be considered to be a phase IV study which is seeking to establish the best standards of care. There is no investigational medicinal product (IMP) in this protocol.

Patient Information and Consent

We have designed patient information sheets (PIS) based on internationally (**Appendix 16**) accepted standards. The PIS is designed to be easily read and understood by the patient and their families.

More detailed information is available if required. At diagnosis, all patients with ALL presenting to the recruiting centres will be offered the ICICLE ALL-14 protocol. Eight days are available for the families to understand the concept of randomisation. If families do not wish to participate in the randomisation, they will be treated on the non-randomised arm of the treatment protocols this is the standard of care. Patients may also opt for one and not both randomisations. They will be consented separately for both, the firstby day 8 and the second prior to starting delayed intensification. All randomisations will be performed by a computerised centralised study management system with instant access. No data that can identify the patient will be held centrally.Where required, consent will be recorded not only on paper but also by video.

The Management Structure

Study Management Group

The Study Managementgroup(TMG)is composed of the Chief Investigator,Co-investigators and the study team and the Clinical Trials Unit (based at Tata Medical Centre in Kolkata). The TMG is responsible for the day-to-day running and management of the study and will communicate by email, telephone and annual meetings.

Data Monitoring Committee

Data analyses will be supplied in confidence to an independent Data Monitoring Committee (DMC), which will be asked to give advice on whether the accumulated data from the study, together with the results from other relevant research, justifies the continuing recruitment of further patients. The DMC will operate in accordance with a study specific charter based upon the template created by the Damocles Group. During the recruitment phase of the study the DMC is scheduled to meet within the six months of the first site opening to recruitment and one month prior to the due date of the Annual Safety Report and annually thereafter. Additional meetings will be held if required. Additional meetings may be called if recruitment is faster than anticipated and the DMC may, at their discretion, request to meet more frequently or continue to meet following completion of recruitment. An emergency meeting may also be convened if a safety issue is identified. The DMC will report directly to the Trial Management Group (TMG) who will convey the findings to the independent ethical committees at the various institutions. The DMC may consider recommending the discontinuation of the study if the recruitment rate or data quality are unacceptable or if any issues are identified which may compromise patient safety. The study would also stop early if the interim analyses show the differences between treatments that were deemed to be convincing to the clinical community .The DMC statistician will be able to directly interrogate the database.

Ethical Considerations

The study will be performed in accordance with the recommendations by guiding physicians in biomedical research involving human subjects, adopted by the 18thWorld Medical Association General Assembly,Helsinki,Finland,June1964,amendedatthe48thWorld Medical Association General Assembly,SomersetWest,RepublicofSouthAfrica,October1996(website:http://www.wma.net/en/30 publications/10policies/b3/index.html). See also Appendix 12 for Declarationof Helsinki.
The study will be conducted in accordance with the International Conference on Harmonisation Guidelines for Good Clinical Practice(ICHGCP).The protocol will be submitted to and approved by the main Research Ethics Committee(REC) or Independent Review Boards (IRB) prior to circulation. Before any patients are enrolled into the study thePrincipal Investigator at each site is required to obtain local ethical approval.Sites will not be permitted to enrol patients until written confirmation of this approval is received by theCTU.

All participating centres need to obtain approval from institutional review boards. An original copy of the authorisation will be kept with the Clinical Trial Manager. The Tata Medical Center, Kolkata will assume the role of the sponsor for Good Clinical Practices and will delegate responsibility to ensure local compliance to each centre via a contract. This does not affect the individual

clinicians'responsibility to take immediate action if thought necessary to protect the health and interest of individual patients.

CONFIDENTIALITY AND DATA PROTECTION

Personal data recorded on all documents will be regarded as strictly confidential. All data will be collected and stored in customized remote entry database. Patients will be identified using only their unique study number,hospital number,initials and date of birth on the Case Report Form and correspondence between the Trials Office and the participating site.

The Investigator must maintain documents not for submission to the Trials Office(e.g.Patient Identification Logs) in strict confidence.In the case of specific issues and/or queries from the regulatory authorities, it will be necessary to have access to the complete study records,providedthat patient confidentiality is protected.

TheTrials Office will maintain the confidentiality of all patients' data andwill not discloseinformation by which patients may be identified to any third party other than those directly involved in the treatment of the patient and organisations for which the patient as given explicit consent for data transfer.Representatives of the study team may be required to have access to patient's notes for quality assurance purposes but patients should be reassured that their confidentiality will be respected at all times.

PUBLICATION POLICY

Results of this study will be submitted for publication in a peer review ed journal. The manuscript will be prepared by the Trial Management Group (TMG) and author ship will be determined by mutual agreement.

Appendix 16: Informed Consent Template

Centre logo Centre Name
Centre Address

Consent Form, ICiCLe ALL-14/INPOG-ALL-15-01 Protocol

Subject Initials: _________
Subject's Name: ______________________________
Date of Birth/ Age: ___/____/________
MR Number: ___________

Start Date: ___/___/_____

I understand that my child has Acute Lymphoblastic Leukemia and further tests and treatments are needed to determine risk. The treatment will consist of periods of hospitalizations and out-patient visits and will involve the use of chemotherapy and possible radiotherapy. The treatment is intensive and toxic but also associated with a high cure rate. I understand that success is not guaranteed and disease may recur or my child may die of infections associated with the treatment.

The objectives of **ICiCLe ALL-14 study** have been explained to me. I also understand that this is the **'standard of care'** for children with Acute Lymphoblastic Leukemia at this centre.

1. I confirm that I have read and understood the information sheet dated ……………………… for the above study ICiCLe ALL-14 and have had the opportunity to ask questions.

Y	N

2. I give consent for my child to participate in the ICiCLe ALL -14 study

Y	N

3. I have been explained the reasons for randomisation in ICiCLe ALL-14 and I understand that this does not affect the care of my child in any way. I give my consent for my child to participate in the randomisation.

Y	N

Randomisation 1

Y	N

Randomisation 2

I understand that during the process of my child's treatment, data will be collected on my child's progress. This data will be used and published but all my family's details will be anonymised. This data will benefit future patients but probably not my child. If any information acquired will benefit my child or my family, my physician will discuss it with me directly.

4. I give consent for data to be acquired and kept for analysis regarding my child's response to treatment.

Y	N

I understand that at variable time points, blood, bone marrow and other tissue samples will be collected from my child both for diagnosis and monitoring therapy. I understand that Tata Medical Centre, Kolkata will store any extra material obtained from these samples at these times to be used for future ethically approved studies. All materials will be anonymised and the research authorised by appropriate independent review board.

5. I give consent for additional tissue material to be collected and banked from my child.

Y	N

Signing consent does not take the right of withdrawal away. You may withdraw any or one consent obtained at any time. You may choose not to give a reason for your withdrawal from the study. Withdrawal from the study will not affect the care of your child in any way.

Signature (or Thumb impression) of the Subject/Legally Acceptable Representative- ___________________________

Date: __/__/____

Signatory's Name: __________________________

Signature of the Investigator: __________________________

Study Investigator's Name: __________________________ Date: __/__/____

Signature of the Witness: __________________________

Name of the Witness: __________________________ Date: __/__/____

Appendix 17: Assent form, Patient Information Sheet for Children- InPOG ALL 15-01 ICiCLe ALL 14 study

For Age Groups 8 years and above:

Dear ……………………………………you will be treated for Acute Lymphoblastic Leukaemia, which we tend to call 'ALL'.

We would like to invite you to take part in a study which hopes to improve the way we treat your disease

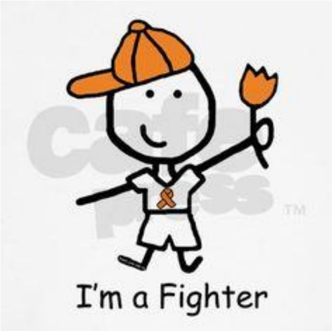

Background:

To make the disease go away, we will have to give you different medicines which are called chemotherapy. The treatment will last for 2.5 years. For the first 6 months you will have to visit the hospital more often for blood tests, bone marrow tests and for your medicines.

1. Why Have I Been Chosen?

We will be asking all children with ALL if they would be willing to take part in this study. The more people that take part, the more information we can gather that will help us in the treatment of children with Acute Lymphoblastic Leukaemia.

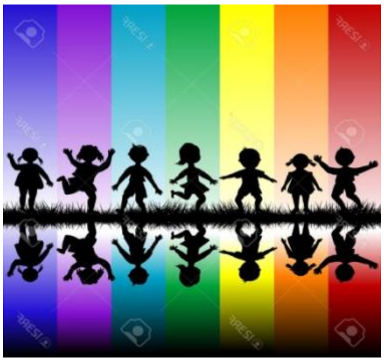

2. Do I Have To Take Part?

You do not have to take part in the study. If you and your parents decide to take part, you can still change your minds at any time and your doctor will not

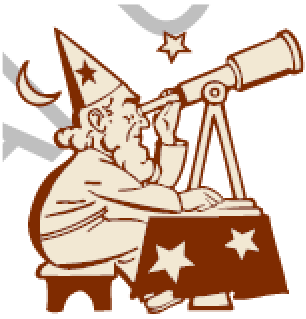

mind at all.

3. What Will Happen If I Do Take Part?

You will have intensive chemotherapy for 6 months.During this time, you will need to come to the hospital frequently and receive your medicines in the Day care. We will admit you in the hospital if you are sick or during some parts of the treatment. At some times you will need bone marrow and lumbar puncture injections, sometimes also for putting a central line. These procedures will be explained to you by the team looking after you.

After the first 6 months, you will be treated for 2years with chemotherapy which you can take by mouth at home. You will need a blood count at least once in 2 weeks to adjust the doses of your medication if needed. During these 2 years of treatment you should start going to school. You will need to be seen in

the clinic when advised by your doctor and may also require a lumbar puncture every three months.

4. Are There Any Other Choices?

Should you or your parents decide not to take part in this study,and then your doctor will treat you on this treatment protocol as this is how we treat all children with Acute Lymphoblastic Leukaemia in our hospital.

5. What Are The Side Effects?

You may feel a bit sick, not feel like eating, have a high temperature and your hair might fall out. This is temporary and your hair will re-grow once the treatment is over.

6. What Are The Benefits?

ALL is a highly curable disease when treated properly. Whatever you and your parents decide, you will only get the very best treatment from your doctors and nurses. We hope that the information we get from your participation in the study will make the treatment even better for children like you in the future.

7. Will anyone else know that I am taking part in this study?

The only people who will know that you are taking part in this study will be the team of doctors and nurses looking after you and a group of people who collect information on all the children like you in this study.

8. What will happen to the results of the study?

When the study is finished, the results will be printed in a special sort of newspaper for doctors but your name will not be in it.

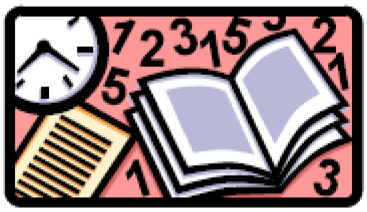

9. If I have any questions now or later?

If you have any questions, don't be afraid to talk to somebody about them. Doctors and nurses get asked questions all the time, so they won't mind.

Thank you for listening to or reading this with a grown up

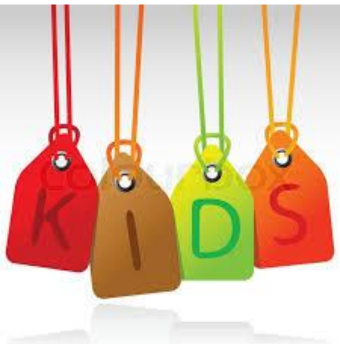

Appendix 18: Assent Form

Centre logo Centre Name
Centre Address

Assent Form

I have had the information read to me. I have had my questions answered and know that I can ask questions later if I have them.
I agree to take part in the study.
OR
I do not wish to take part in the study and I have not signed the assent below.

Print name of child ___________________

Signature/thumb impression of child: ____________________

Date (DD/MM/YYYY):________________

Print name of parent/ guardian___________________

Relation to Child

Signature/thumb impression of parent/guardian ____________________

Date (DD/MM/YYYY):________________

Print name of witness (not a parent) _________________

Signature of witness ______________________

Date (DD/MM/YYYY) _____________________

Print Name of person taking the assent________________________

Signature of person taking the assent __________________________

Date (DD/MM/YYYY) ___________________________

Copy provided to the participant ________

Parent/Guardian has signed an informed consent: Yes ___No _____

Appendix 19: Dose Rounding Rules

DRUG	DOSE ROUNDING	EXAMPLE		DOSE CAPPING
		**If Calculated dose is**	**Recommended dose would be**	
Prednisolone (steroid)	Round off dose to nearest **2.5 mg**	41 mg	40 mg	120 mg
		42 mg	42.5 mg	
Dexamethasone (steroid)	Round off dose to nearest **0.5 mg**	5.3 mg	5.5 mg	20 mg
		4.8 mg	5.0 mg	
Vincristine	Round off dose to nearest **0.1 mg**	1.64 mg	1.6 mg	2 mg
		1.38 mg	1.4 mg	
PEG Asparaginase	Round off dose to nearest **100 Units**	870 Units	900 Units	No
		810 Units	800 Units	
Native E. coli Asparaginase	Round off dose to nearest **500 Units**	7700 Units	7500 Units	No
		7100 Units	7000 Units	
Anthracyclines*	Round off dose & remove decimal	29.5 mg	29 mg	No
		38.6 mg	39 mg	
		42.4 mg	42 mg	
Cyclophosphamide	Round off dose to nearest **50 mg**	1570 mg	1600 mg	No
		770 mg	800 mg	
		680 mg	700 mg	
Cytarabine	Round off dose & remove decimal	29.5 mg	29 mg	No
		38.75 mg	39 mg	
		30.35 mg	30 mg	
High dose Methotrexate ($3\ g/m^2$ & $5\ g/m^2$)	Round off dose to nearest **50 mg**	2640 mg	2650 mg	No
		2040 mg	2000 mg	
		2310 mg	2300 mg	
Capizzi regimen Methotrexate	Round off dose to nearest **5 mg** & remove decimal	97.5 mg	100 mg	No
		38.6 mg	40 mg	
		22.5 mg	25 mg	

*** Includes Daunorubicin, Doxorubicin and Mitoxantrone**

Appendix 20: SentinelToxicities (Descriptions and Grading)

Adverse Event	Grade 3 (Severe)	Grade 4 (Very severe)
Allergy	Allergic reaction, utricaria, angioedema bronchospasm; does not include redness at site of infections. Requires discontinuation of Asparaginase.	Requires ICU, anaphylaxis, immediate fluids and adrenaline
Bleeding	Transfusion of ≥10 m/kg red cells required; platelet and plasma transfusion indicated;	Urgent radiologic, endoscopic, operative intervention indicated; Life-threatening.
Thromboembolic event	Complicated thrombosis, e.g., pulmonary embolism, non-embolic	Life-threatening thrombosis, e.g. major pulmonary embolism, cerebro-vascular event, arterial insufficiency); hemodynamic
Hyperglycaemia	>200 mg/dL requiring oral medication and/or insulin	Life-threatening consequences; DKA leading to coma.
Sepsis	Febrile Neutropenia	Life-threatening consequences; urgent intervention indicated
Infusion site extravasation	Ulceration or necrosis; tissue damage; requiring medical management.	Ulceration or necrosis; tissue damage; requiring surgical management.
Hypertension	Requires medication	Requires ICU
Vomiting	>=6 episodes (separated by 5minutes) in 24 hrs; tube feeding, TPN or hospitalization indicated	Life-threatening consequences; urgent intervention indicated
Oral mucositis	Painful erythema, oedema, or ulcers requiring IV hydration	Severe ulceration requiring parenteral or enteral nutritional support
Constipation/ Ileus	Abdominal pain & tenderness with distension, radiological evidence of distended loops with retained faeces requiring more than one laxative (all of the above should be present to qualify for Grade III constipation)	Unable to tolerate oral feed with the presence of Grade III symptoms
Diarrhoea	Increase of >=7 stools per day over baseline; incontinence hospitalization indicated;	Life-threatening consequences; urgent intervention indicated
Pancreatitis	Severe pain; vomiting; medical intervention indicated(e.g., analgesia, nutritional support)	Life-threatening consequences; urgent intervention indicated
Peripheral neuropathy	Jaw pain requiring analgesics.	CNS palsy, inability to stand and/or walk.
Avascular necrosis	Severe symptoms; limiting self-care ADL; elective operative intervention indicated	Life-threatening consequences; urgent intervention indicated
Seizure (Grade II: Brief generalized seizures not requiring meds)	>1 generalized seizures requiring medical intervention	Life-threatening; prolonged repetitive seizures
Psychosis	Severe psychotic symptoms(e.g., paranoid; extreme disorganization); hospitalisation not indicated	Life-threatening consequences, threats of harm to self or others; hospitalisation indicated
Encephalopathy syndrome	Severe symptoms; abnormal imaging studies; limiting self-care ADL	Life-threatening consequences; urgent intervention indicated
Serum Bilirubin	>3.0-10.0 ×ULN	> 10.0 × ULN
Serum Albumin	<2 g/dL; <20 g/L	Life-threatening consequences; urgent intervention indicated
Serum Creatinine (increased)	>3.0 baseline;>3.0-6.0×ULN	>6.0×ULN
SIADH	Sodium<125, asymptomatic	Sodium<125, symptomatic

Appendix 21: Declaration of Helsinki

Policy

WORLD MEDICAL ASSOCIATION DECLARATION OF HELSINKI

Ethical Principles for Medical Research Involving Human Subjects

Adopted by the 18th WMA General Assembly, Helsinki, Finland, June 1964, and amended by the
29th WMA General Assembly, Tokyo, Japan, October 1975
35th WMA General Assembly, Venice, Italy, October 1983
41st WMA General Assembly, Hong Kong, September 1989
48th WMA General Assembly, Somerset West, Republic of South Africa, October 1996 and the
52nd WMA General Assembly, Edinburgh, Scotland, October 2000

Note of Clarification on Paragraph 29 added by the WMA General Assembly, Washington 2002
Note of Clarification on Paragraph 30 added by the WMA General Assembly, Tokyo 2004

A. Introduction

1. The World Medical Association has developed the Declaration of Helsinki as a statement of ethical principles to provide guidance to physicians and other participants in medical research involving human subjects. Medical research involving human subjects includes research on identifiable human material or identifiable data.

2. It is the duty of the physician to promote and safeguard the health of the people. The physician's knowledge and conscience are dedicated to the fulfillment of this duty.

3. The Declaration of Geneva of the World Medical Association binds the physician with the words, "The health of my patient will be my first consideration," and the International Code of Medical Ethics declares that, "A physician shall act only in the patient's interest when providing medical care which might have the effect of weakening the physical and mental condition of the patient."

4. Medical progress is based on research which ultimately must rest in part on experimentation involving human subjects.

5. In medical research on human subjects, considerations related to the well-being of the human subject should take precedence over the interests of science and society.

6. The primary purpose of medical research involving human subjects is to improve prophylactic, diagnostic and therapeutic procedures and the understanding of the aetiology and pathogenesis of disease. Even the best proven prophylactic, diagnostic, and therapeutic methods must continuously be challenged through research for their effectiveness, efficiency, accessibility and quality.

7. In current medical practice and in medical research, most prophylactic, diagnostic and therapeutic procedures involve risks and burdens.

8. Medical research is subject to ethical standards that promote respect for all human beings and protect their health and rights. Some research populations are vulnerable and need special protection. The particular needs of the economically and medically disadvantaged must be recognized. Special attention is also required for those who cannot give or refuse consent for themselves, for those who may be subject to giving consent under duress, for those who will not benefit personally from the research and for those for whom the research is combined with care.

9. Research Investigators should be aware of the ethical, legal and regulatory requirements for research on human subjects in their own countries as well as applicable international requirements. No national ethical, legal or regulatory requirement should be allowed to reduce or eliminate any of the protections for human subjects set forth in this Declaration.

B. Basic Principles for All Medical Research

10. It is the duty of the physician in medical research to protect the life, health, privacy, and dignity of the human subject.

11. Medical research involving human subjects must conform to generally accepted scientific principles, be based on a thorough knowledge of the scientific literature, other relevant sources of information, and on adequate laboratory and, where appropriate, animal experimentation.

12. Appropriate caution must be exercised in the conduct of research which may affect the environment, and the welfare of animals used for research must be respected.

13. The design and performance of each experimental procedure involving human subjects should be clearly formulated in an experimental protocol. This protocol should be submitted for consideration, comment, guidance, and where appropriate, approval to a specially appointed ethical review committee, which must be independent of the investigator, the sponsor or any other kind of undue influence. This independent committee should be in conformity with the laws and regulations of the country in which the research experiment is performed.
The committee has the right to monitor ongoing trials. The researcher has the obligation to provide monitoring information to the committee, especially any serious adverse events. The researcher should also submit to the committee, for review, information regarding funding, sponsors, institutional affiliations, other potential conflicts of interest and incentives for subjects.

14. The research protocol should always contain a statement of the ethical considerations involved and should indicate that there is compliance with the principles enunciated in this Declaration.

15. Medical research involving human subjects should be conducted only by scientifically qualified persons and under the supervision of a clinically competent medical person. The responsibility for the human subject must always rest with a medically qualified person and never rest on the subject of the research, even though the subject has given consent.

16. Every medical research project involving human subjects should be preceded by careful assessment of predictable risks and burdens in comparison with foreseeable benefits to the subject

or to others. This does not preclude the participation of healthy volunteers in medical research. The design of all studies should be publicly available.

17. Physicians should abstain from engaging in research projects involving human subjects unless they are confident that the risks involved have been adequately assessed and can be satisfactorily managed. Physicians should cease any investigation if the risks are found to outweigh the potential benefits or if there is conclusive proof of positive and beneficial results.

18. Medical research involving human subjects should only be conducted if the importance of the objective outweighs the inherent risks and burdens to the subject. This is especially important when the human subjects are healthy volunteers.

19. Medical research is only justified if there is a reasonable likelihood that the populations in which the research is carried out stand to benefit from the results of the research.

20. The subjects must be volunteers and informed participants in the research project.

21. The right of research subjects to safeguard their integrity must always be respected. Every precaution should be taken to respect the privacy of the subject, the confidentiality of the patient's information and to minimize the impact of the study on the subject's physical and mental integrity and on the personality of the subject.

22. In any research on human beings, each potential subject must be adequately informed of the aims, methods, sources of funding, any possible conflicts of interest, institutional affiliations of the researcher, the anticipated benefits and potential risks of the study and the discomfort it may entail. The subject should be informed of the right to abstain from participation in the study or to withdraw consent to participate at any time without reprisal. After ensuring that the subject has understood the information, the physician should then obtain the subject's freely-given informed consent, preferably in writing. If the consent cannot be obtained in writing, the non-written consent must be formally documented and
witnessed.

23. When obtaining informed consent for the research project the physician should be particularly cautious if the subject is in a dependent relationship with the physician or may consent under duress. In that case the informed consent should be obtained by a well-informed physician who is not engaged in the investigation and who is completely independent of this relationship.

24. For a research subject who is legally incompetent, physically or mentally incapable of giving consent or is a legally incompetent minor, the investigator must obtain informed consent from the legally authorized representative in accordance with applicable law. These groups should not be included in research unless the research is necessary to promote the health of the population represented and this research cannot instead be performed on legally competent persons.

25. When a subject deemed legally incompetent, such as a minor child, is able to give assent to decisions about participation in research, the investigator must obtain that assent in addition to the consent of the legally authorized representative.
26. Research on individuals from whom it is not possible to obtain consent, including proxy or advance consent, should be done only if the physical/mental condition that prevents obtaining informed consent is a necessary characteristic of the research population. The specific reasons for involving research subjects with a condition that renders them unable to give informed consent should be stated in the experimental protocol for consideration and approval of the review committee. The protocol should state that consent to remain in the research should be obtained as soon as possible from the individual or a legally authorized surrogate.

27. Both authors and publishers have ethical obligations. In publication of the results of research, the investigators are obliged to preserve the accuracy of the results. Negative as well as positive results should be published or otherwise publicly available. Sources of funding, institutional affiliations and any possible conflicts of interest should be declared in the publication. Reports of experimentation not in accordance with the principles laid down in this Declaration should not be accepted for publication.

C. Additional Principles For Medical Research Combined with Medical Care

28. The physician may combine medical research with medical care, only to the extent that the research is justified by its potential prophylactic, diagnostic or therapeutic value. When medical research is combined with medical care, additional standards apply to protect the patients who are research subjects.

29. The benefits, risks, burdens and effectiveness of a new method should be tested against those of the best current prophylactic, diagnostic, and therapeutic methods. This does not exclude the use of placebo, or no treatment, in studies where no proven prophylactic, diagnostic or therapeutic method exists.

30. At the conclusion of the study, every patient entered into the study should be assured of access to the best proven prophylactic, diagnostic and therapeutic
methods identified by the study.

31. The physician should fully inform the patient which aspects of the care are related to the research. The refusal of a patient to participate in a study must never interfere with the patient-physician relationship.

32. In the treatment of a patient, where proven prophylactic, diagnostic and therapeutic methods do not exist or have been ineffective, the physician, with informed consent from the patient, must be free to use unproven or new prophylactic, diagnostic and therapeutic measures, if in the physician's judgement it offers hope of saving life, re-establishing health or alleviating suffering. Where possible, these measures should be made the object of research, designed to evaluate their safety and

efficacy. In all cases, new informationshould be recorded and, where appropriate, published. The other relevantguidelines of this Declaration should be followed.

*1.**Note of clarification on paragraph 29 of the WMA Declaration of Helsinki***

The WMA hereby reaffirms its position that extreme care must be taken in making use of a placebo-controlled study and that in general this methodology should only be used in the absence of existing proven therapy. However, a placebo-controlled trial may be ethically acceptable, even if proven therapy is available, under the following circumstances:
- Where for compelling and scientifically sound methodological reasons its use is necessary to determine the efficacy or safety of a prophylactic, diagnostic or therapeutic method; or
- Where a prophylactic, diagnostic or therapeutic method is being investigated for a minor condition and the patients who receive placebo will not be subject to any additional risk of serious or irreversible harm.

All other provisions of the Declaration of Helsinki must be adhered to, especially the need for appropriate ethical and scientific review.

*2.**Note of clarification on paragraph 30 of the WMA Declaration of Helsinki***

The WMA hereby reaffirms its position that it is necessary during the study planning process to identify post-trial access by study participants to prophylactic, diagnostic and therapeutic procedures identified as beneficial in the study or access to other appropriate care. Post-trial access arrangements or other care must be described in the study protocol so the ethical review committee may consider such arrangements during its review.

Appendix 22: IAP Body Mass Index Reference Charts_2015

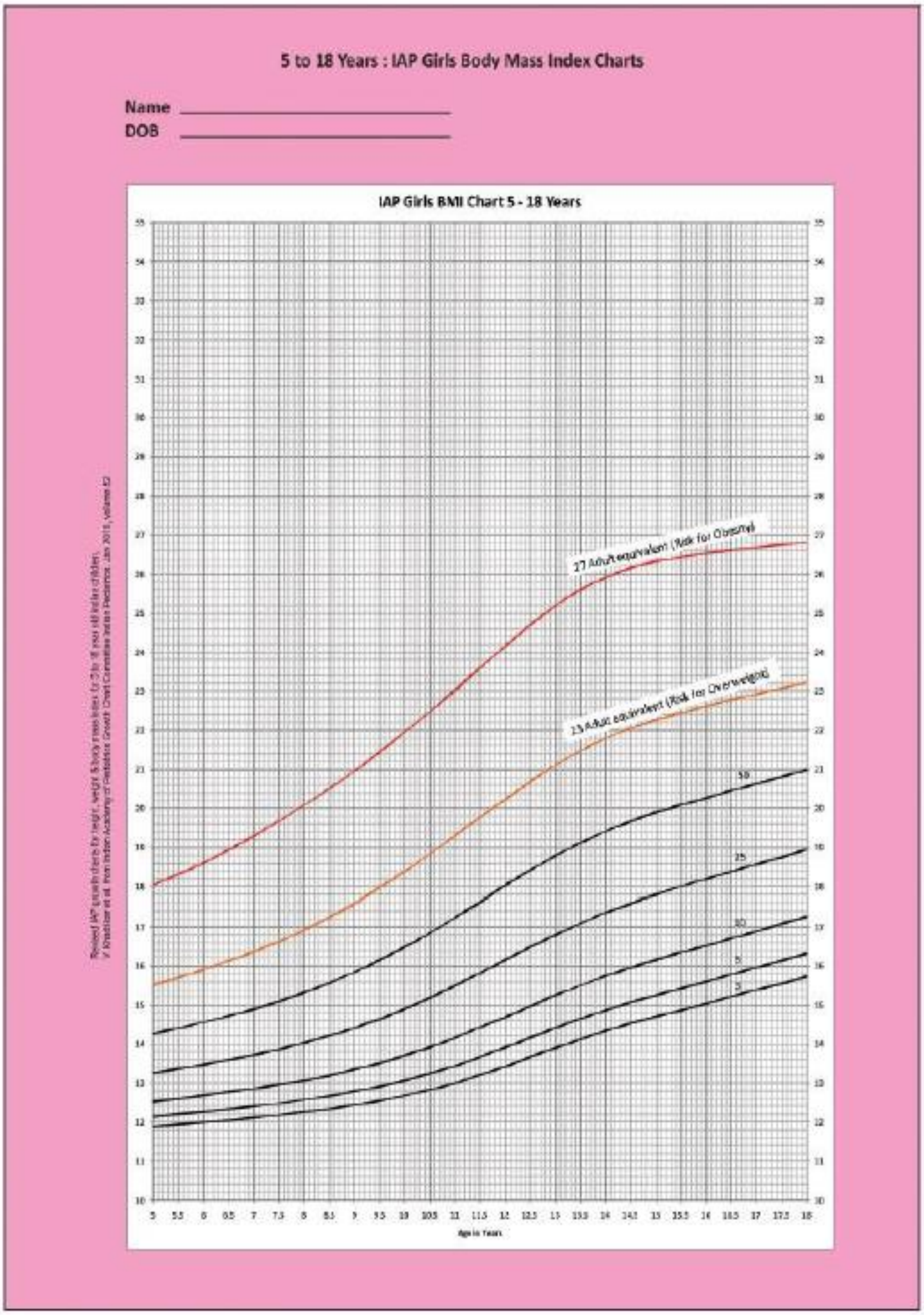

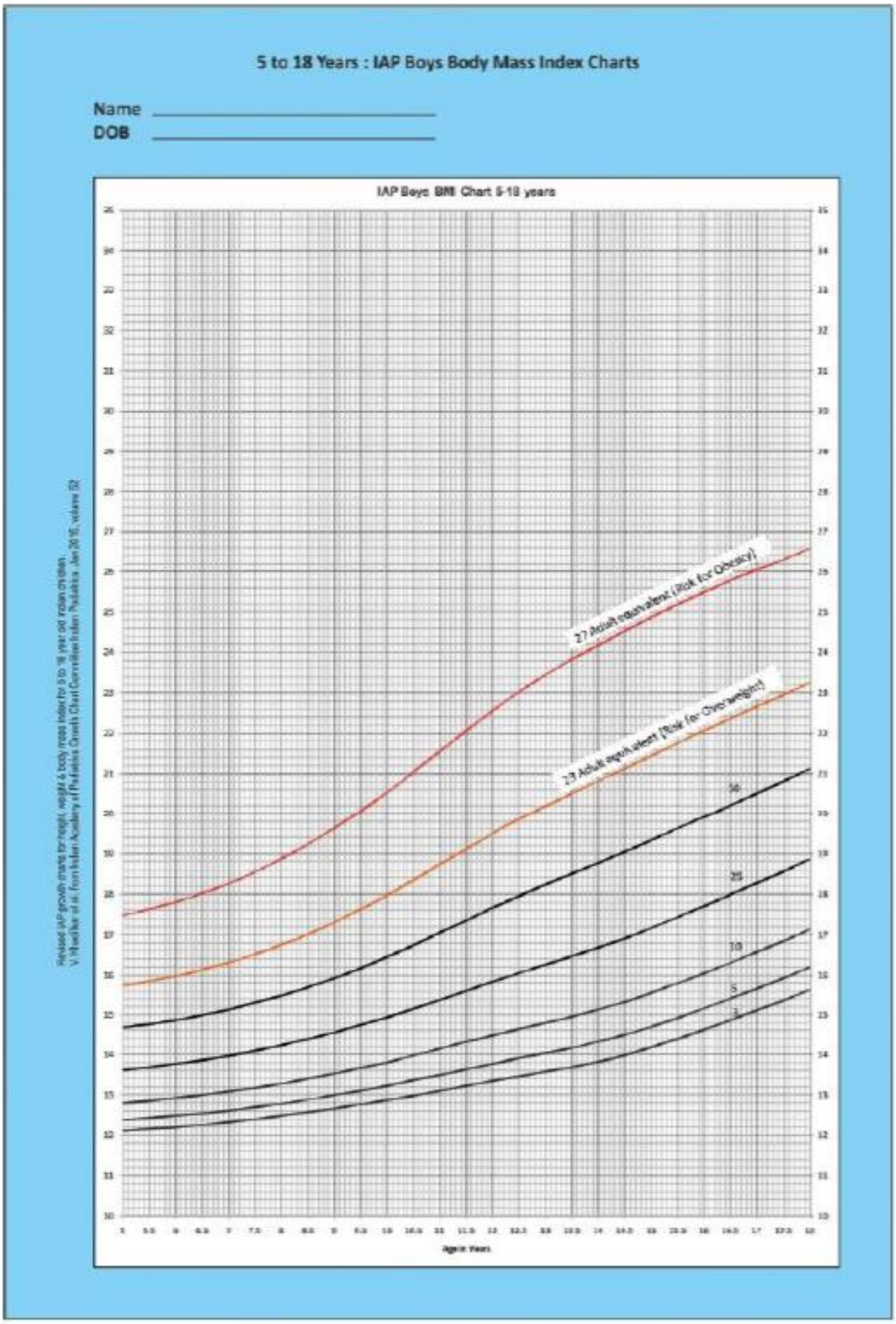
5 to 18 Years : IAP Boys Body Mass Index Charts
Name
DOB
IAP Boys BMI Chart 5-18 years
27 Adult equivalent (Risk for Obesity)
23 Adult equivalent (Risk for Over weight)
50
25
10
5
3
Age in Years

Appendix 23: Trial Withdrawal, Treatment Abandonment, Lost to follow up

1. Withdrawal from trial

The following patients will be withdrawn from trial:

(a) Withdrawal of consent to participate in the trial

(b) Interruption of protocol-based treatment (e.g. due to severe infection, organ injury, drug toxicities). Interruption of protocol-based treatment includes

- (i) De-intensification of treatment, resulting in administration of a less-intensive treatment phase (e.g. Standard Risk Consolidation in a High-Risk patient)
- (ii) Omission of an entire treatment phase (for instance, omission of interim maintenance)

(c) Failure to achieve remission at the end of the consolidation phase, including

- (i) Persistent unequivocal clinical disease (with the exception of persistent testicular enlargement)
- (ii) Bone marrow blasts ≥ 5%
- (iii) Persistent CNS disease, defined as failure of clearance of blasts in cerebrospinal fluid 42 days (6 weeks) from start of weekly intrathecal treatments
- (iv) Relapse or disease progression at any time during treatment

Patients withdrawn from trial will continue to be followed up as part of the overall trial cohort.

2. Treatment abandonment

Patients who have missed scheduled treatment for more than 12 weeks, and cannot be contacted and/or have not responded to repeated reminders.

3. Lost to follow-up

In patients who have completed therapy, absence of follow-up for more than 12 months from last review.
