# Supplementary material for: Toxicity during induction of pulsed versus continuous prednisolone in children with acute lymphoblastic leukaemia: a multi-centre, open label, randomised, phase 3 trial from India (2016–2022)
Source: Lancet Reg Health Southeast Asia. 2026 Jun 7;50:100788. doi: 10.1016/j.lansea.2026.100788 (PMC13366301; doi:10.1016/j.lansea.2026.100788)
Supplement: Statistical Plan v2 [file mmc4.pdf]

InPOG-ALL-15-01 (ICiCLe ALL-14)

Statistical Plan (Dated May 2019) version 2
Based on InPOG-ALL-15-01 (ICiCLe ALL-14) protocol version 5.1 Dated January 2020

1. Introduction

This statistical analysis plan describes the analyses required for the Childhood Acute Lymphoblastic Leukemia (ALL) (ICICLE ALL-14) open label randomised clinical trial. The analysis plan focuses on both efficacy and safety summaries and analysis of efficacy and safety endpoints.

2. Aim of the Trial

The trial seeks to (i) establish a uniform standard of care for newly diagnosed children (aged 1-18 years of age) with ALL across centres in India (ii) improve the prognosis of children with ALL to those published reports (~65%).

3. Participating Centres

Tata Medical Center, Kolkata (Nodal Centre)
Tata Memorial Centre, Mumbai
Postgraduate Institute of Medical Education and Research, Chandigarh
All India Institute of Medical Sciences, New Delhi
MAX Superspeciality Hospital, New Delhi
Cancer Institute (WIA), Chennai

4. Study Design

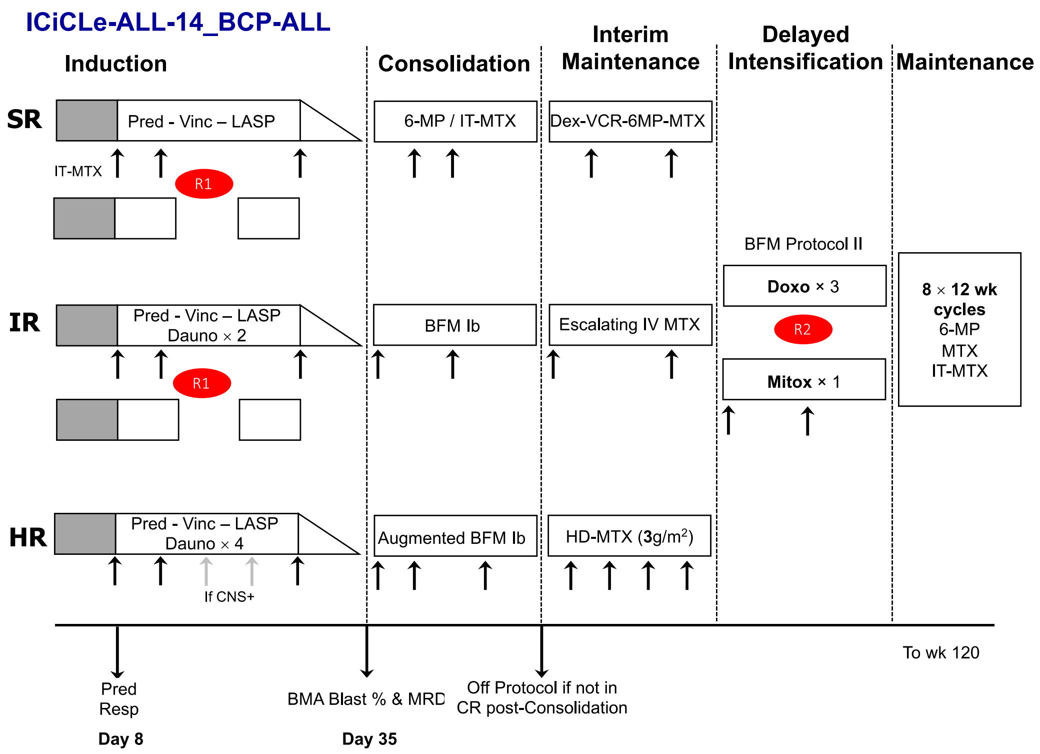

The InPOG-ALL-15-01, is a multicentre, treatment optimisation, open label randomised trial. It contains two phase III randomisations.

R1 - Randomisation: Prospective, randomised, open label phase III study comparing the efficacy and toxicity of 3 weeks of corticosteroid versus 5 weeks of corticosteroid during induction.

R2 - Randomisation: Prospective, randomised, open label phase III study comparing the efficacy and toxicity of 3 doses of Doxorubicin versus 1 dose of Mitoxantrone.

5. Risk Stratification

There is a two-stage risk stratification. For patients with BCP-ALL prior to start of therapy, patients with a poor response to prednisolone on day 8 and/or CNS3 and/or high risk cytogenetics are classified as high risk (HR). Patients with good prednisolone response, non-high risk cytogenetics and CNS1/2 are classified as standard risk (SR) if NCI standard risk and intermediate risk (IR) if NCI high risk. T-ALL patients are not risk stratified.

Where risk stratification is not available prior to diagnosis, patients are classified as HR.

At the end of induction SR and IR BCP-ALL patients are re-classified as HR if the minimal residual disease (MRD) is $\geq 10^{-4}$. Where MRD is not evaluable, patients are classified as HR.

6. Study population

1. Age > 1-year and <18 years at time of enrolment
2. Previously untreated
3. ALL diagnosis confirmed by morphology and flow-cytometry

Exclusion Criteria

Patients with any of the following conditions or treatments will be excluded from this study:

1. Previously treated (see below)
2. ALL-L3

BCP-ALL - SR

Age >1 and <10 years
WC < 50×10^9/L
Prednisolone good responder
No CNS disease
No bulky disease
No high-risk cytogenetics
In CR at end of induction with MRD $<10^{-4}$

BCP-ALL – IR

Age ≥10 and <18years
WC ≥50×10^9/L
Prednisolone good responder

Bulky disease or testicular disease
No CNS disease
No high-risk cytogenetics
In CR at end of induction with MRD <10^{-4}

<u>BCP-ALL – HR</u>

High risk cytogenetics
Prednisolone poor responder
CNS disease
End induction MRD ≥10^{-4}

Patients who have been treated prior to enrollment are not eligble for the trial with the exception of those who have received steroids only provided this is not for longer than 8 days. If the patient has received steroids for longer than this, they can still be enrolled provided peripheral blast can be detected which automatically places them as HR.

Those who have received one dose of vincristine are also eligible for the study but not for the first randomisation.
Those who have received one dose of IT Methotrexate are eligible for recruitment to the study and randomisation, but will be analysed separately.

7. Definitions

Complete Remission (CR): At the end of induction **-** <5% blasts at the end of induction in a regenerating marrow; no blasts in CSF. Testicular disease should be in clinical remission at the end of consolidation
Induction death: a treatment- and/or disease-related death that occurs during induction prior to achievement of a CR.
Non-response: patients who have a persisting M3 marrow (≥ 25% ALL cells) after the induction phase (SR arm A week 5, arm B week 6) or who have not achieved a CR (< 5% ALL cells in the bone marrow, no evidence of extramedullary disease) after the first consolidation element (SR arm A week 9, arm B week 10).
Death in remission: Death dues to any cause after achievement of a CR.
Treatment Related Mortality (TRM) Death in a patient in CR with a temporal and/or causal relationship to treatment
Relapse Death: Death in a patient with relapsed ALL due to any cause
Relapse: Disease recurrence after achieving CR. This includes >5% blasts in a bone marrow aspirate, confirmed with flow cytometry; >5 blasts in CSF; biopsy proven testicular relapse or at other sites
Secondary malignancy: any subsequent malignant disease occurring after diagnosis of the ALL which is not relapsed ALL
Event Free Survival (EFS): EFS is defined as the time from entry into the trial or randomisation, using the date of registration until induction failure, relapse, second malignancy or death, censoring at last contact.
Overall Survival (OS): OS is defined as the time from entry into the trial or randomisation to death, censoring at last contact.
Withdrawal: A patient is considered withdrawn if (i) patient withdraws consent (ii) there is unacceptable toxicity and families and physicians agree to withdraw the patient (iii) there is disease progression. This is defined as persistence of morphological disease after consolidation; persistence of MRD positivity prior to delayed intensification or adverse genetic factors identified during the

course of trial (e.g. TCF3-HLF1) which suggest that alternative therapeutic approaches are in the best interest of the patient.

Toxicity: Toxicity will be defined using CTCAE 4.03. Only trial specific toxicities will be measured and only grade 3-5 toxicities will be analysed. Grades 3-4 will be grouped and grade 5 (death) analysed separately.

Abandonment: Where a patient who is in CR is lost to further follow up

8. Study Objectives

8.1 Primary Objectives

1. During induction comparison of toxicity with 5 weeks of corticosteroid versus 3 weeks of corticosteroid.
2. During intensification EFS of patients randomised to receive either 3 doses of doxorubicin or 1 dose of mitoxantrone
3. EFS of whole cohort, by randomisation 1 and by pre-treatment and post treatment risk stratification and by participating centre

8.2 Secondary Objectives

1. OS of whole cohort, by randomisation, by risk stratification and by participating centre
2. EFS and OS of prognostic subgroups
3. Toxicity of each block of therapy for the whole cohort by phases of therapy, risk stratification and by participating centres

9. Randomisation

Informed consent for participation in the randomised parts of the study is given separately from the consent to the enrolment into the study. This enables the patients and parents (or persons entitled to custody) to have the choice to participate in the randomisation, or not to be randomised.

Non-randomised patients will be treated as specified for the control arm but their data will not be used for the main analysis. After consent has been given and the patient has been randomised, consent can be withdrawn at any time. Randomisation occurs when the diagnostic findings required for inclusion/exclusion criteria and randomisation stratification are complete and the informed consent for randomisation has been obtained. Randomisation sequence was created using statistical software and was stratified by center with a 1:1 allocation using concealed randomisation, performed centrally by the internet-based data entry system. The first randomisation of arm A versus B, is performed by day 8 when the first risk stratification is known. The second randomisation is performed prior the delayed intensification phase.

10. Sample Size Calculation

Though the trial opened at the end of October 2016, all centres have been open only since March 2018. As of May 2019, 1347, patients have been recruited to the trial of which, 727 were recruited between Apr 2018 and March 2019. Thus we expect that by end of March 2021 we will have recruited around 2,800 patients.

Randomisation 1 (R1)

Induction deaths in patients with ALL in India at the respective trial centres have been reported at 10-20%. Serious bacterial infections have been reported in 60-70% and a high incidence of fungal infections (19 of 22 isolates) was also reported in induction. No data is available on other toxicities. The primary association with infection is the severity and duration of severe neutropaenia (ANC <0.5 x 10^9/L) and prolonged steroid therapy. In the ICiCLe study, SR patients do not receive daunorubicin as this is associated with prolonged myelosuppression. IR patients receive 2 doses and HR/T 4 doses of daunorubicin. These are non-randomised interventions. The study is investigating a shortened interrupted steroid randomisation in induction. The steroid pulses are phased to coincide with L-Asparaginase administration to decrease the incidence of allergic reactions.

Not all patients recruited to the study are eligible for the steroid (prednisolone) randomisation. T-ALL patients receive dexamethasone and HR BCP-ALL patients receive the full 5-weeks. The IR group contains children aged over 10-years. Older children have increased toxicity to steroids and they will only receive the shorter regimen. Thus, only SR and IR patients < 10-years of age are eligible for the first randomisation. In the pre-trial cohort, 850 patients were SR and 576 were IR and aged between 1-9 years. Thus around 50% (1426/2680 = 53%) of the study population will be eligible for the R1 randomisation. The projected number in R1 randomisation for the duration of the trial is 1400 or 700 in each arm.

Statistical plan v1 (October 2013) assumed a total patient recruitment of 2400. The sample size calculation was based on an annual recruitment of 750 patients and >95% randomisation. With 1120 patients in each arm, it was powered to detect a 3% difference between the two arms at 80% power and a p = 0.05. However, the plan was based on the magnitude of difference that could be detected based on the presumed sample size rather than the actual toxicities that could be measured and also what would be considered to be a significant difference clinically.

No toxicity data was collected in the pre-trial cohort, but this data is now available in the trial cohort. In induction, of the 419 SR and IR patients eligible for randomisation, 146 (35%) had grade 3-5 sepsis. Hypertension was reported in 36 (8.6%). The incidence of other toxicities attributable to steroid therapy are <2%. Assuming that sepsis occurs at 40% in the continuous steroid group and at 30% in the discontinuous steroid group, we expect 280 grade 3-4 sepsis in the continuous steroid arm and 252 in the randomised arm. To detect a 10% difference between the two groups at a power of 80% and a p-value of 0.05, we need 353 patients in each arm. At a 7% difference, i.e. 260 events in the randomised arm, a sample size of 739 patients are required for each arm. Therefore the study is powered to detect a difference between 7-10%. Increased sepsis rates are expected in the IR arm as these patients receive daunorubicin. Separate sub cohort analyses will be performed to explore the differences in events in the SR and IR patient as secondary analyses. Differences in all other toxicities will be descriptive and differences between the categorical variables will be calculated using the x^2 test.

The secondary objective of this randomisation is EFS and OS. This will be considered to be interpretable when the median follow-up time from first randomisation is at least 40 months and accrual has been completed.

Randomisation 2 (R2)

All patients, irrespective of are eligible for the risk status and first randomisation. However only patients still on trial are eligible for this randomisation. In the pre-Trial cohort, there were 93 deaths

in induction, and 83 failed inductions and another 9 deaths prior to the start of delayed intensification. Thus we estimate at least 90% of the trial cohort are eligible for the second randomisation. The end point for this randomisation is the 3-year EFS. We assume that 5% of patients will be lost to follow up and current randomisation acceptance rate for R2 is >95%. Thus assuming that 80% of patients are available for R2, we will have 1120 patients available in each arm if all patients recruited are also randomised to R2. The 3-year estimated EFS for the whole cohort is estimated to be 70% but this may increase to 75%. The sample size calculations can then be based on the following assumptions

Power 80% and p value of 0.05

**Difference in EFS**	**Daunorubicin**	**Mitoxantrone**	**Sample Size**
5%			
	60%	65%	1468
	65%	70%	1373
	70%	75%	1248
6%			
	60%	66%	1012
	65%	71%	945
	70%	76%	856
7%			
	60%	67%	738
	65%	72%	687
	70%	77%	620
10%			
	60%	70%	353
	65%	75%	325
	70%	80%	290

Thus at a power of 80% and a p-value of 0.05, the study is able to detect a 6-10% difference in EFS.

The EFS of the different post induction risk groups i.e SR, IR and HR are expected to be different. It is also possible that other prognostic variables, e.g. age, presenting white cell count, sex and cytogenetic risk groups may show a difference in outcomes. The patterns of relapses may also differ. These will be analysed variables that determine outcome and will also be modelled within this analyses.

11. Data Entry and Data Management

All patient related data will be recorded in the electronic database with a unique trial number (IDM v3.0). This is a web based system allowing real time registration and randomisation and collection of toxicities and events.

11.1 Data collection/Case Report Form (CRF)

All data will be recorded in an electronic case report form (e-CRF). The study software used is based on the TCS IDM platform. The e-CRFs will be filled in by an authorized person (defined in the study

team log) as soon as possible. All data will be recorded online. Data will be transferred between the workstation computer at the study site and the study server via a secure connection so that the data cannot be manipulated.

11.2 Source Data and Patient Files

The information in original documents and records (e. g. patient files, laboratory notes) are defined as Source Data and will be reviewed by the Monitor for Source Data Verification.

11.3 Data Processing

Centres will file all data electronically. To verify accuracy of the data, range, validity and consistency checks will be performed automatically by the database. Implausible or missing data can be corrected or added after consulting the Investigator. Documentation for these corrections will be stored with the eCRFs. All validated data will be stored in the database. After termination of the study and after completion of all entries, the database will be closed for further entries. This process will be documented.

11.4 Direct Access to Source Data

According to ICH-GCP the principal investigator must permit all authorized third parties access to the trial site and insight into the medical records of the trial subjects (source data). This permission includes the clinical trial monitors, auditors and other authorised employees of the sponsor, as well as members of the competent authorities. All these persons are sworn to secrecy.

11.5 Monitoring

A detailed monitor plan will be provided within a monitor manual by the sponsor. The investigators allow the monitor to have access to all of the study materials needed for source data verification and proper review of the study process. All times, the sponsor/investigators/monitors will maintain the confidentiality of the study documents. Furthermore, problems with inconsistent and incomplete data will be discussed. By signing the declaration of informed consent, participants allow access to their documents. With the signature in the protocol, the investigators confirm that auditors and health authority inspectors may have access to the study documentation and accordant medical records. Auditors and inspectors are bound by professional confidentiality and may not pass on any personal information that comes to their knowledge. In the course of audits or inspections, data in the case report forms will be compared with the data for medical record. All the documentation held by the investigators within the scope of the clinical trial, as well as the drug logs of the study medications will be verified.

11.6 Audits/Inspections

Authorized representatives of the Sponsor, a regulatory authority, or an Independent Ethics Committee (IEC) may visit the centre to perform audits or inspections, including source data verification. The purpose of a Sponsor audit or inspections is to systematically and independently examine all the study activities and documents to determine whether these activities were conducted, and data were recorded, analysed, and accurately reported according to the protocol, Good Clinical Practise (GCP), guidelines of the International Conference on Harmonization (ICH), and any applicable regulatory requirements

12 Data and Safety monitoring Committee and Interim Analyses

The study will be monitored by an independent Data and Safety Monitoring Committee (DSMC) that is specifically chosen to include an expert in paediatric oncology, in biostatistics and in clinical trials to ensure utmost competence and vigilance. The DSMC will be constituted before the start of the trial, first DSMC meeting is expected at 1-year recruitment or 100 patients recruited whichever first,

then every 6 months during the first 4 years recruitment and annually thereafter in order to review the trial's progress, safety data (SAEs) and adherence to protocol. DSMC will make recommendations to the independent Trial Management Group (TMG) who will share the decisions with the main IRB. The main IRB will decide if the trial should continue.
Possible recommendations could include:

- No action needed, trial continues as planned
- Early stopping due, for example, to clear benefit or harm of a treatment, futility, or external evidence
- Stopping recruitment within a subgroup
- Extending recruitment (based on actual control arm response rates being different to predicted rather than on emerging differences) or extending follow-up
- Stopping a single arm of the trial
- Sanctioning and/or proposing protocol changes

During the course of the study one interim analysis after 3-years of recruitment for is planned to exclude significant results of the randomised questions that would require a preterm termination of the trial. The results of this analysis will be discussed with the DSMC and are not planned for general release. The DSMC will recommend discontinuing of the trial in all patients or in selected subgroups only if the result is likely to convince a broad range of clinicians (including participants in the trial) and the general clinical community. The ultimate decision for discontinuation of the trial lies with the IRB (TMC).

The interim analysis will be done by the trial statistician and this analyses will be reviewed by an independent statistician who could be the one within the DSMC.

The open report for the DSMC will have overall results (not split by treatment arm) which will be seen by the CI and with permission from the DSMC can be distributed as a confidential document to the Inter-Group TMG, the funder and the IRB's.

12.1 Interim Safety Analysis

This analysis will check the safety and tolerability for treatment in the randomised arms. Detailed data on toxicity and proportion of patients experiencing any toxicity will be given overall and by stratification groups. SAEs, deaths and main characteristics of them will be assessed overall and by stratification groups. MRD results and proportion of negative/positive MRD will also be made available. This data will also be analysed by centre. Data will be analysed using using Chi-square or Fisher's exact test as appropriate.

12.2 Interim efficacy analysis

Efficacy will be investigated after 3-years of recruitment. In 2,800 patients, at a 70% EFS, 840 events are expected to occur at the end of follow up. Interim analyses will be considered once 252 (30%) of events have occurred. EFS will be investigated at this analysis using a Cox analysis of treatment effect on EFS adjusting for the factors used in the randomisation stratification. Consideration will be given to stopping the trial early if the significance level for treatment effect in the Cox analysis is < 0.00034

13. Early stopping rules

This is extant of the interim analyses and is based on the annual report. This is based on treatment related mortality (TRM) and if the TRM exceeds 3 standardised deviations between the two

randomised arms. This will be shown by a p-value of 0.001 or less on a two-sided Fisher's exact test TRM will be estimated by all deaths in randomised patients regardless of cause and timing. This is done in order to remove bias introduced by multiple centres classifying deaths as treatment related (or not) and in light of the fact that this guideline will be used early in the trial when the majority of the deaths will be treatment related.

Early stopping is based on the difference in proportion of TRM between the two randomised arms when it exceeds three times the standard deviation (SD) for the overall proportion of TRM based on the expected sample size. However, despite defining the expected boundary sample size and proportion of TRM could be different and boundaries may need to be recalculated.

14. Descriptive Analyses

14.1 The Study Population

The recruitment of patient will be illustrated using CONSORT flow diagram (below). In particular numbers will be presented for all patients registered, showing numbers in each randomised arm, compliant, and analysed with respect to the primary objectives. The number of patients who are ineligible or excluded (failure to satisfy inclusion and exclusion criteria, refusal to participate) or withdrawn from treatment (with reasons) will also be recorded.

CONSORT Diagram

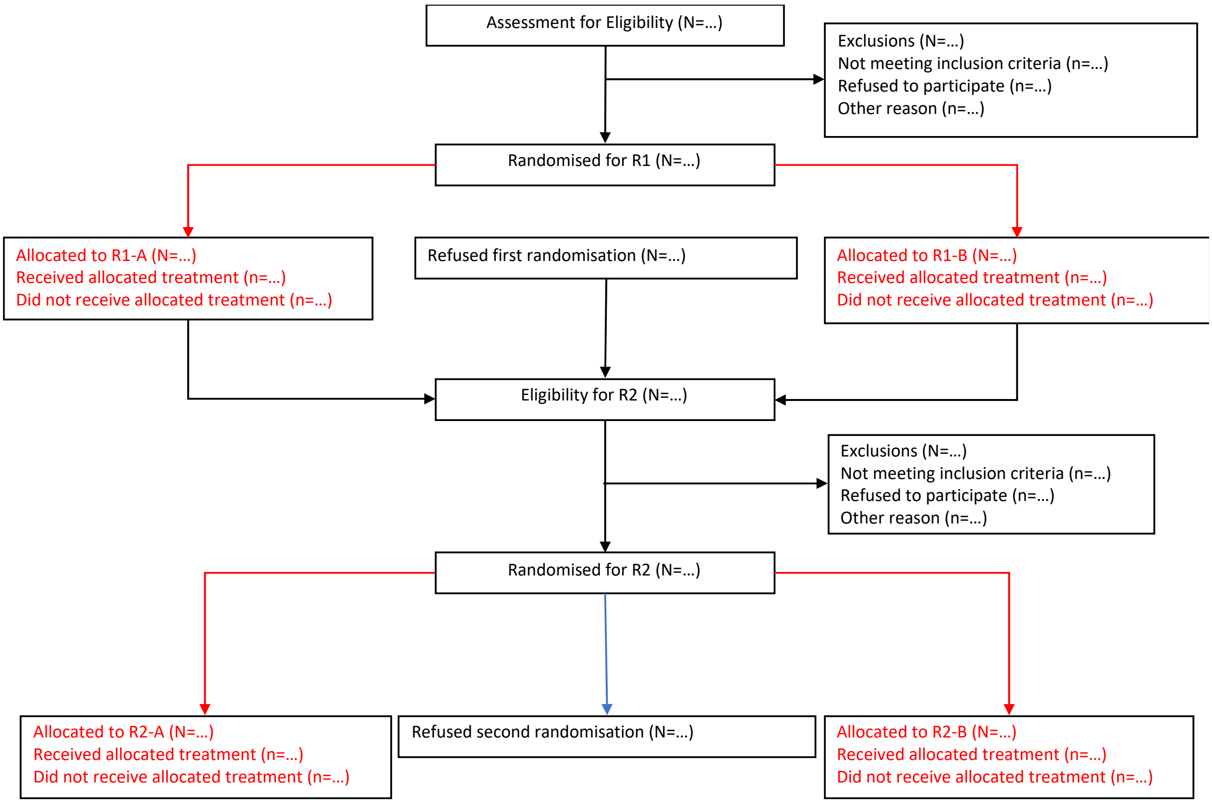

Note: Final analysis needs to also include patients lost to follow up in each arm

A separate table to accompany each section to include reasons for exclusion, e.g. not meeting exclusion criteria; refused to participate; other reasons

14.2 Baseline characteristics

Baseline characteristics will be compared between randomised groups to check that they are similar. Characteristics to be described in tabular form are as follows:

Table 1

**Variables used to define risk groups**	**R1A**	**R1B**	**R2A**	**R2B**
Age at diagnosis				
WC at diagnosis				
Sex				
CNS3				
Bulky disease				
T-ALL				
Prednisolone poor response				
Cytogenetics				
CR Rates				
Pre-Treatment risk group				
MRD Good				
MRD Poor				
Post Treatment Risk				

Each of the characteristics shown above will be summarised by showing number and percentage for R-A vs R-B. Numbers (with percentages) for binary and categorical variables and means (and standard deviations), or medians (with lower and upper quartiles) for continuous variables will be presented. The trial statistician, DSMC and TMG will consider if an imbalance is likely to cause concern and will agree an action accordingly. There will be no tests of statistical significance nor confidence intervals for differences between randomised groups on any baseline variable because the treatment arms will be allocated at random, then any difference are due to chance variation and can lead to false assumptions of significant or non-significant differences.

The follow-up will be summarised within each treatment group for each time to event endpoint using the median from the reverse Kaplan-Meier method and the median follow-up for those without an event.

14.3 Comparison of Losses to Follow-up

The numbers and distribution of losses to follow-up (defaulters and withdrawals) over the period of the study will be reported overall and for each randomisation. For each randomisation, analyses reporting median follow up and number lost to follow up or withdrawn to treatment for patients.

For time to event analyses, the baseline characteristics of those lost to follow-up and those to be analysed will be summarised for each randomisation. No test of significance will be used. Those randomised may be balanced, but those with data to analyse on the secondary objectives may not be. The consequences of this examination of the data will be described separately for each objective.

15. Primary Analyses

All validated data will be stored in the database. After termination of the study and after completion of all entries, the database will be closed for further entries. This process will be documented.

15.1 Randomisation 1

The differences in sepsis rates, as a measure of toxicity will will be calculated using the x^2 test. The data will be presented in a tabular form. Increased sepsis rates are expected in the IR arm as these patients receive daunorubicin. Separate sub cohort analyses will be performed to explore the differences in events in the SR and IR patient as secondary analyses. Differences in all other toxicities will be descriptive and differences between the categorical variables using x^2 test or Fishers test as appropriate.

15.2 Randomisation 2

Kaplan-Meier plots will be generated for EFS. A Cox analysis of treatment effect on EFS adjusting for the factors used in the randomisation stratification to remove possible bias between arms due to chance unbalance and to give more statistical power and unbiased results.

15.3 Survival analyses

Kaplan-Meier plots will be generated for EFS. A Cox analysis of treatment effect on EFS adjusting for the factors used for stratification to remove possible bias between arms due to chance unbalance and to give more statistical power and unbiased results.

A Kaplan-Meier plot will be drawn with a line for each treatment combination in the randomised groups to look for a visual sign of interaction between the treatments. An interaction term will also be added to the Cox models containing both of the treatment main effect to test for significance (although this will have low power). If statistically significant interactions are found, the results will also be presented for R2 within each R1 treatment group.

Predefined subgroup analyses of EFS will include all variables listed in Table 1.

Regression analysis will be multivariate regression, including as covariates the factors used in stratification (risk group), and other factors expected to be prognostic (cytogenetics, age, sex and MRD). Only randomised patients will be used for this analysis.

15.3 Missing Data

In randomisation 1, data is collected from the first 5 weeks of therapy. Given that analyses is done at the end of treatment, we anticipate that data will be available on most patients. Randomisation 2 is a time to event analyses. An annual follow up form is part of the study and data should be available on most patients except those lost to follow up.

The numbers and distribution of losses to follow-up (defaulters and withdrawals) over the period of the study will be reported overall and for each randomisation. For each randomisation, analyses reporting median follow up and number lost to follow up or withdrawn to treatment for patients.

For time to event analyses, the baseline characteristics of those lost to follow-up and those to be analysed will be summarised for each randomisation. No test of significance will be used. Those randomised may be balanced, but those with data to analyse on the secondary objectives may not be. The consequences of this examination of the data will be described separately for each objective.

16. Secondary Analyses

Kaplan-Meier plots will be generated for OS whole cohort, by randomisation, by risk stratification, by participating centre and prognostic subgroups.

Toxicity of each phase of the study will be tabulated and presented as percentage of each toxicity occurring per number of patients in one defined phase of treatment. Multiple reports of toxicity in one phase in the same patient will be counted as a single event. Comparison of incidences of SAE, toxicities, treatment related deaths and second malignancy between groups using x^2 test or Fisher's exact test as appropriate. between A p-value adjustment for multiple testing will be made using Bonferroni corrections to protect against making false positive conclusions.

CR and MRD rates, adjusted for factors contributing to risk stratification in the randomised groups will be tabulated and differences analysed using Mann-Whitney or ANOVA as appropriate.

17. Reporting of adverse events

Serious adverse events (SAE) are defined in the protocol. Febrile neutropenia is not classified as an SAE but reported as an adverse event (AE) or toxicity.
